# Iridium-Catalyzed Borylation of 6-Fluoroquinolines: Access to 6-Fluoroquinolones

**DOI:** 10.1021/acs.joc.2c00973

**Published:** 2022-07-15

**Authors:** Aobha Hickey, Julia Merz, Hamad H. Al Mamari, Alexandra Friedrich, Todd B. Marder, Gerard P. McGlacken

**Affiliations:** † School of Chemistry & Analytical and Biological Chemistry Research Facility, University College Cork, Cork T12 YN60, Ireland; ‡ Institute for Inorganic Chemistry, and Institute for Sustainable Chemistry & Catalysis with Boron, Julius-Maximilians-Universität Würzburg, Am Hubland, 97074 Würzburg, Germany; § Department of Chemistry, College of Science, Sultan Qaboos University, P.O. Box 36, Al Khoudh 123 Muscat, Sultanate of Oman; ∥ Synthesis and Solid State Pharmaceutical Centre, University College Cork, Cork T12 YN60, Ireland

## Abstract

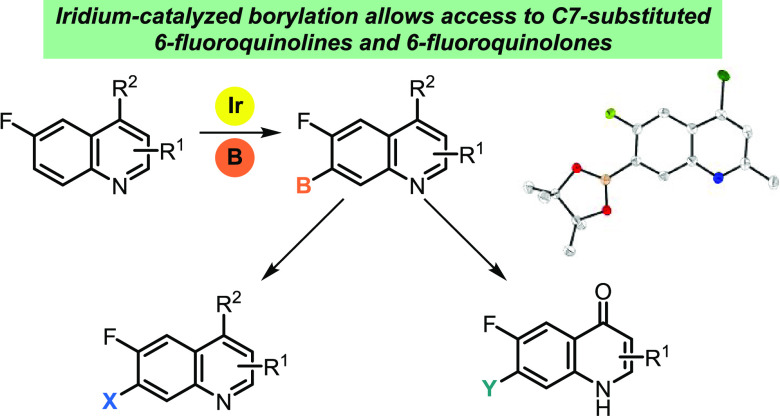

The Ir-catalyzed C–H borylation of fluoroquinolines has been realized. The quinoline boronic ester formed undergoes a range of important transformations of relevance to medicinal chemistry. Judicious choice of the substituent at C4 on the quinoline facilitated the unmasking of a fluoroquinolone—the core structure of many antibiotics.

## Introduction

**Scheme 1:**
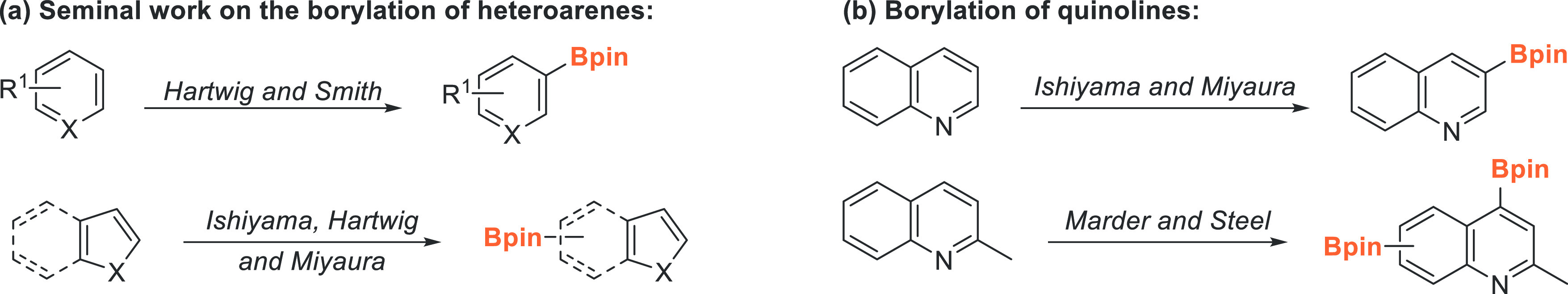
Iridium-Catalyzed Borylation of Quinolines

The C–H bond functionalization of heteroarenes has emerged as an important synthetic methodology,^1^ considering the roles that heteroarenes play in pharmaceuticals, agrochemical products, and electronic materials.^2^ Iridium-catalyzed C–H borylation has proven to be a useful method for the functionalization of heteroarenes because of its ability to produce highly versatile aryl organoboronate ester intermediates without the need for reactive groups, such as halides or sulfonates (Scheme 1a).^3^ In particular, the use of [Ir(OMe)COD]_2_ with bidentate ligands such as 4,4′-di-*tert*-butyl-2,2′-dipyridyl (dtbpy) has emerged as a powerful methodology for the borylation of arenes using both bis(pinacolato)diboron (B_2_pin_2_) and pinacolborane (HBpin).^3,4^ Given its role as a key scaffold in a plethora of synthetic and naturally occurring pharmacologically active compounds,^5^ the quinoline-nucleus has been used in a number of borylation-based methodologies. However, expansion of quinoline C–H borylation strategies requires additional developments regarding the regioselectivity control (Scheme 1b).^3l,3n,6^

**Figure 1:**
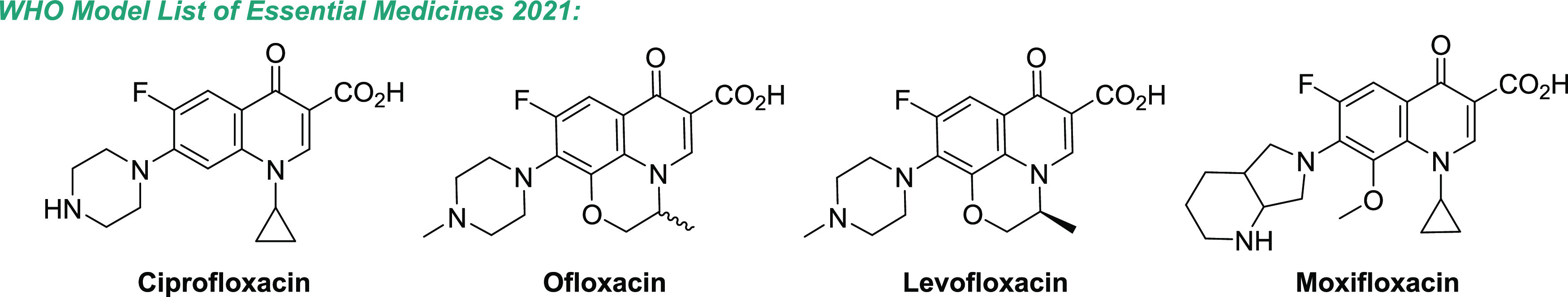
Fluoroquinolone antibiotics contained on the WHO Model List of Essential Medicines 2021.

Fluoroquinolone antibiotics are one of the world’s most commonly prescribed classes of antimicrobials^7^ and are among the World Health Organization (WHO) Model List of Essential Medicines (Figure 1).^8^ Their bioavailability, broad-spectrum activity, and potency profiles have established 6-fluoroquinolones as the treatment of choice for a variety of infections, including urinary tract, soft tissue, and gastrointestinal infections.^9^ Synthesis of the core quinolone structure (and various analogues) is normally achieved through cyclization processes, carried out at elevated temperatures from the corresponding (substituted) aniline and an unsaturated coupling partner.^10^ These preparations are still widely employed but do suffer from a number of issues. The processes are dependent on the availability of highly functionalized starting materials and thus are not amenable to late-stage derivatization.^11^ In addition, the cyclization process can result in the formation of regioisomers. This problem is particularly evident in the case of C-5/C-7 substitution of the quinolone, where the *meta*-substituted starting aniline can cyclize at either *ortho*-position.^10a^ However, considerable strides have also been made toward catalytic processes using halogenated precursors.^12^

**Scheme 2:**
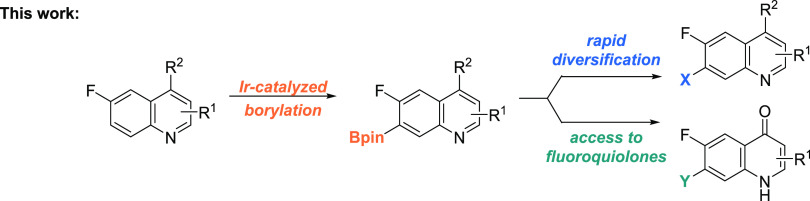
Iridium-Catalyzed Borylation of Substituted Quinolines and Subsequent Access to 6-Fluoroquinolones

Herein, we describe the C7–H borylation of 6-fluoroquinolines. We hoped that the fluorine atom would serve to guide the borylation to the C7 position^13^ and also act as a critical functional group, especially if a quinoline to quinolone transformation could be developed (Scheme 2).^9d,14^

## Results and Discussion

We initiated our optimization experiments using fluoroquinoline **1a** with [Ir(OMe)COD]_2_, dtbpy, and B_2_pin_2_ (Table 1, see the Supporting Information (SI) for more detail). Although tetrahydrofuran (THF) proved to be the most effective solvent, it is worth noting the very good conversion achieved using methyl *tert*-butyl ether (MTBE), an easy to handle, relatively nonperoxidizable ether, which is already widely used in the industry, as the solvent (entries 2 and 3). No conversion to the borylated product was observed in cyclopentyl methyl ether (CPME) (entry 1). As a ligand, 1,10-phenanthroline (phen) also proved useful for this transformation (entries 5 and 6), but the superior reactivity profile of dtbpy is clearly demonstrated under otherwise identical conditions (entry 7). Optimized conditions allowed the formation of **2a** in a 98% yield (by ^1^H NMR analysis, entry 9).

**Table 1:** Optimization of the Borylation of **1a**

entry	B_2_pin_2_ (equiv)	Ir cat. (mol %)	ligand (mol %)	solvent^a^ (mL)	yield (%)^b^
1	1.1	3.0	dtbpy 6.0	CPME 3	0
2	1.1	3.0	dtbpy 6.0	MTBE 3	70
3	1.1.	1.5	dtbpy 3.0	MTBE 1	95
4	1.5	3.0	dtbpy 6.0	THF 3	88
5	1.1	3.0	phen 6.0	THF 3	80
6	1.5	3.0	phen 6.0	THF 3	91
7	1.1	3.0	dtbpy 6.0	THF 3	>99
8	0.75	3.0	dtbpy 6.0	THF 1	89
9	1.1	1.5	dtbpy 3.0	THF 1	98

a Reactions carried out on the 0.2 mmol scale. Reaction temperatures are as follows: CPME 100 °C; MTBE 60 °C; THF 80 °C.

b Yields calculated from ^1^H NMR analysis of the crude reaction mixture using 1,3,5-trimethoxybenzene as an internal standard.

**Scheme 3:**
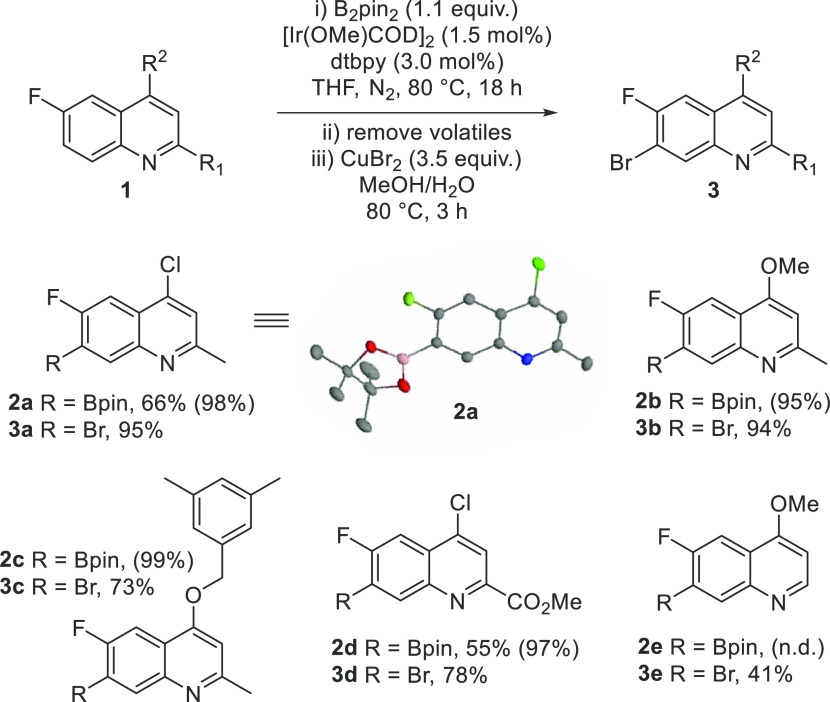
Borylated and Brominated Quinoline Substrates^,^ Yields are isolated. Yields in parentheses calculated from ^1^H NMR analysis of the crude reaction mixture using 1,3,5-trimethoxybenzene as an internal standard. n.d. = not determined, see the SI for more information.

Substrates for borylation were then selected with varying groups at C4 and either an H, Me, or ester group at C2 (Scheme 3). In our choice of groups at C4, we were conscious that a quinoline to quinolone transformation would be very valuable as this methodology could provide an excellent route to substituted fluoroquinolones. We anticipated that compounds **2a**, **2b**, **2d**, and **2e** could undergo acid-hydrolysis to provide the corresponding 4-quinolone,^15^ while orthogonally, **2c** could be deprotected using palladium-catalyzed hydrogenation (*vide infra*).^16^

The borylated quinolines were unstable on silica gel and proved difficult to purify.^3l^ However, we were able to isolate compounds **2a** and **2d** in good yields by recrystallization from methanol. Generally, the intermediate borylation steps gave very good yields (by ^1^H NMR analysis, using an internal standard), with the exception of **2e**; here, the reduction in yield is attributed to **1e** having a competitive site for borylation (see the SI). In any case, and conveniently, the crude material could be converted to the C7-brominated products,^17^ which were purified and characterized (Scheme 3). The C7 selectivity of the C–H borylation was confirmed by the molecular structure of **2a** obtained by single-crystal X-ray diffraction.

**Scheme 4:**
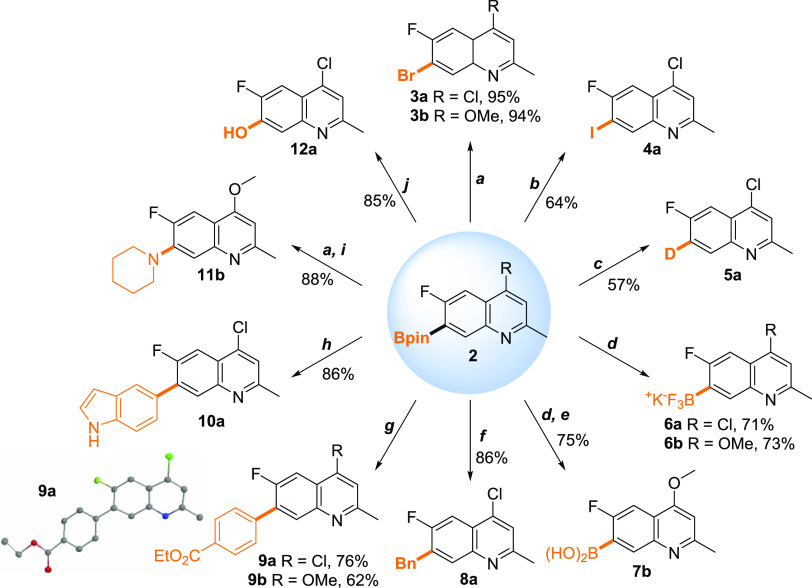
Synthetic Transformations of C7-Borylated Quinolines **Conditions**: (a) CuBr_2_ (3.5 equiv), MeOH/H_2_O (1:1 v/v), 80 °C, 3 h. (b) CuI (10 mol %), 1,10-phenanthroline (20 mol %), KI (1.5 equiv), MeOH/H_2_O (4:1 v/v), N_2_, 80 °C, 2 h. (c) [Ir(OMe)COD]_2_ (1 mol %), THF/D_2_O (4:1 v/v), N_2_, 80 °C, 12 h. (d) KHF_2_ (6.0 equiv), THF/H_2_O (3:1 v/v), rt, 16 h. (e) LiOH·H_2_O (9.0 equiv), THF/H_2_O (5:1 v/v), rt, 24 h. (f) Pd_2_(dba)_3_ (1 mol %), PPh_3_ (4 mol %), K_2_CO_3_ (4.0 equiv), BnBr (1.2 equiv), THF/H_2_O (50:1 v/v), N_2_, 100 °C, 24 h. (g) Pd(PPh_3_)_4_ (5 mol %), K_3_PO_4_ (3.0 equiv), ethyl-4-bromobenzoate (1.5 equiv), THF/H_2_O (5:1 v/v), N_2_, 60 °C, 18 h. (h) Pd(dba)_2_ (2 mol %), P(o-tol)3 (6 mol %), Na_2_CO_3_ (4.0 equiv), 5-bromoindole (0.8 equiv), THF/H_2_O (10:1 v/v), N_2_, 50 °C, 24 h. (i) Pd_2_(dba)_3_ (3 mol %), RuPhos (6 mol %), NaOtBu (2.5 equiv), Piperidine (2.0 equiv), Toluene, N_2_, 80 °C, 16 h. (j) 30% H_2_O_2_ (1.2 equiv), MeOH, 0 °C to rt, 16 h.

In our experience and that of others,^18^ quinolones tend to suffer from solubility issues, especially those lacking pendant organic groups. Here, however, the apparently increased solubility of the quinoline motif meant that the borylated quinoline compounds could undergo a range of synthetically valuable transformations (Scheme 4). Given the commonly encountered issues associated with employing heterocycles as substrates for cross-coupling and other reactions,^19^ it was critically important to demonstrate that the Bpin moiety could be transformed into useful substituted quinolines.

The *in situ* borylated quinolines **2** were converted to the bromo-quinoline analogues in excellent yields over two steps using copper bromide (a). The iodinated compound was accessed in a moderate yield using copper iodide (b), again over two steps. The purified C7-borylated product was converted to the deuterio and hydroxy analogues in good yields (c, j). The useful and more reactive trifluoroborate salt was obtained using potassium bifluoride in aqueous THF (d), and the boronic acid motif was subsequently accessed after reaction of the salt with lithium hydroxide in aqueous THF (e). The borylated quinoline substrate **2a** was coupled with a number of brominated substrates in high-yielding, palladium-catalyzed Suzuki–Miyaura reactions (f, g, h). Finally, amination of the C7 position was achieved *via* a Hartwig–Buchwald coupling reaction of the brominated substrate **3b** with piperidine (i).

**Scheme 5:**
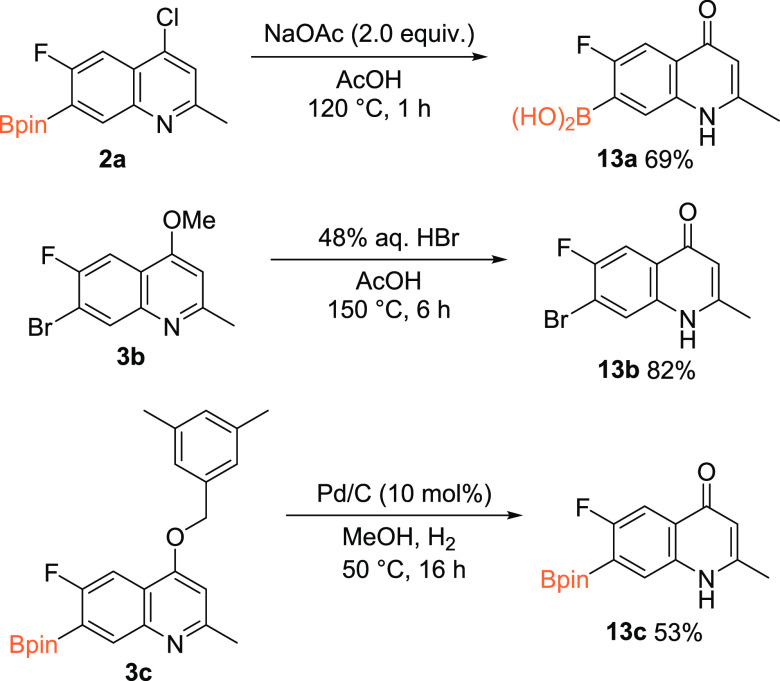
Access to Substituted Fluoroquinolones

While this borylation protocol provides excellent access to a number of substituted quinolines, underpinning our choices for the moiety at C4 was the potential to furnish the quinolone scaffold, thus gaining access to some fluoroquinolones. Indeed, 4-Cl borylated quinoline **2a** and 4-OMe brominated quinoline **3b** were converted to the corresponding quinolones **13a** and **13b** in 68 and 82% yields, respectively. In the case of **2a**, concurrent hydrolysis to the boronic acid **13a** was observed. Debenzylation of compound **3c** was achieved *via* palladium-catalyzed hydrogenolysis in methanol (Scheme 5).

**Scheme 6:**
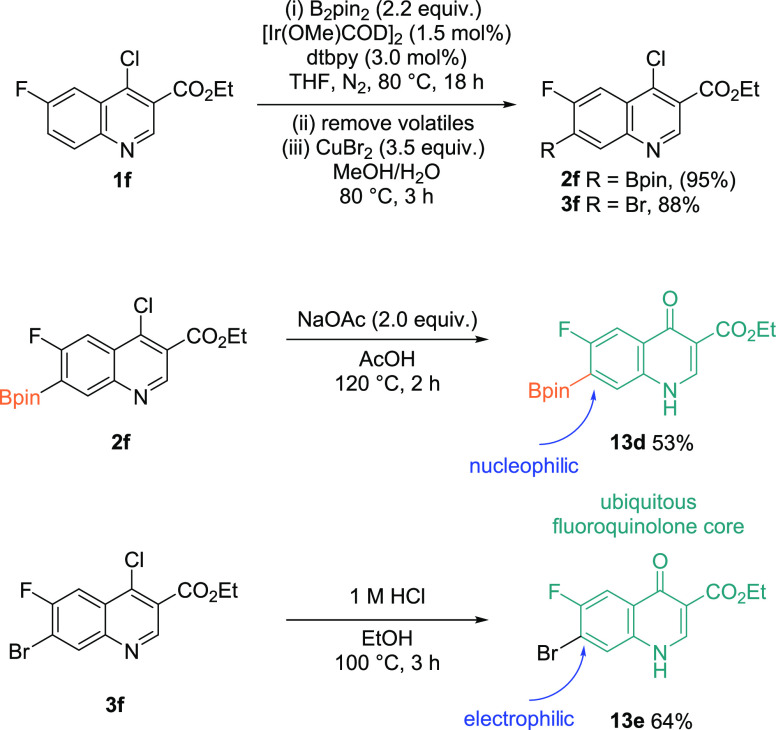
Synthesis of the 6-Fluoroquinolone Antibiotic Framework Yields are isolated. Yields in parentheses calculated from ^1^H NMR analysis of the crude reaction mixture using 1,3,5-trimethoxybenzene as an internal standard.

Finally, in the context of mapping onto the core antibiotic fluoroquinolone structure, initial attempts using our optimized conditions (Table 1) with **1f** gave only trace amounts of the borylated product. However, using 2.2 equiv of B_2_pin_2_ allowed the borylation to **2f** to occur in a very good yield, and subsequent bromination gave **3f** in a yield of 88% (Scheme 6). Finally, the key fluoroquinolone framework was furnished by acid-catalyzed hydrolysis of **2f** to give the boronate ester **13d** (C7 = nucleophilic) in a 53% yield. The complementary brominated fluoroquinolone moiety (C7 = electrophilic) was also accessible by alcoholysis of **3f** to give **13e** in a 64% yield.

## Conclusions

In summary, we have developed an efficient protocol for the C–H functionalization/borylation of quinolines incorporating useful and useable substituents. Importantly, borylation is selective for C7, avoiding, for example, C3. Extensive diversification at C7 is demonstrated in the presence of the basic quinoline group. Finally, the ubiquitous fluoroquinolone moiety can be generated in a simple hydrolysis step.

## Experimental Section

### General Considerations

The catalyst precursor [Ir(OMe)COD]_2_ was synthesized according to a literature procedure^20^ and was stored in a glovebox under an argon atmosphere. Toluene and CPME were dried and stored over flame-dried 4 Å molecular sieves. THF was either freshly distilled from sodium/benzophenone under a nitrogen atmosphere and stored over molecular sieves under the nitrogen atmosphere (University College Cork) or dried using a solvent purification system (SPS) from Innovative Technology, degassed with argon, and stored over molecular sieves under the argon atmosphere (Julius-Maximilians-Universität Würzburg). K_2_CO_3_ and Na_2_CO_3_ were stored in an oven at 150 °C. All other solvents and reagents were used as obtained from commercial sources and without further purification. Melting points were measured using a Thomas Hoover Capillary Melting Point apparatus. Infrared spectra were measured on a PerkinElmer FT-IR spectrometer as thin films in DCM. Column chromatography was carried out using 60 Å (35–70 μm) silica. TLC was carried out on precoated silica gel plates (Merck 60 PF254), and the developed plates were visualized under UV light. High-resolution mass spectra (HRMS) were recorded on a Waters LCT Premier Tof LC-MS instrument in electrospray ionization (ESI) mode (University College Cork) or using a Thermo Scientific Exactive Plus Orbitrap MS system with an Atmospheric Sample Analysis Probe (ASAP) (Julius-Maximilians-Universität Würzburg). Samples were run using 50% acetonitrile–water containing 0.1% formic acid as the eluent and were prepared at a concentration of *ca*. 1 mg mL^–1^. Nuclear magnetic resonance (NMR) spectra were recorded in CDCl_3_, (CD_3_)_2_SO, or CD_3_OD, as specified. ^1^H NMR (600 MHz), ^1^H NMR (500 MHz), ^1^H NMR (400 MHz), and ^1^H NMR (300 MHz) spectra were recorded on Bruker Avance 600, Bruker Avance 500, Bruker Avance 400, and Bruker Avance III 300 NMR spectrometers, respectively. ^13^C NMR (150 MHz), ^13^C NMR (125 MHz), ^13^C NMR (100 MHz), and ^13^C NMR (75 MHz) spectra were recorded on Bruker Avance 600, Bruker Avance 500, Bruker Avance 400, and Bruker Avance III 300 NMR spectrometers, respectively, in proton decoupled mode. ^19^F NMR (470 MHz), ^19^F NMR (376 MHz), and ^19^F NMR (282 MHz) spectra were recorded on Bruker Avance 500, Bruker Avance 400, and Bruker Avance III 300 NMR spectrometers, respectively, in proton decoupled mode. All NMR analyses were carried out at 300 K unless otherwise specified. Chemical shifts (δ) are expressed as parts per million (ppm) and coupling constants (*J*) are expressed in Hertz (Hz).

### Synthesis of Quinoline Starting Materials

#### 4-Chloro-6-fluoro-2-methylquinoline (**1a**)^21^

6-Fluoro-2-methylquinolin-4(1*H*)-one (1.77 g, 10 mmol, 1.0 equiv) was added to a Schlenk flask, dissolved in POCl_3_ (4.6 mL, 50 mmol, 5.0 equiv), and the mixture was stirred in an oil bath at 110 °C for 3 h. The cooled reaction mixture was slowly added to iced water (20 mL) with stirring. Solid NaHCO_3_ was then added gradually until the pH reached ca.∼ 7, and the mixture was allowed to stir until effervescence ceased. The mixture was transferred to a separating funnel, and the organic layer was collected. The aq. layer was extracted with DCM (3 × 20 mL). The combined organic layers were washed with saturated NaHCO_3_ (20 mL), H_2_O (20 mL), and brine (20 mL), dried over MgSO_4_, filtered, and concentrated under reduced pressure. White solid (1.860 g, 95%); m.p. 84–86 °C (lit.^22^ 83–84 °C); ^1^H NMR (300 MHz, CDCl_3_) δ: 8.02 (dd, *J* = 9.2, 5.3 Hz, 1H), 7.79 (dd, *J* = 9.4, 2.8 Hz, 1H), 7.49 (ddd, *J* = 9.2, 8.3, 2.9 Hz, 1H), 7.41 (s, 1H), 2.71 (s, 3H) ppm; ^13^C{^1^H} NMR (75 MHz, CDCl_3_) δ: 160.7 (d, *J* = 248 Hz), 158.2 (d, *J* = 3 Hz), 145.7, 141.7 (d, *J* = 6 Hz), 131.6 (d, *J* = 9 Hz), 125.6 (d, *J* = 10 Hz), 122.5, 120.5 (d, *J* = 26 Hz), 107.8 (d, *J* = 24 Hz), 25.0 ppm; ^19^F NMR (282 MHz, CDCl_3_) δ: −112 ppm; *m*/*z* (ES+): 196 ((M + H)^+^ 100%).

#### 6-Fluoro-4-methoxy-2-methylquinoline (**1b**)

4-Chloro-6-fluoro-2-methylquinoline **1a** (500 mg, 2.6 mmol, 1.0 equiv) in MeOH (8 mL) was added dropwise to a freshly prepared solution of sodium (20 mmol, 8.0 equiv) in MeOH (12 mL) over ice. The mixture was warmed to r.t. and then heated to 80 °C in an oil bath for 18 h, cooled to room temperature, and concentrated under reduced pressure. The residue was dissolved in a mixture of EtOAc/H_2_O (1:1, 20 mL), and the layers were separated. The aqueous layer was extracted with EtOAc (2 × 10 mL), and the combined organic layers were then washed with H_2_O (10 mL) and brine (15 mL), dried over MgSO_4_, filtered, and concentrated under reduced pressure. Beige solid (0.473 g, 97%); m.p. 52–54 °C; IR (film) ν_max_ 1632, 1514, 1353, 1201, 1180 cm^–1^; ^1^H NMR (300 MHz, CDCl_3_) δ: 7.92 (dd, *J* = 9.2, 5.2 Hz, 1H), 7.71 (dd, *J* = 9.5, 2.9 Hz, 1H), 7.40 (ddd, *J* = 9.2, 8.3, 2.9 Hz, 1H), 6.62 (s, 1H), 4.00 (s, 3H), 2.68 (s, 3H) ppm; ^13^C{^1^H} NMR (75 MHz, CDCl_3_) δ: 161.8 (d, *J* = 5 Hz), 159.7 (d, *J* = 245 Hz), 159.3 (d, *J* = 2 Hz), 145.7, 130.4 (d, *J* = 9 Hz), 120.4 (d, *J* = 9 Hz), 119.5 (d, *J* = 25 Hz), 105.6 (d, *J* = 23 Hz), 101.0, 55.6, 25.8 ppm; ^19^F NMR (282 MHz, CDCl_3_) δ: −116 ppm; HRMS (ESI-TOF) *m*/*z*: [M + H]^+^ calcd for C_11_H_11_FNO: 192.0819; found: 192.0817.

#### 4-((3,5-Dimethylbenzyl)oxy)-6-fluoro-2-methylquinoline (**1c**)

To a solution of 6-fluoro-2-methylquinolin-4(1*H*)-one (265.8 mg, 1.5 mmol, 1.0 equiv) in DMF (30 mL) was added K_2_CO_3_ (414.6 mg, 3.0 mmol, 2.0 equiv). The mixture was stirred at 40 °C for 1 h before the portion-wise addition of 3,5-dimethylbenzyl bromide (358.4 mg, 1.8 mmol, 1.2 equiv), and the resulting suspension was heated to 60 °C in an oil bath for 16 h. The mixture was poured onto ice water and extracted with EtOAc (3 × 20 mL). The combined organic layers were washed with water (2 × 20 mL), dried over MgSO_4_, filtered, and concentrated under reduced pressure. The product was purified *via* column chromatography (DCM/EtOAc, 90:10). White solid (0.373 g, 84%); m.p. 93–95 °C; IR (film) ν_max_ 1604, 1514, 1478, 1348, 1185, 1083 cm^–1^; ^1^H NMR (500 MHz, CDCl_3_) δ: 7.94 (dd, *J* = 9.2, 5.2 Hz, 1H), 7.79 (dd, *J* = 9.5, 2.9 Hz, 1H), 7.41 (ddd, *J* = 9.2, 8.2, 2.9 Hz, 1H), 7.08 (s, 2H), 7.02 (bs, 1H), 6.70 (s, 1H), 5.17 (s, 2H), 2.68 (s, 3H), 2.36 (s, 6H) ppm; ^13^C{^1^H} NMR (125 MHz, CDCl_3_) δ: 161.1 (d, *J* = 5 Hz), 159.7 (d, *J* = 245 Hz), 159.3 (d, *J* = 2 Hz), 145.8, 138.5, 135.4, 130.4 (d, *J* = 9 Hz), 130.2, 125.4, 120.6 (d, *J* = 10 Hz), 119.6 (d, *J* = 25 Hz), 105.8 (d, *J* = 23 Hz), 102.0, 70.5, 25.8, 21.3 ppm; ^19^F NMR (470 MHz, CDCl_3_) δ: −115 ppm; HRMS (ESI-TOF) *m*/*z*: [M + H]^+^ calcd for C_19_H_19_FNO: 296.1445; found: 296.1451.

#### Methyl 4-chloro-6-fluoroquinoline-2-carboxylate (**1d**)

Prepared *via* the method described for compound **1a** using methyl 6-fluoro-4-oxo-1,4-dihydroquinoline-2-carboxylate (221.2 mg, 1.0 mmol, 1.0 equiv). White solid (0.230 g, 96%); m.p. 140–141 °C; IR (film) ν_max_ 1751, 1623, 1558, 1208, 1104, 1006, 831 cm^–1^; ^1^H NMR (300 MHz, CDCl_3_) δ: 8.42–8.25 (m, 2H), 7.89 (dd, *J* = 9.2, 2.8 Hz, 1H), 7.62 (ddd, *J* = 9.2, 8.0, 2.8 Hz, 1H), 4.09 (s, 3H) ppm; ^13^C{^1^H} NMR (75 MHz, CDCl_3_) δ: 164.8, 162.5 (d, *J* = 254 Hz), 147.1 (d, *J* = 3 Hz), 145.3, 143.1 (d, *J* = 6 Hz), 134.0 (d, *J* = 10 Hz), 128.8 (d, *J* = 11 Hz), 121.9, 121.8 (d, *J* = 26 Hz), 108.0 (d, *J* = 25 Hz), 53.5 ppm; ^19^F NMR (282 MHz, CDCl_3_) δ: −107 ppm; HRMS (ESI-TOF) *m*/*z*: [M + H]^+^ calcd for C_11_H_8_ClFNO_2_: 240.0222; found: 240.0221.

#### 6-Fluoro-4-methoxyquinoline (**1e**)

Prepared *via* the method described for compound **1b** using 4-chloro-6-fluoroquinoline (320 mg, 1.76 mmol, 1.0 equiv). White solid (0.295 g, 95%); m.p. 50–53 °C; IR (film) ν_max_ 1631, 1598, 1309, 1189, 1069 cm^–1^; ^1^H NMR (500 MHz, CDCl_3_) δ: 8.71 (d, *J* = 5.0 Hz, 1H), 8.02 (dd, *J* = 9.2, 5.3 Hz, 1H), 7.77 (dd, *J* = 9.5, 2.9 Hz, 1H), 7.44 (ddd, *J* = 9.2, 8.2, 2.9 Hz, 1H), 6.73 (d, *J* = 5.1 Hz, 1H), 4.02 (s, 3H) ppm; ^13^C{^1^H} NMR (125 MHz, CDCl_3_) δ: 162.0 (d, *J* = 5 Hz), 160.2 (d, *J* = 247 Hz), 150.7 (d, *J* = 2 Hz), 146.3, 131.4 (d, *J* = 9 Hz), 122.2 (d, *J* = 10 Hz), 119.8 (d, *J* = 26 Hz), 105.8 (d, *J* = 24 Hz), 100.5, 55.8 ppm; ^19^F NMR (282 MHz, CDCl_3_) δ: −114 ppm; HRMS (ESI-TOF) *m*/*z*: [M + H]^+^ calcd for C_10_H_9_FNO: 178.0663; found: 178.0658.

#### Ethyl-4-chloro-6-fluoroquinoline-3-carboxylate (**1f**)^23^

Prepared *via* the method described for compound **1a** using ethyl 6-fluoro-4-oxo-1,4-dihydroquinoline-3-carboxylate (352.8 mg, 1.5 mmol, 1.0 equiv). White solid (0.352 g, 93%); m.p. 67–69 °C (lit.^24^ 62–63 °C); ^1^H NMR (600 MHz, CDCl_3_) δ: 9.16 (s, 1H), 8.17 (dd, *J* = 9.2, 5.3 Hz, 1H), 8.03 (dd, *J* = 9.6, 2.7 Hz, 1H), 7.62 (ddd, *J* = 9.2, 7.9, 2.8 Hz, 1H), 4.51 (q, *J* = 7.1 Hz, 2H), 1.47 (q, *J* = 7.1 Hz, 3H) ppm; ^13^C{^1^H} NMR (150 MHz, CDCl_3_) δ: 163.7, 161.1 (d, *J* = 251 Hz), 148.8, 145.9, 142.1 (d, *J* = 6 Hz), 131.9 (d, *J* = 9 Hz), 126.9 (d, *J* = 7 Hz), 123.1, 121.7 (d, *J* = 26 Hz), 108.7 (d, *J* = 25 Hz), 61.7, 13.7 ppm; ^19^F NMR (282 MHz, CDCl_3_) δ: −109 ppm; *m*/*z* (ES+): 254 ((M + H)^+^ 100%).

### Borylation of 6-Fluoroquinolines

#### General Procedure for the Borylation of 6-Fluoroquinolines

A 15 mL Schlenk flask was oven-dried (150 °C) and cooled under vacuum. The Schlenk flask was refilled with nitrogen, and all reagents were added under a positive pressure of nitrogen in the order: quinoline (1.0 equiv), dtbpy (3 mol %), B_2_pin_2_ (1.1 equiv), and [Ir(OMe)COD]_2_ (1.5 mol %). The Schlenk flask was then placed under vacuum for 20 min before being refilled with nitrogen 3 times. THF (2.5 mL/mmol) was added *via* a syringe through the septum, the reaction was sealed, and the mixture was heated to 80 °C in an aluminum heating block for 12–18 h. The reaction mixture was then cooled to r.t., diluted with MeOH (20 mL/mmol), and concentrated under reduced pressure. The product was purified *via* recrystallization from hot MeOH.

#### 4-Chloro-6-fluoro-2-methyl-7-(4,4,5,5-tetramethyl-1,3,2-dioxaborolan-2-yl)quinoline (**2a**)

Prepared *via* the general procedure using **1a** (391.2 mg, 2.0 mmol, 1.0 equiv); X-ray quality crystals were obtained *via* vapor diffusion from a saturated solution of DCM in Et_2_O; and the CCDC number is 2159956. White solid (0.422 g, 66%); m.p. 97–100 °C; IR (film) ν_max_ 1627, 1500, 1370, 1331, 1261, 1147, 1049, 854 cm^–1^; ^1^H NMR (600 MHz, CDCl_3_) δ: 8.56 (d, *J* = 5.5 Hz, 1H), 7.79 (d, *J* = 9.7 Hz, 1H), 7.47 (s, 1H), 2.77 (s, 3H), 1.45 (s, 12H) ppm; ^13^C{^1^H} NMR (150 MHz, CDCl_3_) δ: 164.0 (d, *J* = 251 Hz), 158.1 (d, *J* = 3 Hz), 145.1, 141.6 (d, *J* = 5 Hz), 139.8 (d, *J* = 9 Hz), 127.6 (d, *J* = 11 Hz), 123.2, 107.5 (d, *J* = 27 Hz), 84.5, 25.0, 24.9 ppm; a signal for the carbon directly attached to the boron atom was not observed; ^19^F NMR (282 MHz, CDCl_3_) δ: −105 ppm; ^11^B NMR (96 MHz, CDCl_3_) δ: 30 ppm; HRMS (ESI-TOF) *m*/*z*: [M + H]^+^ calcd for C_16_H_19_BClFNO_2_: 322.1176; found: 322.1182.

#### Methyl 4-chloro-6-fluoro-7-(4,4,5,5-tetramethyl-1,3,2-dioxaborolan-2-yl)quinoline-2-carboxylate (**2d**)

Prepared *via* the general procedure using **1d** (47.9 mg, 0.2 mmol, 1.0 equiv). White solid (0.040 g, 55%); m.p. 156–158 °C; IR (film) ν_max_ 1727, 1625, 1498, 1336, 1326, 1230, 1118, 1049, 849 cm^–1^; ^1^H NMR (600 MHz, CDCl_3_) δ: 8.84 (d, *J* = 5.6 Hz, 1H), 8.30 (s, 1H), 7.83 (d, *J* = 9.4 Hz, 1H), 4.09 (s, 3H), 1.40 (s, 12H) ppm; ^13^C{^1^H} NMR (150 MHz, CDCl_3_) δ: 165.7 (d, *J* = 257 Hz), 164.9, 147.1 (d, *J* = 3 Hz), 144.8, 142.8 (d, *J* = 6 Hz), 142.5 (d, *J* = 10 Hz), 130.5 (d, *J* = 11 Hz), 122.3, 107.7 (d, *J* = 28 Hz), 84.6, 53.4, 24.9 ppm; a signal for the carbon directly attached to the boron atom was not observed; ^19^F NMR (282 MHz, CDCl_3_) δ: −100 ppm; ^11^B NMR (96 MHz, CDCl_3_) δ: 30 ppm; HRMS (ESI-TOF) *m*/*z*: [M + H]^+^ calcd for C_17_H_19_BClFNO_4_: 366.1074; found: 366.1071.

### General Procedure for the Borylation and Subsequent Bromination of 6-Fluoroquinolines

A 15 mL Schlenk was oven-dried (150 °C) and cooled under vacuum. The Schlenk flask was refilled with nitrogen, and all reagents were added under a positive pressure of nitrogen in the order: quinoline (1.0 equiv), dtbpy (3 mol %), B_2_pin_2_ (1.1 equiv), and [Ir(OMe)COD]_2_ (1.5 mol %). The Schlenk flask was then placed under vacuum for 20 min before being refilled with nitrogen 3 times. THF (2.5 mL/mmol) was added *via* a syringe through the septum, the reaction was sealed, and the mixture was heated to 80 °C in an aluminum heating block for 12–18 h. The reaction mixture was then cooled to r.t., diluted with Et_2_O, and concentrated under reduced pressure. An internal standard 1,3,5-trimethoxybenzene (∼10 mol %) was added to the residue to determine the yield of the C7-borylated product **2a**–**2e** by ^1^H NMR analysis. The residue was redissolved in MeOH (20 mL/mmol), and a solution of CuBr_2_ (3.5 equiv) in H_2_O (20 mL/mmol) was added. The reaction mixture was heated to 80 °C in an oil bath for 3 h, cooled to r.t. diluted with 10% NH_4_OH (40 mL/mmol), and then extracted with Et_2_O (3 × 10 mL/mmol). The combined organic layers were washed with H_2_O (10 mL/mmol) and brine (10 mL/mmol), dried over MgSO_4_, filtered, and concentrated under reduced pressure. The product was purified *via* column chromatography (DCM/EtOAc gradient, unless otherwise specified).

#### 7-Bromo-4-chloro-6-fluoro-2-methylquinoline (**3a**)

Prepared *via* the general procedure using **1a** (39.1 mg, 0.2 mmol, 1.0 equiv). White solid (0.052 g, 95%); m.p. 100–101 °C; IR (film) ν_max_ 1612, 1588, 1016, 845, 707 cm^–1^; ^1^H NMR (600 MHz, CDCl_3_) δ: 8.27 (d, *J* = 6.6 Hz, 1H), 7.81 (d, *J* = 9.0 Hz, 1H), 7.40 (s, 1H), 2.70 (s, 3H) ppm; ^13^C{^1^H} NMR (150 MHz, CDCl_3_) δ: 158.8 (d, *J* = 3 Hz), 156.2 (d, *J* = 250 Hz), 145.2 (d, *J* = 1 Hz), 141.0 (d, *J* = 5 Hz), 133.6 (d, *J* = 1 Hz), 124.2 (d, *J* = 9 Hz), 122.2, 113.7 (d, *J* = 24 Hz), 108.1 (d, *J* = 26 Hz), 24.5 ppm; ^19^F NMR (282 MHz, CDCl_3_) δ: −108 ppm; HRMS (ESI-TOF) *m*/*z*: [M + H]^+^ calcd for C_10_H_7_BrClFN: 273.9429; found: 273.9424.

#### 7-Bromo-6-fluoro-4-methoxy-2-methylquinoline (**3b**)

Prepared *via* the general procedure using **1b** (49.8 mg, 0.2 mmol, 1.0 equiv). 95% yield **2b** by ^1^H NMR analysis using 1,3,5-trimethoxybenzene as an internal standard. White solid (0.051 g, 94%); m.p. 113–115 °C; IR (film) ν_max_ 1612, 1588, 1352, 1203, 1010, 726 cm^–1^; ^1^H NMR (400 MHz, CDCl_3_) δ: 8.23 (d, *J* = 6.5 Hz, 1H), 7.77 (d, *J* = 9.1 Hz, 1H), 6.64 (s, 1H), 4.03 (s, 3H), 2.69 (s, 3H) ppm; ^13^C{^1^H} NMR (100 MHz, CDCl_3_) δ: 162.0 (d, *J* = 5 Hz), 160.6 (d, *J* = 3 Hz), 155.9 (d, *J* = 247 Hz), 145.6, 132.9, 119.6 (d, *J* = 8 Hz), 113.4 (d, *J* = 24 Hz), 106.7 (d, *J* = 25 Hz), 101.3, 55.9, 25.6 ppm; ^19^F NMR (282 MHz, CDCl_3_) δ: −110 ppm; HRMS (ESI-TOF) *m*/*z*: [M + H]^+^ calcd for C_11_H_10_BrFNO: 269.9924; found: 269.9923.

#### 7-Bromo-4-((3,5-dimethylbenzyl)oxy)-6-fluoro-2-methylquinoline (**3c**)

Prepared *via* the general procedure using **1c** (59.1 mg, 0.2 mmol, 1.0 equiv). 99% yield **2c** by ^1^H NMR analysis using 1,3,5-trimethoxybenzene as an internal standard. White solid (0.055 g, 73%); m.p. 138–140 °C; IR (film) ν_max_ 1601, 1561, 1500, 1347, 1193, 1099, 747 cm^–1^; ^1^H NMR (500 MHz, CDCl_3_) δ: 8.20 (d, *J* = 6.5 Hz, 1H), 7.83 (d, *J* = 9.2 Hz, 1H), 7.07 (s, 2H), 7.02 (s, 1H), 6.70 (s, 1H), 5.16 (s, 2H), 2.66 (s, 3H), 2.36 (s, 6H) ppm; ^13^C{^1^H} NMR (125 MHz, CDCl_3_) δ: 160.9 (d, *J* = 5 Hz), 160.6 (d, *J* = 2 Hz), 155.8 (d, *J* = 247 Hz), 146.1, 138.5, 135.2, 133.1, 130.3, 125.5, 119.7 (d, *J* = 8 Hz), 113.2 (d, *J* = 24 Hz), 106.9 (d, *J* = 25 Hz), 102.3, 70.7, 25.8, 21.3 ppm; ^19^F NMR (282 MHz, CDCl_3_) δ: −111 ppm; HRMS (ESI-TOF) *m*/*z*: [M + H]^+^ calcd for C_19_H_18_BrFNO: 374.0550; found: 374.0558.

#### Methyl 7-bromo-4-chloro-6-fluoroquinoline-2-carboxylate (**3d**)

Prepared *via* the general procedure using **1d** (24.0 mg, 0.1 mmol, 1.0 equiv) and purified *via* column chromatography (DCM). 97% yield **2d** by ^1^H NMR analysis using 1,3,5-trimethoxybenzene as an internal standard. White solid (0.025 g, 78%); m.p. 153–155 °C; IR (film) ν_max_ 1725, 1613, 1544, 1345, 1204, 1019, 787, 696 cm^–1^; ^1^H NMR (400 MHz, CDCl_3_) δ: 8.62 (d, *J* = 6.6 Hz, 1H), 8.31 (s, 1H), 7.96 (d, *J* = 8.8 Hz, 1H), 4.10 (s, 3H) ppm; ^13^C{^1^H} NMR (100 MHz, CDCl_3_) δ: 164.6, 158.7 (d, *J* = 255 Hz), 148.1, 145.4 (d, *J* = 1 Hz), 143.2 (d, *J* = 6 Hz), 136.4 (d, *J* = 2 Hz), 127.9 (d, *J* = 9 Hz), 122.1, 115.9 (d, *J* = 25 Hz), 108.8 (d, *J* = 26 Hz), 53.6 ppm; ^19^F NMR (376 MHz, CDCl_3_) δ: −102 ppm; HRMS (ESI-TOF) *m*/*z*: [M + H]^+^ calcd for C_11_H_7_BrClFNO_2_: 317.9327; found: 317.9328.

#### 7-Bromo-6-fluoro-4-methoxyquinoline (**3e**)

Prepared *via* the general procedure using **1f** (35.4 mg, 0.2 mmol, 1.0 equiv). White solid (0.021 g, 41%); m.p. 139–141 °C; IR (film) ν_max_ 1596, 1570, 1499, 1348, 1181, 1093, 717 cm^–1^; ^1^H NMR (400 MHz, CDCl_3_) δ: 8.72 (d, *J* = 5.2 Hz, 1H), 8.30 (d, *J* = 6.6 Hz, 1H), 7.85 (d, *J* = 9.2 Hz, 1H), 6.77 (d, *J* = 5.2 Hz, 1H), 4.06 (s, 3H) ppm; ^13^C{^1^H} NMR (100 MHz, CDCl_3_) δ: 162.0 (d, *J* = 5 Hz), 156.2 (d, *J* = 248 Hz), 151.6 (d, *J* = 3 Hz), 146.3 (d, *J* = 1 Hz), 133.9, 121.2 (d, *J* = 8 Hz), 113.6 (d, *J* = 24 Hz), 106.8 (d, *J* = 25 Hz), 100.7, 56.0 ppm; ^19^F NMR (376 MHz, CDCl_3_) δ: −109 ppm; HRMS (ESI-TOF) *m*/*z*: [M + H]^+^ calcd for C_10_H_8_BrFNO: 255.9768; found: 255.9767.

#### Ethyl 7-bromo-4-chloro-6-fluoroquinoline-3-carboxylate (**3f**)

Prepared *via* the general procedure using **1e** (25.4 mg, 0.1 mmol, 1.0 equiv) and B_2_pin_2_ (55.9 mg, 0.22 mmol, 2.2 equiv) and purified *via* column chromatography (hexane/Et_2_O, 9:1). 95% yield **2c** by ^1^H NMR analysis using 1,3,5-trimethoxybenzene as an internal standard. White solid (0.029 g, 88%); m.p. 119–120 °C; IR (film) ν_max_ 1727, 1609, 1551, 1336, 1153, 1037, 788, 689 cm^–1^; ^1^H NMR (400 MHz, CDCl_3_) δ: 9.17 (s, 1H), 8.42 (d, *J* = 6.6 Hz, 1H), 8.08 (d, *J* = 9.2 Hz, 1H), 4.51 (q, *J* = 7.1 Hz, 2H), 1.47 (q, *J* = 7.1 Hz, 3H) ppm; ^13^C{^1^H} NMR (100 MHz, CDCl_3_) δ: 164.0, 157.9 (d, *J* = 252 Hz), 150.5, 142.6, 142.6 (d, *J* = 6 Hz), 135.1, 126.6 (d, *J* = 8 Hz), 123.7, 116.6 (d, *J* = 25 Hz), 110.1 (d, *J* = 26 Hz), 62.4, 14.2 ppm; ^19^F NMR (376 MHz, CDCl_3_) δ: −104 ppm; HRMS (ESI-TOF) *m*/*zm*/*z*: [M + H]^+^ calcd for C_12_H_9_BrClFNO_2_: 331.9484; found: 331.9481.

### Derivatization of C7-Borylated-6-fluoroquinolines

#### 4-Chloro-6-fluoro-7-iodo-2-methylquinoline (**4a**)

A 15 mL Schlenk flask was oven-dried (150 °C) and cooled under vacuum. The Schlenk flask was refilled with nitrogen, and all reagents were added under a positive pressure of nitrogen in the order: **1a** (39.1 mg, 0.2 mmol, 1.0 equiv), dtbpy (1.6 mg, 0.006 mmol, 3 mol %), B_2_pin_2_ (55.9 mg, 0.22 mmol, 1.1 equiv), and [Ir(OMe)COD]_2_ (2.0 mg, 0.003 mmol, 1.5 mol %). The Schlenk flask was then placed under vacuum for 20 min before being refilled with nitrogen 3 times. THF (0.5 mL) was added *via* syringe through a septum, the reaction was sealed, and the mixture was heated to 80 °C in an aluminum heating block for 14 h, cooled to r.t., and concentrated under reduced pressure. The residue was dissolved in MeOH (1.6 mL) and added to a 5 mL screw-capped vial containing CuI (3.8 mg, 0.02 mmol, 10 mol %), 1,10-phenanthroline (7.2 mg, 0.04 mmol, 0.2 equiv), and KI (49.8 mg, 0.3 mmol, 1.5 equiv). The mixture was stirred at r.t. for 5 min, H_2_O (0.4 mL) was then added, the vial was sealed, and the solution was heated to 80 °C in an aluminum heating block for 2 h.^25^ The solution was cooled to r.t., diluted with DCM and H_2_O, and the layers were separated. The aqueous portion was extracted with DCM (2 ×5 mL), and the combined organic layers were then washed with brine (10 mL), dried over MgSO_4_, filtered, and concentrated under reduced pressure. The product was purified *via* column chromatography (DCM/EtOAc, 95:5). White solid (0.038 g, 64%); m.p. 108–111 °C; IR (film) ν_max_ 1594, 1538, 1097, 704, 628 cm^–1^; ^1^H NMR (300 MHz, CDCl_3_) δ: 8.52 (d, *J* = 6.0 Hz, 1H), 7.76 (d, *J* = 8.4 Hz, 1H), 7.41 (s, 1H), 2.70 (s, 3H) ppm; ^13^C{^1^H} NMR (75 MHz, CDCl_3_) δ: 159.1 (d, *J* = 3 Hz), 159.0 (d, *J* = 247 Hz), 146.1, 141.7, 140.8 (d, *J* = 3 Hz), 125.6 (d, *J* = 9 Hz), 122.9, 107.5 (d, *J* = 27 Hz), 87.2 (d, *J* = 29 Hz), 25.0 ppm; ^19^F NMR (282 MHz, CDCl_3_) δ: −95 ppm; HRMS (ESI-TOF) *m*/*z*: [M + H]^+^ calcd for C_10_H_7_ClFIN: 321.9290; found: 321.9286.

#### 4-Chloro-6-fluoro-2-methylquinoline-7-d (**5a**)

A 15 mL Schlenk flask was flame-dried and cooled under vacuum. The Schlenk flask was refilled with nitrogen, **2a** (64.3 mg, 0.2 mmol, 1.0 equiv) and [Ir(OMe)COD]_2_ (1.3 mg, 0.002 mmol, 1.0 mol %) were added, and the Schlenk flask was evacuated and backfilled with nitrogen 3 times. THF (0.8 mL) and D_2_O (0.2 mL) were added, the vessel was sealed, and the mixture was heated to 80 °C in an oil bath for 12 h. The solution was cooled to r.t. and extracted with Et_2_O (2 ×5 mL).^26^ The combined organic layers were washed with brine (10 mL), dried over MgSO_4_, filtered, and concentrated under reduced pressure. The product was purified *via* column chromatography (DCM/EtOAc, 95:5). Colorless crystalline solid (0.022 g, 57%); m.p. 85–86 °C; IR (film) ν_max_ 1617, 1555, 1025, 830 cm^–1^; ^1^H NMR (400 MHz, CDCl_3_) δ: 8.02 (d, *J* = 5.2 Hz, 1H), 7.79 (d, *J* = 9.4 Hz, 1H), 7.41 (s, 1H), 2.71 (s, 3H) ppm; ^13^C{^1^H} NMR (100 MHz, CDCl_3_) δ: 160.8 (d, *J* = 249 Hz), 158.3 (d, *J* = 3 Hz), 145.6, 142.1 (d, *J* = 5 Hz), 131.5 (d, *J* = 9 Hz), 125.8 (d, *J* = 11 Hz), 122.7, 121.0–120.0 (m), 107.9 (d, *J* = 24 Hz), 25.0 ppm; ^19^F NMR (282 MHz, CDCl_3_) δ: −113 ppm; HRMS (ESI-TOF) *m*/*z*: [M + H]^+^ calcd for C_10_H_7_DClFN: 197.0387; found: 197.0380.

#### (4-Chloro-6-fluoro-2-methylquinolin-7-yl)trifluoroborate potassium salt (**6a**)

A 15 mL Schlenk flask was oven-dried (150 °C) and cooled under vacuum. The Schlenk flask was refilled with nitrogen, and all reagents were added under a positive pressure of nitrogen in the order: **1a** (195.6 mg, 1.0 mmol, 1.0 equiv), dtbpy (8.1 mg, 0.03 mmol, 3 mol %), B_2_pin_2_ (279.3 mg, 1.1 mmol, 1.1 equiv), and [Ir(OMe)COD]_2_ (9.9 mg, 0.015 mmol, 1.5 mol %). The Schlenk flask was then placed under vacuum for 20 min before being refilled with nitrogen 3 times. THF (5 mL) was added *via* a syringe through a septum, the reaction was sealed, and the mixture was heated to 80 °C in an oil bath for 18 h. The solution was cooled, H_2_O (3 mL) and KHF_2_ (468.6 mg, 6.0 mmol, 6.0 equiv) were added, and the mixture was allowed to stir for a further 24 h before being diluted with acetone and H_2_O. Volatiles were removed under reduced pressure, and the resulting precipitate was isolated by suction filtration and washed with H_2_O (15 mL) and cold hexane (15 mL).^27^ Beige solid (0.215 g, 71%); m.p. >250 °C; IR (film) ν_max_ 1640, 1583, 1120, 1033, 799 cm^–1^; ^1^H NMR (600 MHz, (CD_3_)_2_SO) δ: 7.93 (d, *J* = 5.6 Hz, 1H), 7.56 (s, 1H), 7.45 (d, *J* = 9.2 Hz, 1H), 2.61 (s, 3H) ppm; ^13^C{^1^H} NMR (150 MHz, (CD_3_)_2_SO) δ: 164.8 (d, *J* = 245 Hz), 156.7 (d, *J* = 2 Hz), 145.2, 139.8, 134.3 (d, *J* = 15 Hz), 123.5 (d, *J* = 11 Hz), 121.1, 104.8 (d, *J* = 30 Hz), 24.4 ppm; a signal for the carbon directly attached to the boron atom was not observed; ^19^F NMR (282 MHz, (CD_3_)_2_SO) δ: −105, −138 ppm; ^11^B NMR (96 MHz, (CD_3_)_2_SO) δ: 2 ppm; HRMS (ESI-TOF) *m*/*z*: [M + H]^+^ calcd for C_10_H_7_BClF_3_N: 244.0307; found: 244.0311.

#### (6-Fluoro-4-methoxy-2-methylquinolin-7-yl)trifluoroborate potassium salt (**6b**)

Prepared *via* the procedure described for **6a** using **1b** (153 mg, 0.8 mmol, 1.0 equiv). Brown solid (0.174 g, 73%); m.p. >250 °C; IR (film) ν_max_ 1645, 1592, 1146, 1017, 834 cm^–1^; ^1^H NMR (600 MHz, (CD_3_)_2_SO) δ: 8.01 (d, *J* = 4.6 Hz, 1H), 7.59 (d, *J* = 8.4 Hz, 1H), 7.38 (s, 1H), 4.21 (s, 3H), 2.80 (s, 3H) ppm; ^13^C{^1^H} NMR (150 MHz, (CD_3_)_2_SO) δ: 166.9 (d, *J* = 5 Hz), 164.6 (d, *J* = 247 Hz), 156.8, 135.4, 125.1 (d, *J* = 14 Hz), 118.7 (d, *J* = 10 Hz), 104.7 (d, *J* = 30 Hz), 102.4, 58.2, 20.7 ppm; a signal for the carbon directly attached to the boron atom was not observed; ^19^F NMR (282 MHz, (CD_3_)_2_SO) δ: −103, −139 ppm; ^11^B NMR (96 MHz, (CD_3_)_2_SO) δ: 2 ppm; HRMS (ESI-TOF) *m*/*z*: [M + H]^+^ calcd for C_11_H_10_BF_3_NO: 240.0802; found: 240.0807.

#### (6-Fluoro-4-methoxy-2-methylquinolin-7-yl)boronic Acid (**7b**)

**6b** (98 mg, 0.33 mmol, 1.0 equiv) was suspended in THF (11 mL), and a solution of LiOH.H_2_O (124.6 mg, 2.97 mmol, 9.0 equiv) in H_2_O (2.5 mL) was added. The resulting mixture was stirred at r.t. for 24 h. THF was removed under reduced pressure, and the mixture was acidified to pH∼5 using saturated NH_4_Cl (4 mL) and 1 M HCl (2 mL). The resulting precipitate was collected by suction filtration, redissolved in 1 M HCl, and extracted once with Et_2_O. The aqueous portion was then neutralized using solid NaHCO_3_ and extracted with DCM/iPrOH (9:1, 3 × 10 mL). The organic layers were dried over MgSO_4_, filtered, and concentrated under reduced pressure. White solid (0.058 g, 75%); m.p. >250 °C; IR (film) ν_max_ 1646, 1504, 1362, 1250, 1202, 1091 cm^–1^; ^1^H NMR (600 MHz, (CD_3_)_2_SO) δ: 8.52 (s, 2H), 8.04 (d, *J* = 5.7 Hz, 1H), 7.56 (d, *J* = 9.6 Hz, 1H), 6.95 (s, 1H), 4.01 (s, 3H), 2.59 (s, 3H) ppm; ^13^C{^1^H} NMR (150 MHz, (CD_3_)_2_SO) δ: 161.8 (d, *J* = 243 Hz), 160.9 (d, *J* = 5 Hz), 159.2 (d, *J* = 2 Hz), 144.9, 136.1 (d, *J* = 10 Hz), 120.6 (d, *J* = 10 Hz), 104.3 (d, *J* = 27 Hz), 101.9, 56.1, 25.4 ppm; a signal for the carbon directly attached to the boron atom was not observed; ^19^F NMR (282 MHz, (CD_3_)_2_SO) δ: −108 ppm; ^11^B NMR (96 MHz, (CD_3_)_2_SO) δ: 27 ppm; HRMS (ESI-TOF) *m*/*z*: [M + H]^+^ calcd for C_11_H_12_BFNO_3_: 236.0889; found: 236.0894.

#### 7-Benzyl-4-chloro-6-fluoro-2-methylquinoline (**8a**)

A 15 mL Schlenk flask was dried under vacuum, cooled to r.t., and refilled with nitrogen. **2a** (64.3 mg, 0.2 mmol, 1.0 equiv), Pd_2_(dba)_3_ (1.8 mg, 0.002 mmol, 1.0 mol %), PPh_3_ (2.1 mg, 0.008 mmol, 4.0 mol %), and K_2_CO_3_ (110.6 mg, 0.8 mmol, 4.0 equiv) were added, and the Schlenk flask was evacuated and backfilled with nitrogen 3 times. Benzyl bromide (0.03 mL, 0.24 mmol, 1.2 equiv), THF (1 mL), and H_2_O (0.02 mL) were added, the vessel was sealed, and the mixture was heated to 100 °C in an aluminum heating block for 24 h.^24^ The solution was cooled to r.t., diluted with H_2_O, and extracted with EtOAc (3 × 10 mL). The combined organic layers were washed with brine (10 mL), dried over MgSO_4_, filtered, and concentrated under reduced pressure. The product was purified *via* column chromatography (DCM). Off-white solid (0.049 g, 86%); m.p. 94–96 °C; IR (film) ν_max_ 1635, 1598, 1551, 1027, 871 cm^–1^; ^1^H NMR (400 MHz, CDCl_3_) δ: 7.88–7.66 (m, 2H), 7.39–7.14 (m, 6H), 4.16 (s, 2H), 2.66 (s, 3H) ppm; ^13^C{^1^H} NMR (100 MHz, CDCl_3_) δ: 159.7 (d, *J* = 250 Hz), 158.1 (d, *J* = 2 Hz), 145.6, 141.6 (d, *J* = 5 Hz), 138.5, 134.3 (d, *J* = 20 Hz), 131.0 (d, *J* = 6 Hz), 129.1, 128.7, 126.6, 124.4 (d, *J* = 10 Hz), 121.9, 107.7 (d, *J* = 26 Hz), 35.5 (d, *J* = 3 Hz), 24.9 ppm; ^19^F NMR (376 MHz, CDCl_3_) δ: −117 ppm; HRMS (ESI-TOF) *m*/*z*: [M + H]^+^ calcd for C_17_H_14_ClFN: 286.0793; found: 286.0785.

#### Ethyl-4-(4-chloro-6-fluoro-2-methylquinolin-7-yl)benzoate (**9a**)

A 15 mL Schlenk flask was oven-dried (150 °C) and cooled under vacuum. The Schlenk flask was refilled with nitrogen, and all reagents were added under a positive pressure of nitrogen in the order: **1a** (39.1 mg, 0.2 mmol, 1.0 equiv), dtbpy (1.6 mg, 0.006 mmol, 3 mol %), B_2_pin_2_ (55.9 mg, 0.22 mmol, 1.1 equiv), and [Ir(OMe)COD]_2_ (2.0 mg, 0.003 mmol, 1.5 mol %). The Schlenk flask was then placed under vacuum for 20 min before being refilled with nitrogen 3 times. THF (0.5 mL) was added *via* septum, the reaction was sealed, and the mixture was heated to 80 °C in an aluminum heating block for 14 h, cooled to r.t., and concentrated under reduced pressure. A 15 mL Schlenk was heated under vacuum, cooled to r.t., and refilled with nitrogen. The crude reaction mixture for **2a** (0.2 mmol, 1.0 equiv), Pd(PPh_3_)_4_ (11.6 mg, 0.01 mmol, 5.0 mol %), and K_3_PO_4_ (127.4 mg, 0.6 mmol, 3.0 equiv) was added, and the Schlenk flask was evacuated and backfilled with nitrogen 3 times. Ethyl-4-bromobenzoate (0.05 mL, 0.3 mmol, 1.5 equiv), THF (2 mL), and H_2_O (0.4 mL) were added, the vessel was sealed, and the mixture was heated to 60 °C for 18 h.^3l^ The solution was cooled to r.t., diluted with H_2_O, and extracted with Et_2_O (3 × 10 mL). The combined organic layers were washed with brine (10 mL), dried over MgSO_4_, filtered, and concentrated under reduced pressure. The product was purified *via* column chromatography (DCM:Et_2_O, 90:10); X-ray quality crystals were obtained *via* vapor diffusion from a saturated solution of DCM in Et_2_O; and the CCDC number is 2159957. White solid (0.052 g, 76%); m.p. 120–122 °C; IR (film) ν_max_ 1710, 1610, 1598, 1383, 1291, 1027, 857 cm^–1^; ^1^H NMR (300 MHz, CDCl_3_) δ: 8.25–8.09 (m, 3H), 7.89 (d, *J* = 11.2 Hz, 1H), 7.81–7.67 (m, 2H), 7.42 (s, 1H), 4.42 (q, *J* = 7.1 Hz, 2H), 2.73 (s, 3H), 1.43 (t, *J* = 7.1 Hz, 3H) ppm; ^13^C{^1^H} NMR (75 MHz, CDCl_3_) δ: 166.2, 158.9 (d, *J* = 2 Hz), 158.1 (d, *J* = 251 Hz), 145.6, 141.5 (d, *J* = 6 Hz), 139.1 (d, *J* = 2 Hz), 133.1 (d, *J* = 18 Hz), 131.3 (d, *J* = 4 Hz), 130.4, 129.8, 129.2 (d, *J* = 3 Hz), 125.3 (d, *J* = 10 Hz), 122.6, 108.9 (d, *J* = 26 Hz), 61.1, 25.1, 14.4 ppm; ^19^F NMR (282 MHz, CDCl_3_) δ: −116 ppm; HRMS (ESI-TOF) *m*/*z*: [M + H]^+^ calcd for C_19_H_16_ClFNO_2_: 344.0848; found: 344.0839.

#### Ethyl-4-(6-fluoro-4-methoxy-2-methylquinolin-7-yl)benzoate (**9b**)

Prepared *via* the procedure described for **9a** using **1b** (57.4 mg, 0.3 mmol, 1.0 equiv). White solid (0.063 g, 62%); m.p. 93–95 °C; IR (film) ν_max_ 1712, 1608, 1556, 1348, 1280, 1201, 1107, 1024 cm^–1^; ^1^H NMR (300 MHz, CDCl_3_) δ: 8.19–8.11 (m, 2H), 8.06 (d, *J* = 7.3 Hz, 1H), 7.84 (d, *J* = 11.4 Hz, 1H), 7.79–7.69 (m, 2H), 6.66 (s, 1H), 4.42 (q, *J* = 7.1 Hz, 2H), 4.05 (s, 3H), 2.71 (s, 3H), 1.42 (t, *J* = 7.1 Hz, 3H) ppm; ^13^C{^1^H} NMR (75 MHz, CDCl_3_) δ: 166.4, 161.7 (d, *J* = 5 Hz), 160.1 (d, *J* = 2 Hz), 157.1 (d, *J* = 248 Hz), 145.8, 139.9, 132.0 (d, *J* = 17 Hz), 130.4 (d, *J* = 4 Hz), 130.0, 129.7, 129.2 (d, *J* = 3 Hz), 120.2 (d, *J* = 10 Hz), 106.8 (d, *J* = 25 Hz), 101.2, 61.1, 55.7, 25.9, 14.4 ppm; ^19^F NMR (282 MHz, CDCl_3_) δ: −120 ppm; HRMS (ESI-TOF) *m*/*z*: [M + H]^+^ calcd for C_20_H_19_FNO_3_: 340.1343; found: 340.1336.

#### 4-Chloro-6-fluoro-7-(1*H*-indol-5-yl)-2-methylquinoline (**10a**)

A 15 mL Schlenk flask was oven-dried (150 °C), cooled to r.t. under vacuum, and refilled with nitrogen. 5-Bromoindole (23.5 mg, 0.12 mmol, 1.0 equiv), **2a** (48.2 mg, 0.15 mmol, 1.25 equiv), Pd(dba)_2_ (1.4 mg, 0.0024 mmol, 2.0 mol %), P(o-tol)_3_ (2.2 mg, 0.0072 mmol, 6.0 mol %), and Na_2_CO_3_ (50.9 mg, 0.48 mmol, 4.0 equiv) were added, and the Schlenk flask was evacuated and backfilled with nitrogen 3 times. THF (0.45 mL) and H_2_O (0.05 mL) were added, the vessel was sealed, and the mixture was heated to 50 °C in an oil bath for 24 h.^28^ The mixture was cooled to r.t., filtered through a short plug of Celite with DCM, and concentrated under reduced pressure. The product was purified *via* column chromatography (DCM/EtOAc, 98:2). Bright yellow solid (0.032 g, 86%); m.p. 160–162 °C; IR (film) ν_max_ 3415, 1626, 1596, 1545, 1011, 867 cm^–1^; ^1^H NMR (400 MHz, (CD_3_)_2_SO) δ: 11.30 (bs, 1H), 8.06 (d, *J* = 7.5 Hz, 1H), 7.97–7.77 (m, 2H), 7.66 (s, 1H), 7.55 (d, *J* = 8.4 Hz), 7.51–7.32 (m, 2H), 6.54 (bs, 1H), 2.64 (s, 3H) ppm; ^13^C{^1^H} NMR (100 MHz, (CD_3_)_2_SO) δ: 158.9 (d, *J* = 2 Hz), 158.0 (d, *J* = 249 Hz), 145.2, 140.1 (d, *J* = 5 Hz), 135.9, 135.1 (d, *J* = 18 Hz), 130.4 (d, *J* = 3 Hz), 128.0, 126.4, 124.7 (d, *J* = 1 Hz), 123.4 (d, *J* = 11 Hz), 122.27, 122.24, 121.1 (d, *J* = 3 Hz), 111.8, 108.0 (d, *J* = 26 Hz), 101.8, 24.5 ppm; ^19^F NMR (282 MHz, (CD_3_)_2_SO) δ: −116 ppm; HRMS (ESI-TOF) *m*/*z*: [M + H]^+^ calcd for C_18_H_13_ClFN_2_: 311.0746; found: 311.0741.

#### 6-Fluoro-4-methoxy-2-methyl-7-(piperidin-1-yl)quinoline (**11b**)

A 15 mL Schlenk flask was dried under vacuum, cooled to r.t., and refilled with nitrogen. **3b** (27.0 mg, 0.1 mmol, 1.0 equiv), RuPhos (2.8 mg, 0.006 mmol, 6.0 mol %), and NaO*t*-Bu (24 mg, 0.25 mmol, 2.5 equiv) were added, and the Schlenk flask was evacuated and backfilled with nitrogen 3 times. Piperidine (0.02 mL, 0.2 mmol, 2.0 equiv) and toluene (1 mL) were added, and the mixture was degassed with nitrogen for 5 min. Pd_2_(dba)_3_ (2.7 mg, 0.003 mmol, 2.0 mol %) was then added, the vessel was sealed, and the suspension was heated to 80 °C in an oil bath for 16 h. The product was purified *via* column chromatography (DCM/EtOAc, 98:2 with 1% NEt_3_ as modifier). Yellow oil (0.024 g, 88%); IR (film) ν_max_ 1605, 1514, 1381, 1350, 1241, 1131, 1019 cm^–1^; ^1^H NMR (500 MHz, CDCl_3_) δ: 7.64 (d, *J* = 13.5 Hz, 1H), 7.41 (d, *J* = 8.3 Hz, 1H), 6.51 (s, 1H), 3.99 (s, 3H), 3.23–3.09 (m, 4H), 2.67 (s, 3H), 1.86–1.70 (m, 4H), 1.68–1.56 (m, 2H) ppm; ^13^C{^1^H} NMR (125 MHz, CDCl_3_) δ: 161.9 (d, *J* = 5 Hz), 159.2 (d, *J* = 2 Hz), 154.5 (d, *J* = 249 Hz), 146.6, 145.9 (d, *J* = 12 Hz), 115.5 (d, *J* = 3 Hz), 114.3 (d, *J* = 10 Hz), 106.4 (d, *J* = 24 Hz), 99.3, 55.6, 51.9 (d, *J* = 4 Hz), 26.0, 25.5, 24.2 ppm; ^19^F NMR (282 MHz, CDCl_3_) δ: −120 ppm; HRMS (ESI-TOF) *m*/*z*: [M + H]^+^ calcd for C_16_H_20_FN_2_O: 275.1554; found: 275.1555.

#### 4-Chloro-6-fluoro-2-methylquinolin-7-ol (**12a**)

A solution of **2a** (64.3 mg, 0.2 mmol, 1.0 equiv) in MeOH (0.5 mL) in a 5 mL screw-capped vial was cooled to 0 °C over ice. 30% H_2_O_2_ solution (0.02 mL, 0.24 mmol, 1.18 equiv) was gradually added, and the mixture was allowed warm slowly to r.t. and stirred at this temperature for 16 h. The reaction mixture was diluted with MeOH, quenched using saturated Na_2_S_2_O_3_ solution (∼1 mL), and MeOH was removed under reduced pressure. The mixture was then extracted with EtOAc (3 × 10 mL), dried over MgSO_4_, filtered, and concentrated under reduced pressure. White solid (0.036 g, 85%); m.p. 192–195 °C; IR (film) ν_max_ 1629, 1539, 1261, 1032, 797 cm^–1^; ^1^H NMR (600 MHz, (CD_3_)_2_SO) δ: 11.09 (bs, 1H), 7.78 (d, *J* = 11.7 Hz, 1H), 7.49 (s, 1H), 7.40 (d, *J* = 8.5 Hz, 1H), 2.58 (s, 3H) ppm; ^13^C{^1^H} NMR (150 MHz, (CD_3_)_2_SO) δ: 158.7 (d, *J* = 2 Hz), 152.1 (d, *J* = 249 Hz), 149.2 (d, *J* = 15 Hz), 146.5, 140.0 (d, *J* = 5 Hz), 119.8, 117.8 (d, *J* = 9 Hz), 113.2 (d, *J* = 3 Hz), 108.1 (d, *J* = 21 Hz), 24.5 ppm; ^19^F NMR (282 MHz, (CD_3_)_2_SO) δ: −131 ppm; HRMS (ESI-TOF) *m*/*z*: [M + H]^+^ calcd for C_10_H_8_ClFNO: 212.0273; found: 212.0269.

### Methods to Access 4-Fluoroquinolones

#### (6-Fluoro-2-methyl-4-oxo-1,4-dihydroquinolin-7-yl)boronic Acid (**13a**)

A 15 mL Schlenk flask was oven-dried (150 °C) and cooled under vacuum. The Schlenk flask was refilled with nitrogen, and all reagents were added under a positive pressure of nitrogen in the order: **1a** (39.1 mg, 0.2 mmol, 1.0 equiv), dtbpy (1.6 mg, 0.006 mmol, 3 mol %), B_2_pin_2_ (55.9 mg, 0.22 mmol, 1.1 equiv), and [Ir(OMe)COD]_2_ (2.0 mg, 0.003 mmol, 1.5 mol %). The Schlenk flask was then placed under vacuum for 20 min before being refilled with nitrogen 3 times. THF (0.5 mL) was added *via* a syringe through a septum, the reaction was sealed, and the mixture was heated to 80 °C in an aluminum heating block for 14 h, cooled to r.t., and concentrated under reduced pressure. The crude reaction mixture for **2a** (0.2 mmol, 1.0 equiv) and NaOAc (32.8 mg, 0.4 mmol, 2.0 equiv) was added to a 5 mL screw-capped vial followed by glacial acetic acid (1.25 mL). The vial was sealed and stirred at 120 °C in an aluminum heating block for 1 h, cooled to r.t., concentrated under reduced pressure, diluted with H_2_O, and allowed to stand for 1 h. The resulting precipitate was isolated by suction filtration and washed with H_2_O and cold Et_2_O. Pink solid (0.030 g, 69%); m.p. 245–249 °C; IR (film) ν_max_ 1655, 1608, 1505, 1375, 1275, 1110 cm^–1^; ^1^H NMR (300 MHz, (CD_3_)_2_SO) δ: 11.68 (bs, 1H), 8.48 (s, 2H), 7.67 (d, *J* = 4.8 Hz, 1H), 7.56 (d, *J* = 9.3 Hz, 1H), 5.90 (s, 1H), 2.34 (s, 3H) ppm; ^13^C{^1^H} NMR (75 MHz, (CD_3_)_2_SO) δ: 176.0 (d, *J* = 3 Hz), 160.9 (d, *J* = 241 Hz), 149.9, 136.3, 126.4 (d, *J* = 7 Hz), 125.0 (d, *J* = 10 Hz), 108.0 (d, *J* = 26 Hz), 107.6, 19.5 ppm; a signal for the carbon directly attached to the boron atom was not observed; ^19^F NMR (282 MHz, (CD_3_)_2_SO) δ: −111 ppm; ^11^B NMR (96 MHz, (CD_3_)_2_SO) δ: 27 ppm; HRMS (ESI-TOF) *m*/*z*: [M + H]^+^ calcd for C_10_H_10_BFNO_3_: 222.0732; found: 222.0738.

#### 7-Bromo-6-fluoro-2-methylquinolin-4(1*H*)-one (**13b**)

**3b** (27.0 mg, 0.1 mmol, 1.0 equiv) was added to a 5 mL screw-capped vial followed by 48% HBr solution (0.25 mL) and glacial acetic acid (0.5 mL). The vial was sealed and stirred at 150 °C in an aluminum heating block for 6 h, cooled to r.t., diluted with H_2_O, and neutralized using 6 M NaOH. The resulting precipitate was isolated by suction filtration and washed with H_2_O and cold Et_2_O. Off-white solid (0.021 g, 82%); m.p. >250 °C; IR (film) ν_max_ 1639, 1601, 1558, 1272, 1021, 751 cm^–1^; ^1^H NMR (600 MHz, (CD_3_)_2_SO) δ: 11.73 (bs, 1H), 8.00–7.62 (m, 2H), 5.95 (s, 1H), 2.36 (s, 3H) ppm; ^13^C{^1^H} NMR (150 MHz, (CD_3_)_2_SO) δ: 175.4, 154.2 (d, *J* = 242 Hz), 150.5, 137.3, 125.0 (d, *J* = 5 Hz), 122.8, 113.0 (d, *J* = 24 Hz), 110.4 (d, *J* = 23 Hz), 108.1, 19.5 ppm; ^19^F NMR (282 MHz, (CD_3_)_2_SO) δ: −115 ppm; HRMS (ESI-TOF) *m/z*: [M + H]^+^ calcd for C_10_H_8_BrFNO: 255.9768; found: 255.9772.

#### 6-Fluoro-2-methyl-7-(4,4,5,5-tetramethyl-1,3,2-dioxaborolan-2-yl)quinolin-4(1*H*)-one (**13c**)

A 15 mL Schlenk flask was oven-dried (150 °C) and cooled under vacuum. The Schlenk flask was refilled with nitrogen, and all reagents were added under a positive pressure of nitrogen in the order: **1c** (59.1 mg, 0.2 mmol, 1.0 equiv), dtbpy (1.6 mg, 0.006 mmol, 3 mol %), B_2_pin_2_ (55.9 mg, 0.22 mmol, 1.1 equiv), and [Ir(OMe)COD]_2_ (2.0 mg, 0.003 mmol, 1.5 mol %). The Schlenk flask was then placed under vacuum for 20 min before being refilled with nitrogen 3 times. THF (0.5 mL) was added *via* a syringe through a septum, the reaction was sealed, and the mixture was heated to 80 °C in an aluminum heating block for 16 h. The reaction was cooled to r.t., diluted with MeOH, and concentrated under reduced pressure. The residue was dissolved in MeOH (2 mL) and added to a small Schlenk flask, followed by 10% Pd/C (2.0 mg, 0.02 mmol, 10 mol %). The resulting suspension was purged with H_2_ and then stirred under a balloon of H_2_ at 50 °C for 16 h. The mixture was cooled to r.t., diluted with DCM, filtered through a short plug of Celite, and concentrated under reduced pressure. The product was purified *via* trituration from DCM/Et_2_O (1:1). Beige solid (0.032 g, 53%); m.p. >250 °C; IR (film) ν_max_ 1643, 1561, 1373, 1340, 1265, 1142, 1035 cm^–1^; ^1^H NMR (600 MHz, (CD_3_)_2_SO) δ: 11.71 (bs, 1H), 7.92 (d, *J* = 4.8 Hz, 1H), 7.60 (d, *J* = 9.5 Hz, 1H), 5.93 (s, 1H), 2.34 (s, 3H), 1.34 (s, 12H) ppm; ^13^C{^1^H} NMR (150 MHz, (CD_3_)_2_SO) δ: 175.7, 161.4 (d, *J* = 246 Hz), 150.2, 136.2, 127.9 (d, *J* = 7 Hz), 127.4 (d, *J* = 8 Hz), 108.7 (d, *J* = 25 Hz), 107.9, 84.2, 24.7, 19.5 ppm; a signal for the carbon directly attached to the boron atom was not observed; ^19^F NMR (282 MHz, (CD_3_)_2_SO) δ: −110 ppm; ^11^B NMR (96 MHz, (CD_3_)_2_SO) δ: 30 ppm; HRMS (ESI-TOF) *m*/*z*: [M + H]^+^ calcd for C_16_H_20_BFNO_3_: 304.1515; found: 304.1515. *Note that compound **13c** contained a trace impurity **13a**; over time, complete conversion from **13c** to **13a** was observed in solution.

#### Ethyl 6-fluoro-4-oxo-7-(4,4,5,5-tetramethyl-1,3,2-dioxaborolan-2-yl)-1,4-dihydroquinoline-3-carboxylate (**13d**)

Prepared *via* the procedure described for **13a** using **1f** (50.7 mg, 0.2 mmol, 1.0 equiv), with the acid-catalyzed hydrolysis step being carried out for 2 h at 120 °C. The product was purified *via* trituration from a mixture of hot acetone/MeOH. White solid (0.039 g, 53%); m.p. >250 °C; IR (film) ν_max_ 1694, 1613, 1534, 1401, 1334, 1265, 1143, 1043 cm^–1^; ^1^H NMR (600 MHz, (CD_3_)_2_SO) δ: 8.60 (s, 1H), 8.01 (d, *J*= 4.8 Hz, 1H), 7.71 (d, *J* = 9.4 Hz, 1H), 4.21 (q, *J* = 7.1 Hz, 2H), 1.39–1.18 (m, 15H) ppm; ^13^C{^1^H} NMR (150 MHz, (CD_3_)_2_SO) δ: 172.5, 164.8, 162.5 (d, *J* = 248 Hz), 145.5, 135.6, 131.0 (d, *J* = 6 Hz), 128.6, 109.8 (d, *J* = 25 Hz), 109.1, 84.2, 59.6, 26.7, 14.3 ppm; a signal for the carbon directly attached to the boron atom was not observed; ^19^F NMR (282 MHz, (CD_3_)_2_SO) δ: −108 ppm; ^11^B NMR (96 MHz, (CD_3_)_2_SO) δ: 30 ppm; HRMS (ESI-TOF) *m*/*z*: [M + H]^+^ calcd for C_18_H_22_BFNO_5_: 362.1570; found: 362.1577. *Note that compound **13d** was highly insoluble in (CD_3_)_2_SO and degraded in solution over a matter of hours; once synthesized, the compound (∼2 mg) was dissolved in (CD_3_)_2_SO with gentle heating and immediately taken for NMR analysis.

#### Ethyl 7-bromo-6-fluoro-4-oxo-1,4-dihydroquinoline-3-carboxylate (**13e**)

**3f** (30 mg, 0.09 mmol, 1.0 equiv) was dissolved in EtOH (2.5 mL), and 1 M HCl (0.5 mL) was added. The mixture was stirred at 100 °C in an oil bath for 3 h. Upon cooling, a white precipitate formed, which was isolated *via* suction filtration and rinsed with H_2_O, cold EtOH, and Et_2_O to give **13e** as a white solid (0.018 g, 64%); m.p. >250 °C; IR (film) ν_max_ 1694, 1549, 1507, 1358, 1250, 1192, 1103 cm^–1^; ^1^H NMR (water suppression, 600 MHz, 313 K, (CD_3_)_2_SO) δ: 8.59 (s, 1H), 7.98 (d, *J* = 5.8 Hz, 1H), 7.91 (d, *J* = 9.1 Hz, 1H), 4.22 (q, *J* = 7.1 Hz, 2H), 1.28 (q, *J* = 7.1 Hz, 3H) ppm; ^13^C{^1^H} NMR (150 MHz, 313 K, (CD_3_)_2_SO) δ: 172.2, 164.8, 155.2 (d, *J* = 244 Hz), 146.3 (d, *J* = 2 Hz), 137.3 (d, *J* = 3 Hz), 128.2 (d, *J* = 6 Hz), 124.8, 113.7 (d, *J* = 25 Hz), 111.3 (d, *J* = 23 Hz), 109.4, 59.7, 14.3 ppm; ^19^F NMR (282 MHz, (CD_3_)_2_SO) δ: −113 ppm; HRMS (ESI-TOF) *m*/*z*: [M + H]^+^ calcd for C_12_H_10_BrFNO_3_: 313.9823; found: 313.9826.

## Supporting Information Available

Supplementary experiments, experimental details, and copies of NMR spectra (PDF)
