# Supplementary material for: Iridium-Catalyzed Borylation of 6-Fluoroquinolines: Access to 6-Fluoroquinolones
Source: J Org Chem. 2022 Jul 15;87(15):9977–87. doi: 10.1021/acs.joc.2c00973 (PMC9368603; doi:10.1021/acs.joc.2c00973)
Supplement: Supplementary file 1 — jo2c00973_si_001.pdf [file jo2c00973_si_001.pdf]

Supporting Information for:

Iridium-catalyzed Borylation of 6-Fluoroquinolines; Access to 6-Fluoroquinolones

Aobha Hickey,[a] Julia Merz,[b] Hamad H. Al Mamari,[b,c] Alexandra Friedrich,[b] Todd B. Marder,[b] Gerard P. McGlacken[a,d]*

[a]School of Chemistry & Analytical and Biological Chemistry Research Facility, University College Cork, T12 YN60, Ireland

[b]Institute for Inorganic Chemistry, and Institute for Sustainable Chemistry & Catalysis with Boron, Julius-Maximilians-Universität Würzburg, Am Hubland, 97074 Würzburg, Germany

[c]Department of Chemistry, College of Science, Sultan Qaboos University, PO Box 36, Al Khoudh 123, Muscat, Sultanate of Oman

[d]Synthesis and Solid State Pharmaceutical Centre, University College Cork, T12 YN60, Ireland

*g.mcglacken@ucc.ie

Contents

Experimental data S2

Supplementary experiments S2

Optimization of the C–H borylation of 4-chloro-6-fluoro-2-methylquinoline S4

Synthesis of quinoline starting materials and precursors S5

Borylation of quinolones S7

Borylation and subsequent bromination of 6-fluoroquinolines S9

Single-crystal X-ray diffraction S11

^{1}H NMR and $^{13}C\{^{1}H\}$ NMR Spectra S14

References S124

Experimental data

Supplementary experiments

As the N-H bond of quinolones is labile and would likely be the preferred site for borylation,[1] our initial studies involved protecting this site using a methyl group. Preliminary investigations aimed to exploit the inherent steric and electronic biases of the protected quinolone motif **S1** to target the C7 position for borylation. Indeed, by using dtbpy as a ligand in THF at rt for 10 d, the C7 borylated quinolone **S2A** was preferentially, but not exclusively synthesized. Using this system, we observed incomplete consumption of the quinolone starting material along with the formation of the minor C6 isomer **S2B** in a ratio of 1:0.28. As the quinolone starting material is sparingly soluble in THF (and insoluble in all other common borylation solvents), we then increased the temperature to encourage reaction progress. After 24 h, **S1** was consumed but the ratio of the unwanted C6 isomer **S2B** had almost doubled to give a ratio of 1:0.4 (**Scheme S1**). The borylated products were not stable on silica and all other attempts to separate the two isomers were ineffective.

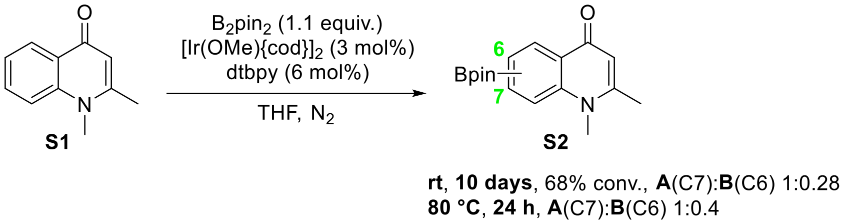

Scheme S1 Borylation of 1,2-dimethylquinolone.

We wanted to explore the applicability of a similar system to target the C7 position of a 6-fluoroquinolone **S3**. Although borylation did occur at the desired site **S4A**, reduction of the C2-C3 bond **S4B** was also observed in a ratio of 1:0.36, as determined by ^{1}H NMR analysis (**Scheme S2**).

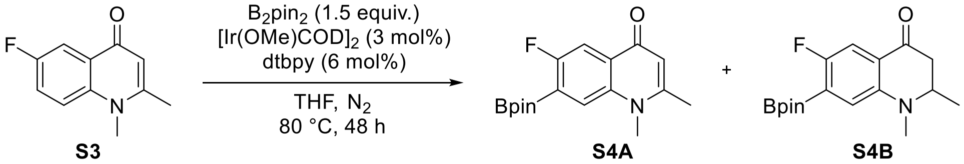

Scheme S2 Borylation of 6-fluoro-2-methylquinolone.

Exclusion of the methyl group at the C2 position **S5** gave a mixture of starting material, the C7-borylated **S6A** and C3/C7-bisborylated **S6B** products, which were identified and characterized subsequent to bromination (**Scheme S3**).

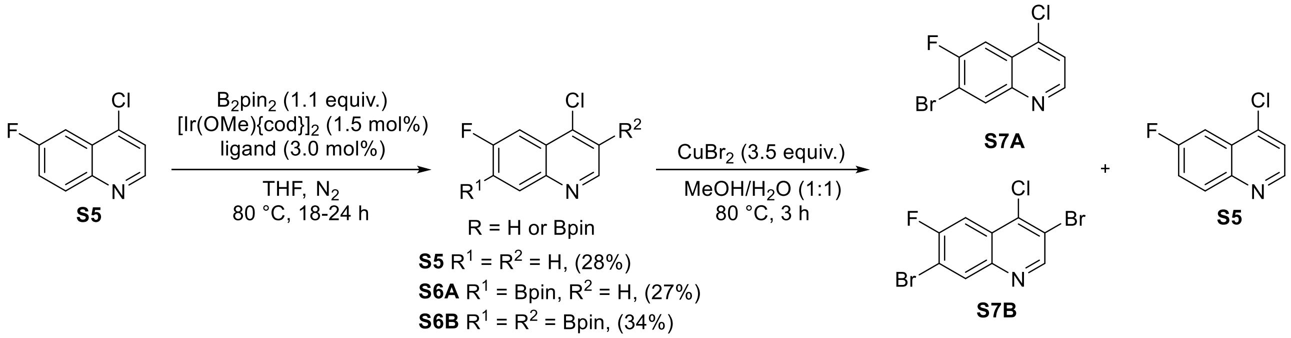

Scheme S3 Borylation and subsequent bromination of 4-chloro-6-fluoroquinoline. Yields in parentheses were calculated from ^{1}H NMR analysis of the crude reaction mixture using 1,3,5-trimethoxybenzene as an internal standard.

Substitution of the chlorine atom with a methoxy group to discourage borylation at C3 through steric hinderance gave greater conversion to the C7-borylated compound. However, another by-product was observed by ^{1}H NMR analysis of the crude reaction mixture. The other species was identified subsequent to bromination as the C2/C7-bisborylated product (**Scheme S4**).

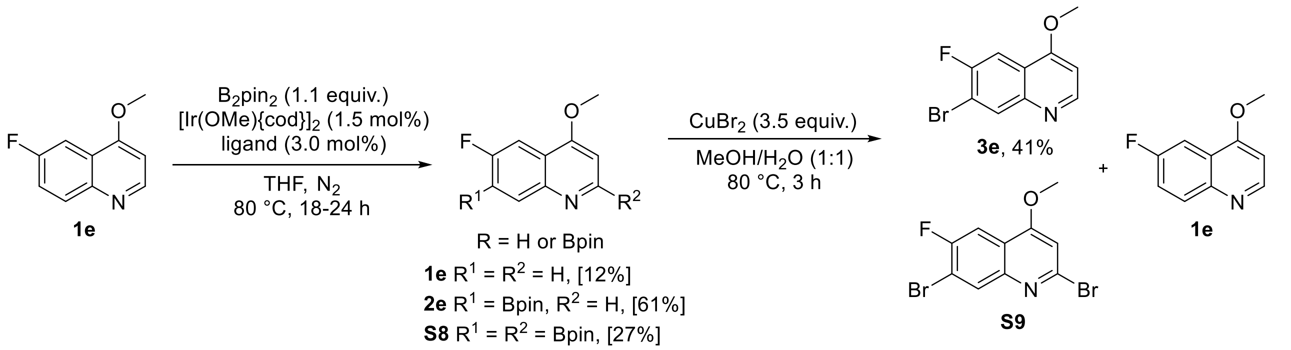

Scheme S4 Borylation and subsequent bromination of 6-fluoro-4-methoxyquinoline. Isolated yield for **3f**. Percentages in square brackets represent approximate conversions as calculated from ^{1}H NMR analysis of the crude reaction mixture.

Optimization of the C–H borylation of 4-chloro-6-fluoro-2-methylquinoline

Table S1 Optimization of the borylation of **1a**.

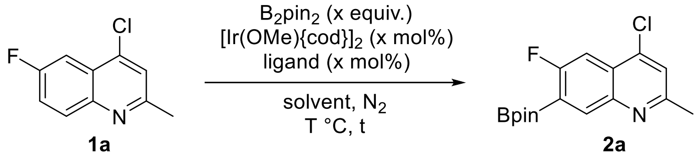

entry	B_2pin_2 (equiv.)	Ir cat. (mol%)	ligand (mol%)	solvent (mL)	temp. (°C)	time (h)	yield[a]
1	1.5	3.0	dtbpy 6.0	THF 3 mL	80	12–48	45-88%
2	0.5	3.0	dtbpy 6.0	THF 3 mL	80	24	61%
3	0.75	3.0	dtbpy 6.0	THF 3 mL	80	24	89%
4	1.05	3.0	dtbpy 6.0	THF 3 mL	80	24	89%
5	1.1	3.0	dtbpy 6.0	THF 3 mL	80	24	>99%
6	1.5	3.0	phen 6.0	THF 3 mL	80	24	91%
7	0.5	3.0	phen 6.0	THF 3 mL	80	24	43%
8	1.1	3.0	phen 6.0	THF 3 mL	80	24	72%
9	1.1	3.0	dtbpy 6.0	CPME 3 mL	100	24	–
10	1.1	3.0	dtbpy 6.0	MTBE 3 mL	60	24	70%
11	1.1	1.5	dtbpy 3.0	MTBE 1 mL	60	18	87%
12	1.1	1.5	dtbpy 3.0	MTBE 1 mL	60	24	95%
13	1.1	2.0	dtbpy 4.0	THF 3 mL	80	24	90%
14	1.1	1.5	dtbpy 3.0	THF 3 mL	80	24	93%
15	1.1	3.0	dtbpy 6.0	THF 1 mL	80	18	95%
16	1.1	1.5	dtbpy 3.0	THF 1 mL	80	12	90%
**17**	**1.1**	**1.5**	**dtbpy 3.0**	**THF 1 mL**	**80**	**18**	**98%**
18	1.1	1.5	none	THF 1 mL	80	24	–
19[b]	1.1	1.5	dtbpy 3.0	THF (dry) 1 mL	80	18	–
20[c]	1.1	1.5	dtbpy 3.0	THF 1 mL	80	18	86%

Reactions carried out on 0.2 mmol scale. [a]Yields calculated from ^{1}H NMR analysis of the crude reaction mixture using 1,3,5-trimethoxybenzene as an internal standard. [b]Solvent dried over flame-dried 4 Å molecular sieves (not distilled). [c]Reaction carried out in air.

Synthesis of quinoline starting materials and precursors

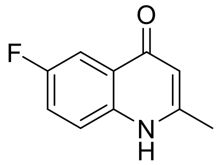

6-Fluoro-2-methylquinolin-4(1*H*)-one[2]

An equimolar solution of 4-fluoroaniline (3.45 mL, 36 mmol, 1.0 equiv.) and ethyl acetoacetate (4.55 mL, 36 mmol, 1.0 equiv.) was added to polyphosphoric acid (20 g, 5% w/w) and stirred at 150 °C in a DrySyn heating block for 3 h. The reaction mixture was poured directly into ice water and neutralized using 3 M NaOH. The resulting yellow precipitate was isolated and redissolved in 5% NaOH solution, treated with charcoal, filtered, and the filtrate neutralized using 6 M HCl. The precipitate was isolated by suction filtration, washed with water, and dried at 150 °C overnight.

White solid (2.537 g, 39%); m.p. >250 °C (lit.[2] 268–269 °C); ^{1}H NMR (300 MHz, $(CD_3)_2SO$) δ: 11.91 (bs, 1H), 7.78–7.42 (m, 3H), 5.96 (s, 1H), 2.36 (s, 3H) ppm; ^{13}C{^{1}H} NMR (75 MHz, $(CD_3)_2SO$) δ: 175.5, 158.1 (d, *J* = 241 Hz), 150.2, 136.8, 125.4 (d, *J* = 7 Hz), 120.5 (d, *J* = 8 Hz), 120.3 (d, *J* = 25 Hz), 108.7 (d, *J* = 22 Hz), 107.6, 19.4 ppm; ^{19}F NMR (282 MHz, $(CD_3)_2SO$) δ: -115 ppm; *m/z* (ES+): 178 ((M+H)$^+$ 100%).

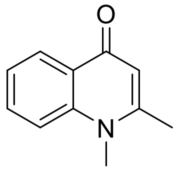

1,2-Dimethylquinolin-4(1*H*)-one (S1)[3]

2-Methylquinolin-4(1*H*)-one (5.0 g, 31.4 mmol, 1.0 equiv.) and KOH (5.29 g, 94.2 mmol, 3.0 equiv.) were suspended in MeOH (32 mL) and MeI (19.6 mL, 314 mmol, 10.0 equiv.) was added dropwise. The resulting mixture was stirred at r.t. for 24 h, then filtered through a pad of celite using MeOH and concentrated under reduced pressure. The residue was taken up in DCM, again filtered through a pad of celite concentrated under reduced pressure and was then purified *via* column chromatography ($CHCl_3$:MeOH).

Beige solid (3.035 g, 56%); m.p. 169–170 °C (lit.[3] 174–175 °C); ^{1}H NMR (300 MHz, $CDCl_3$) δ: 8.43 (dd, *J* = 8.0, 1.4 Hz, 1H), 7.64 (ddd, *J* = 8.7, 7.0, 1.7 Hz, 1H), 7.47 (d, *J* = 8.6 Hz, 1H), 7.36 (ddd, *J* = 8.0, 7.0, 0.9 Hz), 6.20 (s, 1H), 3.71 (s, 3H), 2.45 (s, 3H) ppm; ^{13}C{^{1}H} NMR (75 MHz, $CDCl_3$) δ: 177.7, 150.9, 141.7, 132.0, 126.7, 126.6, 123.2, 115.1, 111.8, 34.2, 22.2 ppm; *m/z* (ES+): 174 ((M+H)$^+$ 100%).

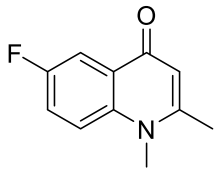

6-Fluoro-1,2-dimethylquinolin-4(1*H*)-one (S3)

Prepared *via* the method described for compound **S1** using 6-fluoro-2-methylquinolin-4(1*H*)-one (1.17 g, 6.1 mmol, 1.0 equiv.).

White solid (0.466 g, 40%); m.p. 212–215 °C; IR (film) ν_{max} 1638, 1568, 1503, 1260, 1161 cm^{-1}; ^1H NMR (300 MHz, CD_3OD) δ: 7.99–7.79 (m, 2H), 7.55 (ddd, *J* = 9.2, 7.8, 3.1 Hz, 1H), 6.27 (s, 1H), 3.87 (s, 3H), 2.58 (s, 3H) ppm; $^{13}C\{^1H\}$ NMR (75 MHz, CD_3OD) δ: 177.0 (d, *J* = 3 Hz), 159.2 (d, *J* = 245 Hz), 154.2, 138.5 (d, *J* = 2 Hz), 127.1 (d, *J* = 9 Hz), 120.7 (d, *J* = 25 Hz), 119.2 (d, *J* = 8 Hz), 109.9, 109.3 (d, *J* = 23 Hz), 34.4, 20.7 ppm; ^{19}F NMR (282 MHz, $CDCl_3$) δ: -120 ppm; HRMS (ESI-TOF) *m/z*: $[M+H]^+$ calcd for $C_{11}H_{11}FNO$: 192.0819; found: 192.0811.

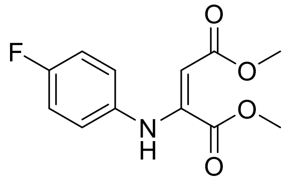

Dimethyl 2-((4-fluorophenyl)amino)maleate

An equimolar solution of 4-fluoroaniline (0.47 mL, 5.0 mmol, 1.0 equiv.) and dimethyl acetylene dicarboxylate (0.61 mL, 5.0 mmol, 1.0 equiv.) in MeOH (18 mL) was heated to 80 °C in an oil bath for 20 h. The mixture was cooled to r.t. and concentrated under reduced pressure. The residue was dissolved in DCM and washed with 1 M HCl (10 mL) and brine (10 mL), dried over $MgSO_4$, filtered and concentrated under reduced pressure.

Yellow oil (1.169 g, 92%); IR (film) ν_{max} 3308, 1726, 1603, 1509, 1276, 1142, 1028 cm^{-1}; ^1H NMR (300 MHz, $CDCl_3$) δ: 9.58 (bs, 1H), 7.03–6.94 (m, 2H), 6.93–6.84 (m, 2H), 5.40 (s, 1H), 3.74 (s, 3H), 3.68 (s, 3H) ppm; $^{13}C\{^1H\}$ NMR (75 MHz, $CDCl_3$) δ: 169.9, 164.5, 159.8 (d, *J* = 244 Hz), 148.2, 136.5 (d, *J* = 3 Hz), 122.9 (d, *J* = 8 Hz), 115.9 (d, *J* = 23 Hz), 93.4, 52.7, 51.1 ppm; ^{19}F NMR (282 MHz, $CDCl_3$) δ: -118 ppm; HRMS (ESI-TOF) *m/z*: $[M+H]^+$ calcd for $C_{12}H_{13}FNO_4$: 254.0823; found: 254.0819.

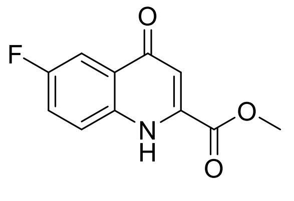

Methyl 6-fluoro-4-oxo-1,4-dihydroquinoline-2-carboxylate[4]

A solution of dimethyl 2-((4-fluorophenyl)amino)maleate (702 mg, 2.77 mmol, 1.0 equiv.) in Ph_2O (5 mL) was added dropwise to refluxing (>260 °C) Ph_2O (15 mL) and left to cyclize at this temperature in a DrySyn heating block for 1 h. After cooling to r.t., Et_2O (50 mL) was added. The resulting precipitate was collected by suction filtration and washed with Et_2O and hexane.

Beige solid (0.403 g, 66%); m.p. 250–251 °C (lit.[4] 250–251 °C); ^1H NMR (300 MHz, $(CD_3)_2SO$) δ: 12.22 (bs, 1H), 8.02 (dd, *J* = 9.0, 4.6 Hz, 1H), 7.85–7.48 (m, 2H), 6.61 (s, 1H), 3.97 (s, 3H) ppm; $^{13}C\{^1H\}$ NMR (75 MHz, $(CD_3)_2SO$) δ: 176.8 (d, *J* = 3 Hz), 162.5, 158.8 (d, *J* = 243 Hz), 137.8, 136.7, 127.1 (d, *J* = 5 Hz), 122.5 (d, *J* = 7 Hz), 121.6 (d, *J* = 25 Hz), 109.2, 108.6 (d, *J* = 22 Hz), 53.5 ppm; ^{19}F NMR (282 MHz, $(CD_3)_2SO$) δ: -117 ppm; *m/z* (ES+): 222 ($(M+H)^+$ 100%).

Diethyl 2-[(4-fluorophenylamino)methylene]malonate[5]

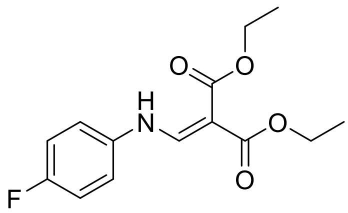

An equimolar solution of 4-fluoroaniline (1.71 mL, 18 mmol, 1.0 equiv.) and diethyl ethoxymethylenemalonate (3.64 mL, 18 mmol, 1.0 equiv.) was heated to 90 °C in an oil bath for 2.5 h. The mixture was cooled to r.t., concentrated under reduced pressure. The residue was dissolved in DCM and washed with 1 M HCl (10 mL) and brine (10 mL), dried over $MgSO_4$, filtered and concentrated under reduced pressure.

White crystalline solid (2.107 g, 75%); m.p. 64–65 °C (lit.[5] 69–70 °C); ^{1}H NMR (300 MHz, $CDCl_3$) δ: 11.00 (bd, *J* = 13.3 Hz, 1H), 8.43 (d, *J* = 13.6 Hz, 1H), 7.21–6.96 (m, 4H), 4.50–4.08 (m, 4H), 1.52–1.18 (m, 6H) ppm; ^{13}C{^{1}H} NMR (75 MHz, $CDCl_3$) δ: 169.1, 165.7, 160.0 (d, *J* = 245 Hz), 152.1, 135.6, 118.9 (d, *J* = 8 Hz), 116.6 (d, *J* = 23 Hz), 93.6, 60.4, 60.1, 14.4, 14.3 ppm; ^{19}F NMR (282 MHz, $CDCl_3$) δ: -118 ppm; *m/z* (ES-): 280 ($(M\text{-}H)^-$ 56%).

Ethyl 6-fluoro-4-oxo-1,4-dihydroquinoline-3-carboxylate[5]

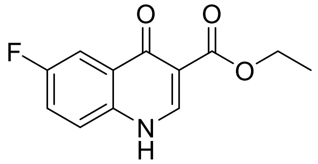

Prepared *via* the method described for compound methyl 6-fluoro-4-oxo-1,4-dihydroquinoline-2-carboxylate using diethyl 2-[(4-fluorophenylamino)methylene]malonate (1.41 g, 5.0 mmol, 1.0 equiv.).

White solid (0.691 g, 59%); m.p. >250 °C (lit.[5] 296–298 °C); ^{1}H NMR (600 MHz, $(CD_3)_2SO$) δ: 8.59 (s, 1H), 7.80 (dd, $^{3}J_{(H,F)}$ = 9.3 Hz, *J* = 2.9 Hz, 1H), 7.71 (dd, *J* = 9.0, 4.6 Hz, 1H, C(8)**H**), 7.62 (td, *J* = 8.8, 2.9 Hz, 1H), 4.22 (q, *J* = 7.1 Hz, 2H), 1.28 (t, *J* = 7.1 Hz, 3H) ppm; ^{13}C{^{1}H} NMR (150 MHz, $(CD_3)_2SO$) δ: 172.6, 164.9, 159.2 (d, *J* = 243 Hz), 145.3, 136.1, 128.8 (d, *J* = 7 Hz), 122.0 (d, *J* = 8 Hz), 121.0 (d, *J* = 25 Hz), 110.0 (d, *J* = 23 Hz), 109.0, 59.7, 14.4 ppm; ^{19}F NMR (282 MHz, $(CD_3)_2SO$) δ: -116 ppm; *m/z* (ES-): 234 ($(M\text{-}H)^-$ 100%).

Borylation of quinolones

1,2-Dimethyl-6/7-(4,4,5,5-tetramethyl-1,3,2-dioxaborolan-2-yl)quinolin-4(1*H*)-one (S2A, S2B)

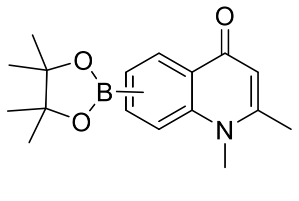

In an argon-filled glovebox, $[Ir(OMe)COD]_2$ (2.0 mg, 0.003 mmol, 1.5 mol%), dtbpy (1.6 mg, 0.006 mmol, 3 mol%), B_2pin_2 (50.8 mg, 0.2 mmol, 1.0 equiv.), **S1** (34.6 mg, 0.2 mmol, 1.0 equiv.), and THF (10 mL) were added to a sealable cylindrical microwave vial. The vial was sealed with a crimptop septum cap, taken out of the glovebox and the reaction mixture was stirred in an aluminium heating block at 80

°C for 24 h. The reaction was cooled to r.t. and concentrated under reduced pressure to afford the crude product. Analysis acquired from a mixture of isomers.

1,2-Dimethyl-7-(4,4,5,5-tetramethyl-1,3,2-dioxaborolan-2-yl)quinolin-4(1*H*)-one (S2A)

^{1}H NMR (500 MHz, $CDCl_3$) δ: 8.43 (dd, *J* = 7.9 Hz, 0.5 Hz, 1H), 7.96 (s, 1H), 7.77 (dd, *J* = 7.9, 0.5 Hz, 1H), 6.29 (s, 1H), 3.81 (s, 3H), 2.50 (d, *J* = 0.5 Hz, 3H) ppm; ^{13}C{^{1}H} NMR (125 MHz, $CDCl_3$) δ: 177.9, 151.5, 141.3, 129.2, 128.5, 126.0, 122.1, 112.2, 84.6, 34.7, 24.8, 22.5 ppm; a signal for the carbon directly attached to the boron atom was not observed; ^{11}B NMR (160 MHz $CDCl_3$) *δ* 31.0 ppm; HRMS (ASAP+): *m/z* calcd for $[M+H]^+$ 299.1802, found 299.1795.

1,2-Dimethyl-6-(4,4,5,5-tetramethyl-1,3,2-dioxaborolan-2-yl)quinolin-4(1*H*)-one (S2B)

^{1}H NMR (500 MHz, $CDCl_3$) δ: 8.93 (dd, *J* = 1.7 Hz, 0.5 Hz, 1H), 8.03 (dd, *J* = 8.7, 1.7 Hz, 1H), 7.45 (d, *J* = 8.7, 1H), 6.27 (s, 1H), 3.74 (s, 3H), 2.48 (d, *J* = 0.5 Hz, 3H) ppm; ^{13}C{^{1}H} NMR (125 MHz, $CDCl_3$) δ: 178.1, 151.2, 143.8, 137.9, 134.8, 125.9, 114.7, 112.6, 84.2, 34.5, 25.1, 22.4 ppm; a signal for the carbon directly attached to the boron atom was not observed; ^{11}B NMR (160 MHz $CDCl_3$) *δ* 31.0 ppm; HRMS (ASAP+): *m/z* calcd for $[M+H]^+$ 299.1802, found 299.1795.

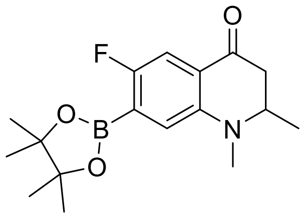

6-Fluoro-1,2-dimethyl-7-(4,4,5,5-tetramethyl-1,3,2-dioxaborolan-2-yl)-2,3-dihydroquinolin-4(1*H*)-one (S4B)

Prepared using the general procedure using 6-fluoro-1,2-dimethylquinolin-4(1*H*)-one (57.4 mg, 0.3 mmol, 1.0 equiv.) and B_2pin_2 (114.3 mg, 0.45 mmol, 1.5 equiv.) and isolated *via* column chromatography (DCM:MeOH).

^{1}H NMR (500 MHz, $CDCl_3$) δ: 7.50 (d, *J*= 9.1 Hz, 1H), 6.97 (d, *J*= 4.2 Hz, 1H), 3.77–3.62 (m, 1H), 3.05–2.91 (m, 4H), 2.52 (dd, *J* = 16.3, 3.3 Hz, 1H), 1.36 (s, 12H), 1.12 (d, *J* = 6.7 Hz) ppm; ^{13}C{^{1}H} NMR (125 MHz, $CDCl_3$) δ: 193.3 (d, *J*= 2 Hz), 159.0 (d, *J*= 241 Hz), 146.3 (d, *J*= 1 Hz), 121.7 (d, *J*= 6 Hz), 121.4 (d, *J*= 7 Hz), 112.3 (d, *J*= 26 Hz), 84.4, 56.9, 44.9, 37.7, 24.9, 14.1 ppm; a signal for the carbon directly attached to the boron atom was not observed; ^{19}F NMR (470 MHz, $CDCl_3$) δ: -120 ppm; ^{11}B NMR (96 MHz, $CDCl_3$) δ: 30 ppm; HRMS (ESI-TOF) *m/z*: $[M+H]^+$ calcd for $C_{17}H_{24}BFNO_3$: 320.1828; found: 320.1830.

The compound was not sufficiently pure for complete analysis or calculation of yield.

Borylation and subsequent bromination of 6-fluoroquinolines

A 15 mL Schlenk was oven dried (150 °C) and cooled under vacuum. The Schlenk flask was refilled with nitrogen and all reagents were added under a positive pressure of nitrogen in the order: quinoline (1.0 equiv.), dtbpy (3 mol%), B_2pin_2 (1.1 equiv.), and $[Ir(OMe)COD]_2$ (1.5 mol%). The Schlenk flask was then placed under vacuum for 20 mins before being refilled with nitrogen three times. THF (2.5 mL/mmol) was added *via* syringe through the septum, the reaction was sealed and the mixture was heated to 80 °C in an aluminium heating block for 12-18 h. The reaction mixture was then cooled to r.t., diluted with Et_2O and concentrated under reduced pressure. An internal standard, 1,3,5-trimethoxybenzene (~10 mol%) was added to the residue to determine the yield of the C7-borylated product **2a**–**2e** by ^{1}H NMR analysis. The residue was redissolved in MeOH (20 mL/mmol) and a solution of $CuBr_2$ (3.5 equiv.) in H_2O (20 mL/mmol) was added. The reaction mixture was heated to 80 °C in an oil bath for 3 h, cooled to r.t. diluted with 10% NH_4OH (40 mL/mmol) and then extracted with Et_2O (3 x 10 mL/mmol). The combined organic layers were washed with H_2O (10 mL/mmol) and brine (10 mL/mmol), dried over $MgSO_4$, filtered and concentrated under reduced pressure. The product was purified *via* column chromatography (DCM:EtOAc, unless otherwise specified).

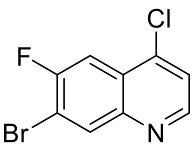

7-Bromo-4-chloro-6-fluoroquinoline (S7A)

Prepared *via* the general procedure using **1a** (36.3 mg, 0.2 mmol, 1.0 equiv.). ^{1}H NMR (500 MHz, $CDCl_3$) δ: 8.76 (d, *J* = 4.7 Hz, 1H), 8.41 (d, *J* = 6.6 Hz, 1H), 7.92 (d, *J* = 9.0 Hz, 1H), 7.53 (d, *J* = 4.7 Hz, 1H) ppm; ^{13}C{^{1}H} NMR (125 MHz, $CDCl_3$) δ: 157.4 (d, *J* = 251 Hz), 150.1 (d, *J* = 3 Hz), 146.3 (d, *J* = 1 Hz), 141.9 (d, *J* = 6 Hz), 135.1 (d, *J* = 1 Hz), 126.7 (d, *J* = 9 Hz), 122.0, 114.7 (d, *J* = 25 Hz), 108.8 (d, *J* = 26 Hz) ppm; ^{19}F NMR (470 MHz, $CDCl_3$) δ: -106 ppm; HRMS (ESI-TOF) *m/z*: $[M+H]^+$ calcd for $C_9H_5BrClFN$: 259.9272; found: 259.9277.

The compound was not sufficiently pure for complete analysis or calculation of yield.

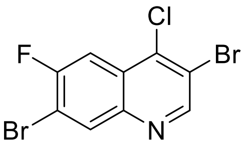

3,7-Dibromo-4-chloro-6-fluoroquinoline (S7B)

Prepared *via* the general procedure using **1a** (36.3 mg, 0.2 mmol, 1.0 equiv.).

^{1}H NMR (500 MHz, $CDCl_3$) δ: 8.90 (s, 1H), 8.37 (d, *J* = 6.6 Hz, 1H), 7.92 (d, *J* = 9.1 Hz, 1H) ppm; ^{13}C{^{1}H} NMR (125 MHz, $CDCl_3$) δ: 157.0 (d, *J* = 252 Hz), 151.1 (d, *J* = 3 Hz), 143.4 (d, *J* = 2 Hz),

139.7 (d, *J* = 5 Hz), 134.2 (d, *J* = 2 Hz), 126.6 (d, *J* = 9 Hz), 118.3, 113.5 (d, *J* = 25 Hz), 108.3 (d, *J* = 26 Hz) ppm; ^{19}F NMR (470 MHz, $CDCl_3$) δ: -104 ppm; HRMS (ESI-TOF) *m/z*: $[M+H]^+$ calcd for $C_9H_4Br_2ClFN$: 337.8378; found: 337.8383.

The compound was not sufficiently pure for complete analysis or calculation of yield.

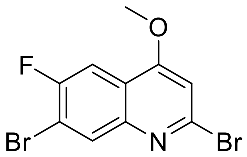

2,7-Dibromo-6-fluoro-4-methoxyquinoline (S9)

Prepared *via* the general procedure using **1f** (35.4 mg, 0.2 mmol, 1.0 equiv.).

^{1}H NMR (400 MHz, $CDCl_3$) δ: 8.21 (d, *J* = 6.4 Hz, 1H), 7.92 (d, *J* = 9.1 Hz, 1H), 8.90 (s, 1H) ppm; ^{13}C{^{1}H} NMR (100 MHz, $CDCl_3$) δ: 162.4 (d, *J* = 5 Hz), 156.5 (d, *J* = 249 Hz), 145.8 (d, *J* = 2 Hz), 142.8 (d, *J* = 3 Hz), 133.3), 120.5 (d, *J* = 9 Hz), 114.6 (d, *J* = 24 Hz), 107.3 (d, *J* = 25 Hz), 105.5, 56.4 ppm; ^{19}F NMR (376 MHz, $CDCl_3$) δ: -108 ppm; HRMS (ESI-TOF) *m/z*: $[M+H]^+$ calcd for $C_{10}H_7Br_2FNO$: 333.8873; found: 333.8870.

The compound was not sufficiently pure for complete analysis or calculation of yield.

Single-crystal X-ray diffraction

Crystal structure determination

The crystals of compounds **2a** and **9a** were grown by vapor diffusion by dissolving the compound in DCM in a small vial and placing that vial in a larger vial containing Et_2O. Crystals suitable for single-crystal X-ray diffraction were selected, coated in perfluoropolyether oil, and mounted on a polyimide microloop. Diffraction data of **2a** and **9a** were collected on a RIGAKU OXFORD DIFFRACTION XTALAB SYNERGY-R diffractometer with a semiconductor HPA area detector (HyPix-Arc150) using a rotating-anode X-ray tube for X-ray generation and multi-layer mirror monochromated Cu-K_α radiation. The crystals were cooled using an Oxford Cryostreams 800 low-temperature device. Data were collected at 100 K. The images were processed and corrected for Lorentz-polarization effects and absorption as implemented in the CrysAlis^{Pro} software. The structure was solved using the intrinsic phasing method (SHELXT)[6] and Fourier expansion technique. All non-hydrogen atoms were refined in anisotropic approximation, with hydrogen atoms 'riding' on idealised positions by full-matrix least squares against F^2 of all data, using SHELXL software[7] and the SHELXLE graphical user interface.[8] The crystal structure of **2a** was refined as an inversion twin and the twin fraction refined to 47.2%. The unit cell of **2a** contains disordered diethyl ether solvent molecules which have been treated as a diffuse contribution to the overall scattering without specific atom positions by SQUEEZE/PLATON.[9] Diamond[10] software was used for graphical representation. Crystal data and experimental details are listed in **Table S2**; full structural information has been deposited with Cambridge Crystallographic Data Centre. CCDC-2159956 (**2a**) and 2159957 (**9a**).

Table S2 Single-crystal X-ray diffraction data and refinement details of compounds **2a** and **9a.**

Data	**2a**	**9a**
CCDC number	2159956	2159957
Empirical formula	$C_{16}H_{18}BClFNO_2$ [+ diethyl ether solvent]	$C_{19}H_{15}ClFNO_2$
Formula weight / g·mol^{-1}	321.57	343.77
T / K	100(2)	100(2)
Radiation, λ / Å	Cu-K$_\alpha$ 1.54184	Cu-K$_\alpha$ 1.54184
Crystal size / mm^3	0.179×0.083×0.027	0.432×0.055×0.042
Crystal color, habit	colorless plate	colorless needle
μ / mm^{-1}	2.102	2.351
Crystal system	orthorhombic	monoclinic
Space group	$P2_12_12_1$	$P2_1/n$
a / Å	7.1800(1)	3.81971(14)
b / Å	16.3318(1)	21.4427(10)
c / Å	29.2511(3)	19.2195(6)
α / °	90	90
β / °	90	93.267(3)
γ / °	90	90
Volume / Å^3	3430.05(6)	1571.61(11)
Z	8	4
ρ_{calc} / g·cm^{-3}	1.245	1.453
F(000)	1344	712
θ range / °	3.021 – 74.486	3.091 – 74.492
Reflections collected	45414	15732
Unique reflections	6984	3181
Parameters	408	219
GooF on F^2	1.036	1.092
R_1 [I>2σ(I)]	0.0303	0.0409
wR_2 (all data)	0.0799	0.1162
Max. / min. residual electron density / e·Å^{-3}	0.211 / –0.231	0.336 / –0.437

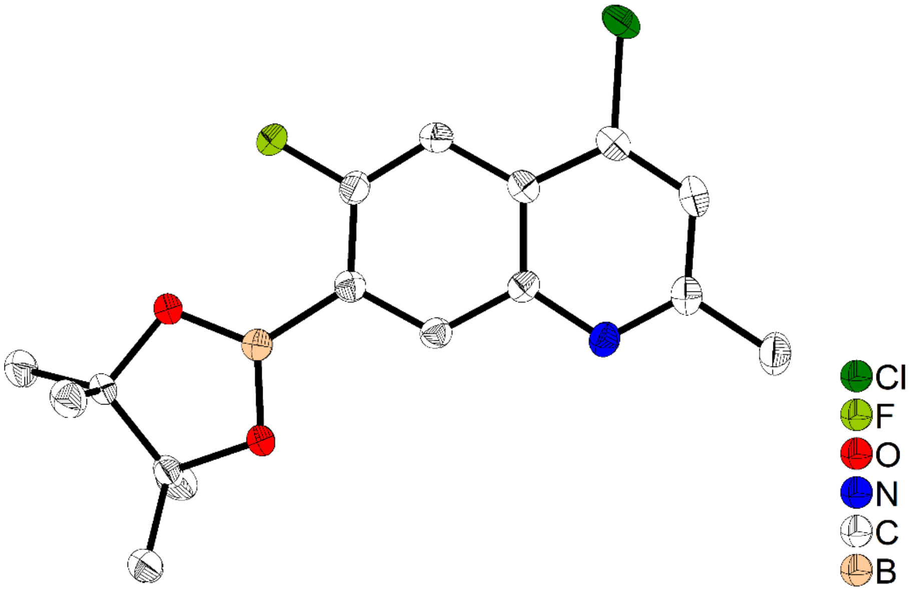

Figure S1 The solid-state molecular structure of **2a** determined by single-crystal X-ray diffraction at 100 K. All ellipsoids are drawn at the 50% probability level. H atoms are omitted for clarity. Only one of two symmetrically independent molecules is shown here.

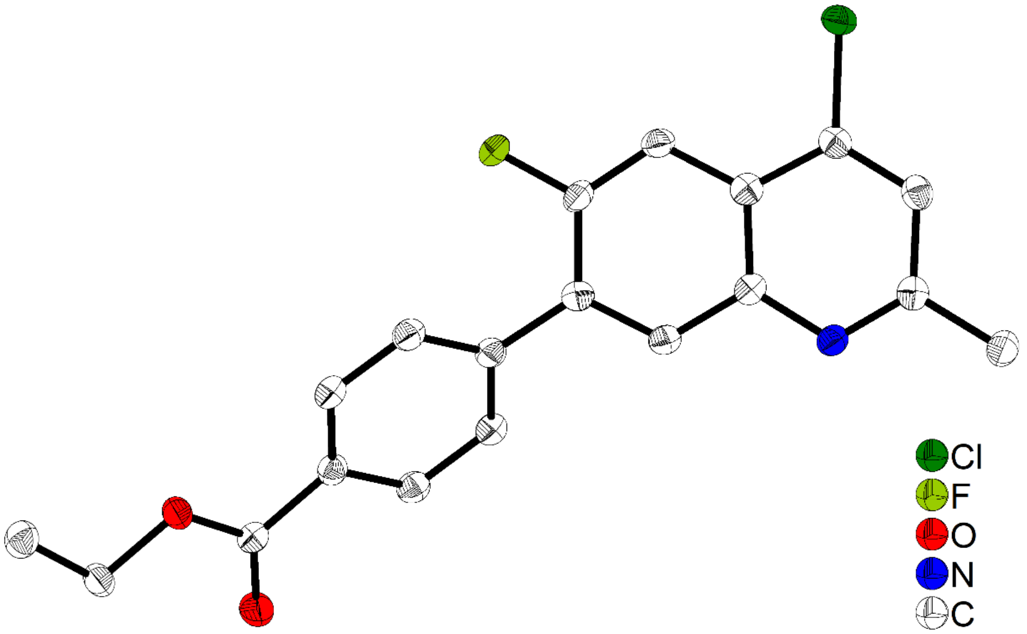

Figure S2 The solid-state molecular structure of **9a** determined by single-crystal X-ray diffraction at 100 K. All ellipsoids are drawn at the 50% probability level. H atoms are omitted for clarity.

^{1}H NMR and $^{13}C\{^{1}H\}$ NMR Spectra

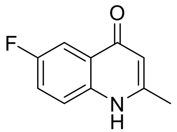

^{1}H NMR (300 MHz, $(CD_3)_2SO$)

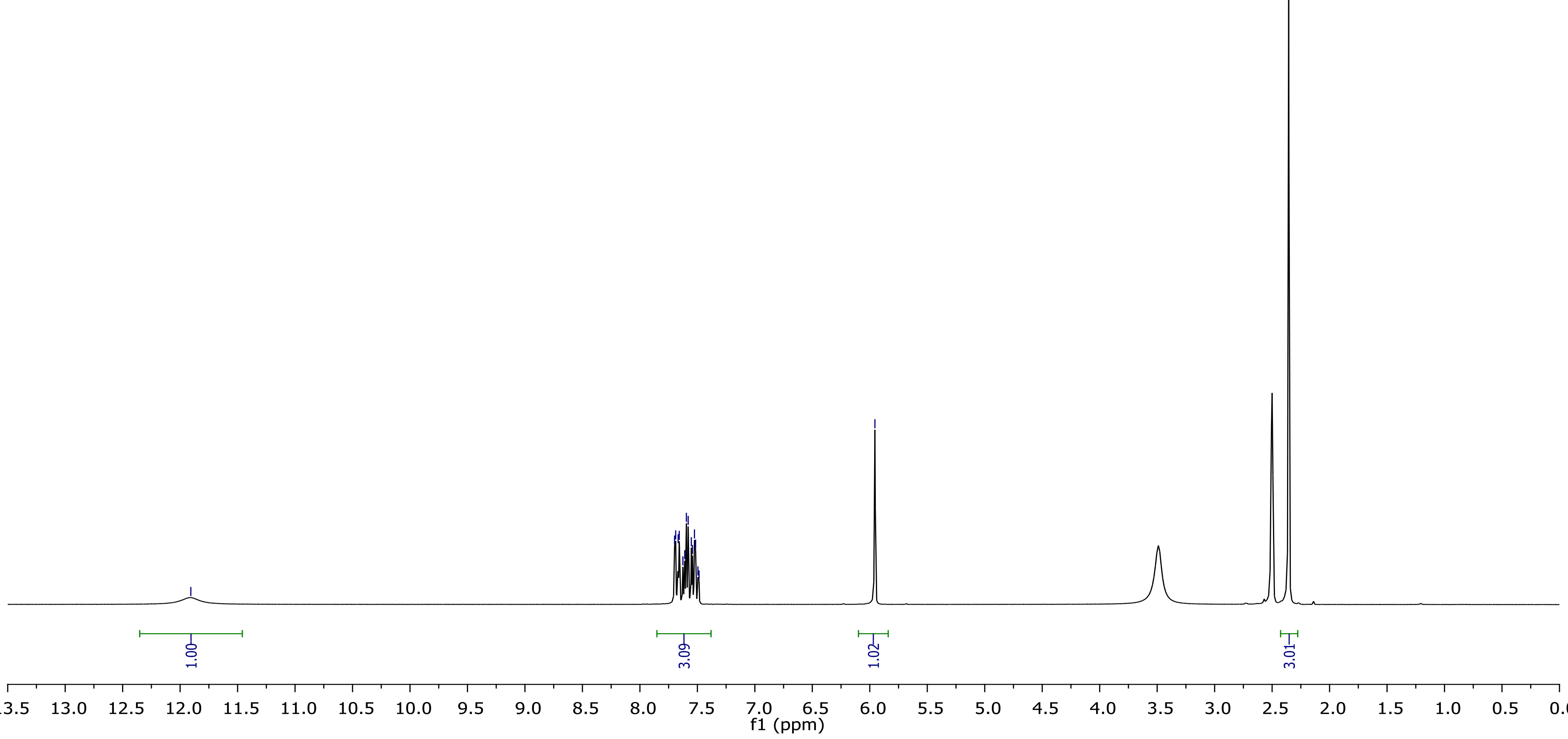

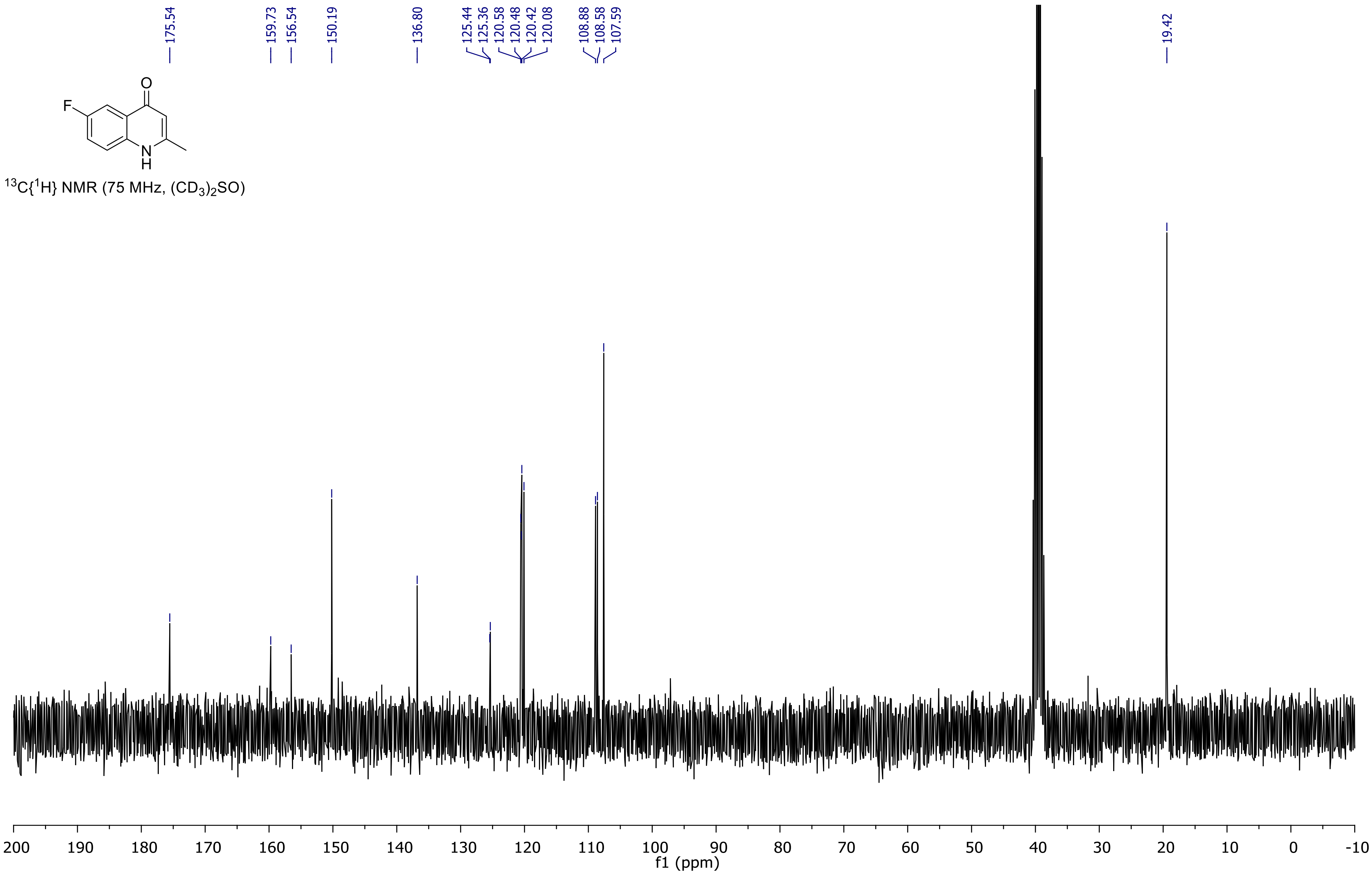

F
O
N
H
$^{13}C\{^1H\}$ NMR (75 MHz, $(CD_3)_2SO$)
175.54
159.73
156.54
150.19
136.80
125.44
125.36
120.58
120.48
120.42
120.08
108.88
108.58
107.59
19.42
200 190 180 170 160 150 140 130 120 110 100 90 80 70 60 50 40 30 20 10 0 -10
f1 (ppm)

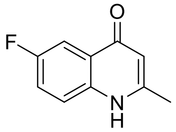

^{19}F NMR (282 MHz, $(CD_3)_2SO$)

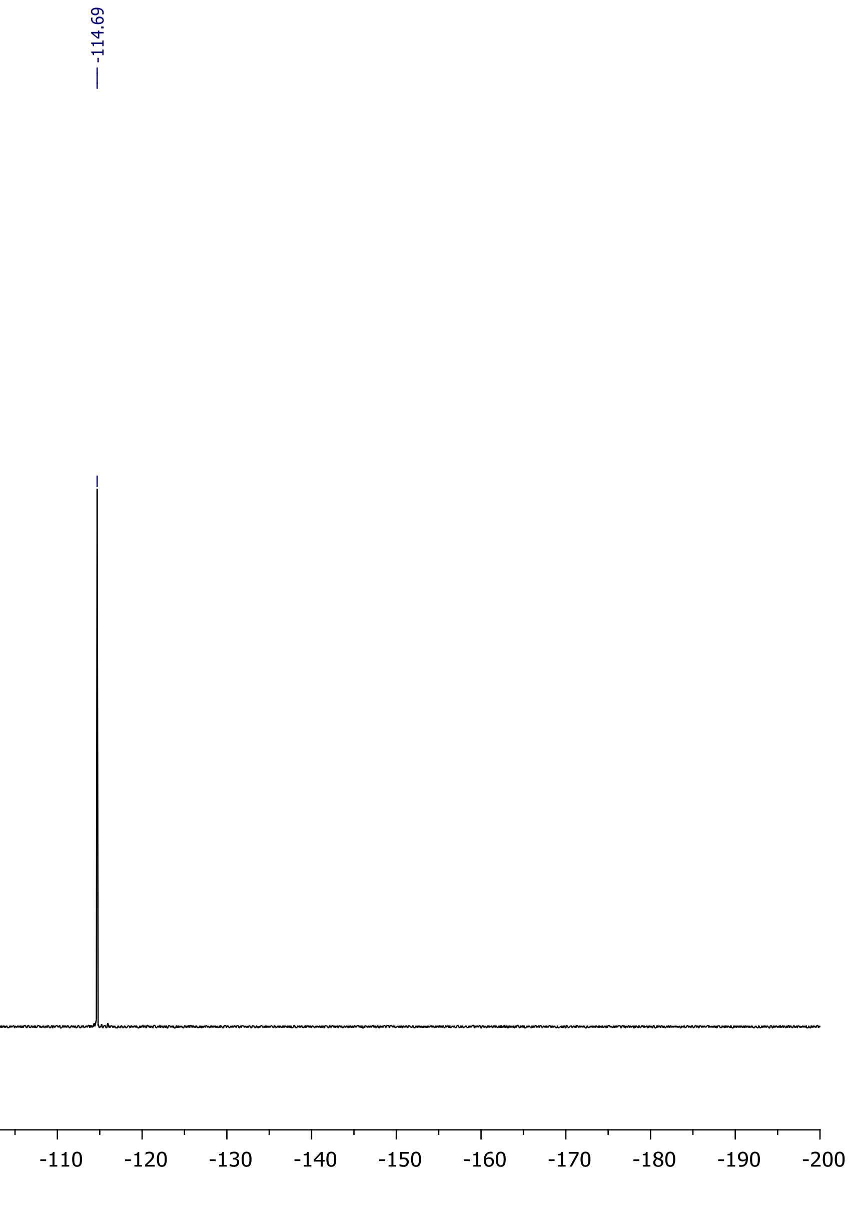

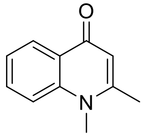

S1

^{1}H NMR (300 MHz, $CDCl_3$)

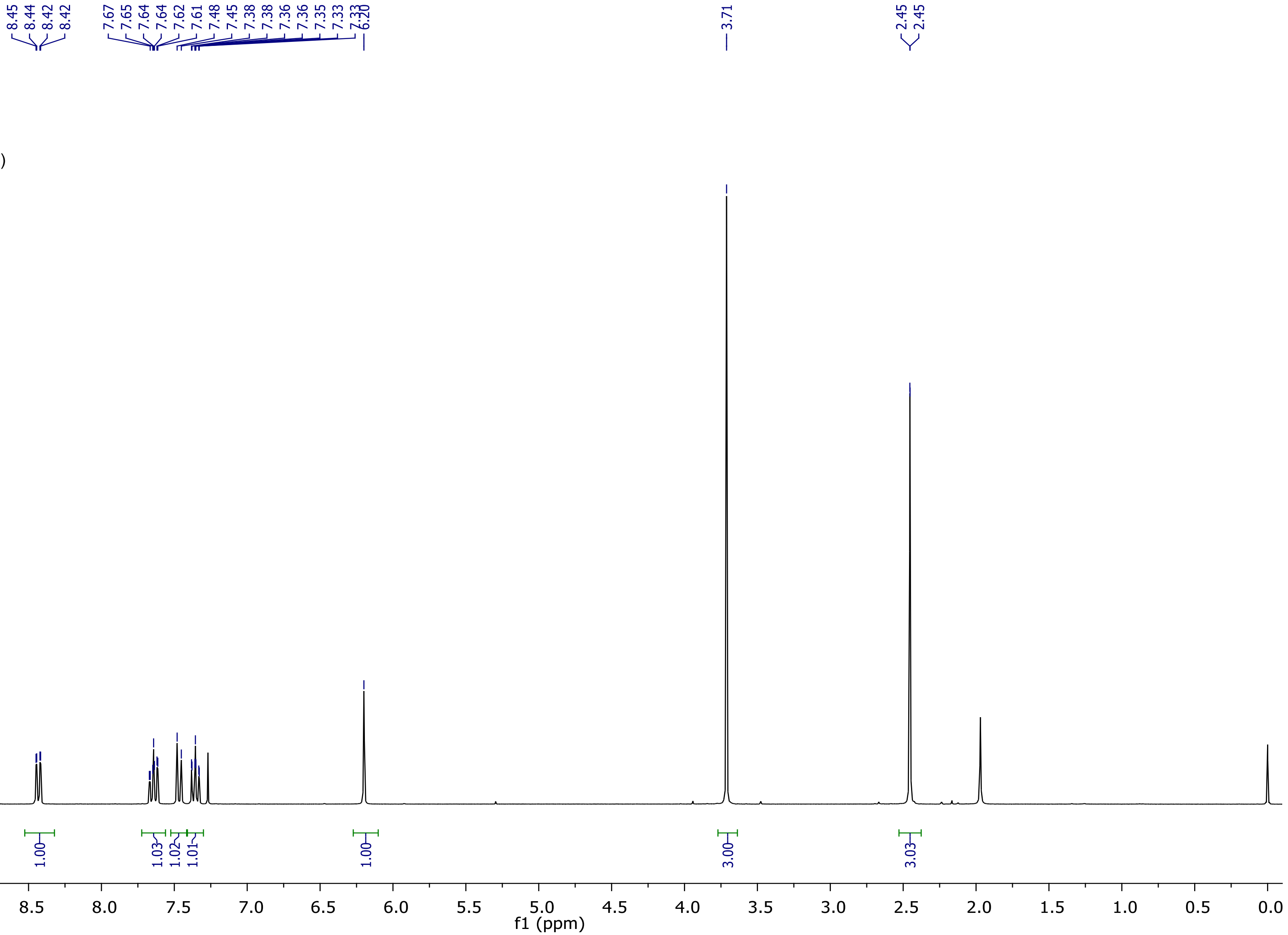

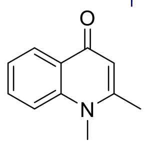

S1

$^{13}C\{^1H\}$ NMR (75 MHz, $CDCl_3$)

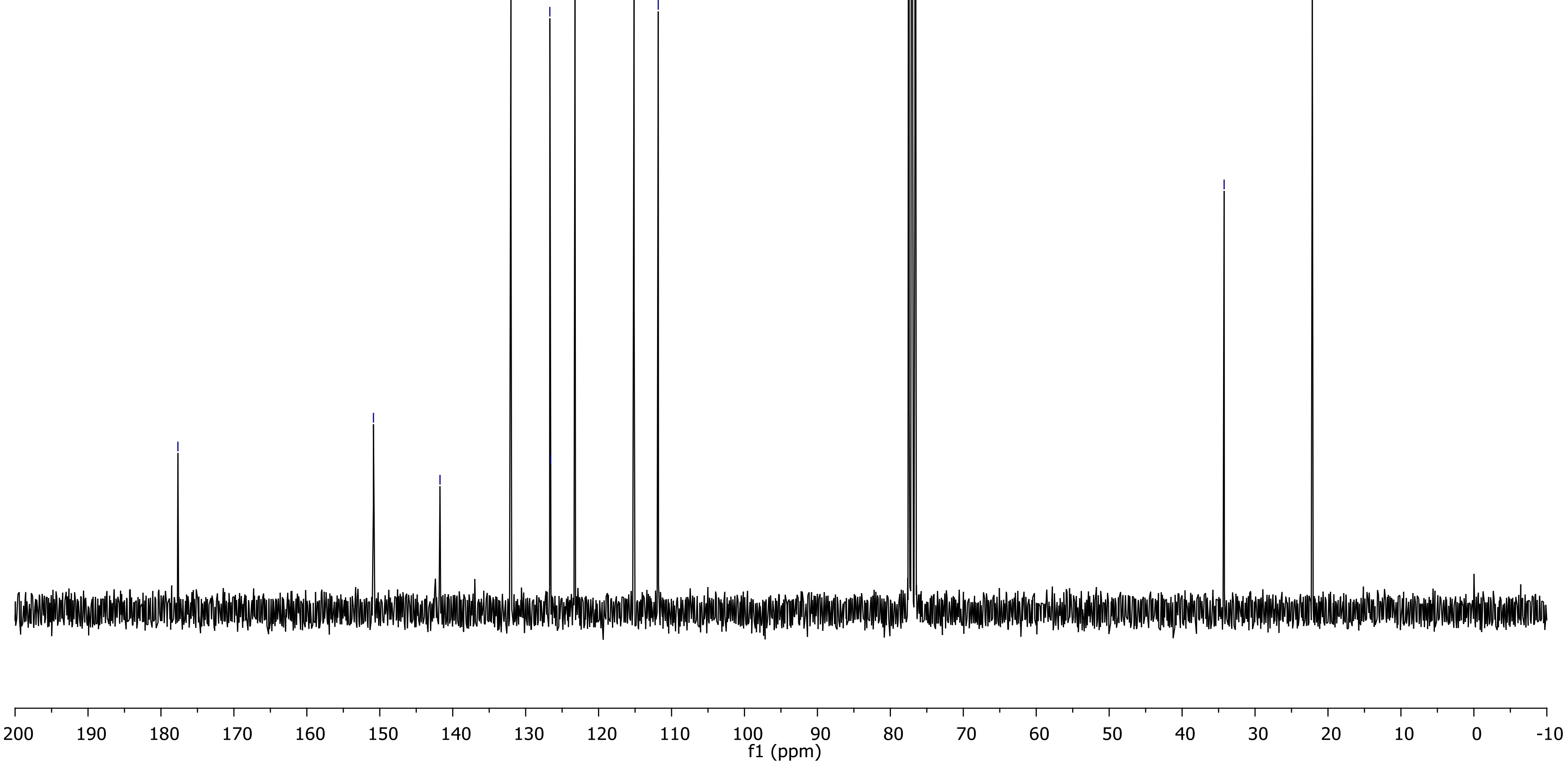

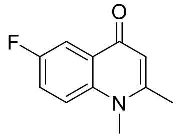

S3

^{1}H NMR (300 MHz, CD_3OD)

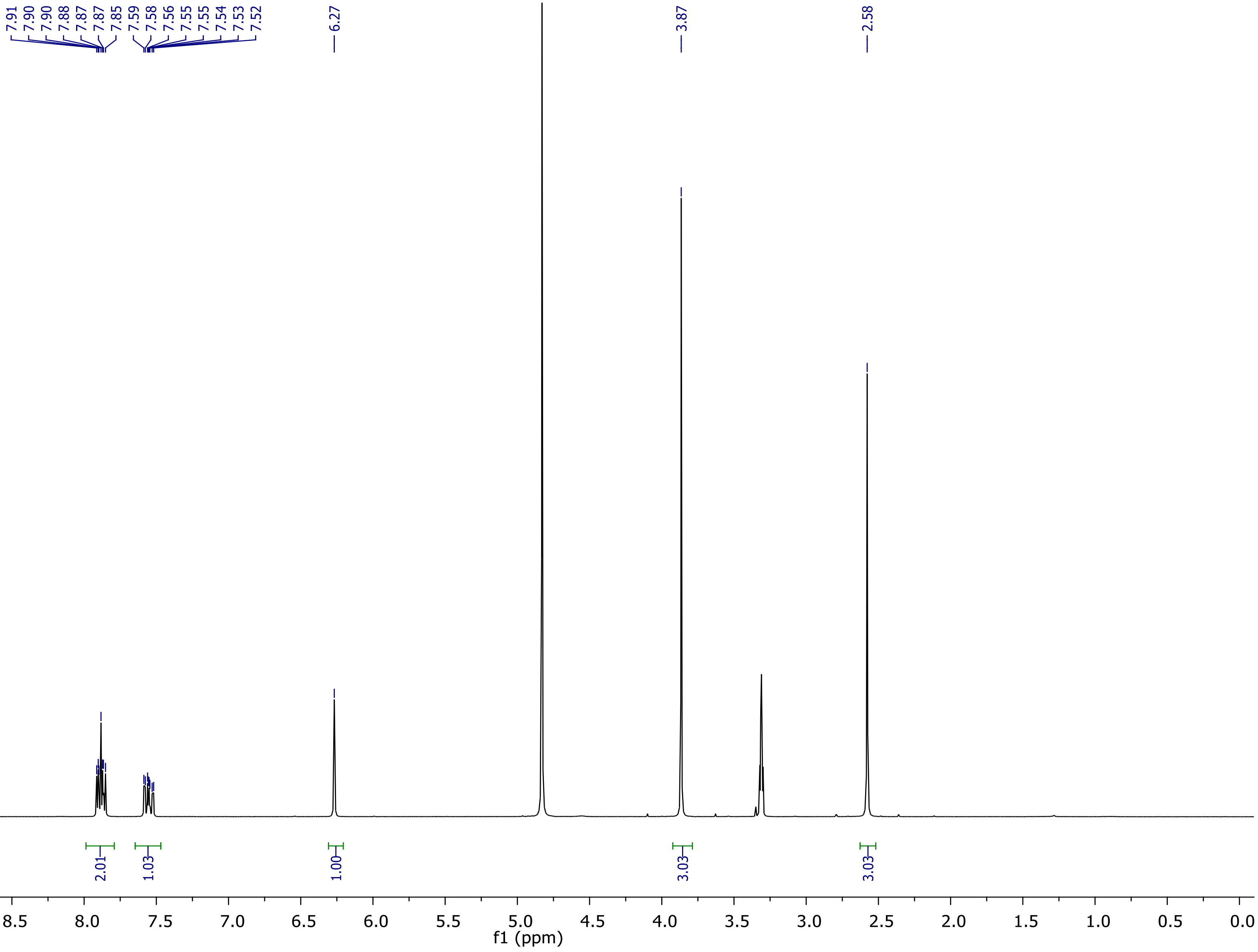

S3

$^{13}C\{^{1}H\}$ NMR (75 MHz, CD_3OD)

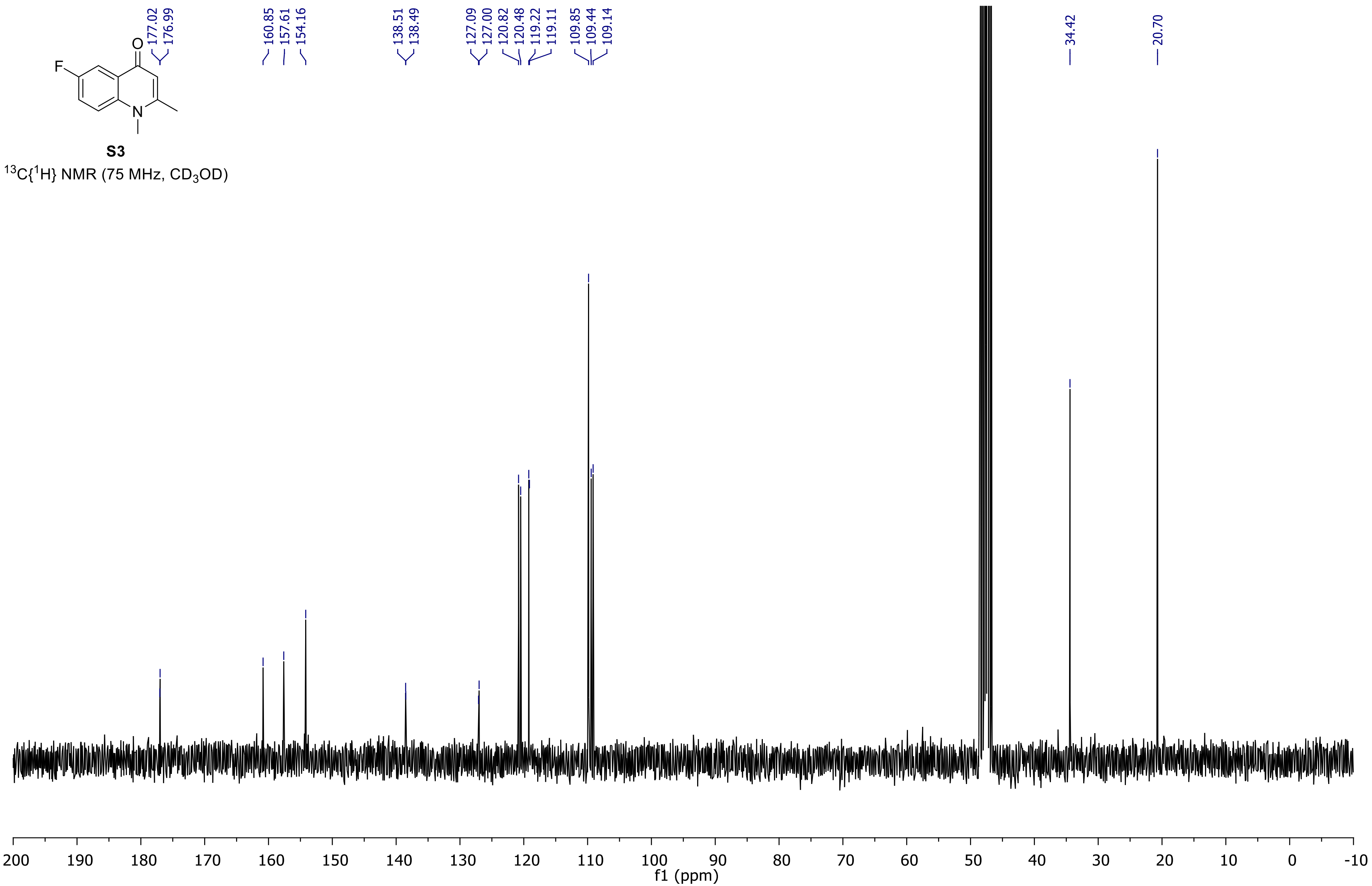

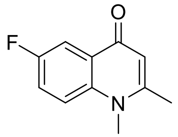

S3

^{19}F NMR (282 MHz, CD_3OD)

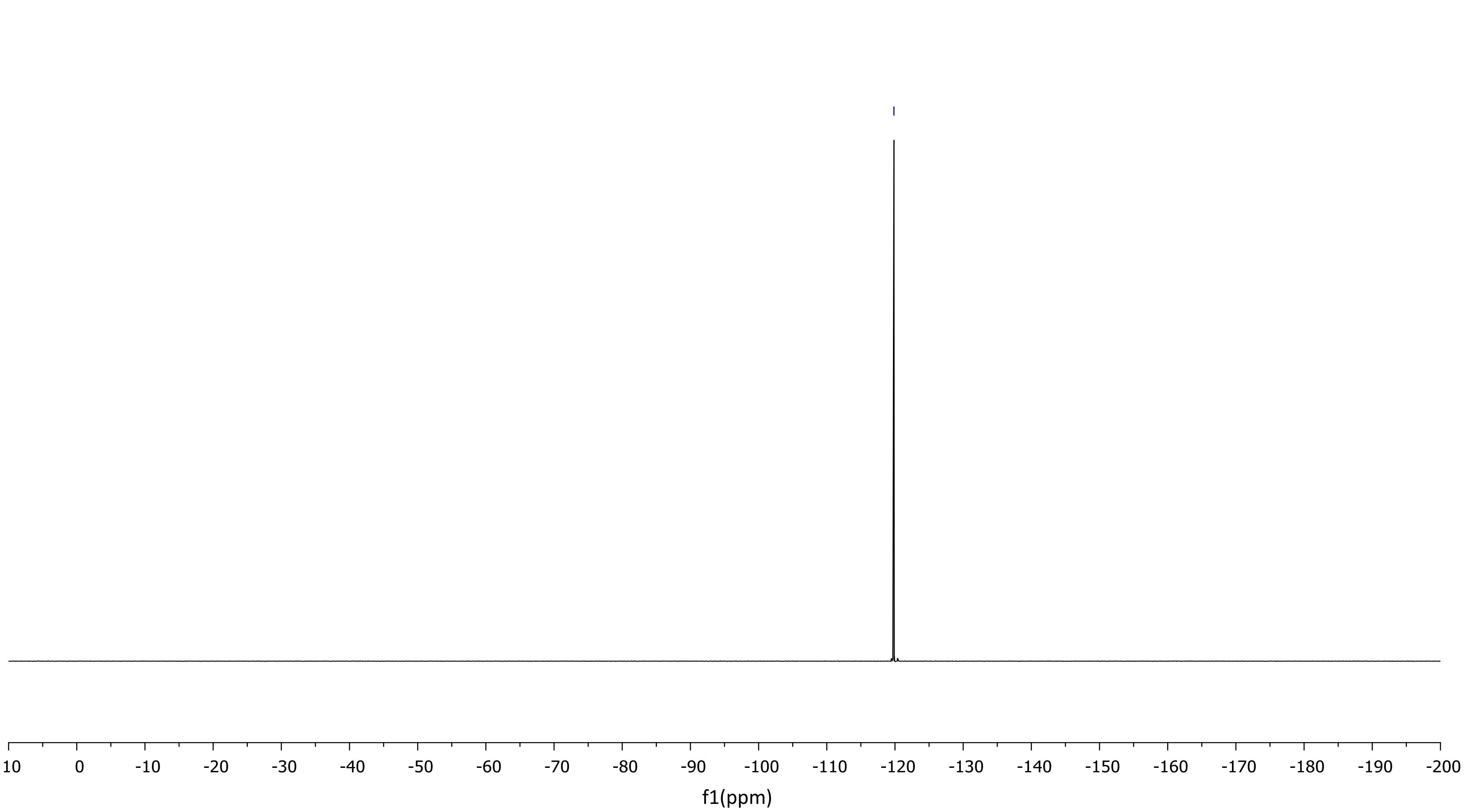

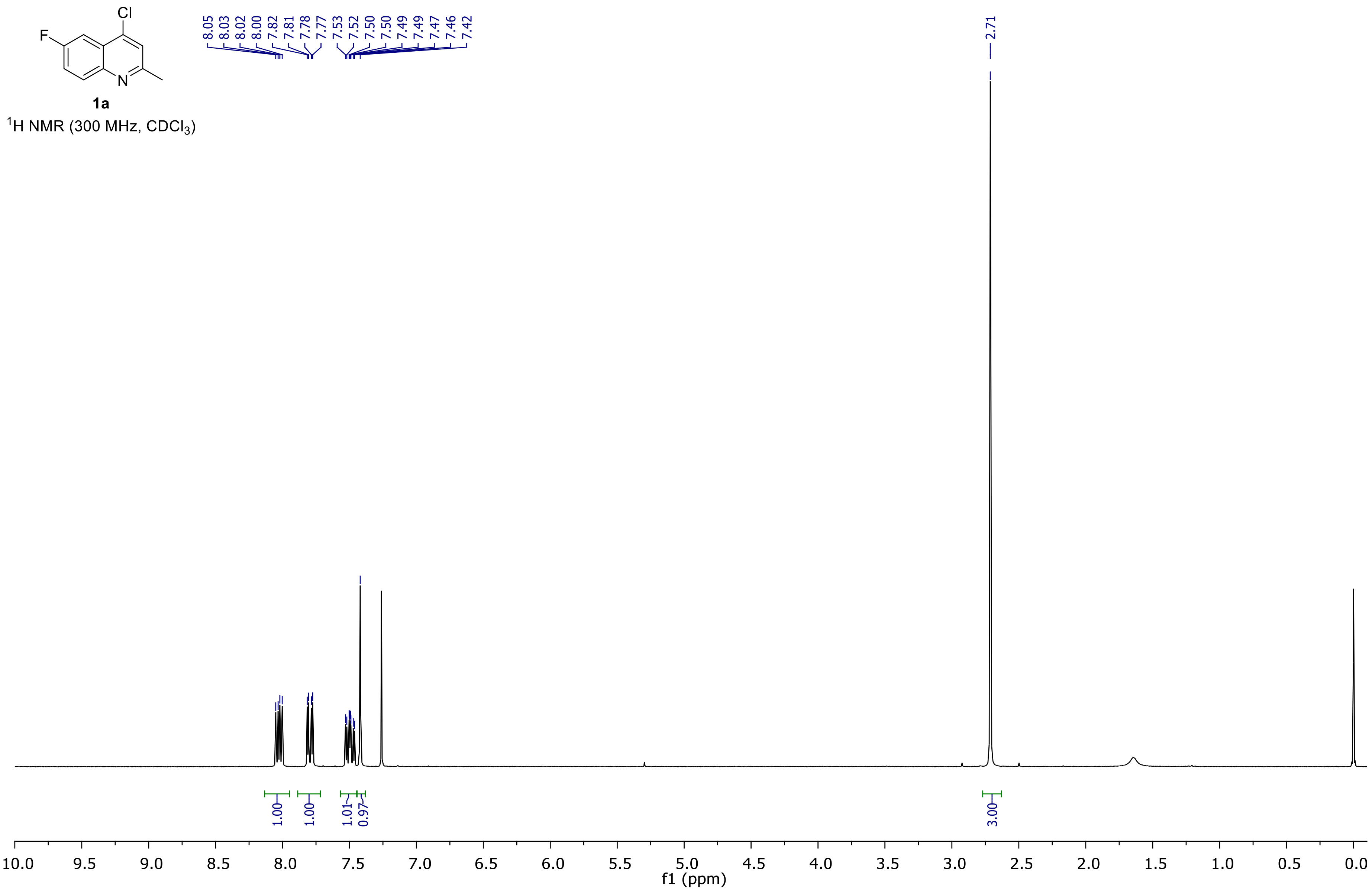
Cl
F
N
1a
^{1}H NMR (300 MHz, $CDCl_3$)
8.05
8.03
8.02
8.00
7.82
7.81
7.78
7.77
7.53
7.52
7.50
7.50
7.49
7.49
7.47
7.46
7.42
2.71
1.00
1.00
1.01
0.97
3.00
10.0 9.5 9.0 8.5 8.0 7.5 7.0 6.5 6.0 5.5 5.0 4.5 4.0 3.5 3.0 2.5 2.0 1.5 1.0 0.5 0.0
f1 (ppm)

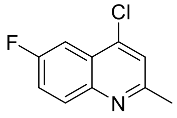

1a

$^{13}C\{^1H\}$ NMR (75 MHz, $CDCl_3$)

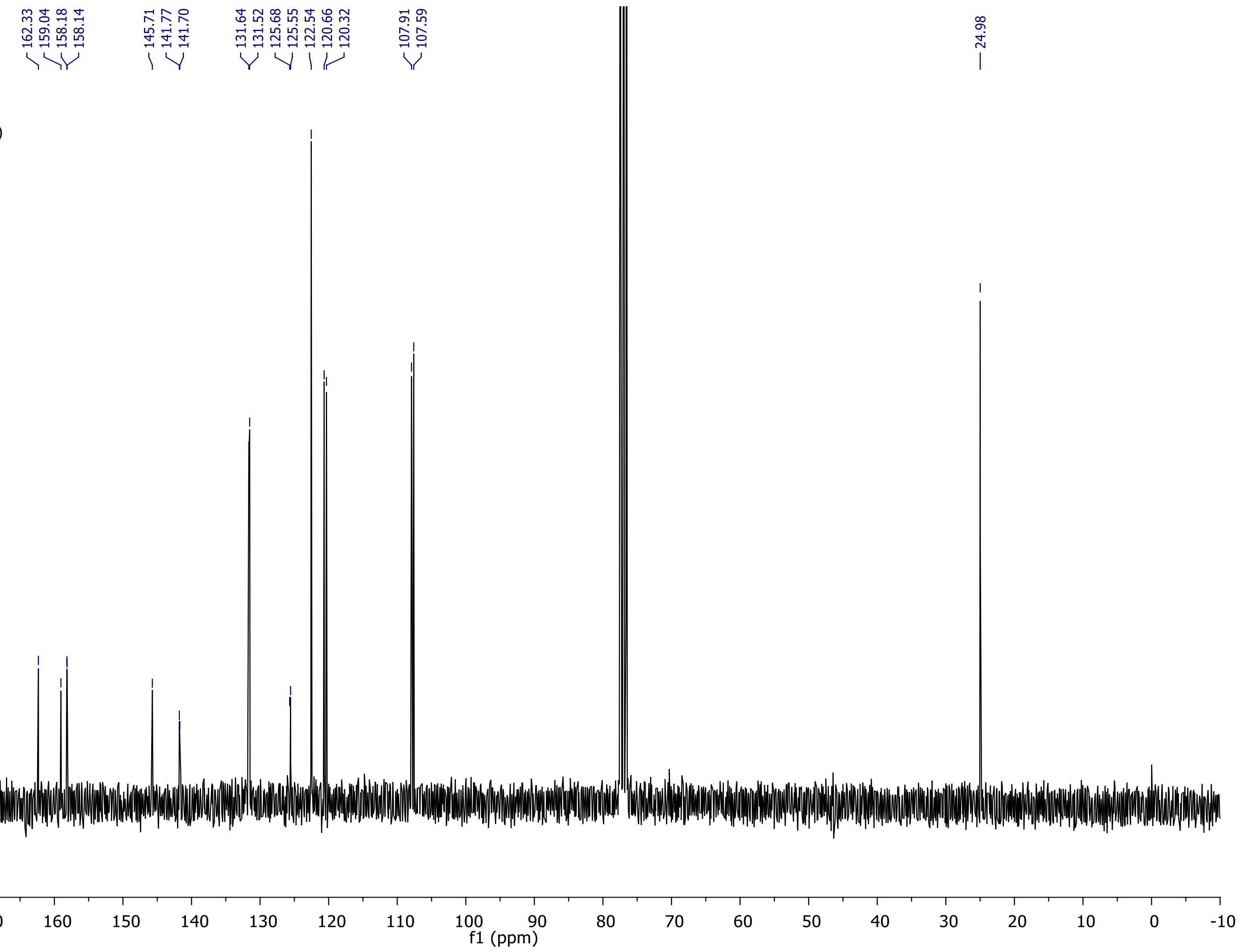

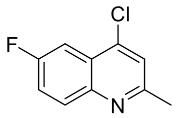

1a

^{19}F NMR (282 MHz, $CDCl_3$)

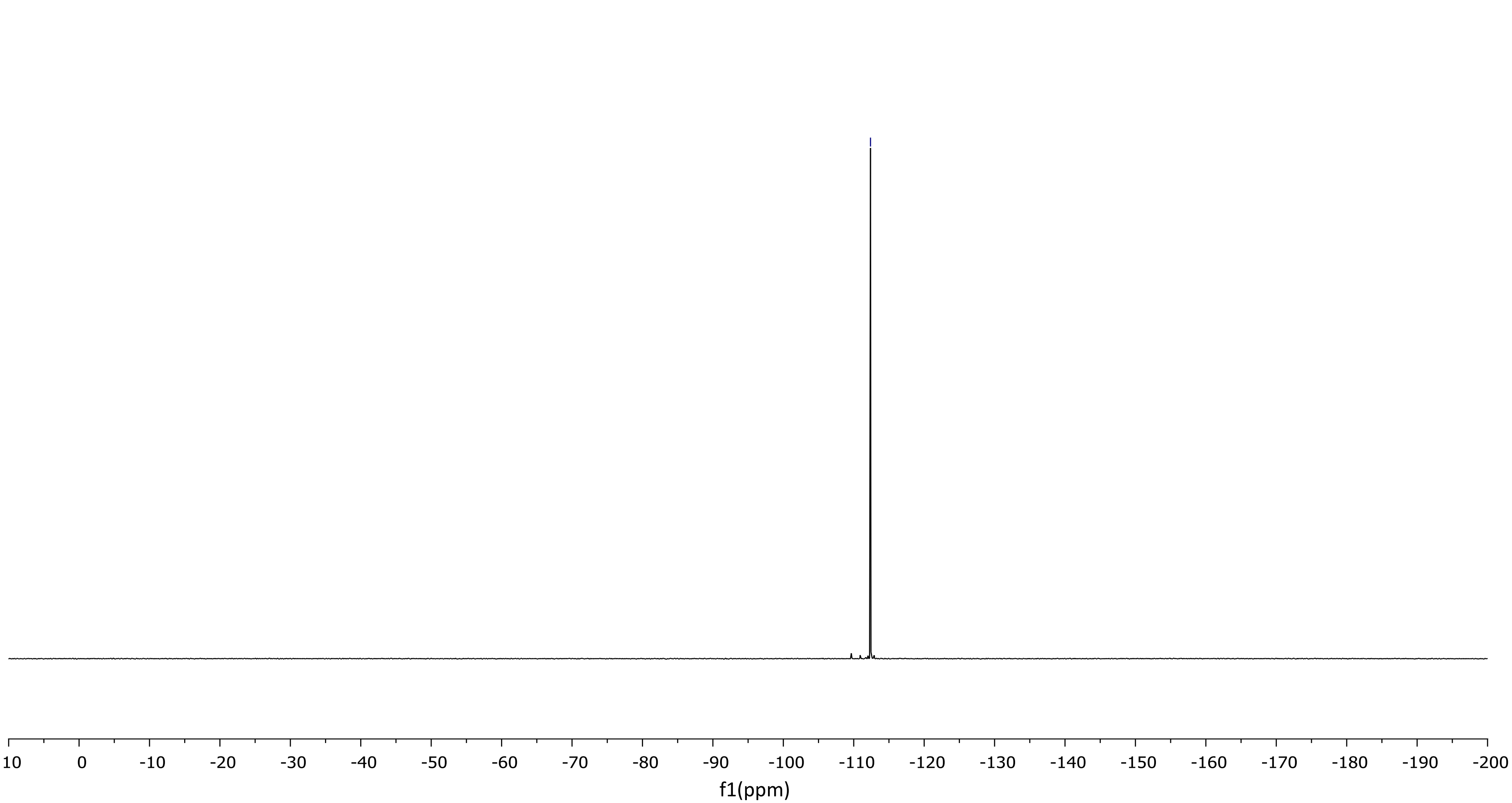

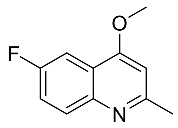

1b

^{1}H NMR (300 MHz, $CDCl_3$)

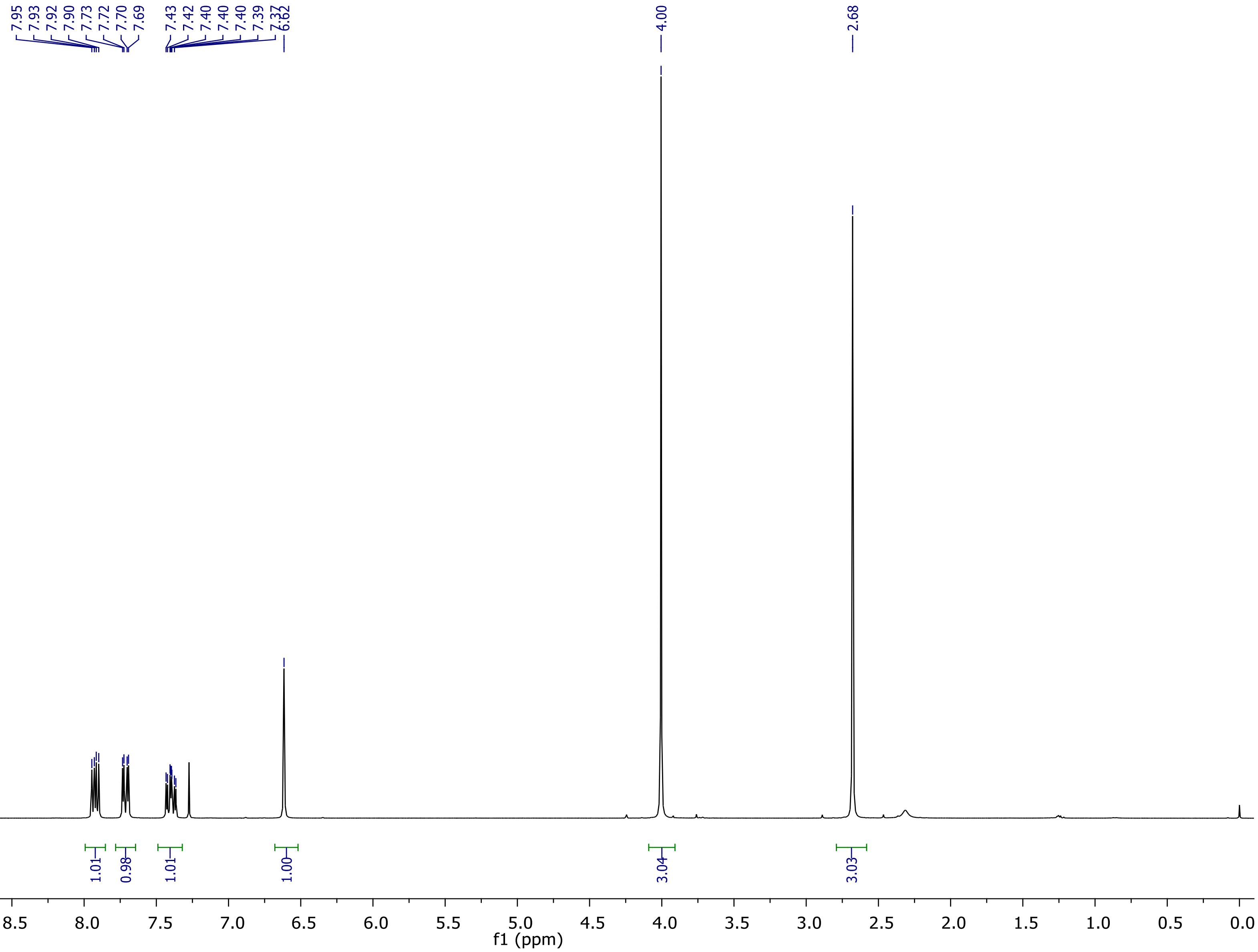

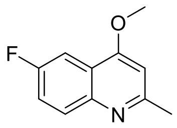

1b

$^{13}C\{^{1}H\}$ NMR (75 MHz, $CDCl_3$)

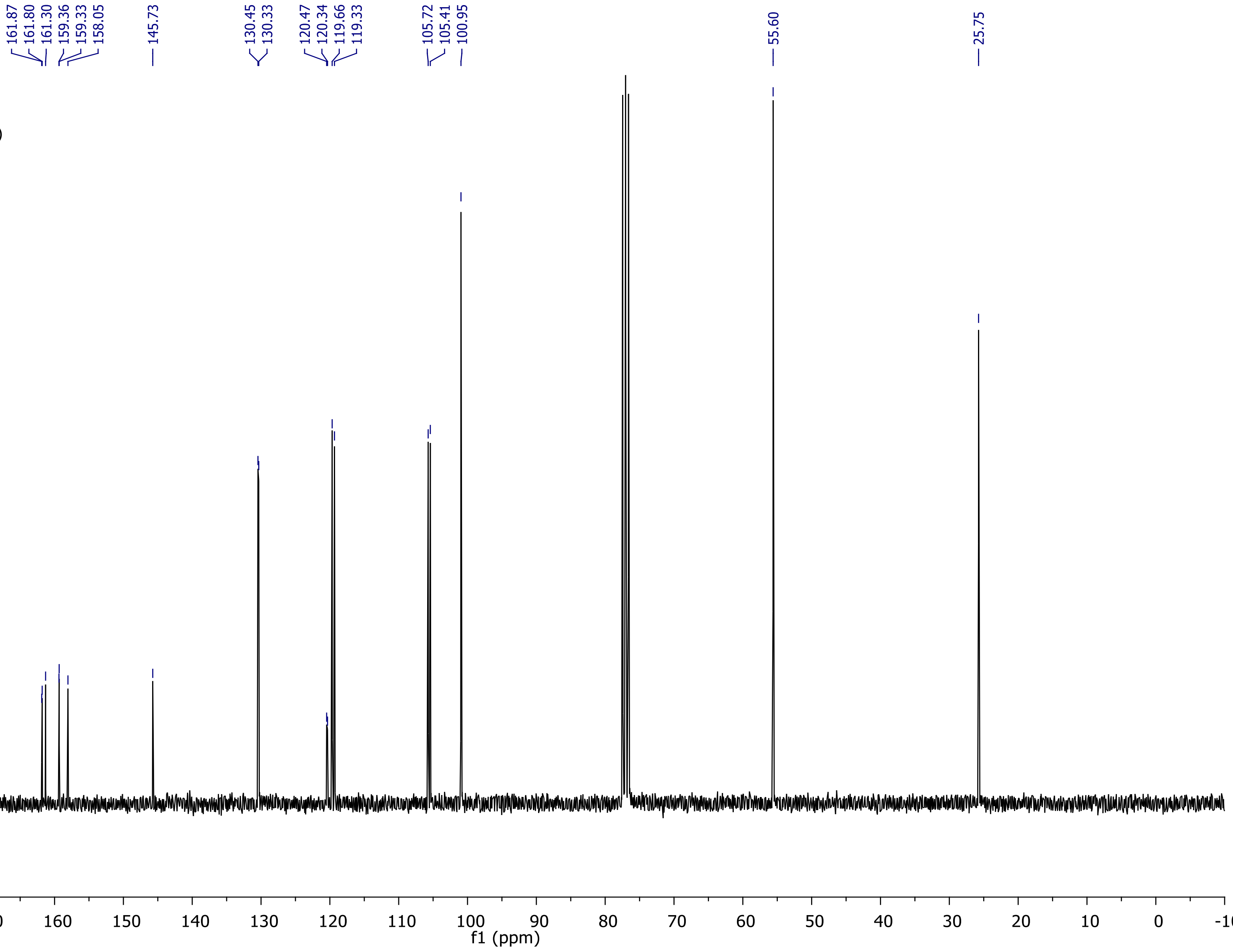

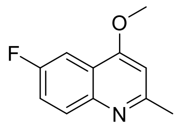

1b

^{19}F NMR (282 MHz, $CDCl_3$)

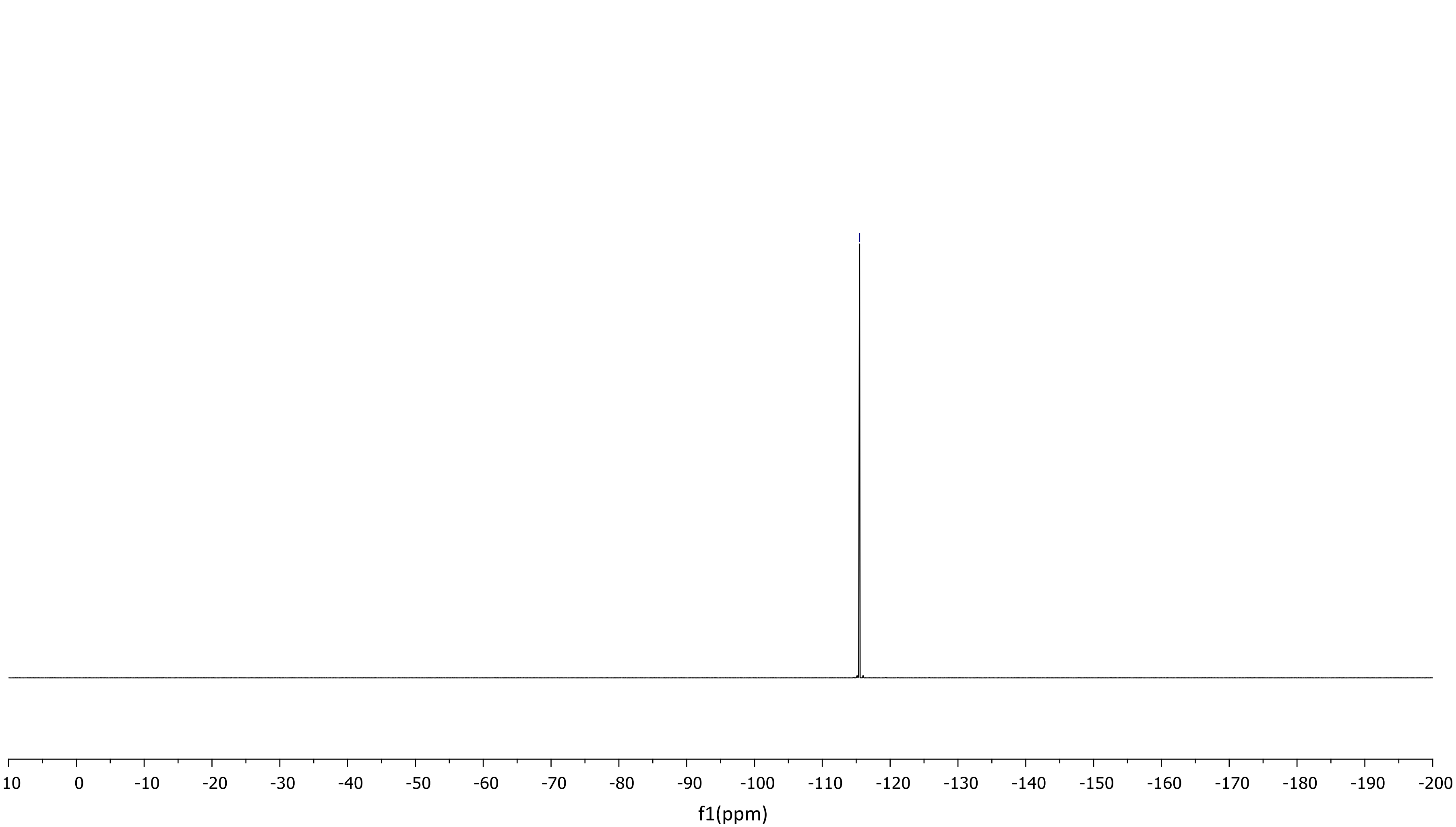

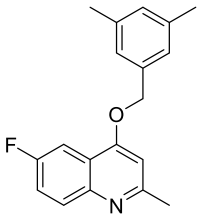

1c

^{1}H NMR (500 MHz, $CDCl_3$)

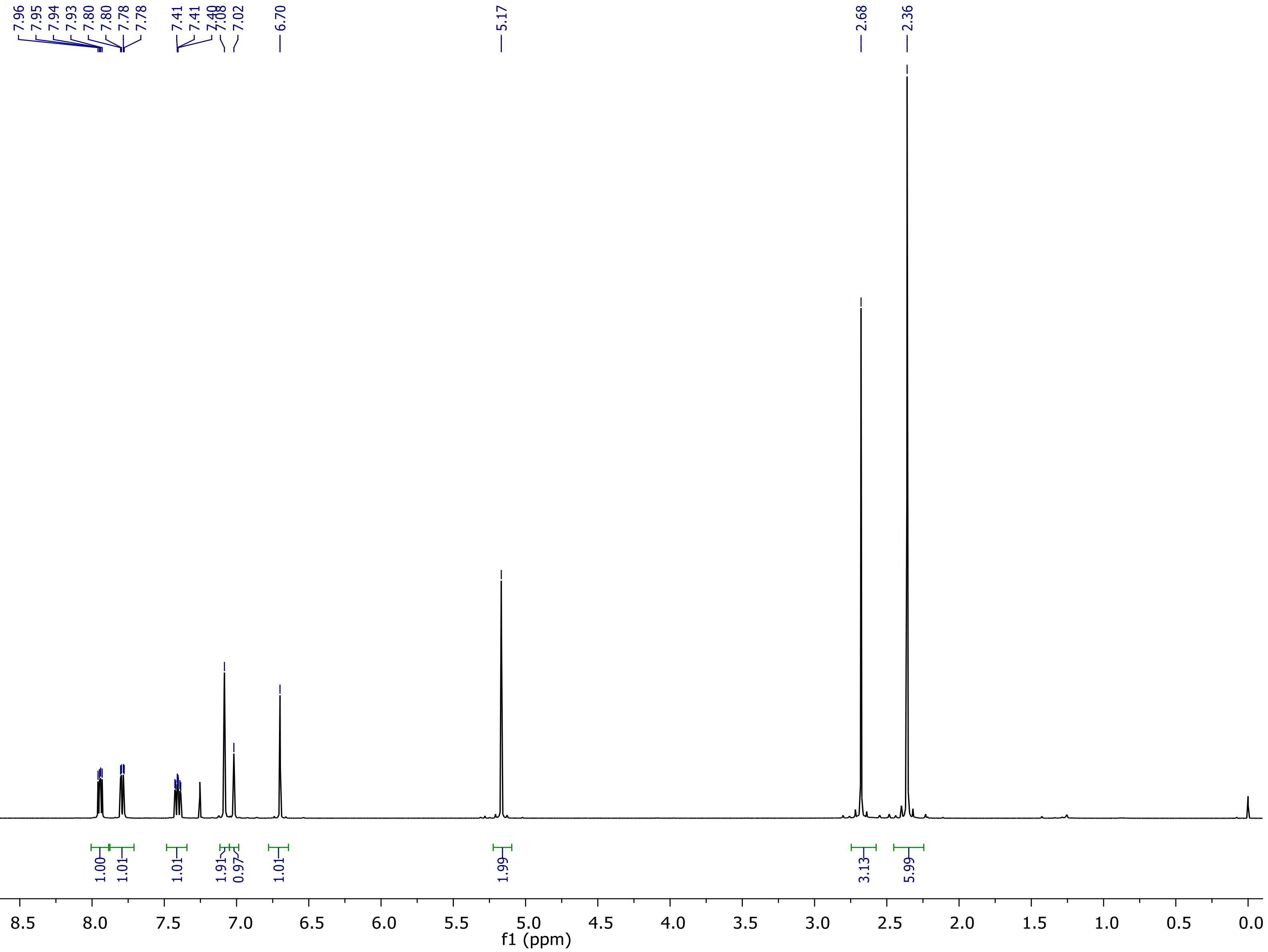

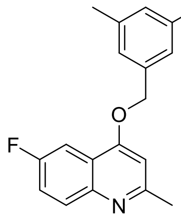

1c

$^{13}C\{^1H\}$ NMR (125 MHz, $CDCl_3$)

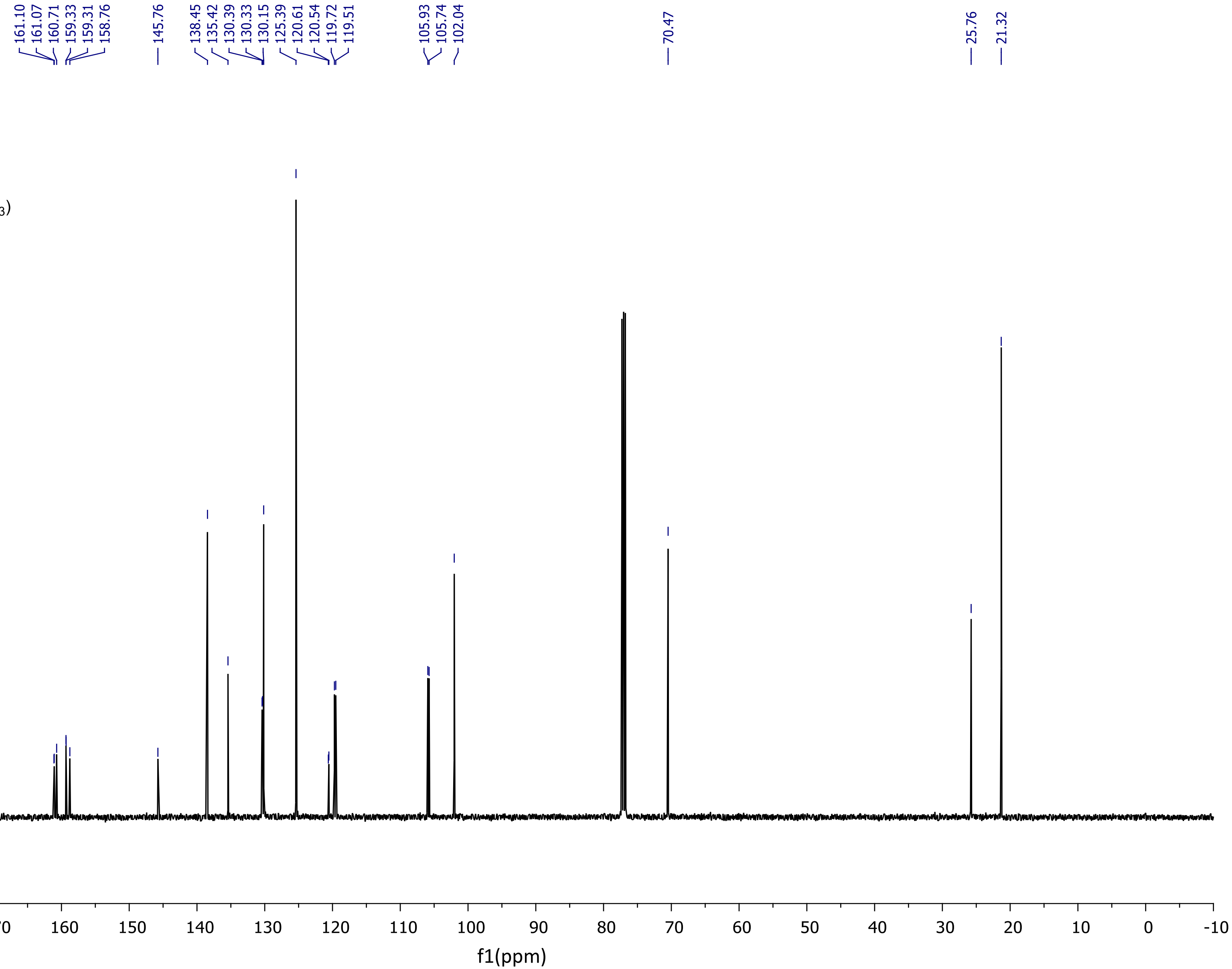

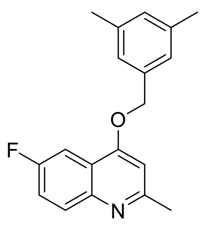

1c

^{19}F NMR (470 MHz, $CDCl_3$)

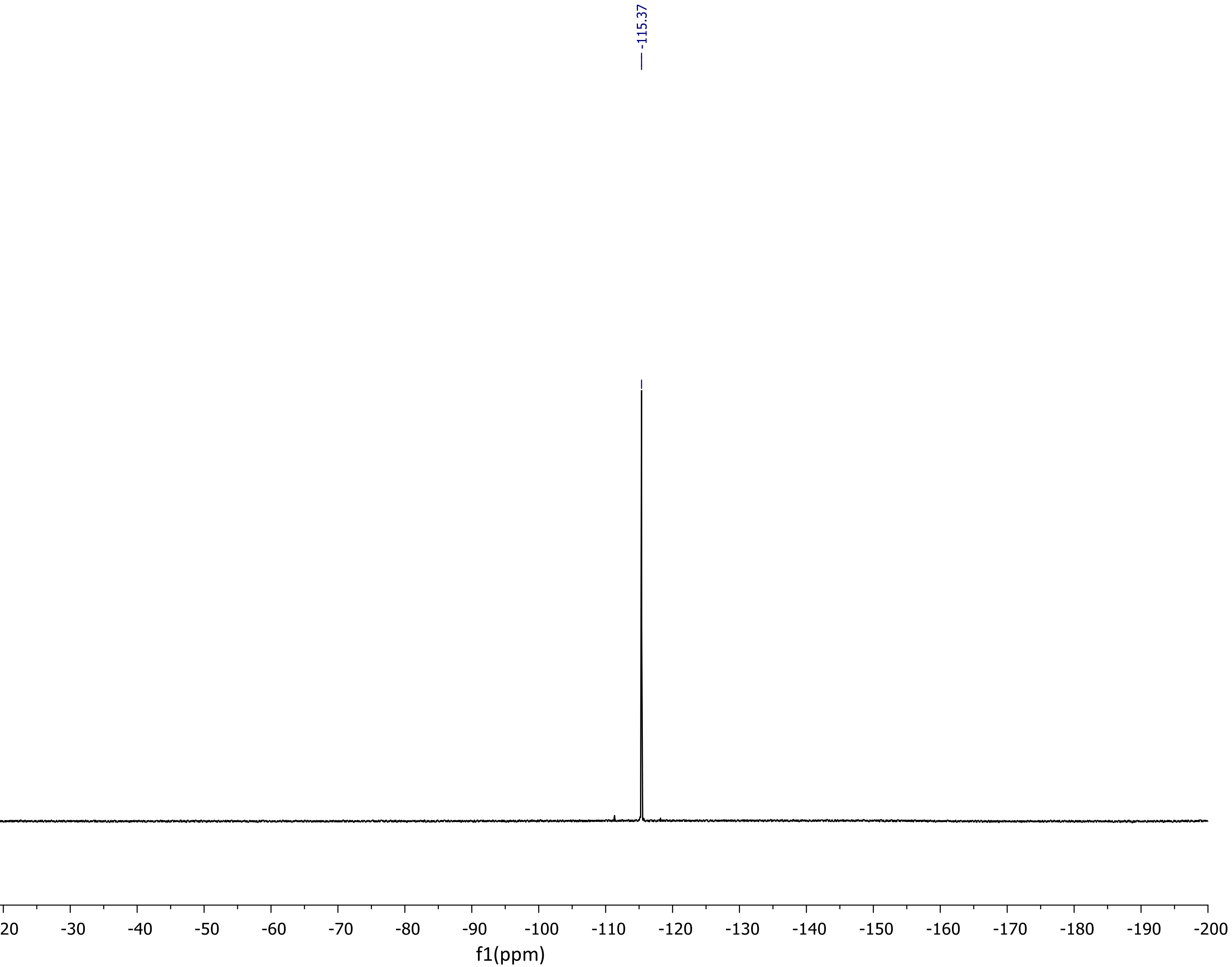

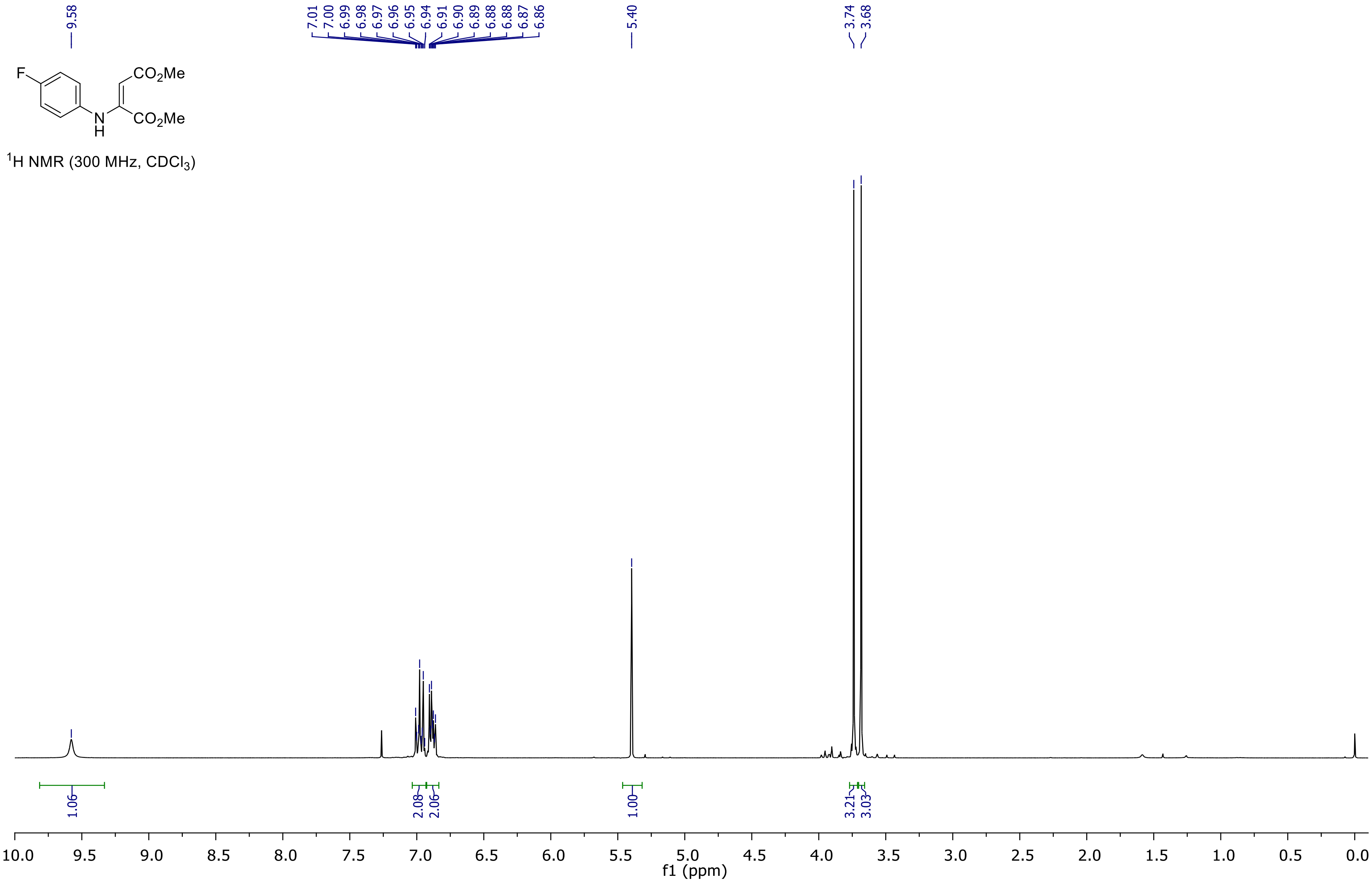

9.58
7.01 7.00 6.99 6.98 6.97 6.96 6.95 6.94 6.91 6.90 6.89 6.88 6.88 6.87 6.86
5.40
3.74 3.68
F
CO2Me
N
H
CO2Me
1H NMR (300 MHz, CDCl3)
1.06
2.08
2.06
1.00
3.21
3.03
10.0 9.5 9.0 8.5 8.0 7.5 7.0 6.5 6.0 5.5 5.0 4.5 4.0 3.5 3.0 2.5 2.0 1.5 1.0 0.5 0.0
f1 (ppm)

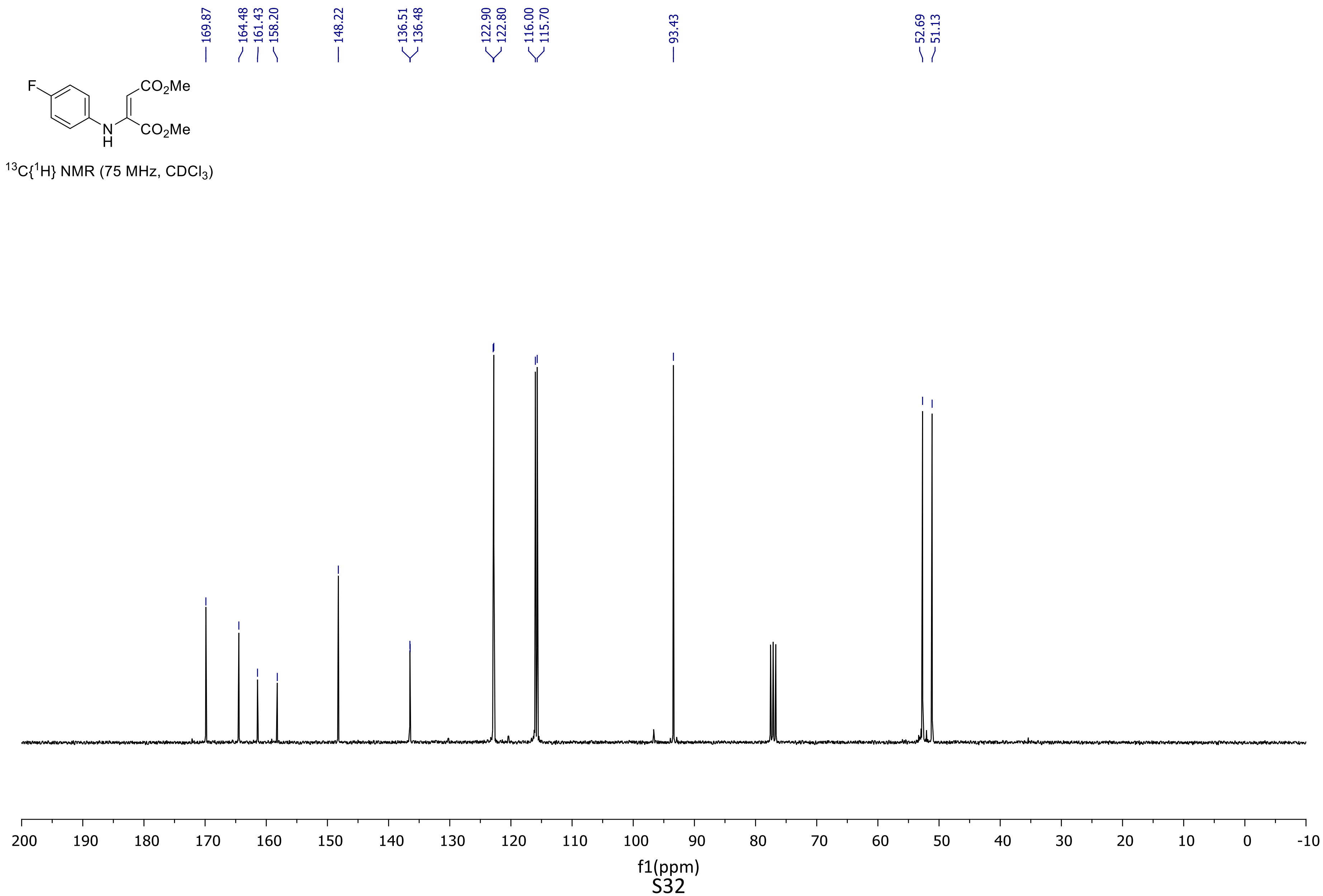
169.87
164.48
161.43
158.20
148.22
136.51
136.48
122.90
122.80
116.00
115.70
93.43
52.69
51.13
F
CO2Me
N
H
CO2Me
13C{1H} NMR (75 MHz, CDCl3)
200
190
180
170
160
150
140
130
120
110
100
90
80
70
60
50
40
30
20
10
0
-10
f1(ppm)

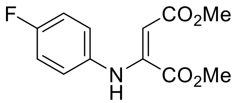

^{19}F NMR (282 MHz, $CDCl_3$)

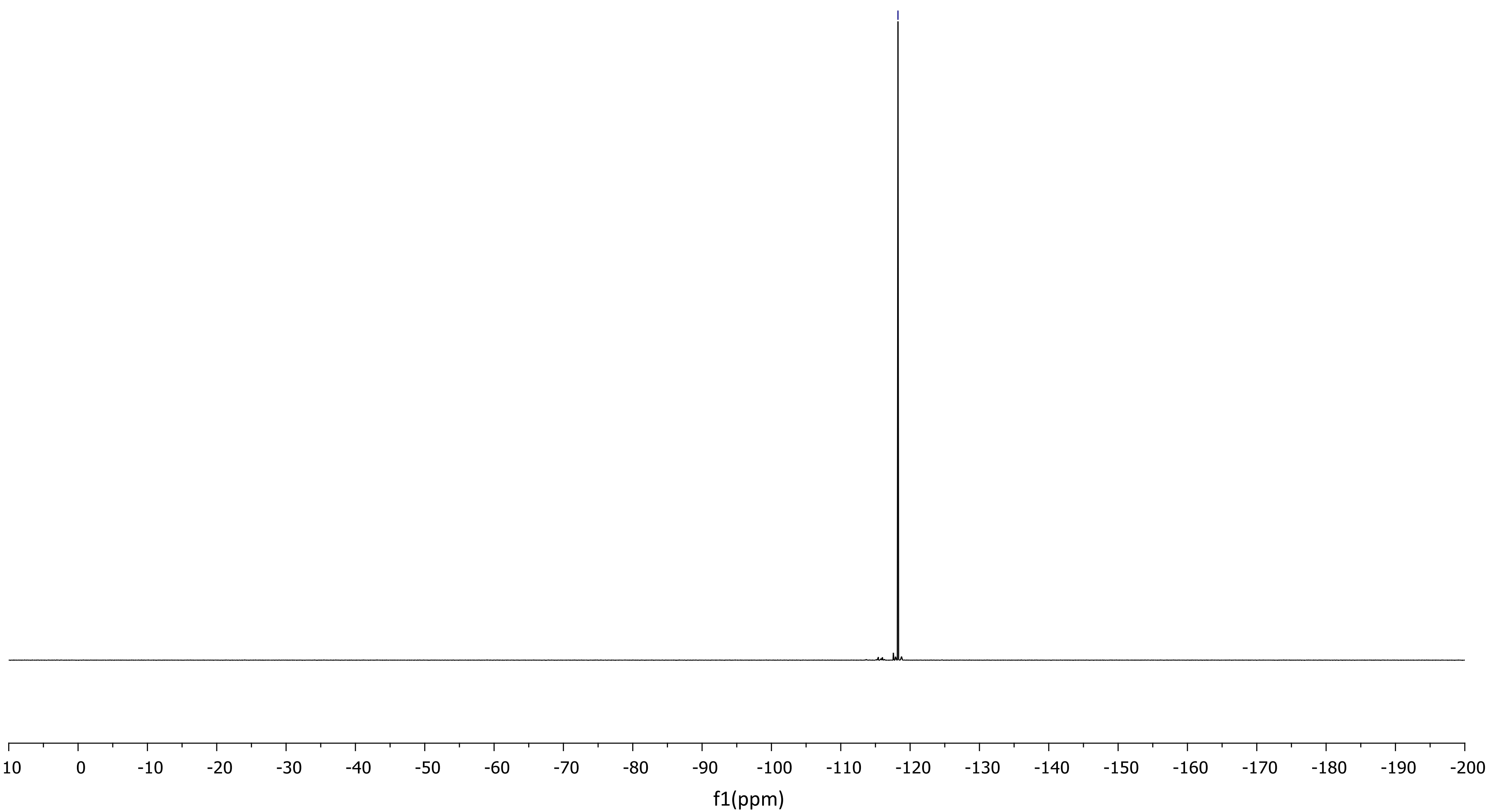

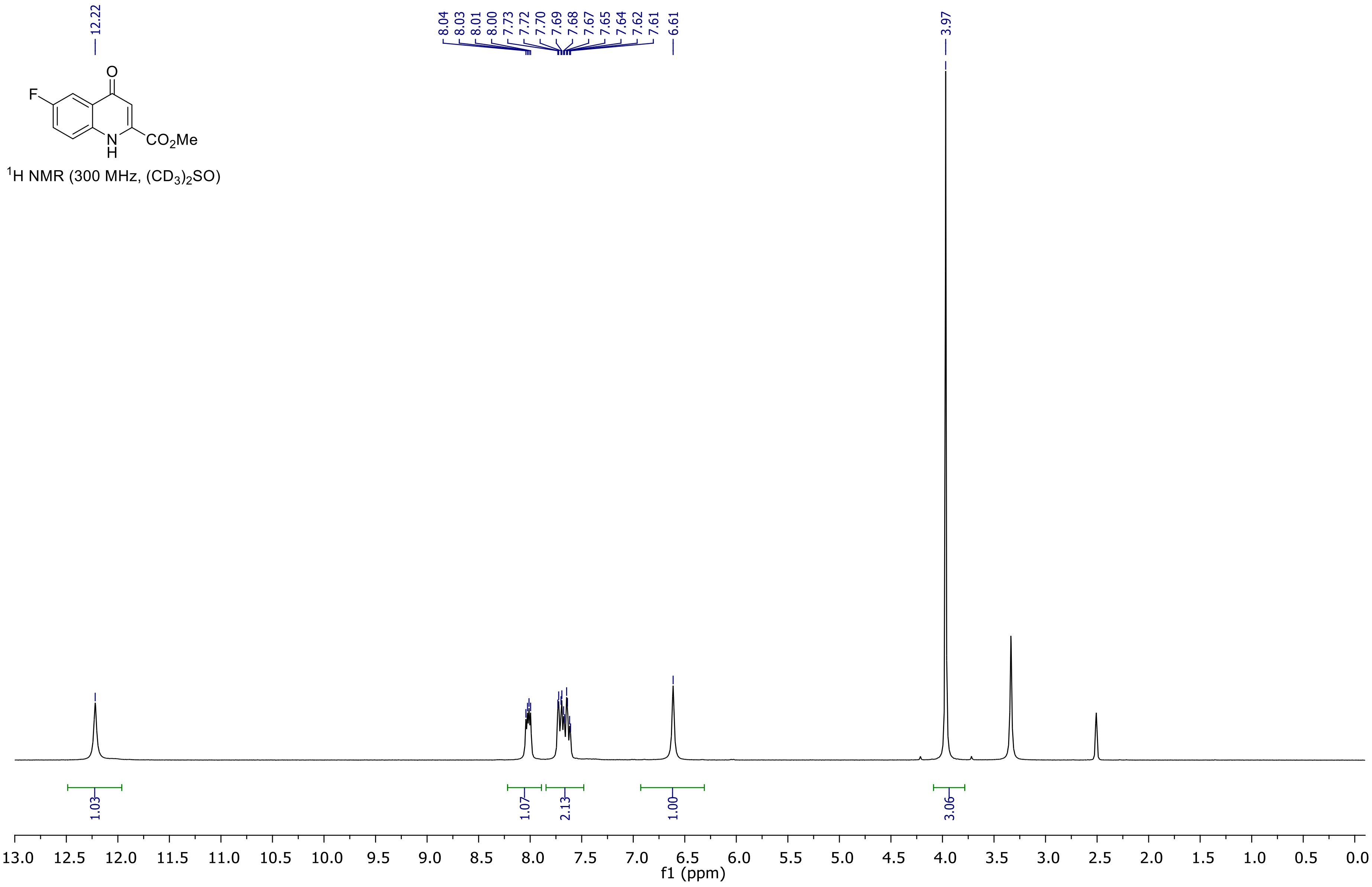
F
O
N
H
CO2Me
1H NMR (300 MHz, (CD3)2SO)
12.22
8.04
8.03
8.01
8.00
7.73
7.72
7.70
7.69
7.68
7.67
7.65
7.64
7.62
7.61
6.61
3.97
1.03
1.07
2.13
1.00
3.06
13.0 12.5 12.0 11.5 11.0 10.5 10.0 9.5 9.0 8.5 8.0 7.5 7.0 6.5 6.0 5.5 5.0 4.5 4.0 3.5 3.0 2.5 2.0 1.5 1.0 0.5 0.0
f1 (ppm)

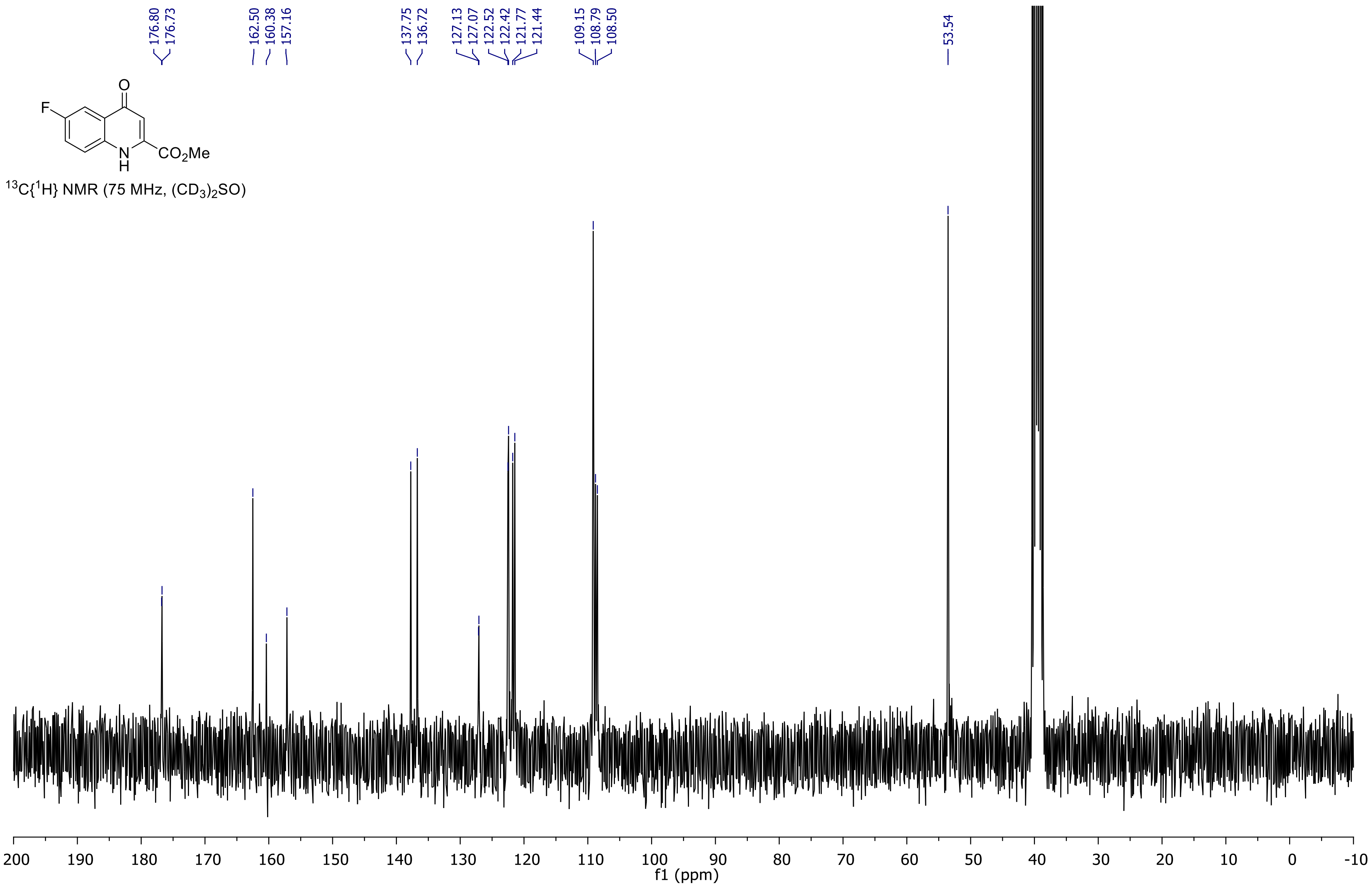
176.80
176.73
162.50
160.38
157.16
137.75
136.72
127.13
127.07
122.52
122.42
121.77
121.44
109.15
108.79
108.50
53.54
F
O
N
H
CO2Me
13C{1H} NMR (75 MHz, (CD3)2SO)
200
190
180
170
160
150
140
130
120
110
100
90
80
70
60
50
40
30
20
10
0
-10
f1 (ppm)

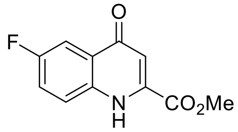

^{19}F NMR (282 MHz, $(CD_3)_2SO$)

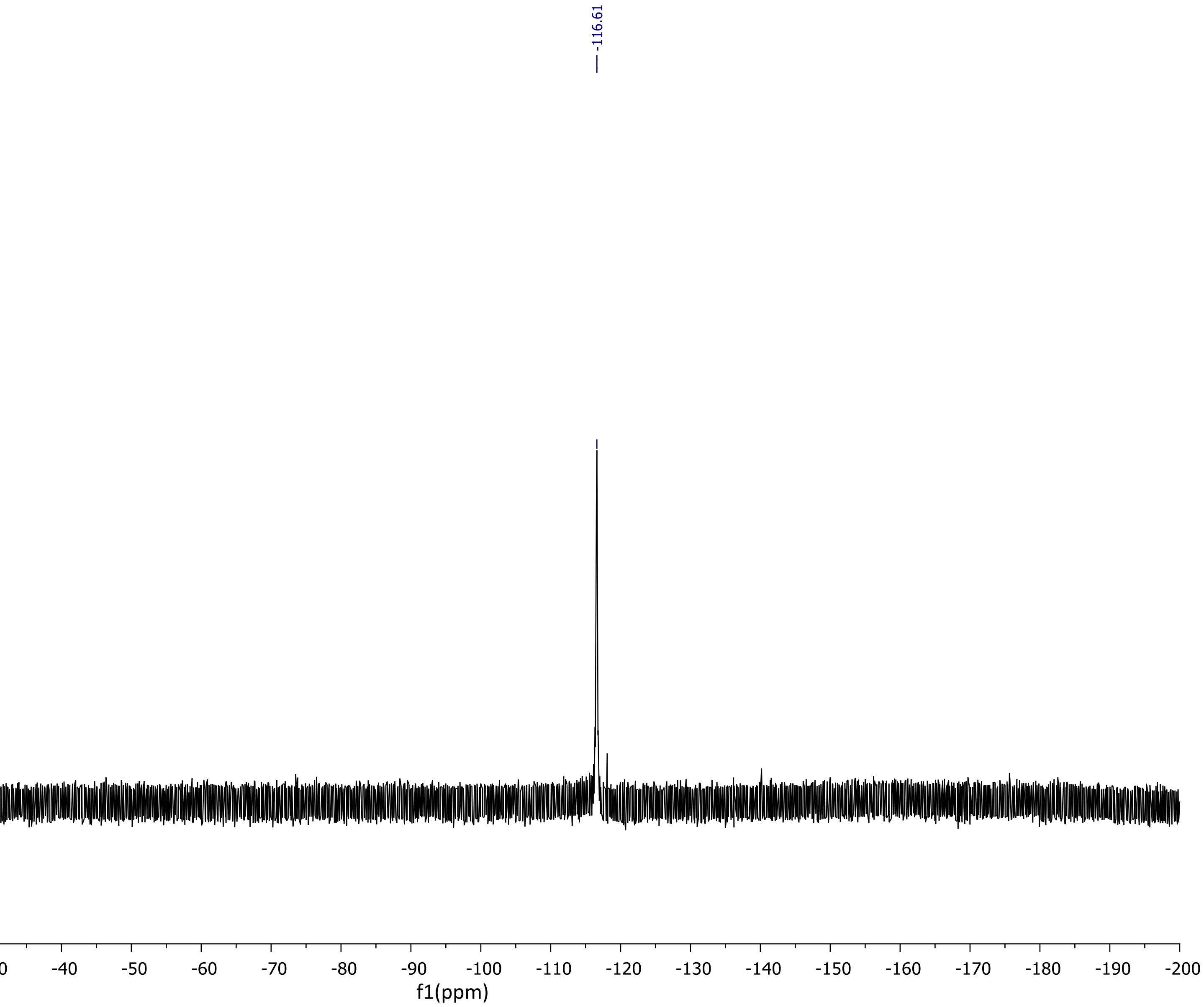

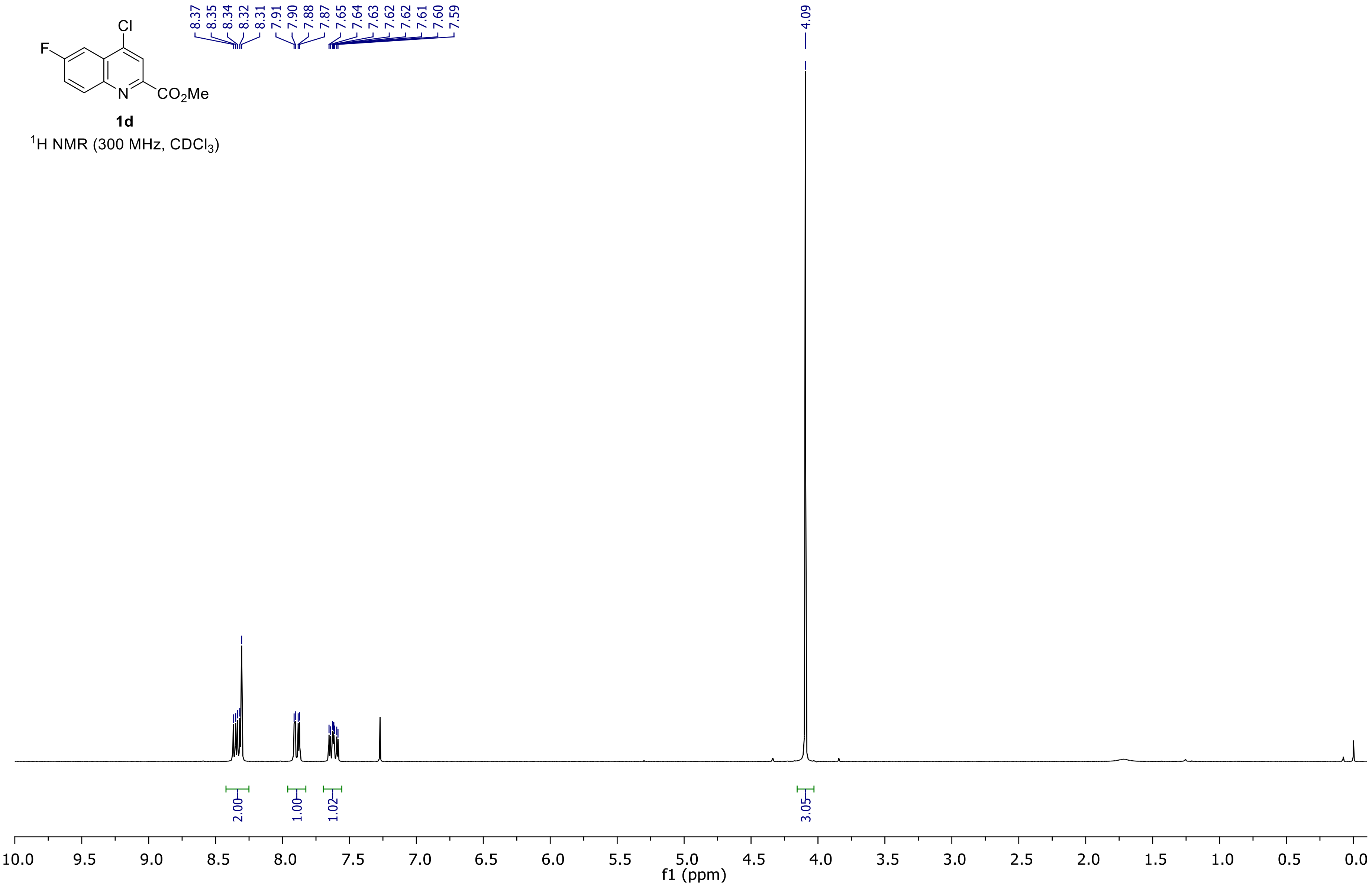
Cl
F
N
CO2Me
1d
1H NMR (300 MHz, CDCl3)
8.37 8.35 8.34 8.32 8.31 7.91 7.90 7.88 7.87 7.65 7.64 7.63 7.62 7.62 7.61 7.60 7.59
4.09
2.00
1.00
1.02
3.05
10.0 9.5 9.0 8.5 8.0 7.5 7.0 6.5 6.0 5.5 5.0 4.5 4.0 3.5 3.0 2.5 2.0 1.5 1.0 0.5 0.0
f1 (ppm)

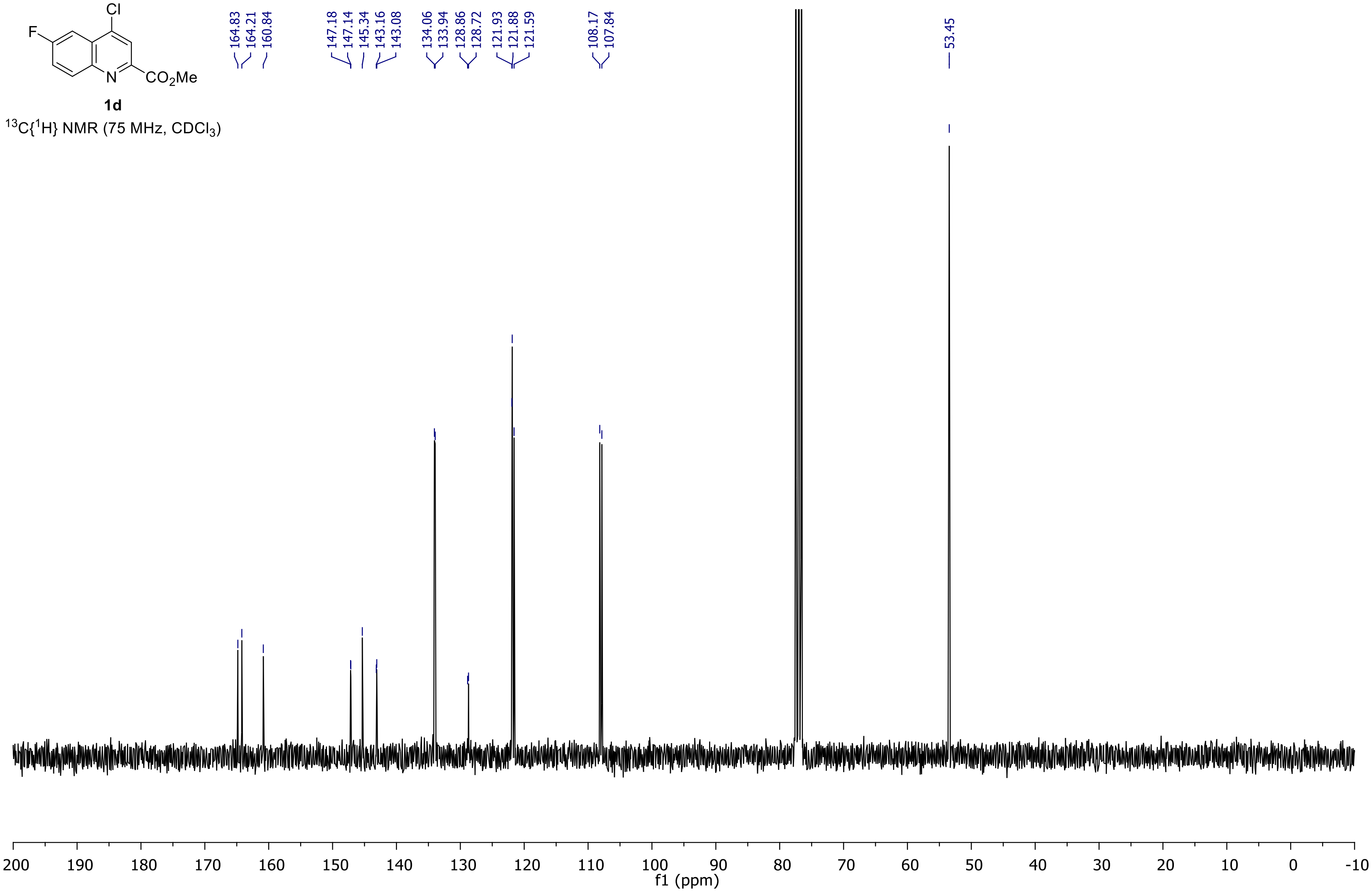

1d

$^{13}C\{^1H\}$ NMR (75 MHz, $CDCl_3$)

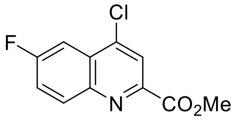

1d

^{19}F NMR (282 MHz, $CDCl_3$)

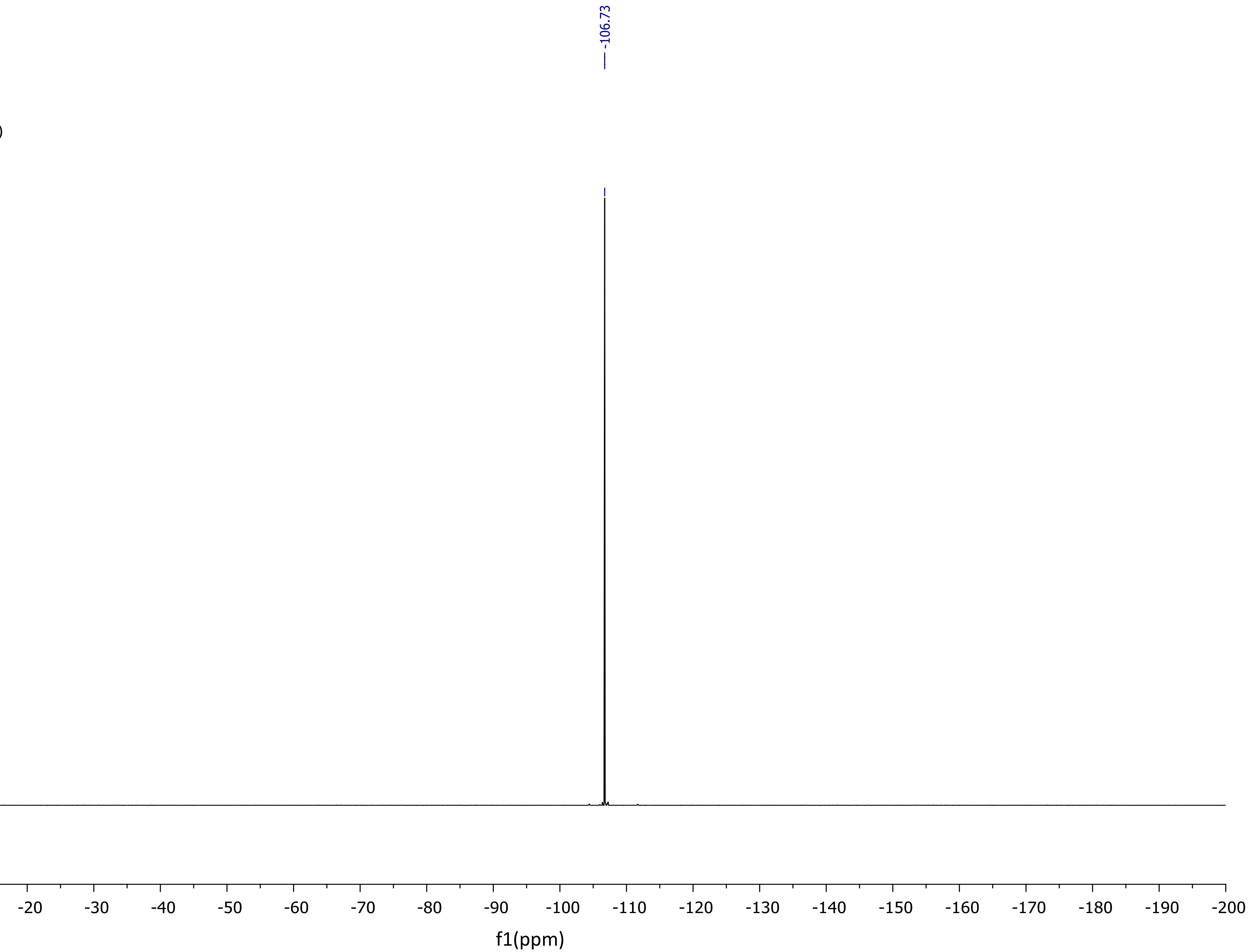

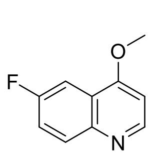

1e

^{1}H NMR (500 MHz, $CDCl_3$)

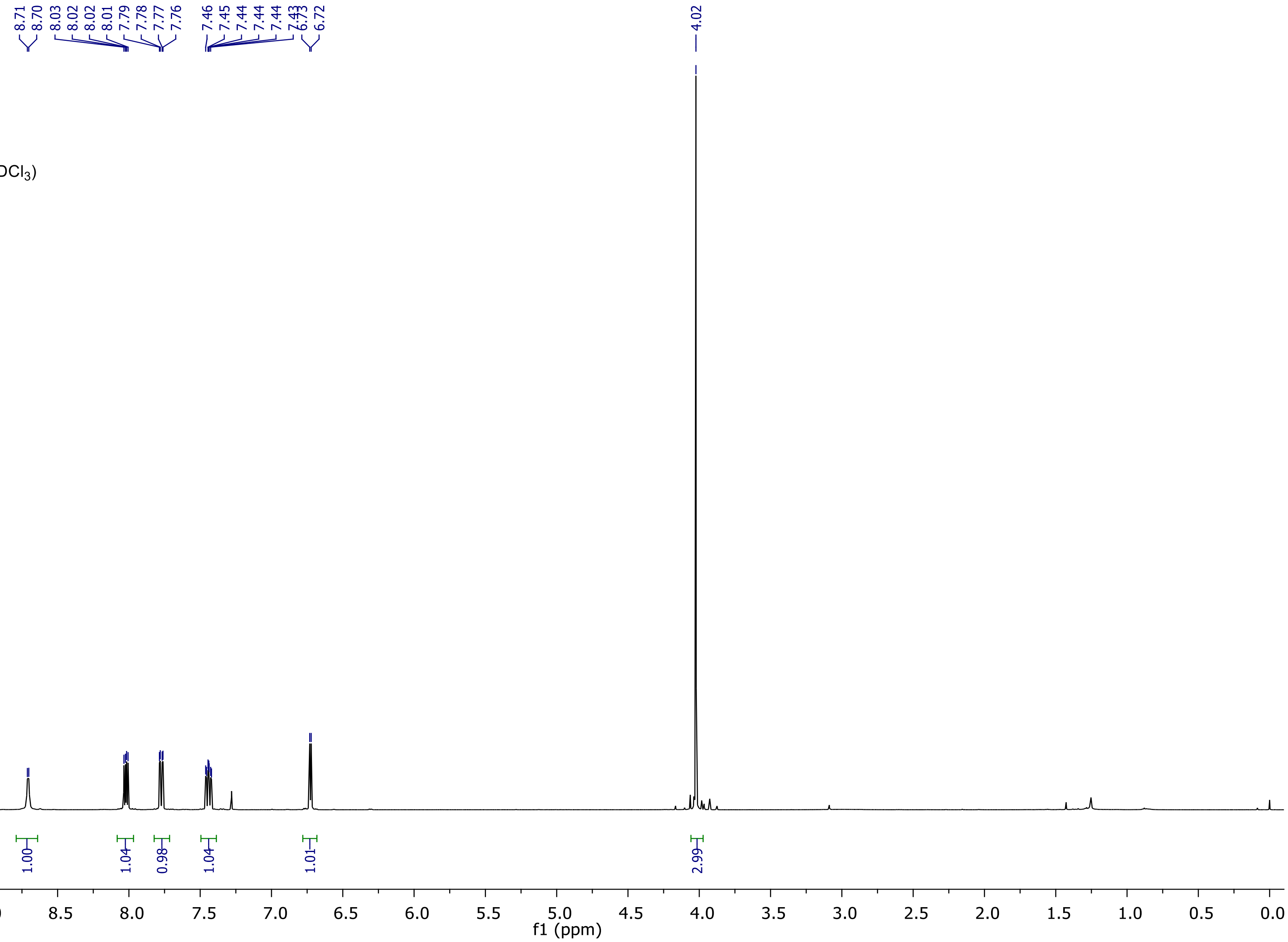

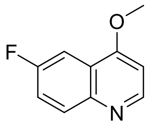

1e

$^{13}C\{^{1}H\}$ NMR (125MHz, $CDCl_3$)

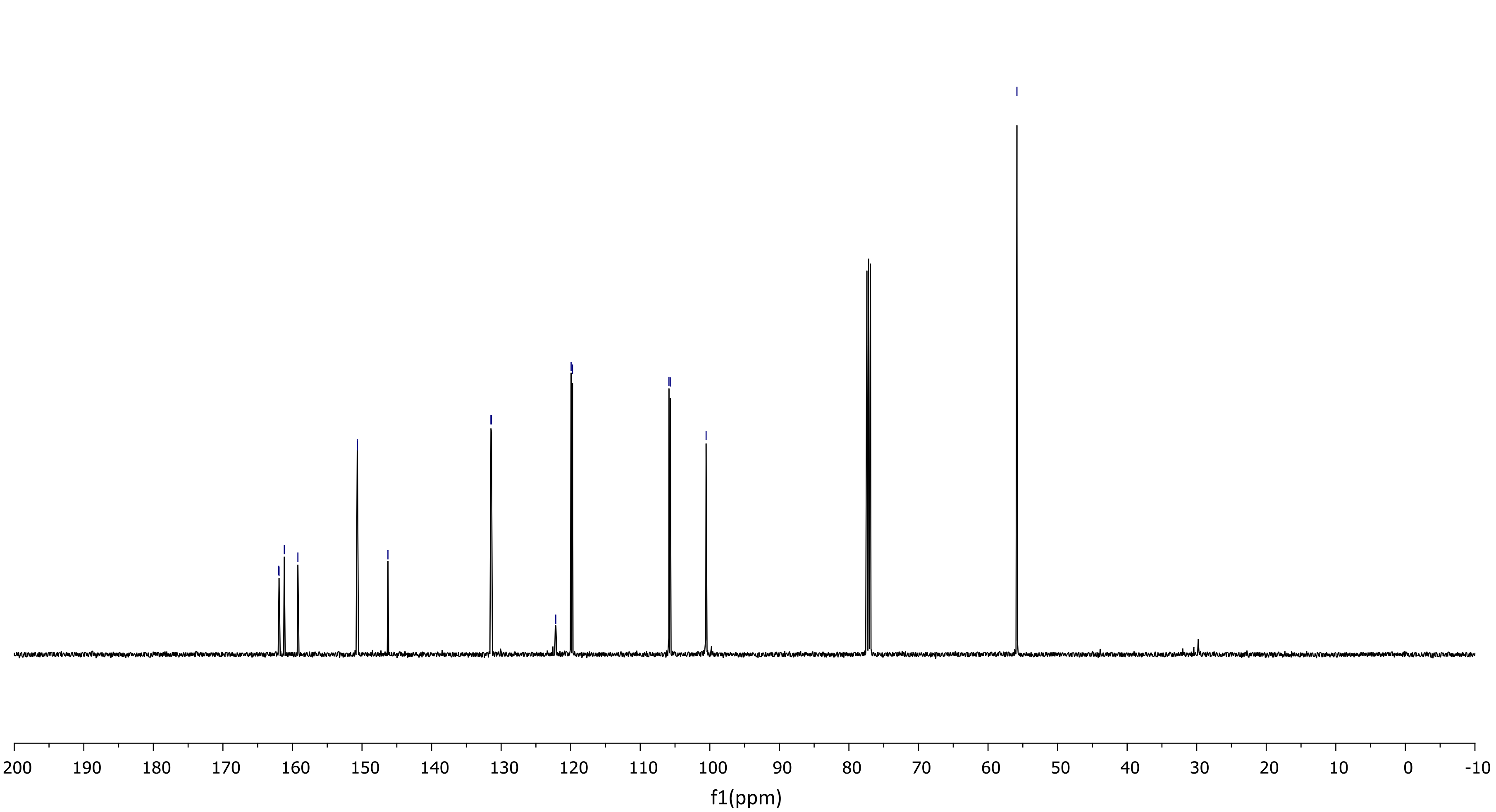

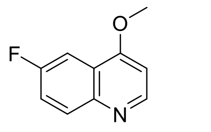

1e

^{19}F NMR (282 MHz, $CDCl_3$)

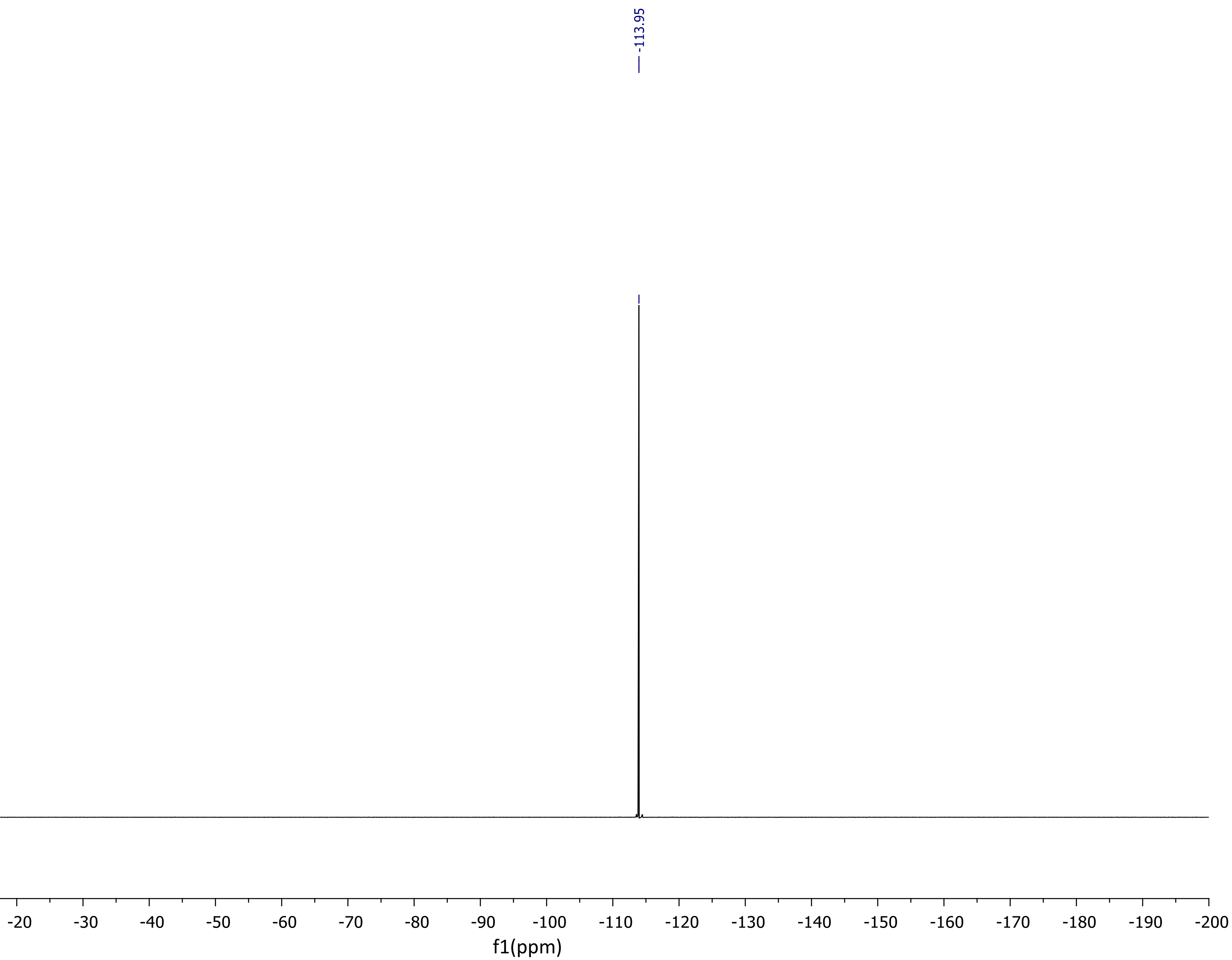

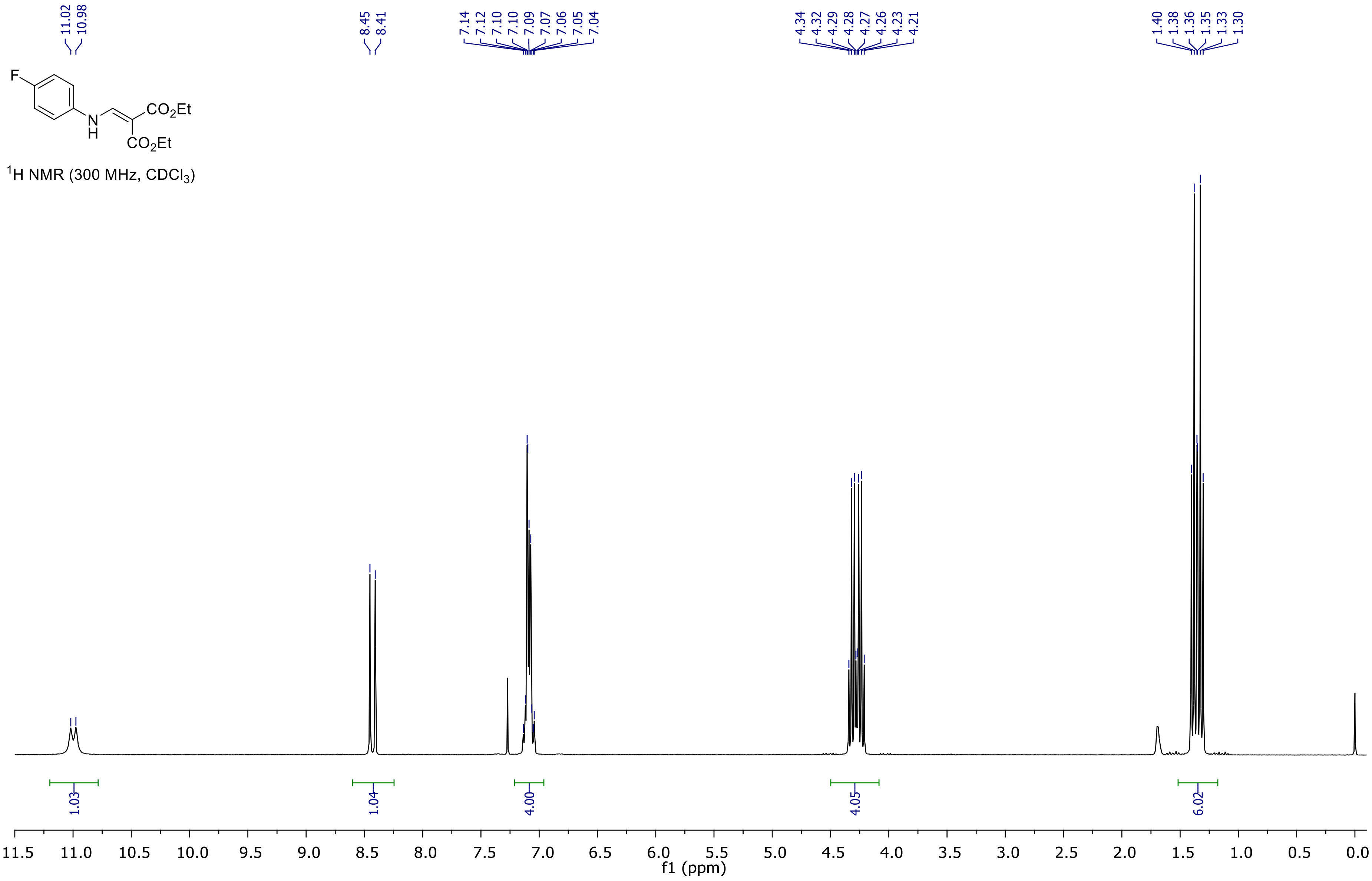
F
N
H
CO2Et
CO2Et
1H NMR (300 MHz, CDCl3)
11.02
10.98
8.45
8.41
7.14
7.12
7.10
7.10
7.09
7.07
7.06
7.05
7.04
4.34
4.32
4.29
4.28
4.27
4.26
4.23
4.21
1.40
1.38
1.36
1.35
1.33
1.30
1.03
1.04
4.00
4.05
6.02
11.5 11.0 10.5 10.0 9.5 9.0 8.5 8.0 7.5 7.0 6.5 6.0 5.5 5.0 4.5 4.0 3.5 3.0 2.5 2.0 1.5 1.0 0.5 0.0
f1 (ppm)

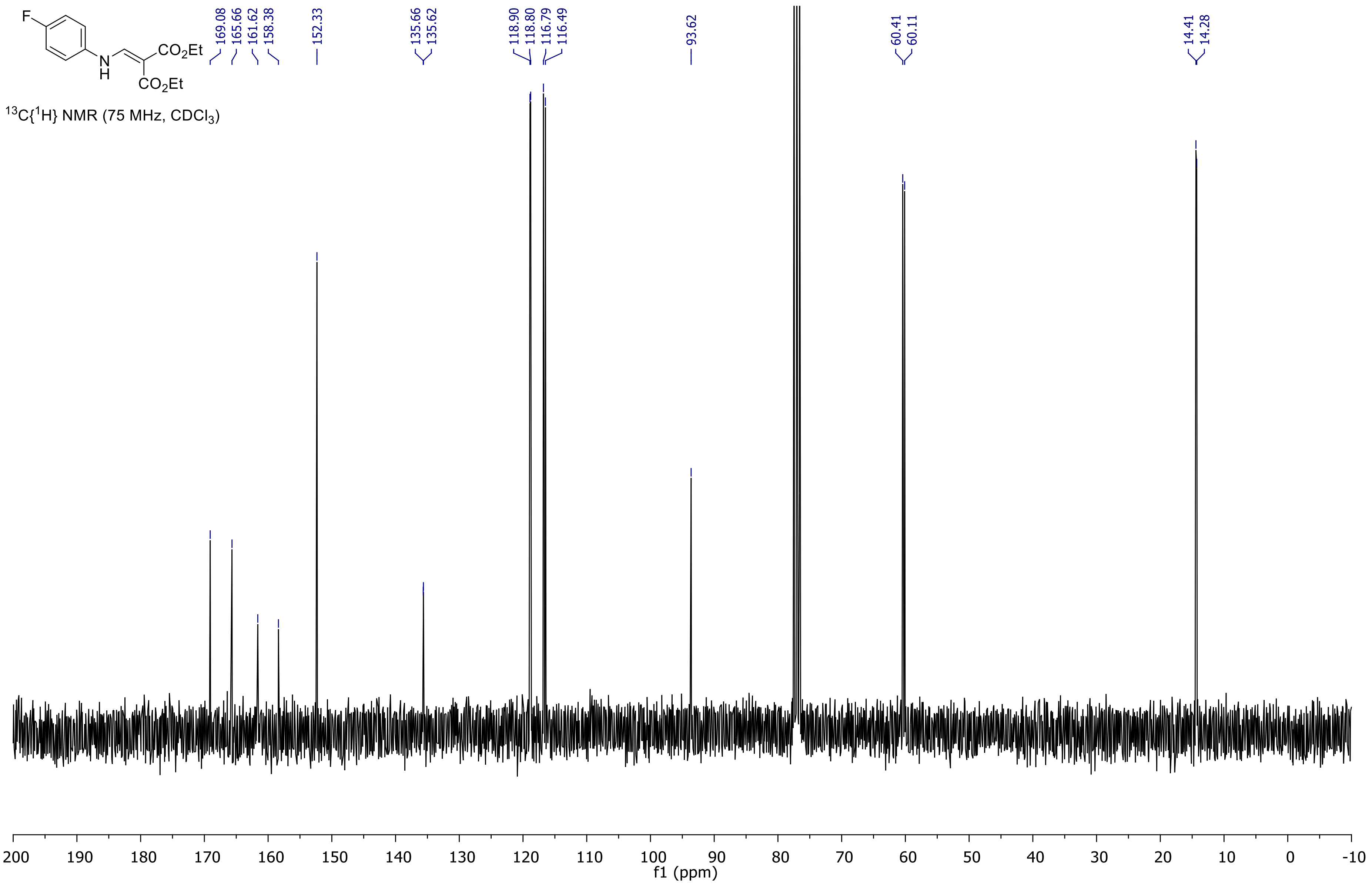
F
N
H
CO2Et
CO2Et
13C{1H} NMR (75 MHz, CDCl3)
169.08
165.66
161.62
158.38
152.33
135.66
135.62
118.90
118.80
116.79
116.49
93.62
60.41
60.11
14.41
14.28
200 190 180 170 160 150 140 130 120 110 100 90 80 70 60 50 40 30 20 10 0 -10
f1 (ppm)

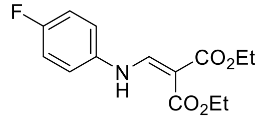

^{19}F NMR (282 MHz, $CDCl_3$)

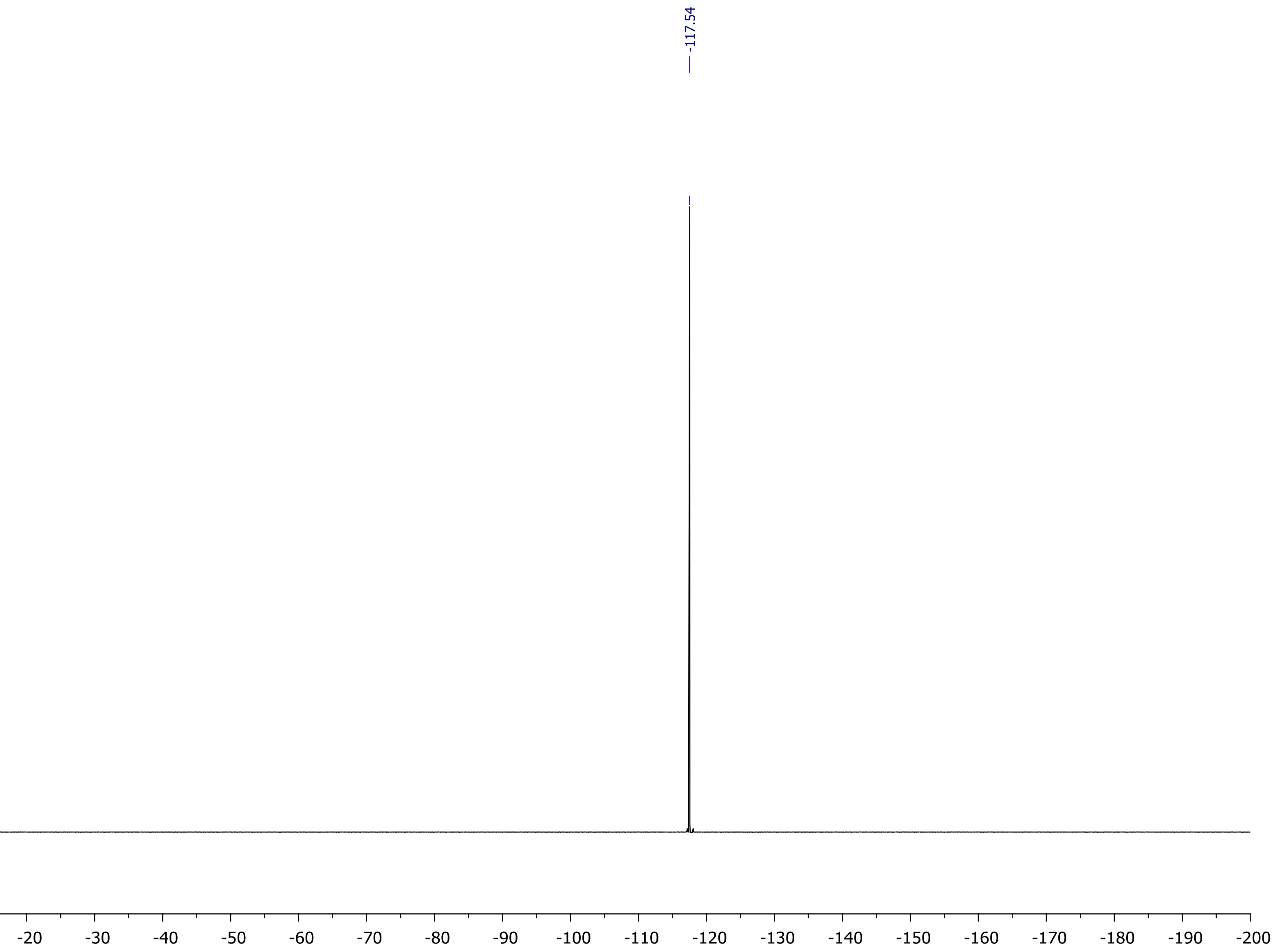

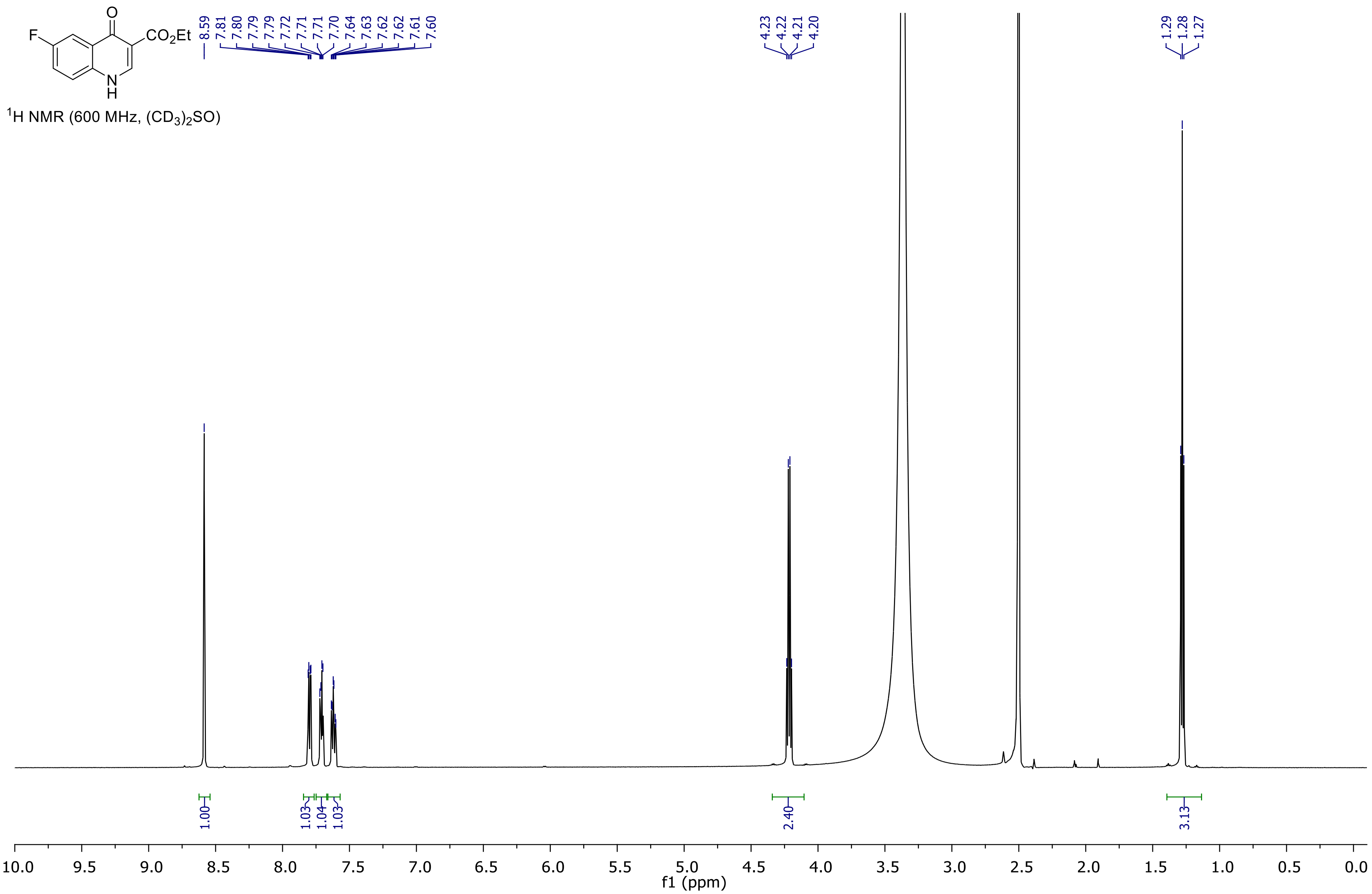

^{1}H NMR (600 MHz, $(CD_3)_2SO$)
CO2Et
F
O
N
H
8.59
7.81
7.80
7.79
7.79
7.72
7.71
7.71
7.70
7.64
7.63
7.62
7.62
7.61
7.60
4.23
4.22
4.21
4.20
1.29
1.28
1.27
1.00
1.03
1.04
1.03
2.40
3.13
10.0
9.5
9.0
8.5
8.0
7.5
7.0
6.5
6.0
5.5
5.0
4.5
4.0
3.5
3.0
2.5
2.0
1.5
1.0
0.5
0.0
f1 (ppm)

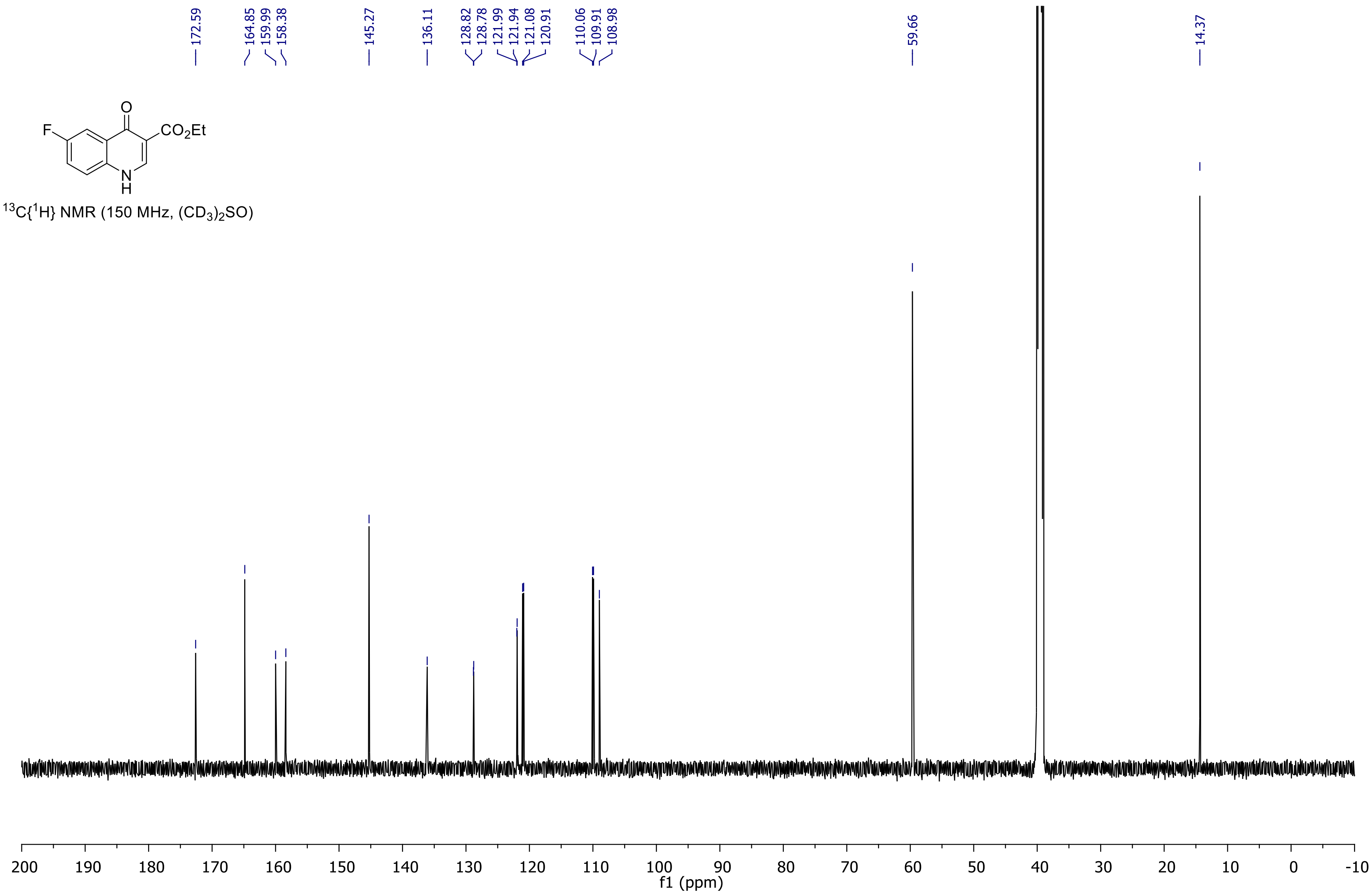

172.59
164.85
159.99
158.38
145.27
136.11
128.82
128.78
121.99
121.94
121.08
120.91
110.06
109.91
108.98
59.66
14.37
F
O
CO2Et
N
H
13C{1H} NMR (150 MHz, (CD3)2SO)
200
190
180
170
160
150
140
130
120
110
100
90
80
70
60
50
40
30
20
10
0
-10
f1 (ppm)

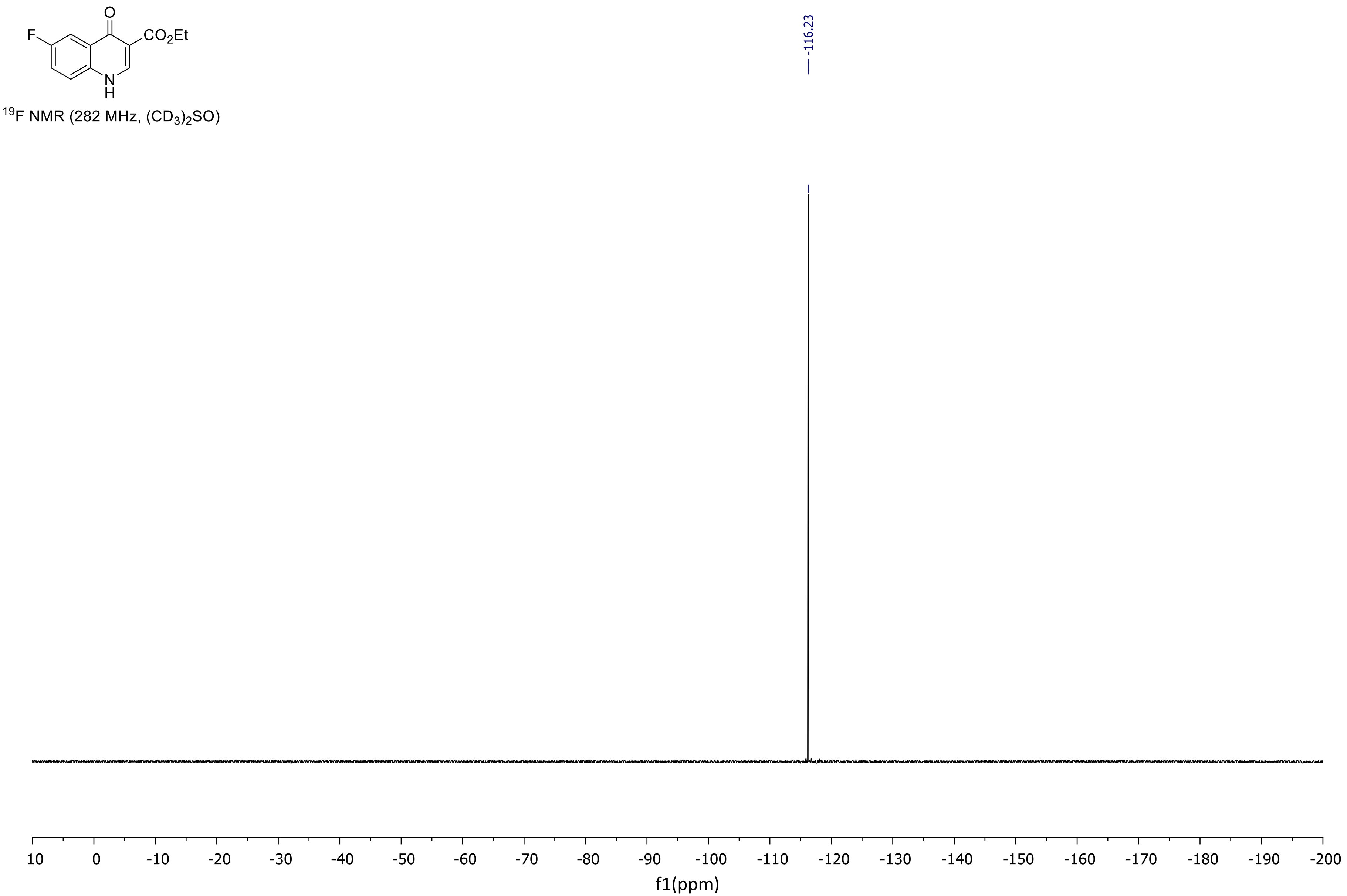

^{19}F NMR (282 MHz, $(CD_3)_2SO$)

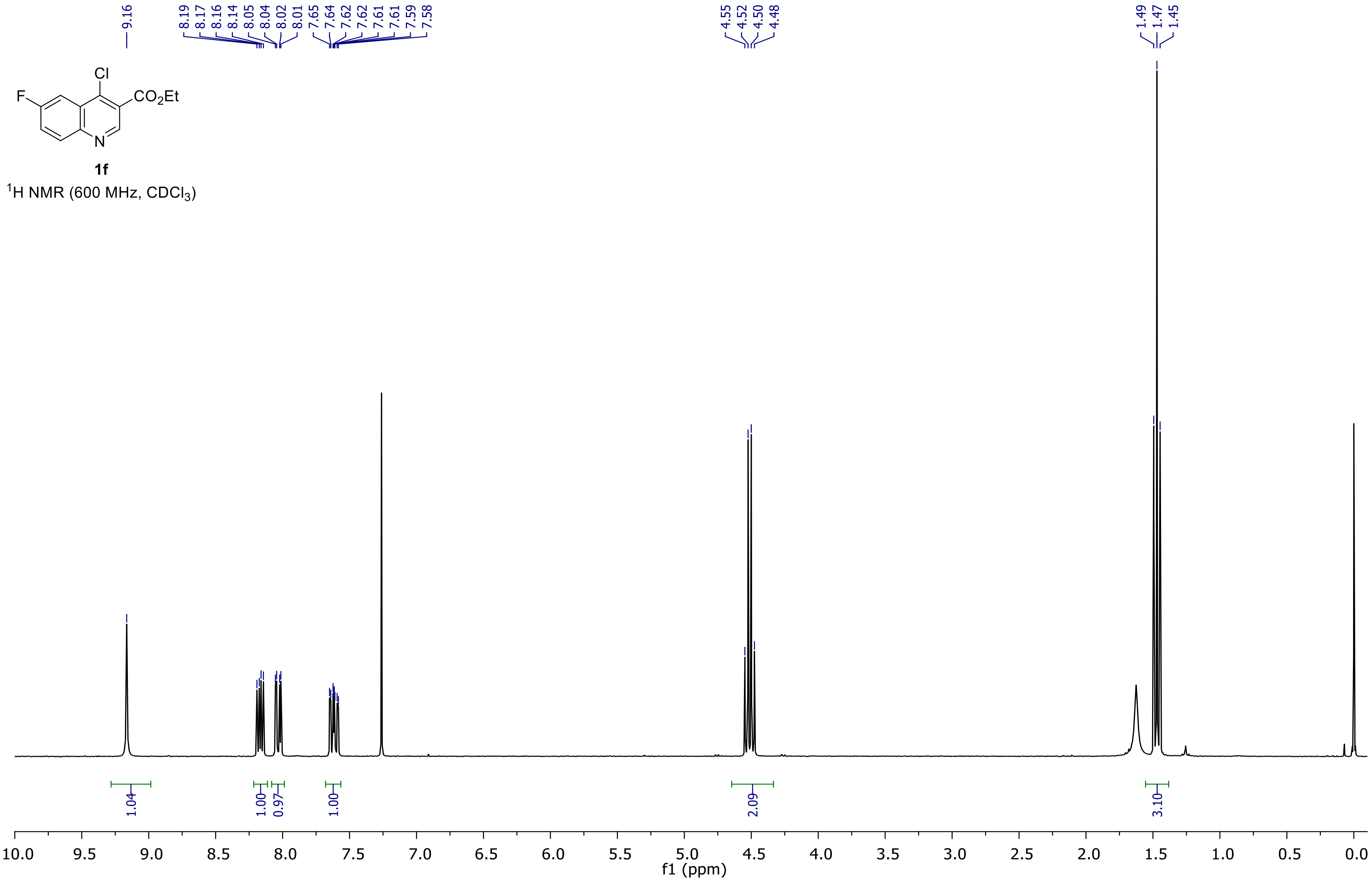

9.16
8.19
8.17
8.16
8.14
8.05
8.04
8.02
8.01
7.65
7.64
7.62
7.62
7.61
7.61
7.59
7.58
4.55
4.52
4.50
4.48
1.49
1.47
1.45
Cl
F
CO2Et
N
1f
1H NMR (600 MHz, CDCl3)
1.04
1.00
0.97
1.00
2.09
3.10
10.0
9.5
9.0
8.5
8.0
7.5
7.0
6.5
6.0
5.5
5.0
4.5
4.0
3.5
3.0
2.5
2.0
1.5
1.0
0.5
0.0
f1 (ppm)

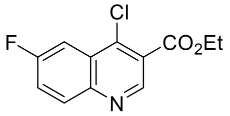

1f

$^{13}C\{^{1}H\}$ NMR (150 MHz, $CDCl_3$)

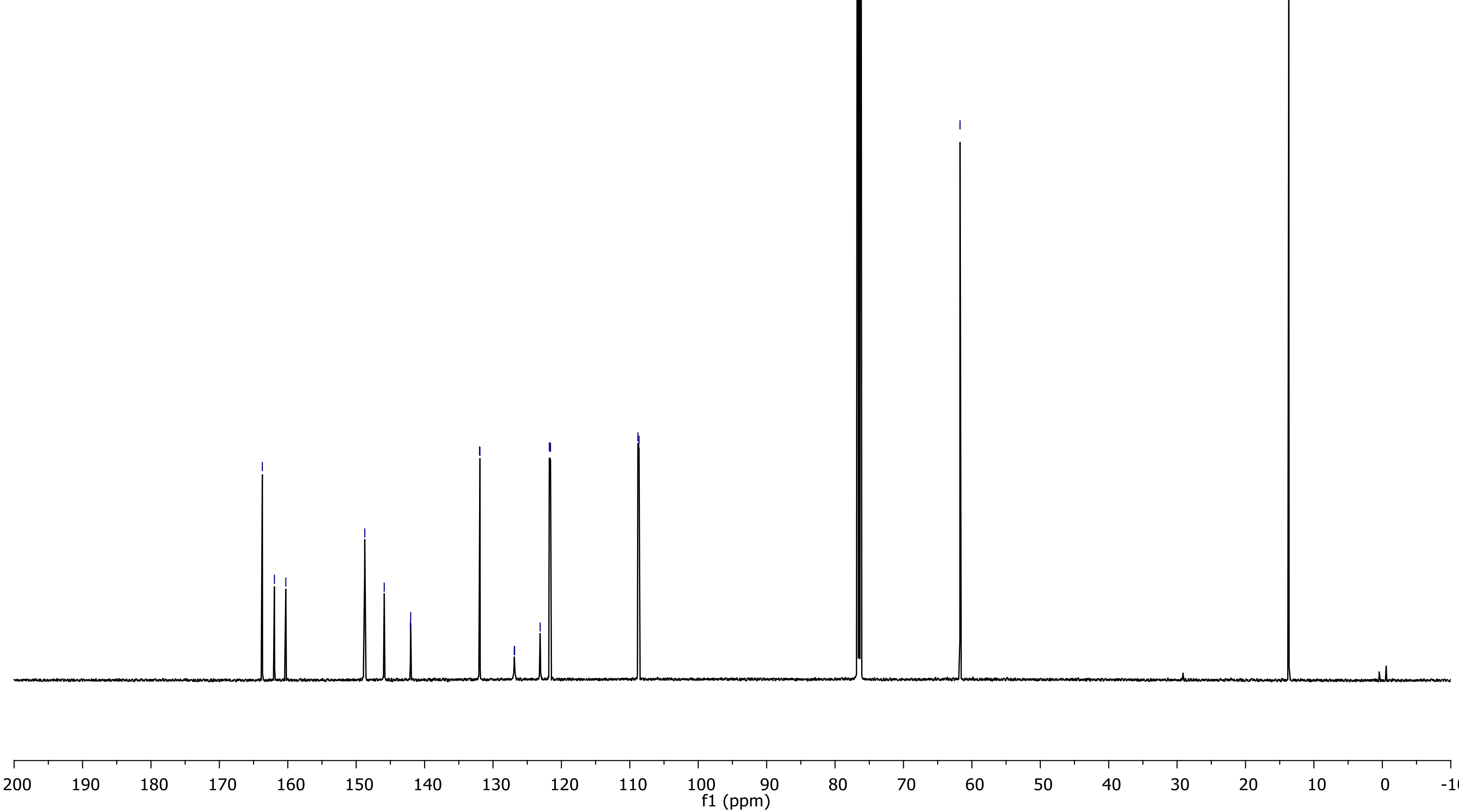

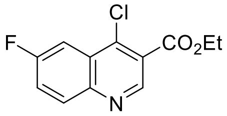

1f

^{19}F NMR (282 MHz, $CDCl_3$)

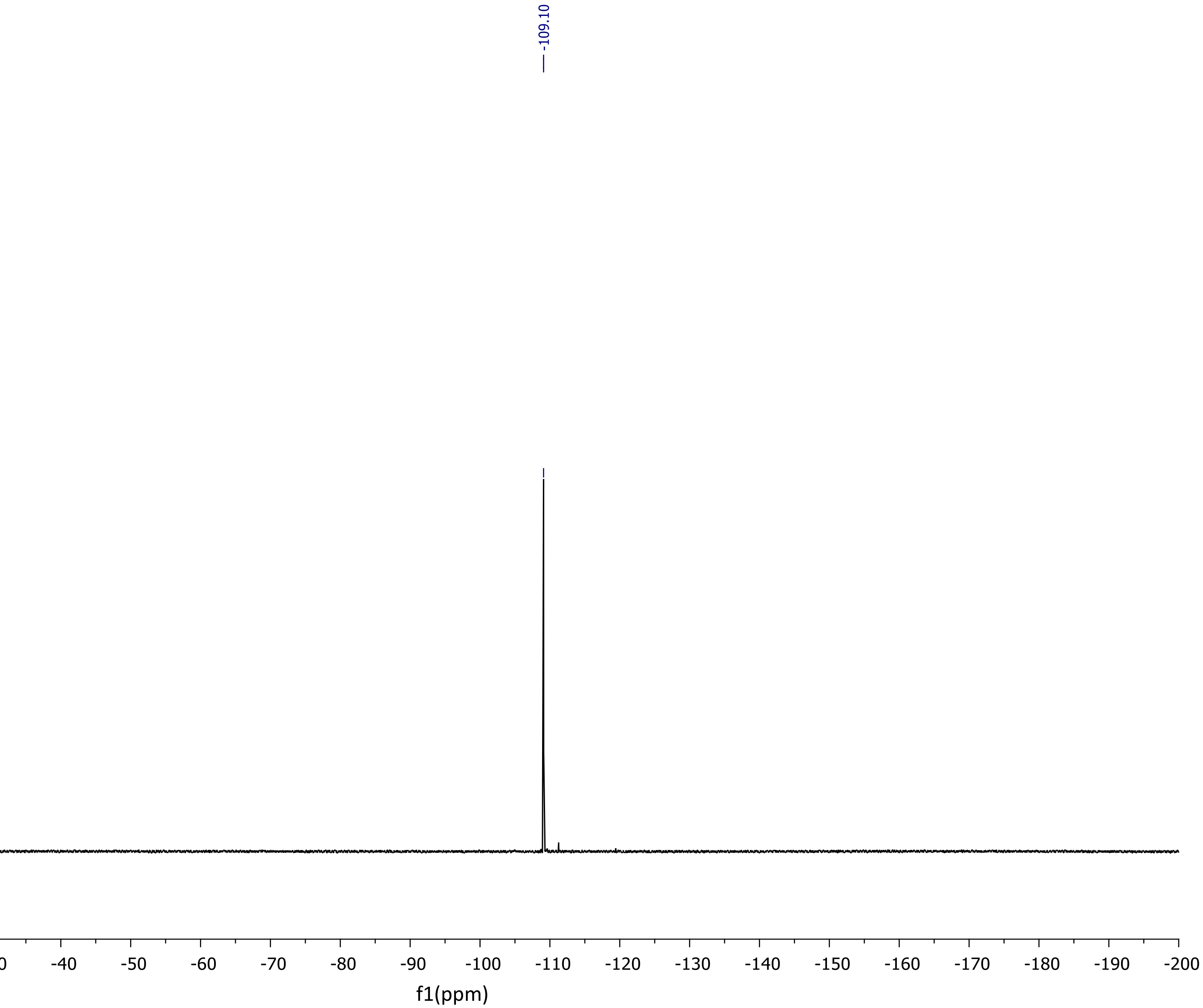

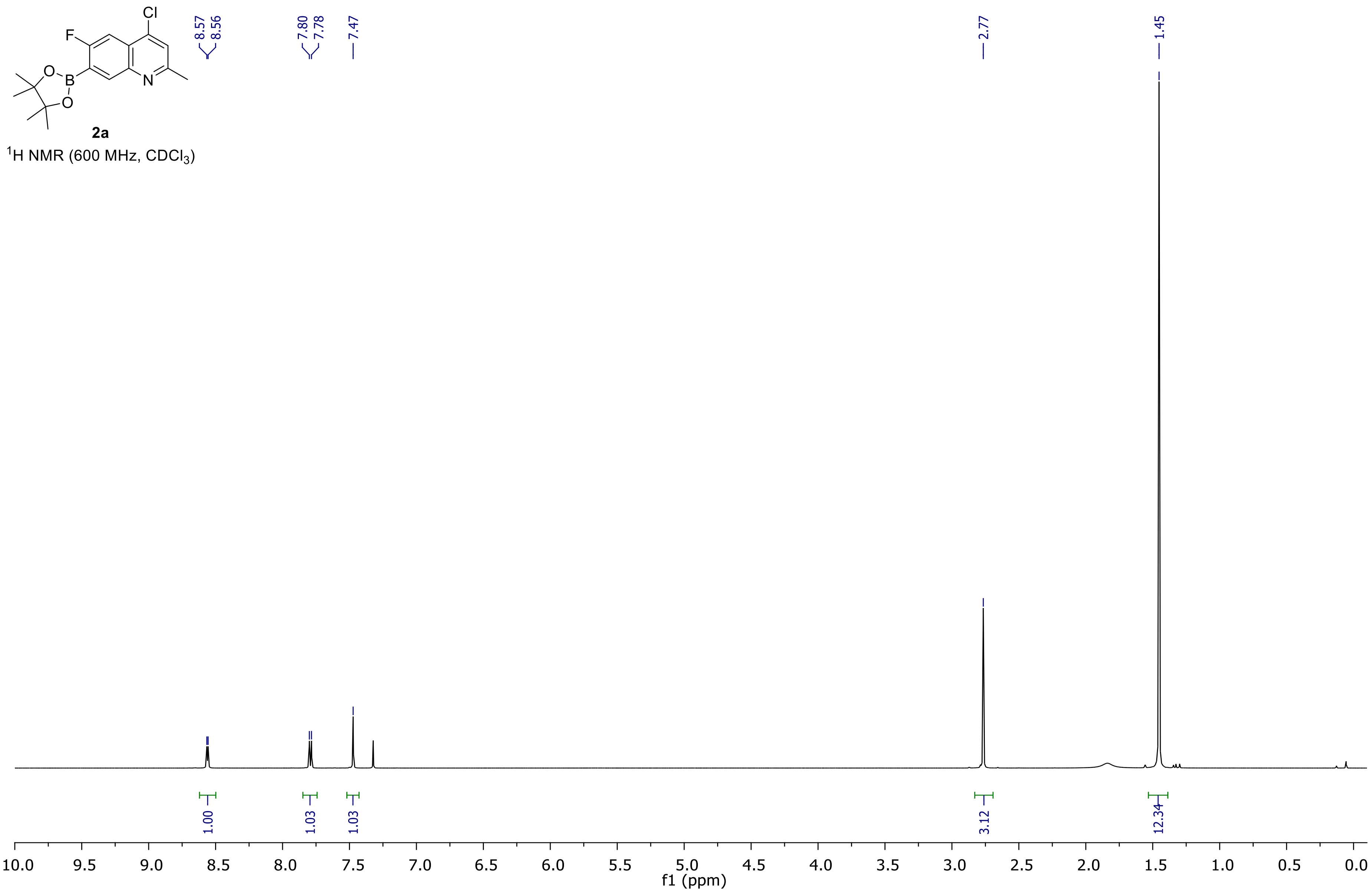

2a

^{1}H NMR (600 MHz, $CDCl_3$)

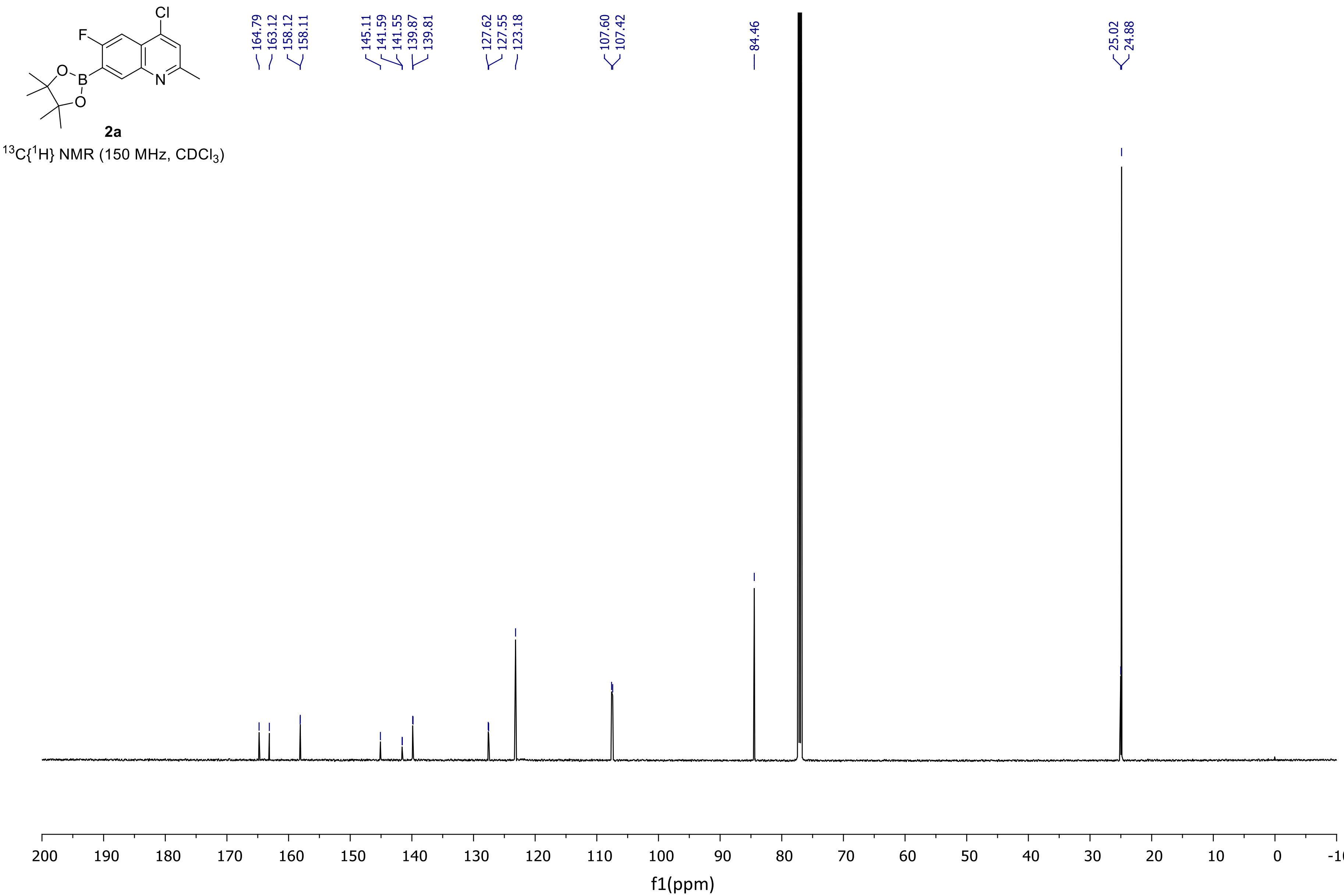
Cl
F
O
B
O
N
2a
$^{13}C\{^{1}H\}$ NMR (150 MHz, $CDCl_3$)
164.79
163.12
158.12
158.11
145.11
141.59
141.55
139.87
139.81
127.62
127.55
123.18
107.60
107.42
84.46
25.02
24.88
200 190 180 170 160 150 140 130 120 110 100 90 80 70 60 50 40 30 20 10 0 -10
f1(ppm)

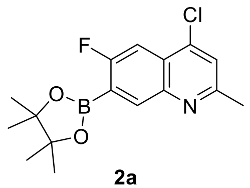

2a

^{19}F NMR (282 MHz, $CDCl_3$)

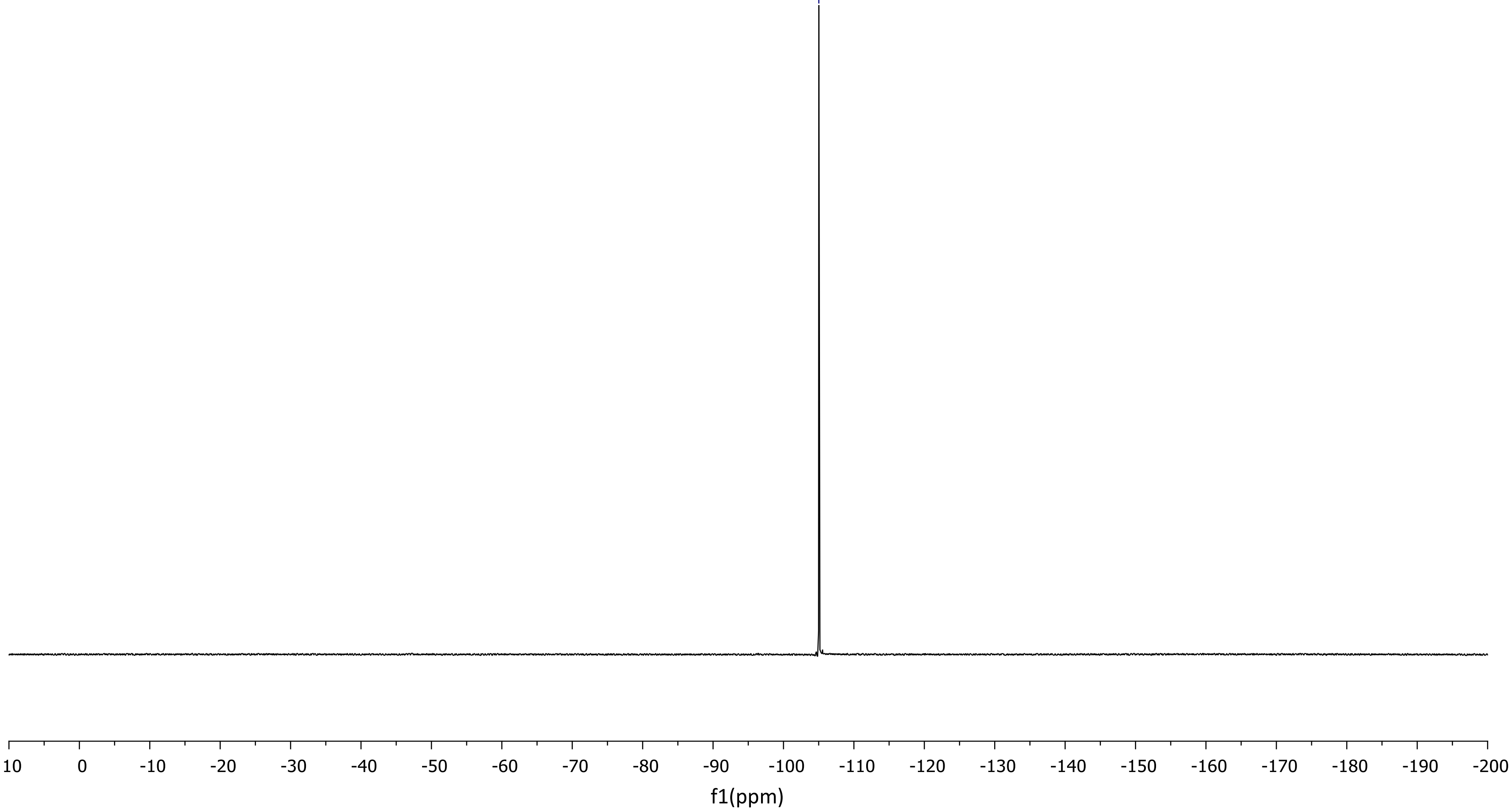

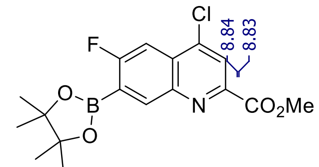

2d

^{1}H NMR (600 MHz, $CDCl_3$)

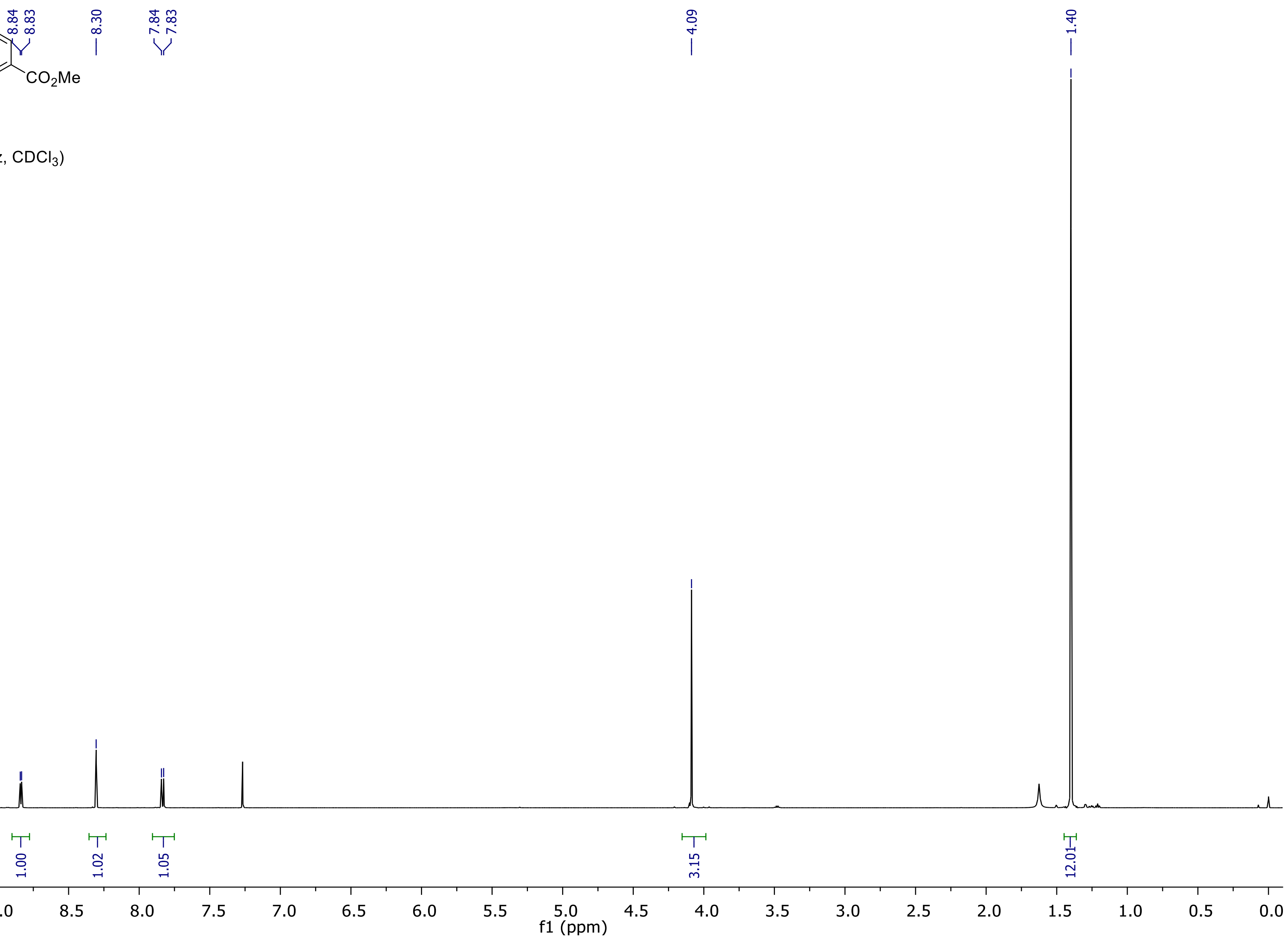

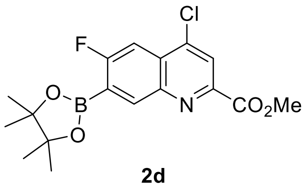

2d

$^{13}C\{^1H\}$ NMR (150 MHz, $CDCl_3$)

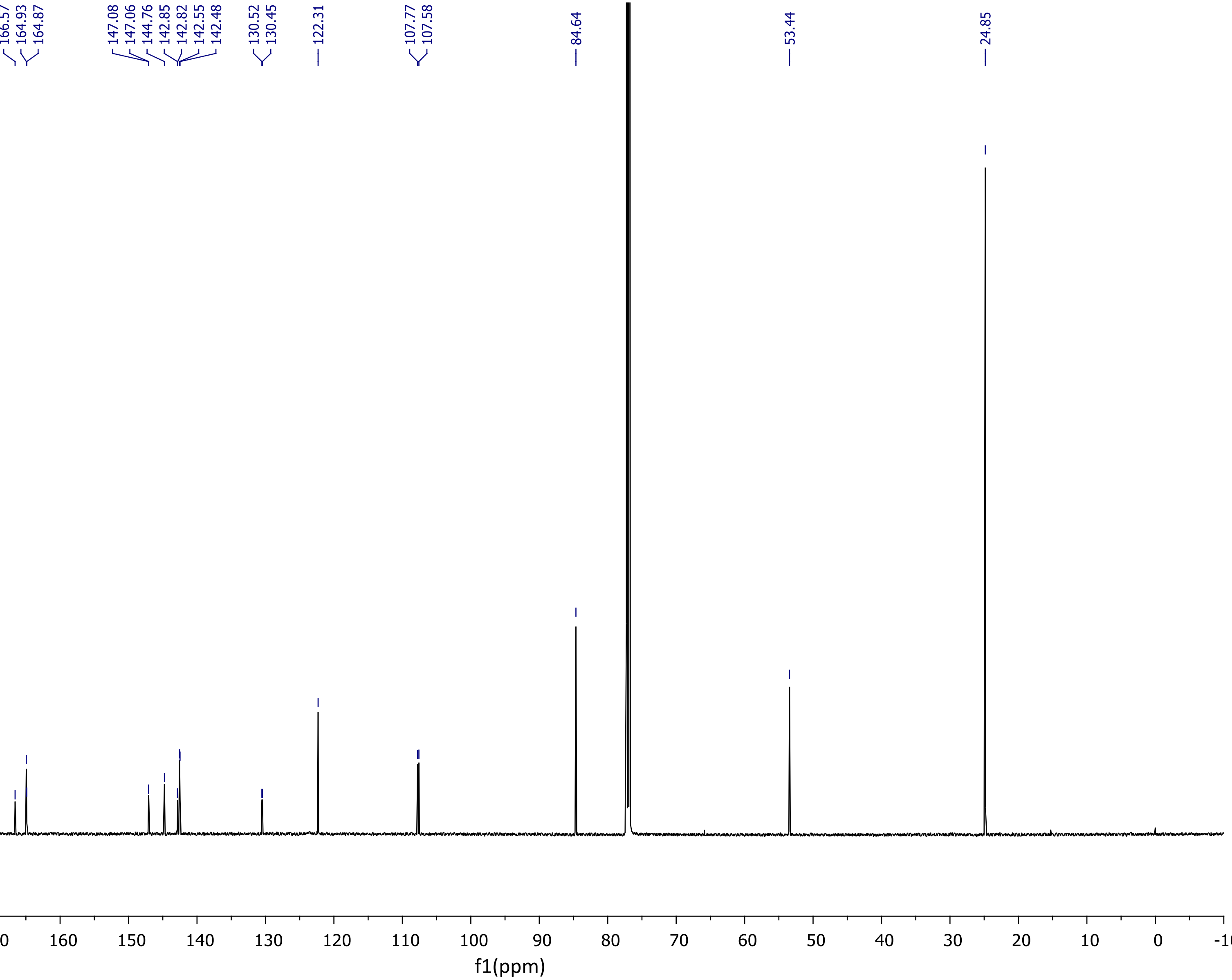

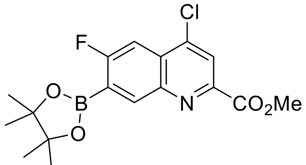

2d

^{19}F NMR (282 MHz, $CDCl_3$)

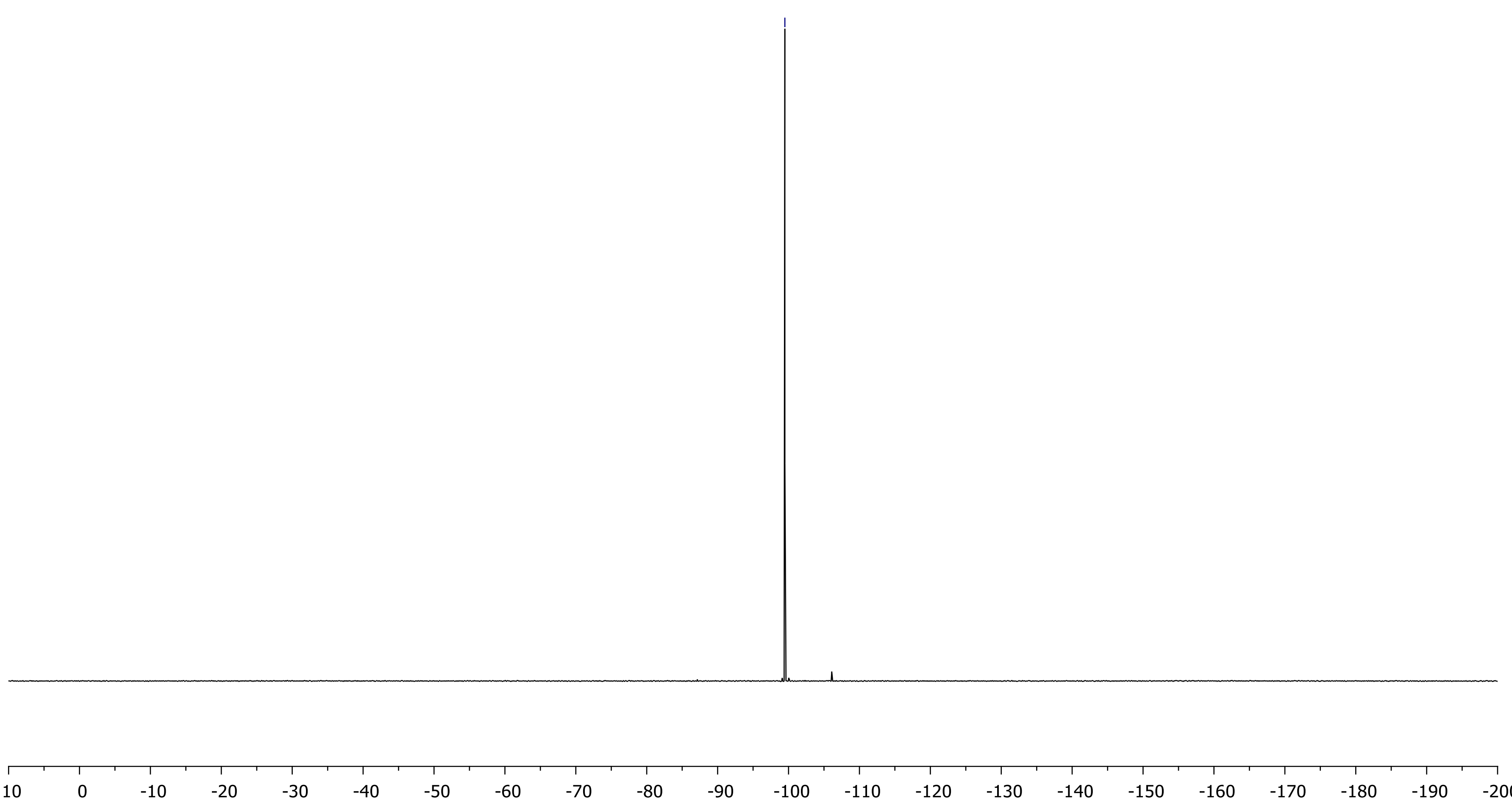

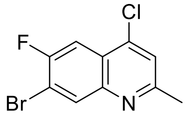

3a

^{1}H NMR (600 MHz, $CDCl_3$)

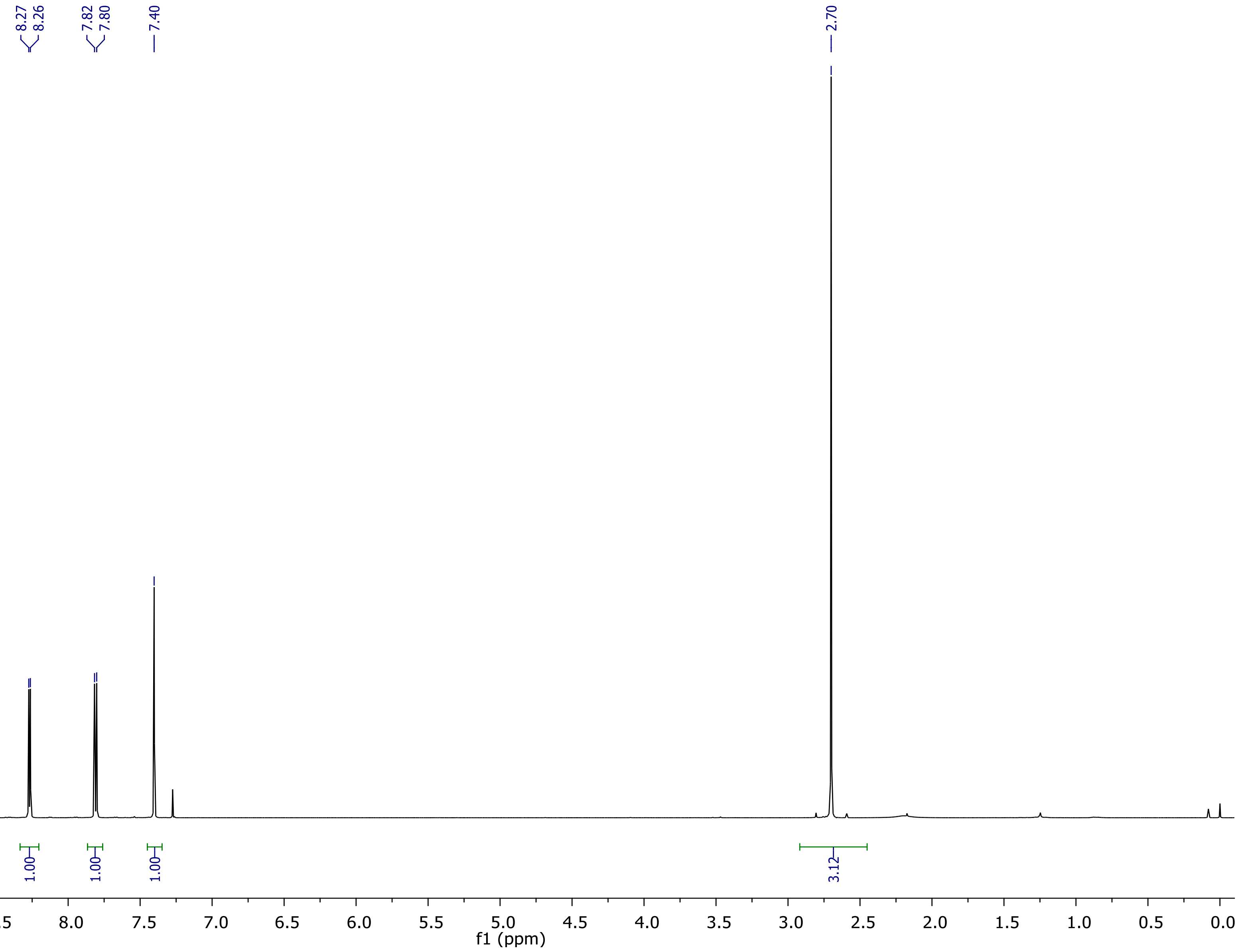

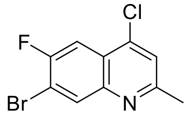

3a

$^{13}C\{^{1}H\}$ NMR (150 MHz, $CDCl_3$)

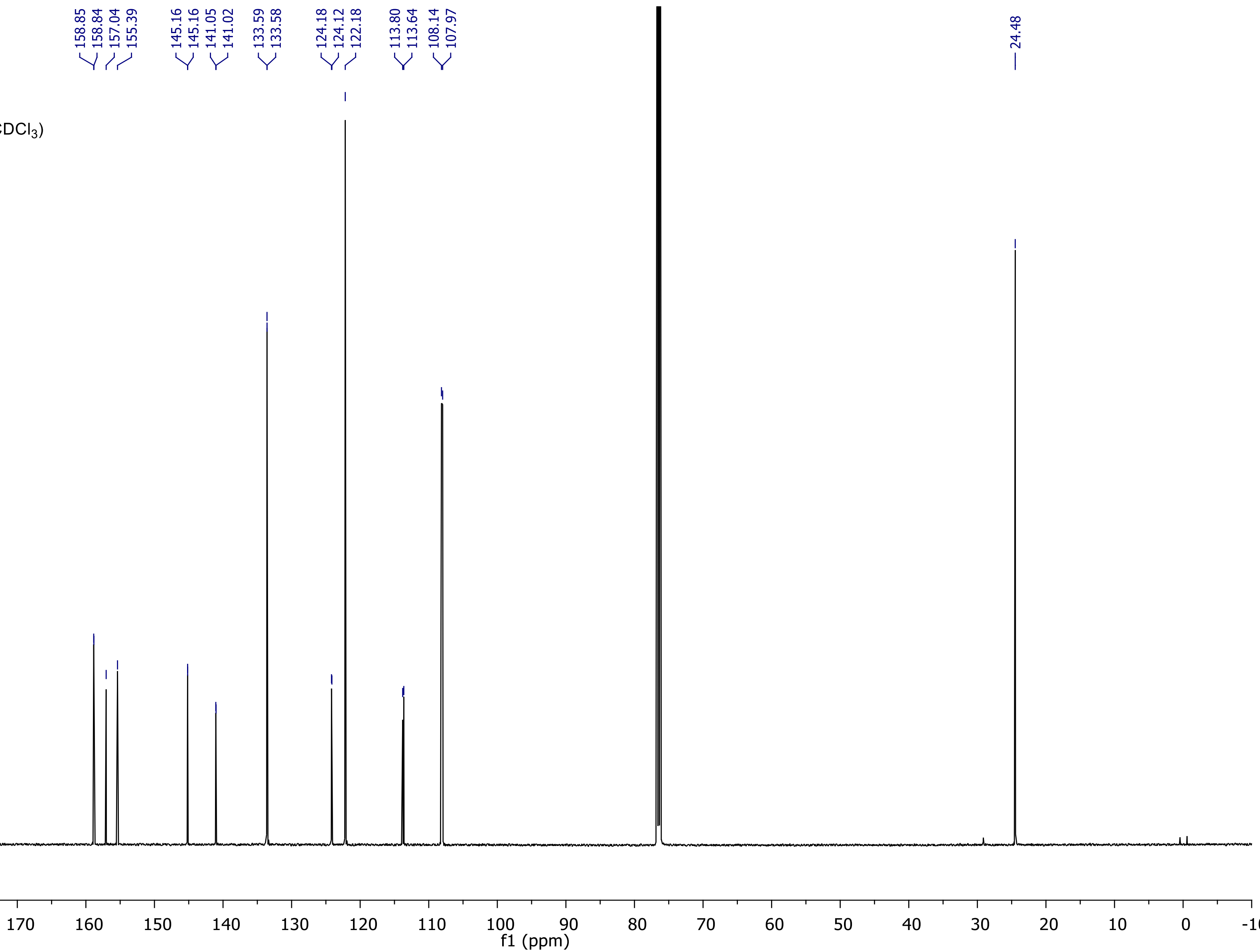

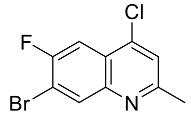

3a
^{19}F NMR (282 MHz, $CDCl_3$)

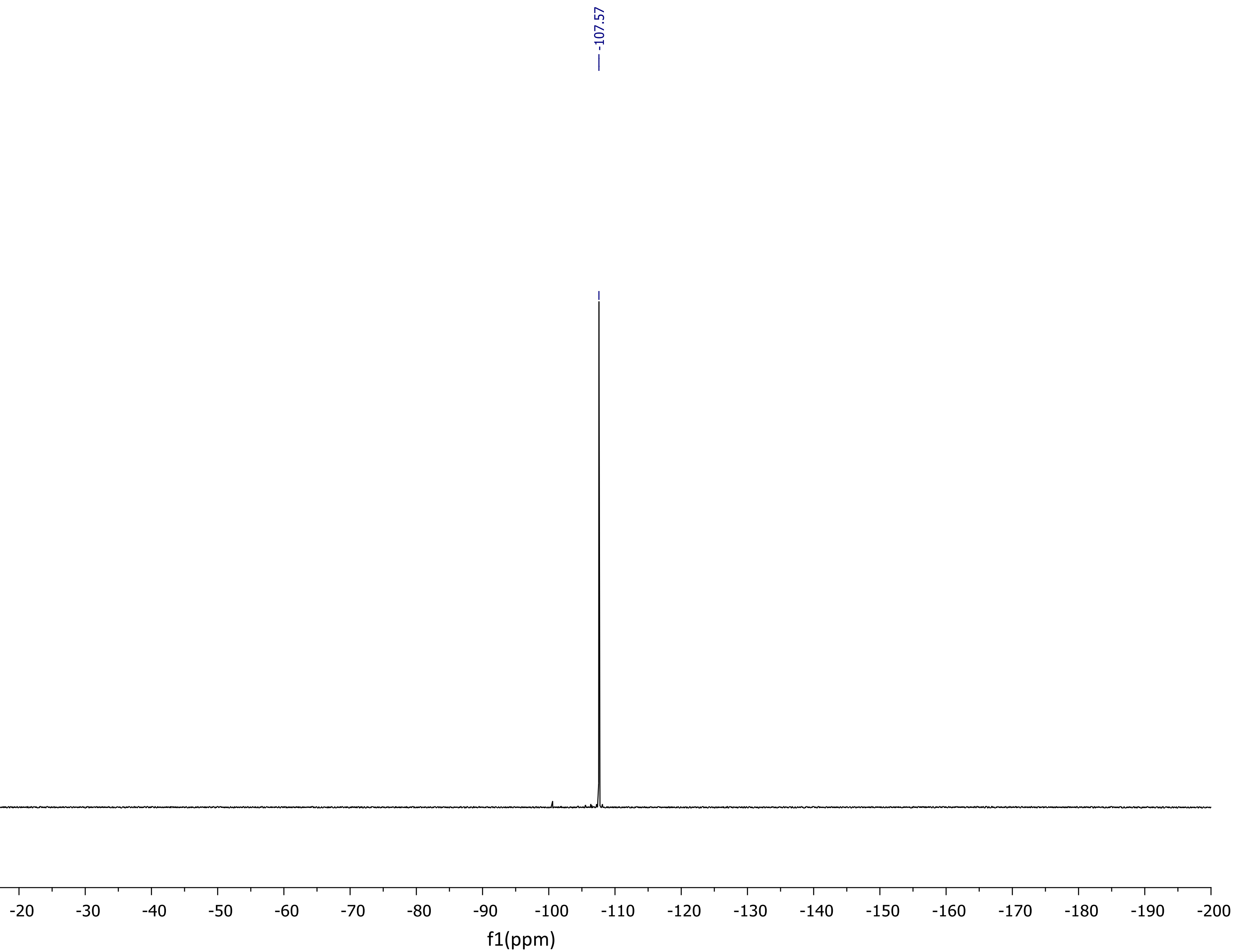

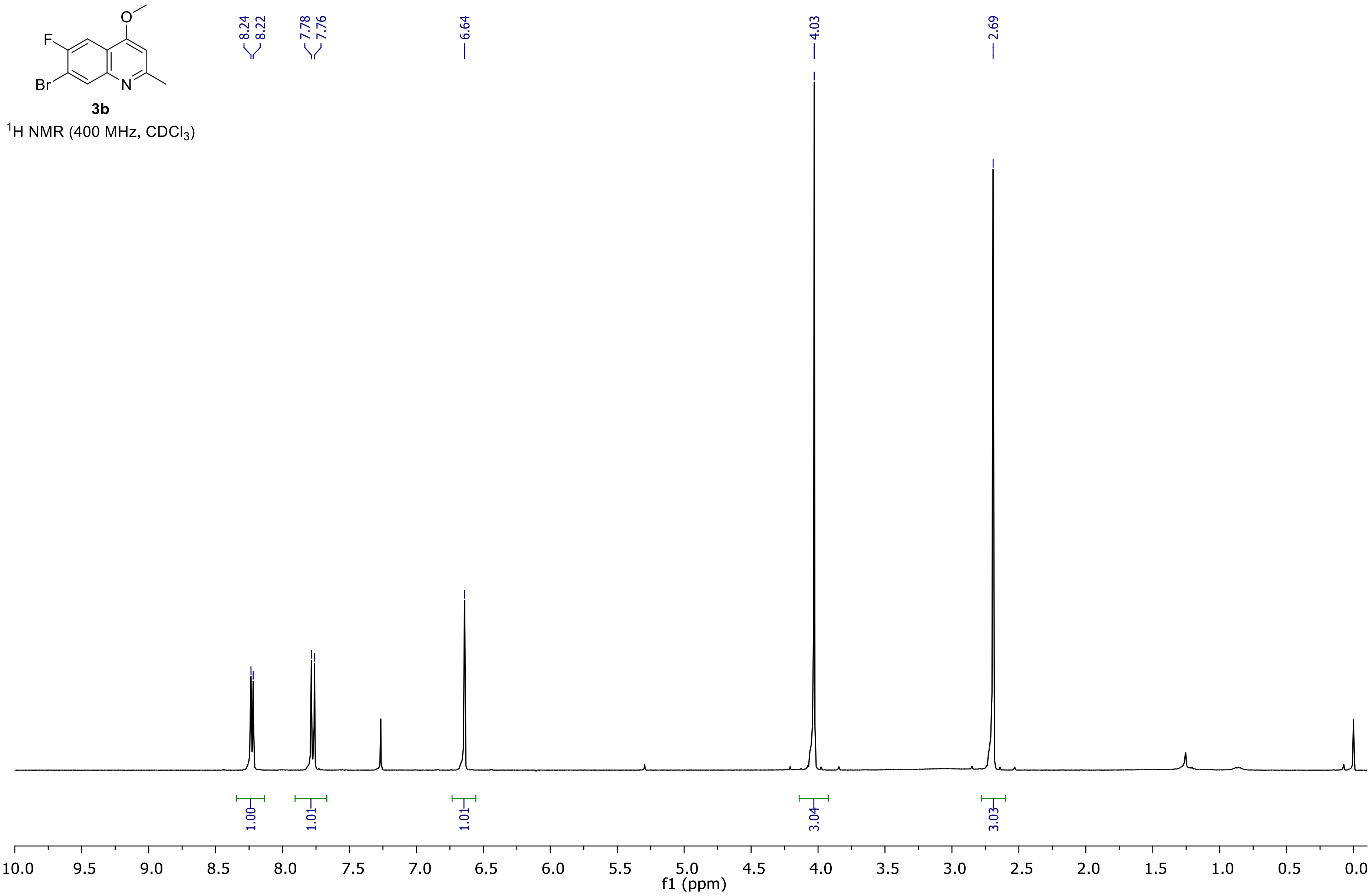

3b

^{1}H NMR (400 MHz, $CDCl_3$)

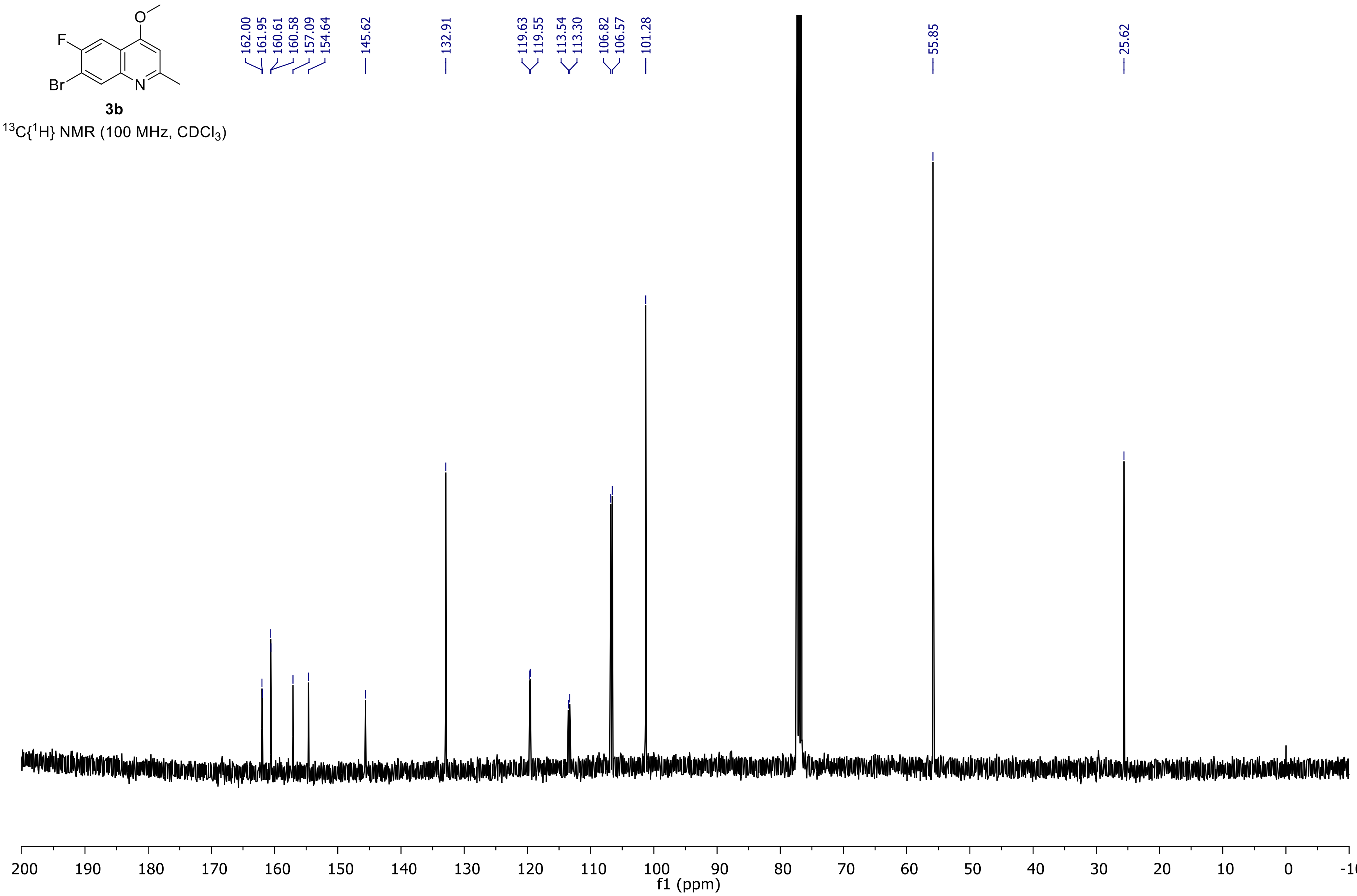
F
Br
N
O
3b
$^{13}C\{^1H\}$ NMR (100 MHz, $CDCl_3$)
162.00
161.95
160.61
160.58
157.09
154.64
145.62
132.91
119.63
119.55
113.54
113.30
106.82
106.57
101.28
55.85
25.62
200 190 180 170 160 150 140 130 120 110 100 90 80 70 60 50 40 30 20 10 0 -10
f1 (ppm)

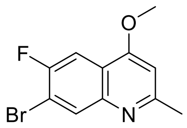

3b

^{19}F NMR (282 MHz, $CDCl_3$)

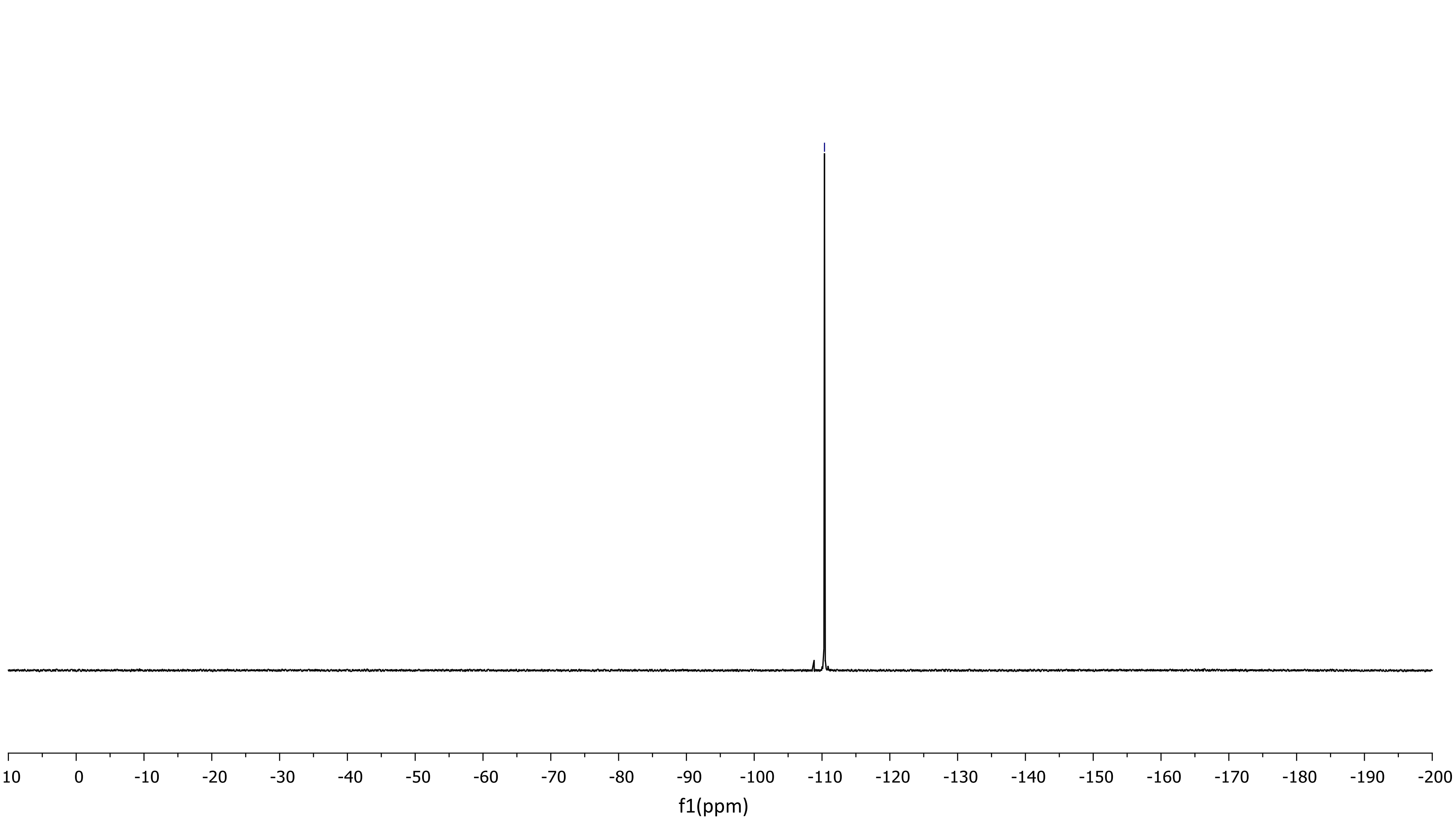

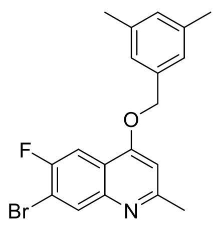

3c

^{1}H NMR (500 MHz, $CDCl_3$)

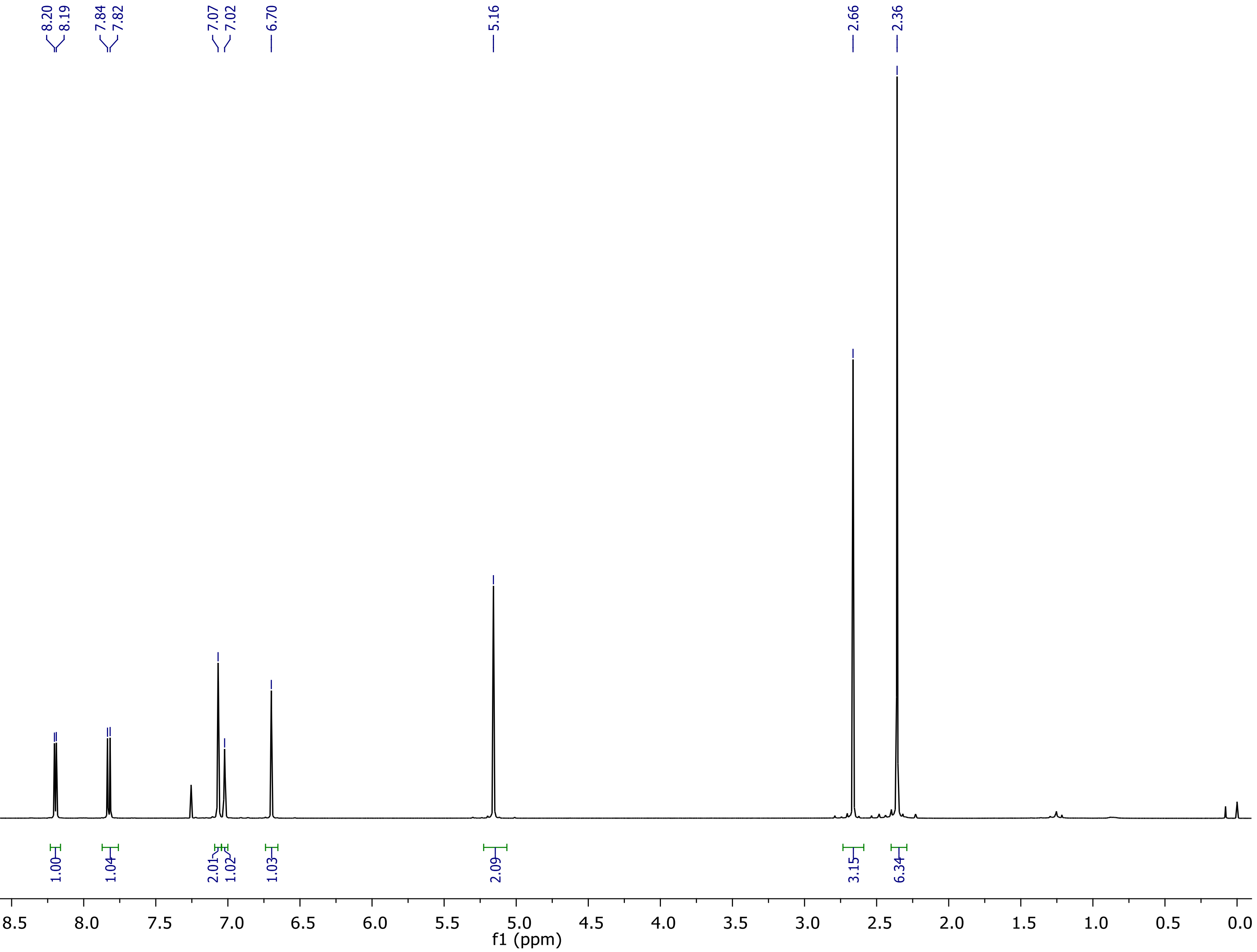

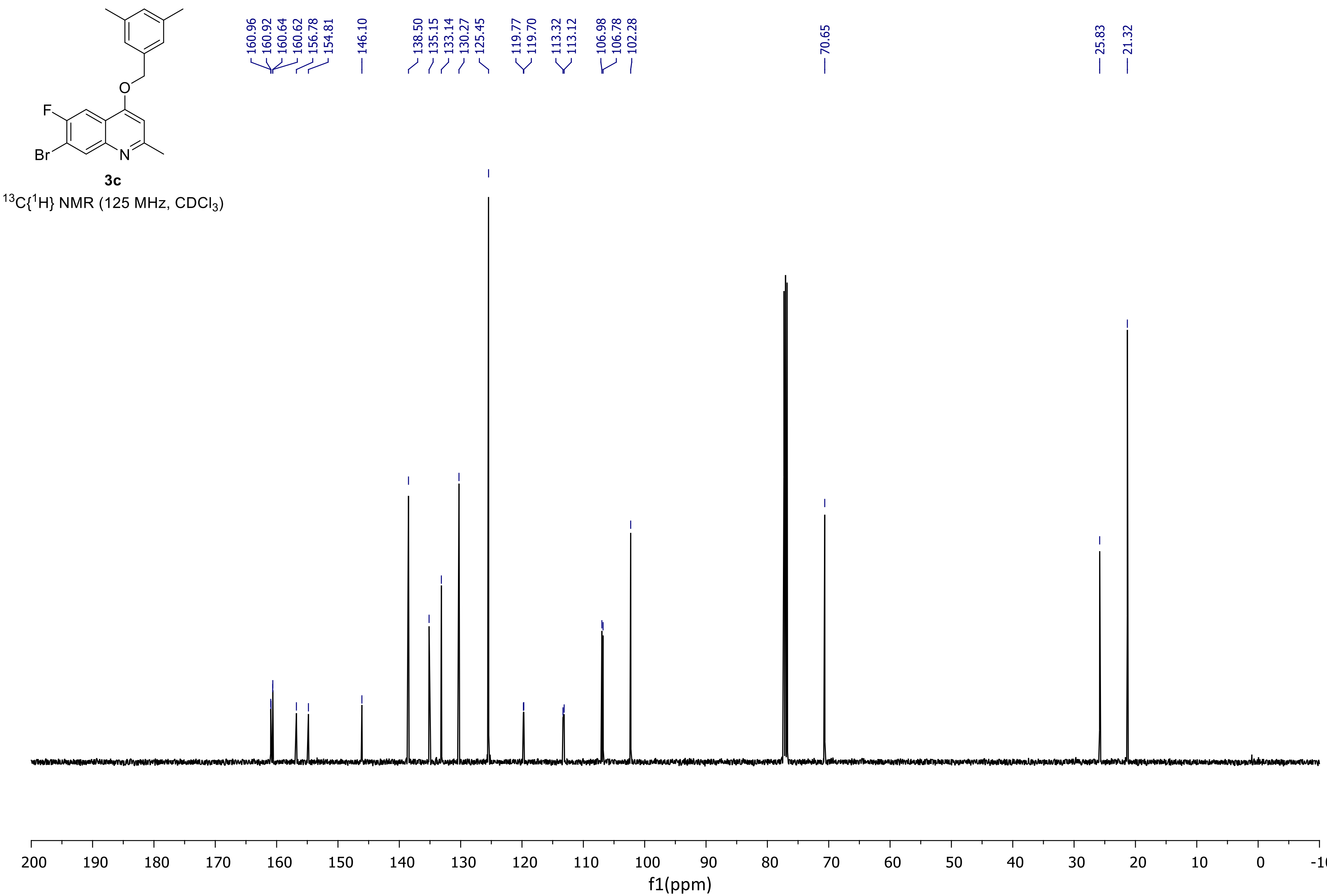

F
Br
N
O
3c
$^{13}C\{^1H\}$ NMR (125 MHz, $CDCl_3$)
160.96
160.92
160.64
160.62
156.78
154.81
146.10
138.50
135.15
133.14
130.27
125.45
119.77
119.70
113.32
113.12
106.98
106.78
102.28
70.65
25.83
21.32
200
190
180
170
160
150
140
130
120
110
100
90
80
70
60
50
40
30
20
10
0
-10
f1(ppm)

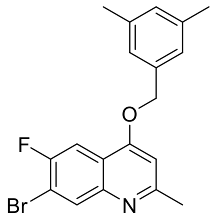

3c

^{19}F NMR (282 MHz, $CDCl_3$)

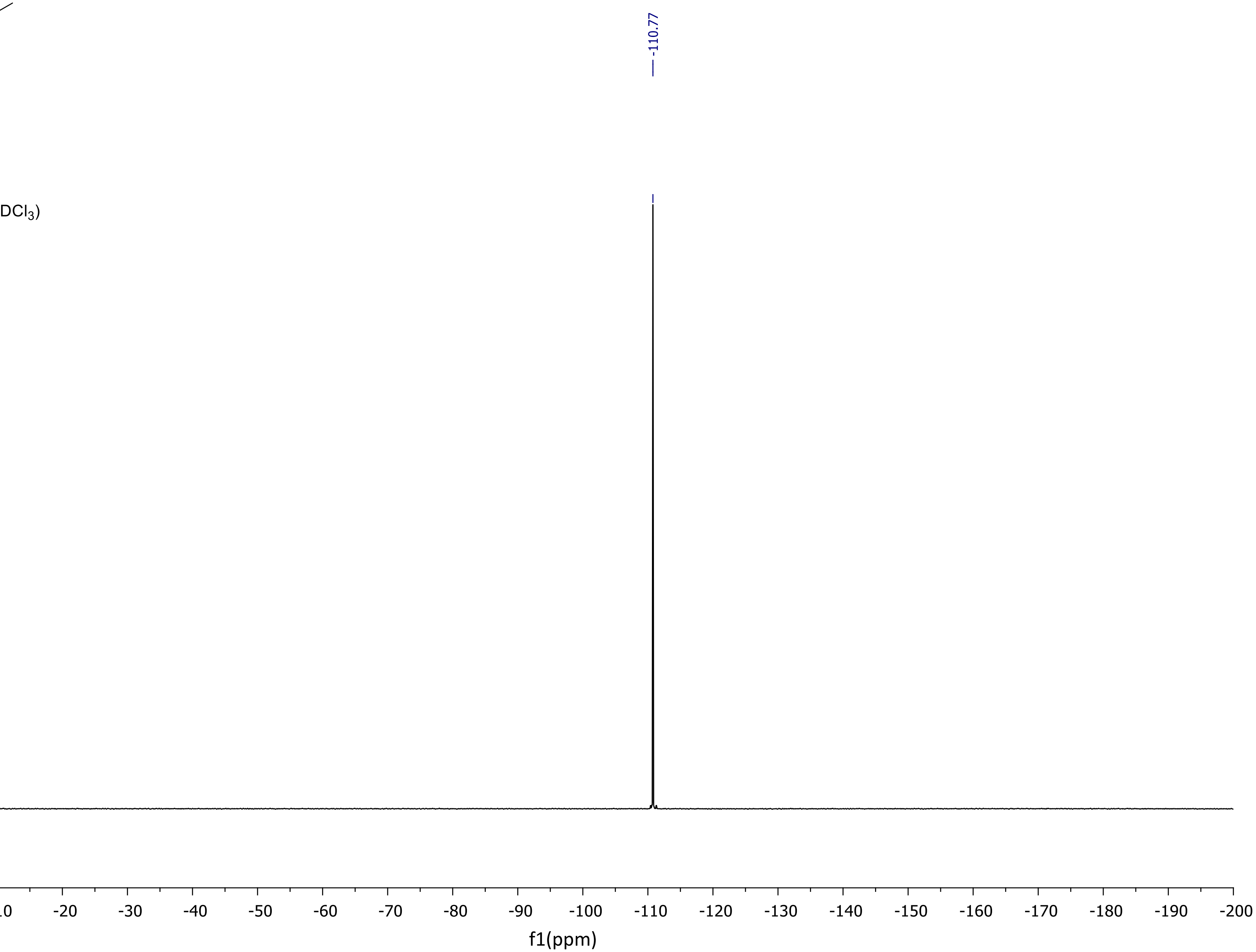

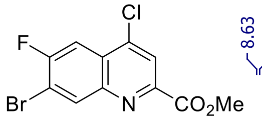

3d

^1H NMR (400 MHz, $CDCl_3$)

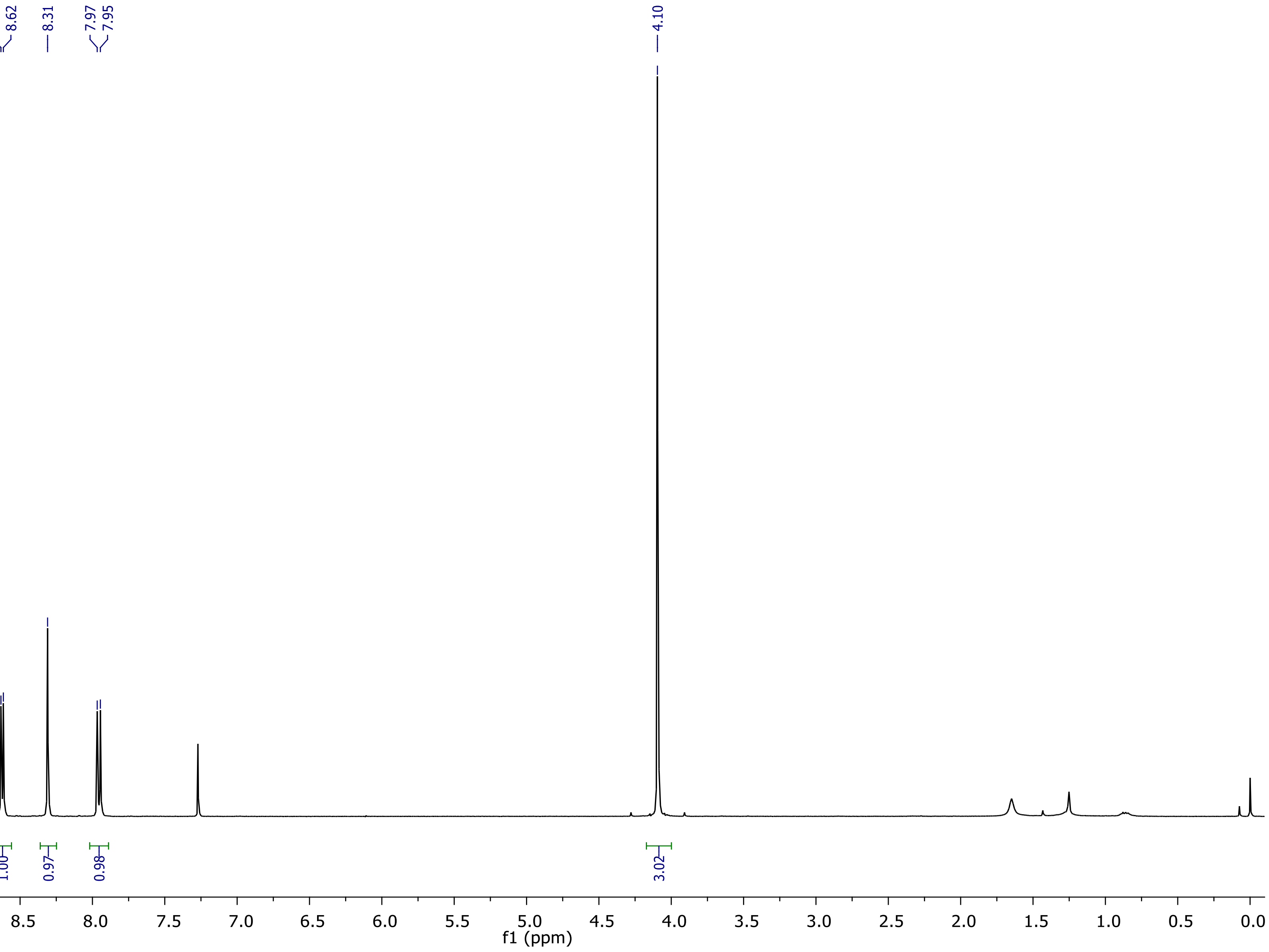

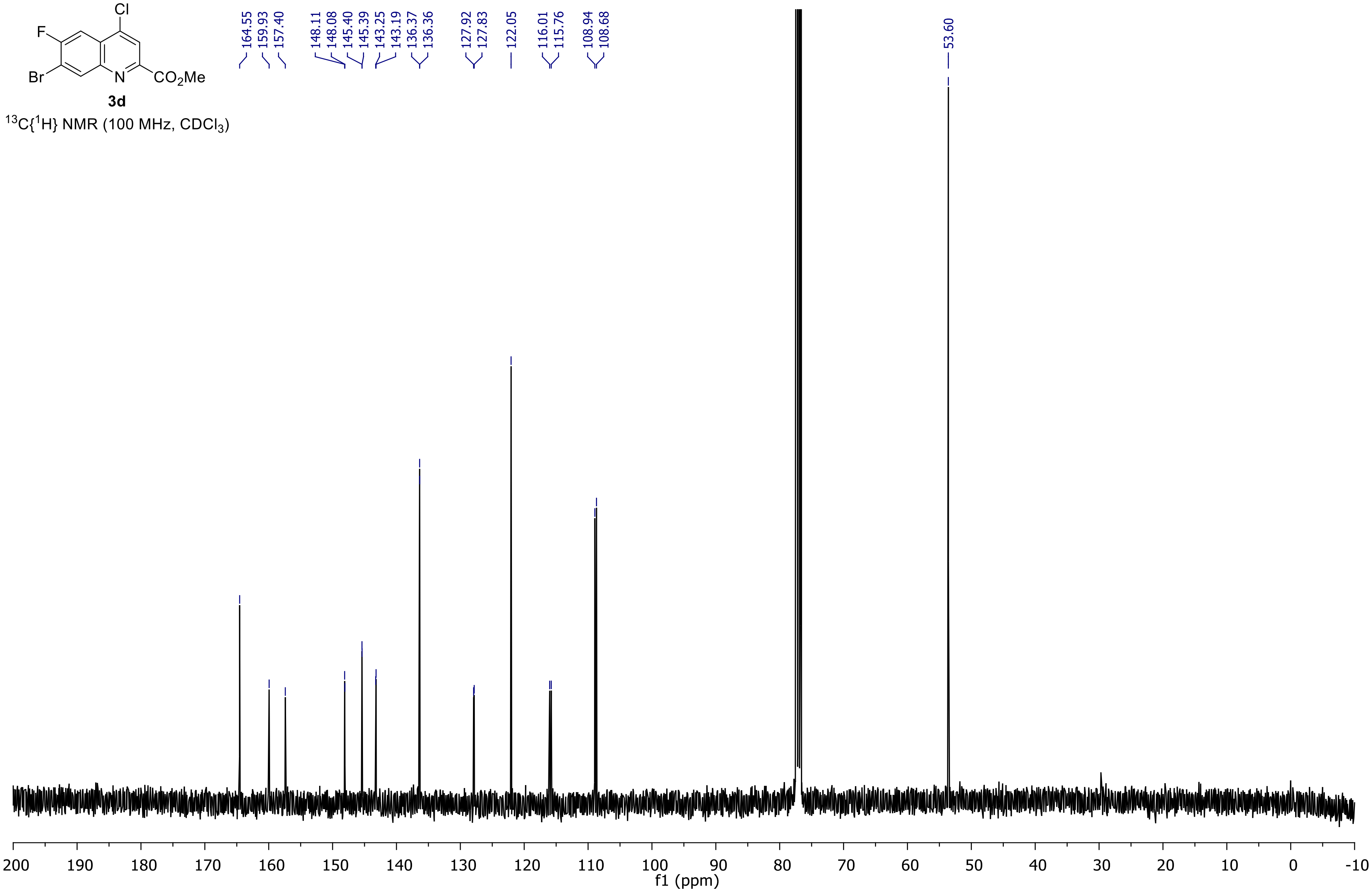

Cl
F
Br
N
CO2Me
3d
13C{1H} NMR (100 MHz, CDCl3)
164.55
159.93
157.40
148.11
148.08
145.40
145.39
143.25
143.19
136.37
136.36
127.92
127.83
122.05
116.01
115.76
108.94
108.68
53.60
200 190 180 170 160 150 140 130 120 110 100 90 80 70 60 50 40 30 20 10 0 -10
f1 (ppm)

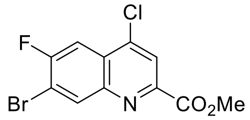

3d

^{19}F NMR (376 MHz, $CDCl_3$)

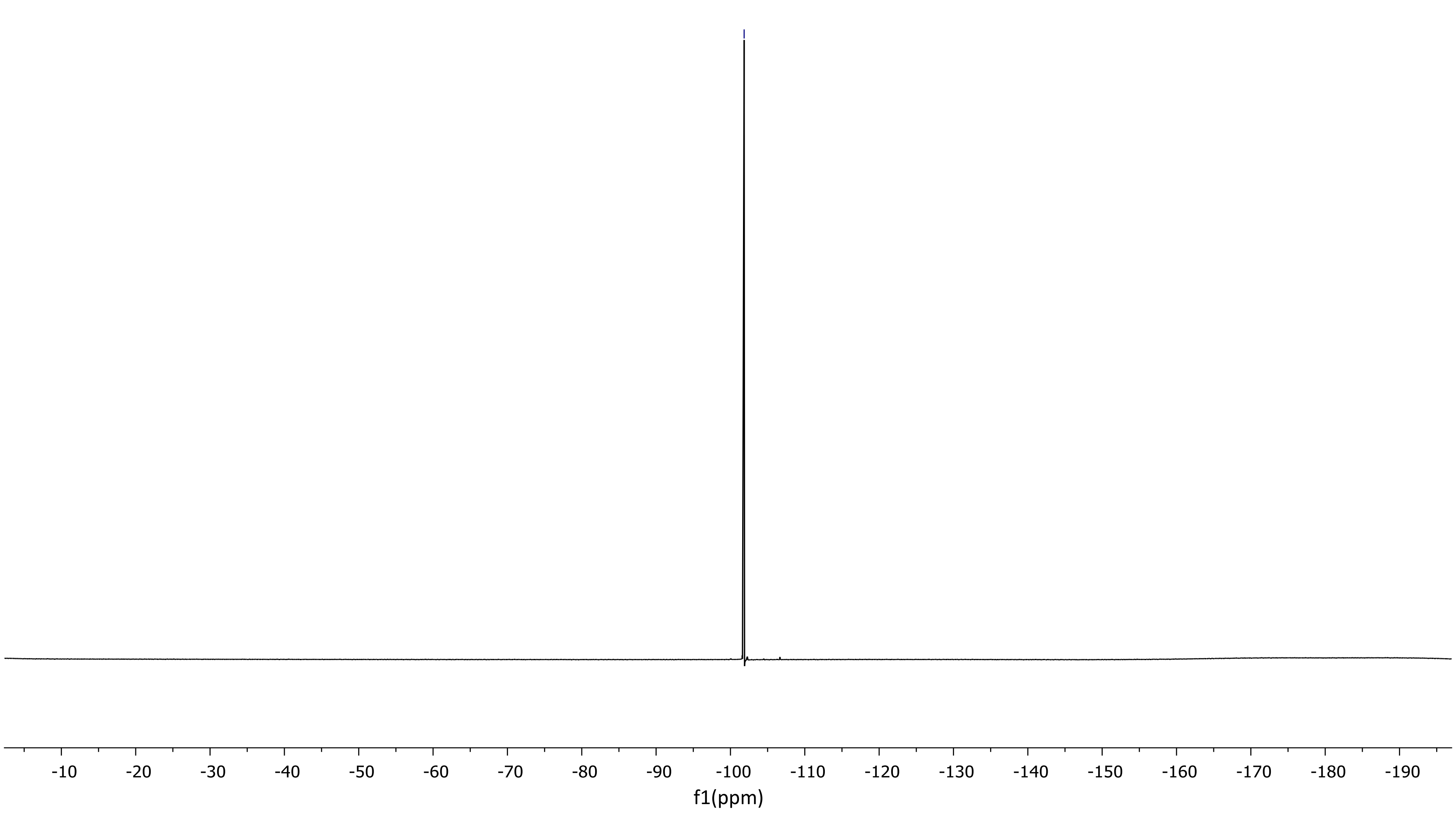

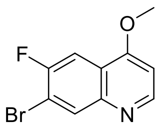

3e

^{1}H NMR (400 MHz, $CDCl_3$)

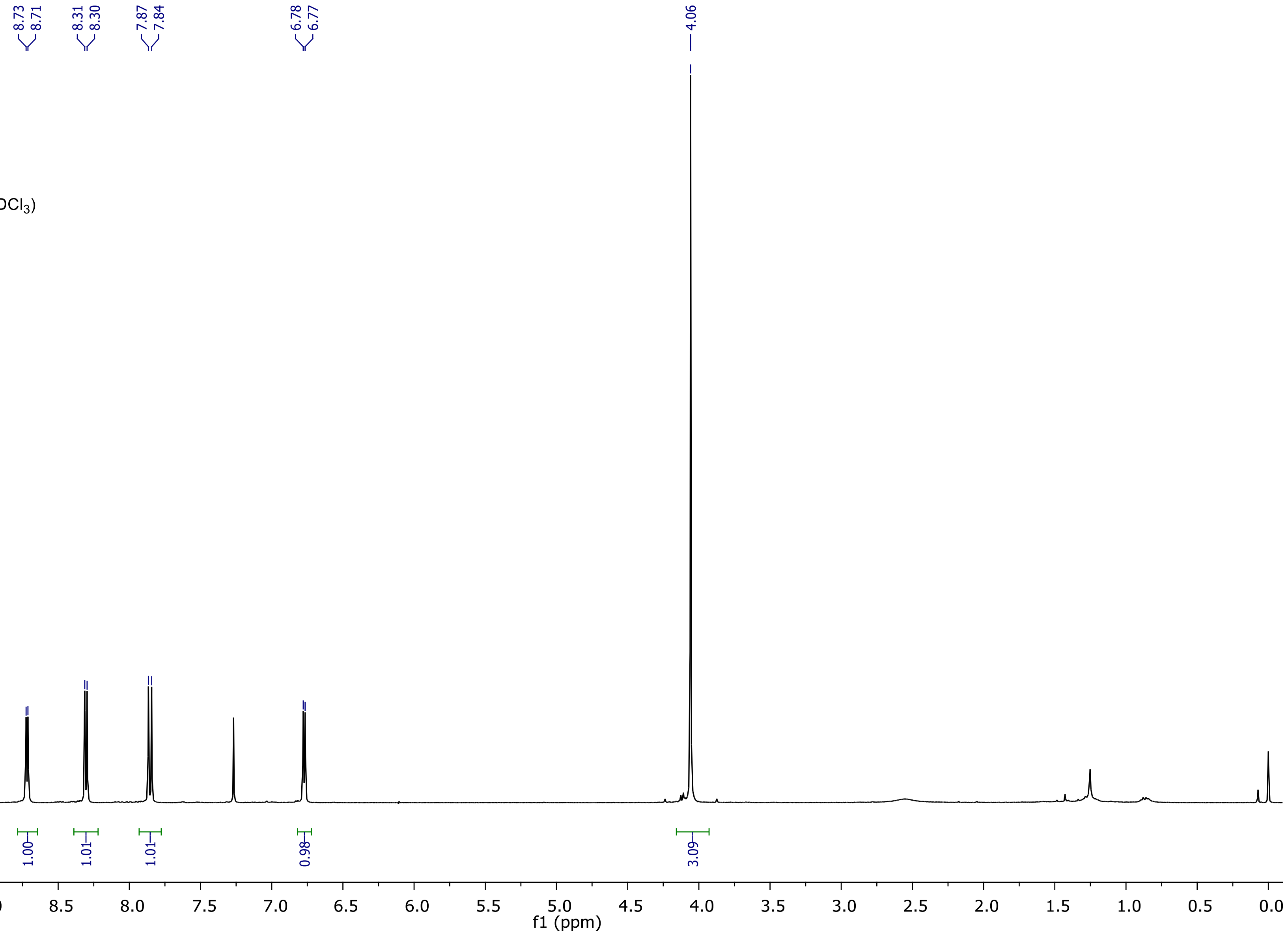

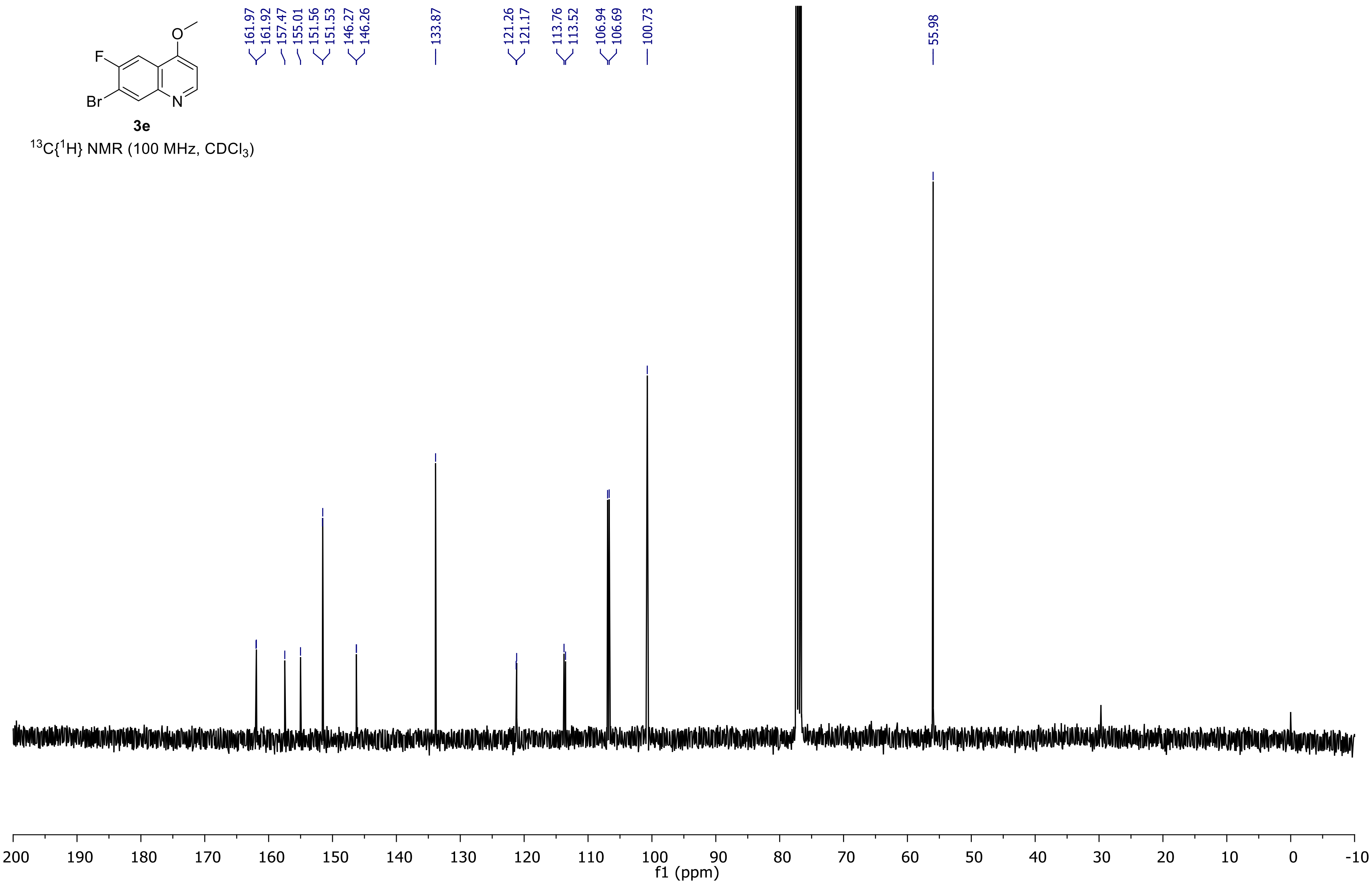

3e

$^{13}C\{^1H\}$ NMR (100 MHz, $CDCl_3$)

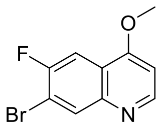

3e

^{19}F NMR (376 MHz, $CDCl_3$)

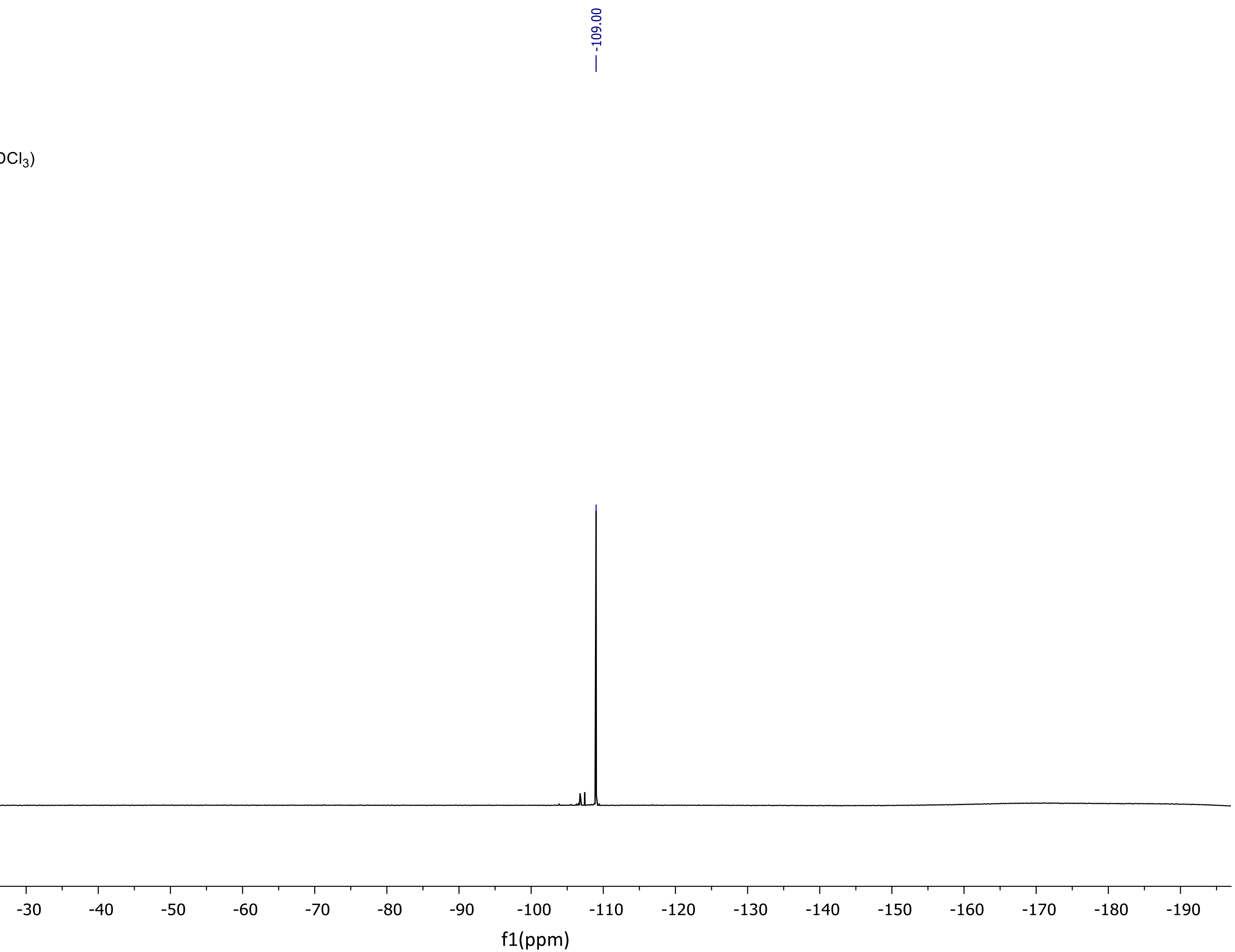

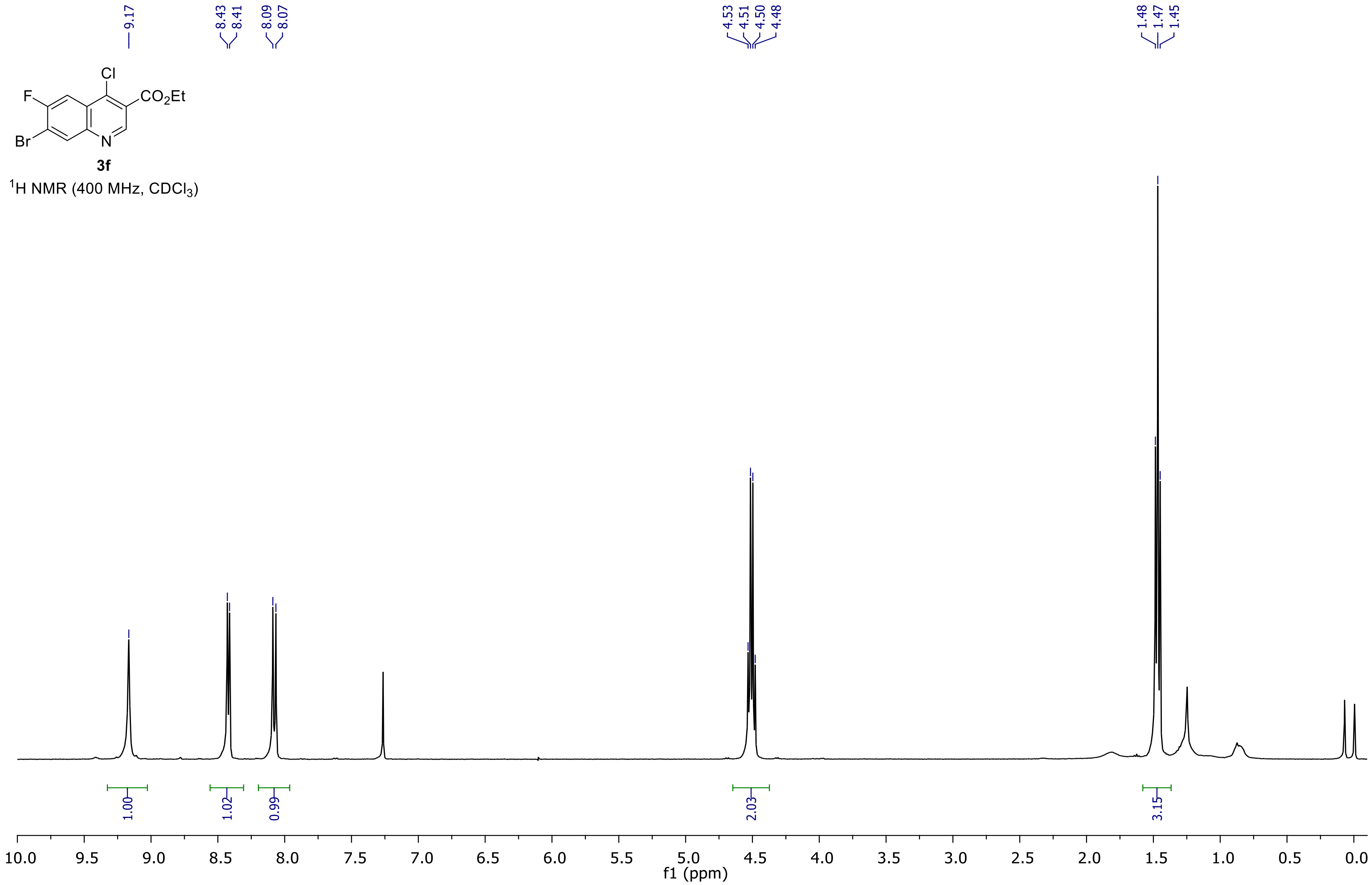
9.17
8.43
8.41
8.09
8.07
4.53
4.51
4.50
4.48
1.48
1.47
1.45
Cl
F
CO2Et
Br
N
3f
1H NMR (400 MHz, CDCl3)
1.00
1.02
0.99
2.03
3.15
10.0
9.5
9.0
8.5
8.0
7.5
7.0
6.5
6.0
5.5
5.0
4.5
4.0
3.5
3.0
2.5
2.0
1.5
1.0
0.5
0.0
f1 (ppm)

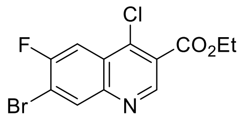

3f

$^{13}C\{^{1}H\}$ NMR (100 MHz, $CDCl_3$)

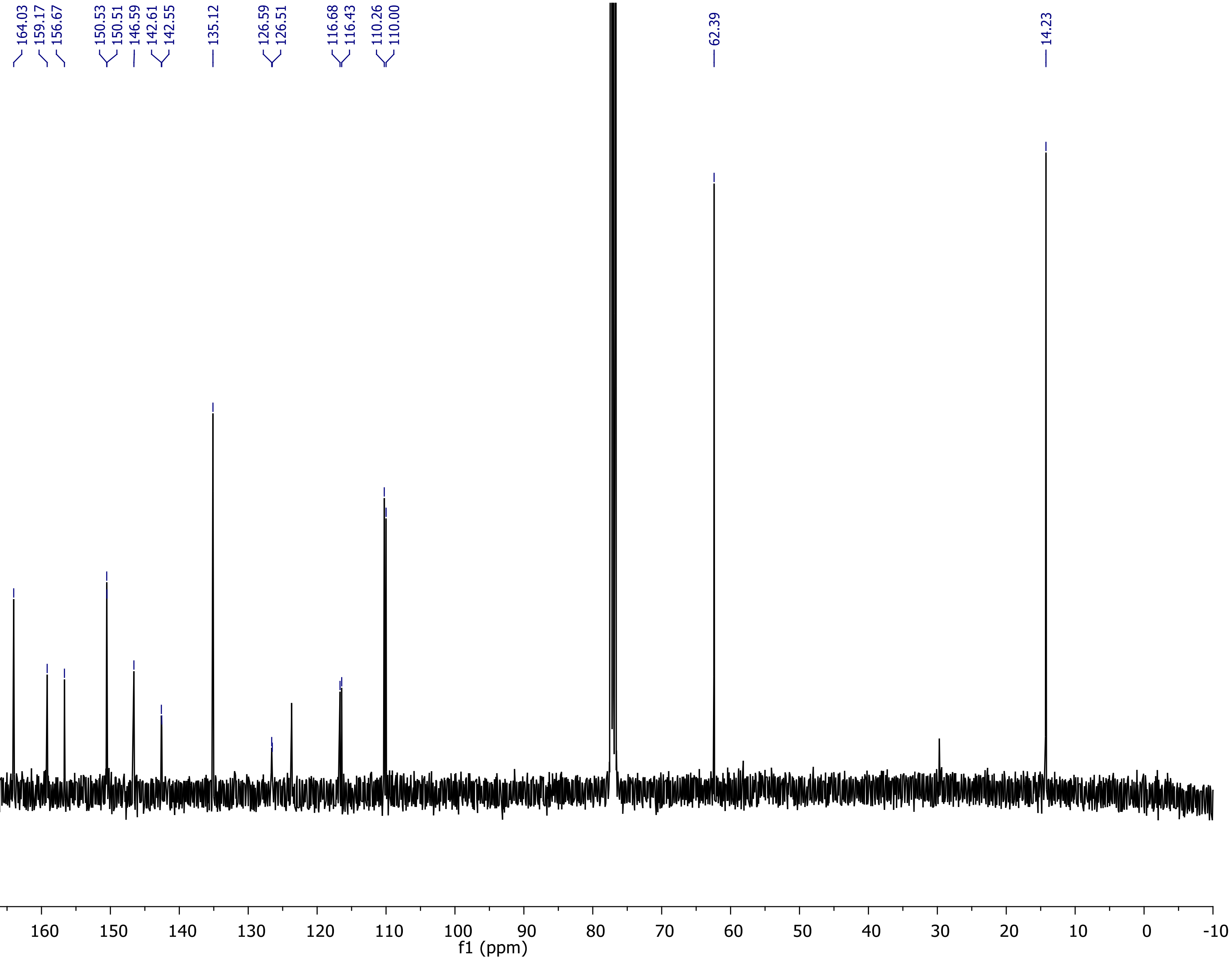

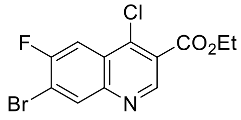

3f

^{19}F NMR (376 MHz, $CDCl_3$)

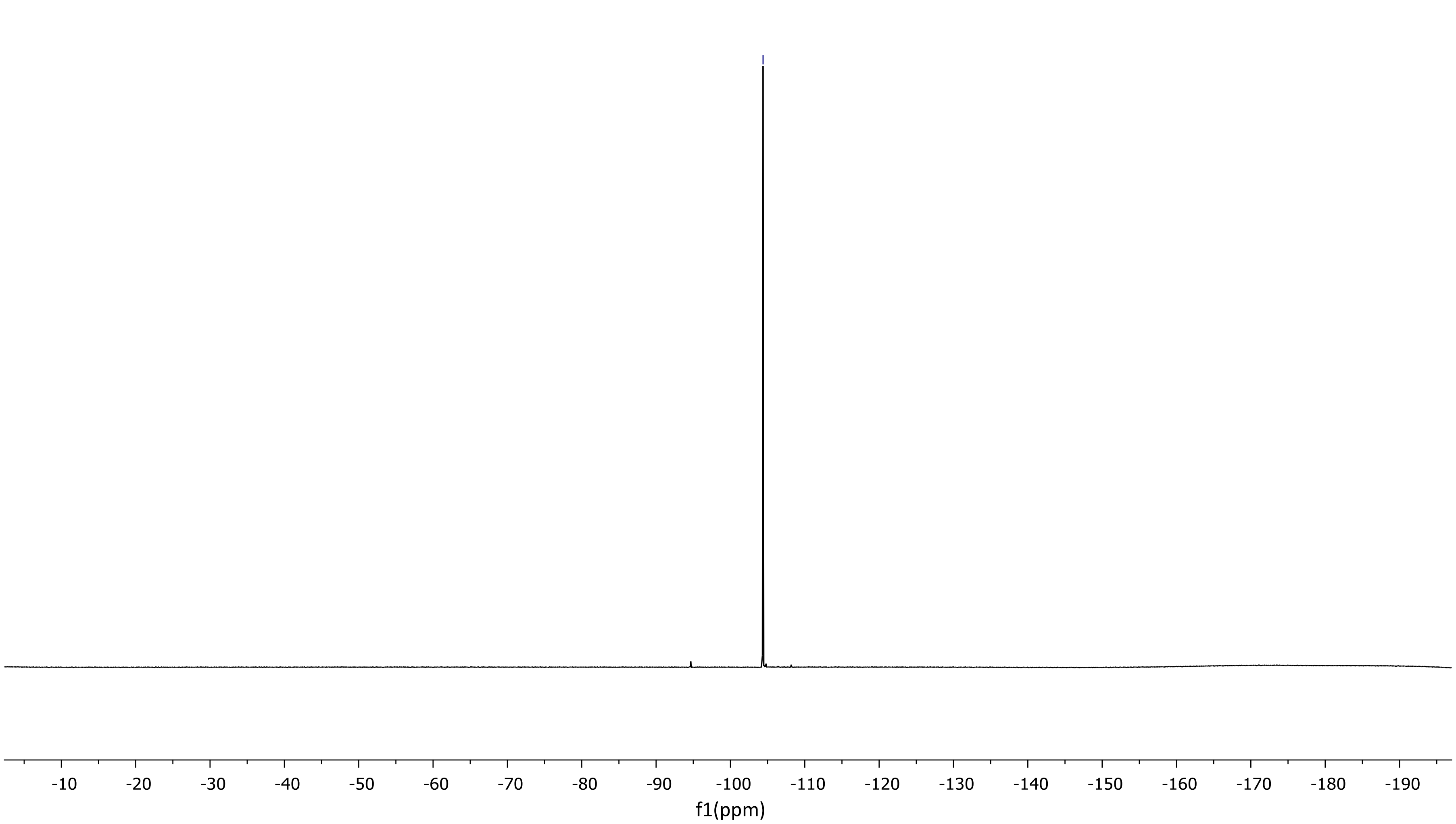

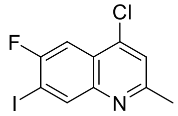

4a

^{1}H NMR (300 MHz, $CDCl_3$)

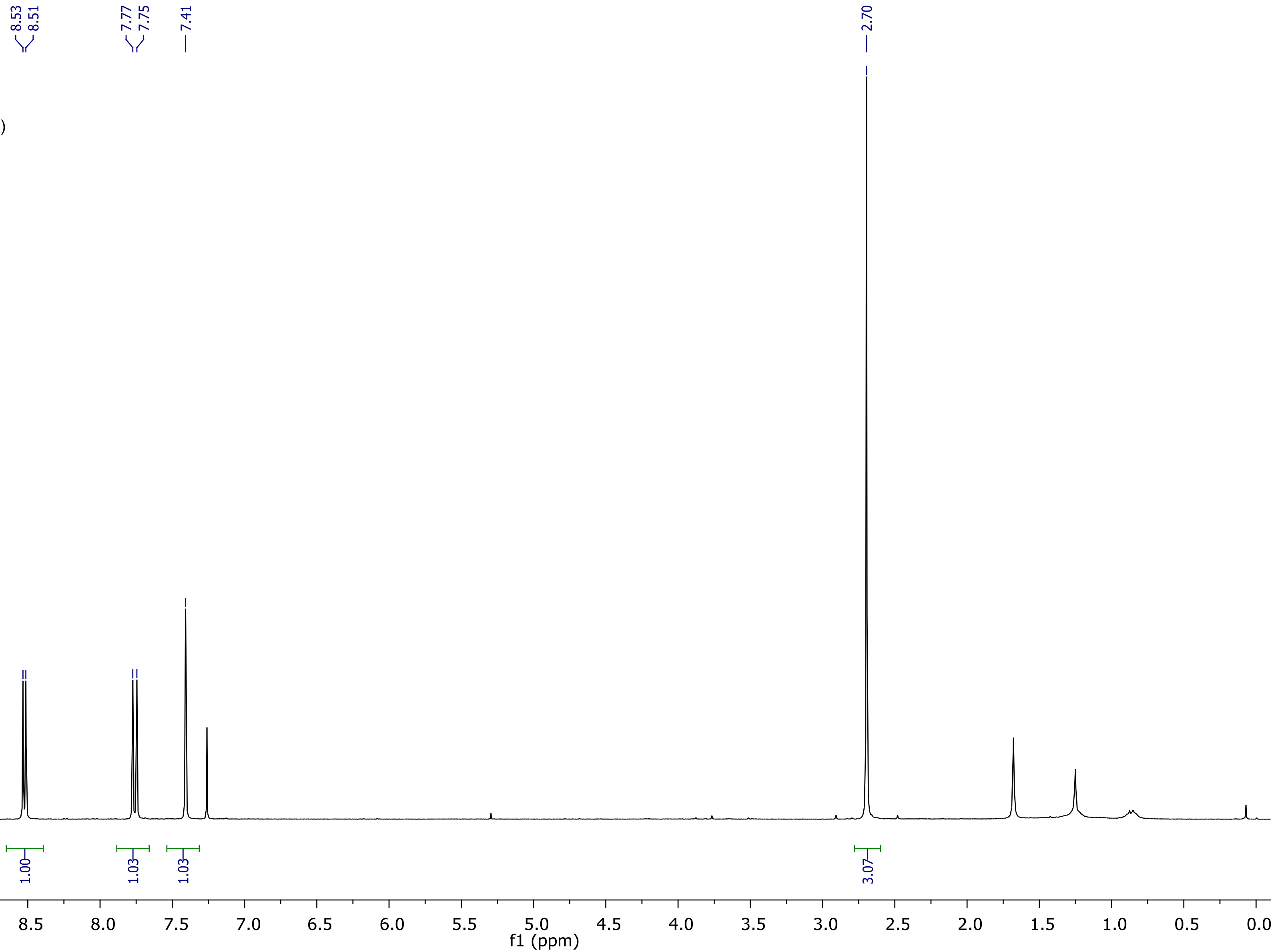

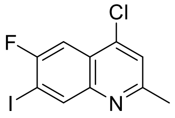

4a

$^{13}C\{^1H\}$ NMR (75 MHz, $CDCl_3$)

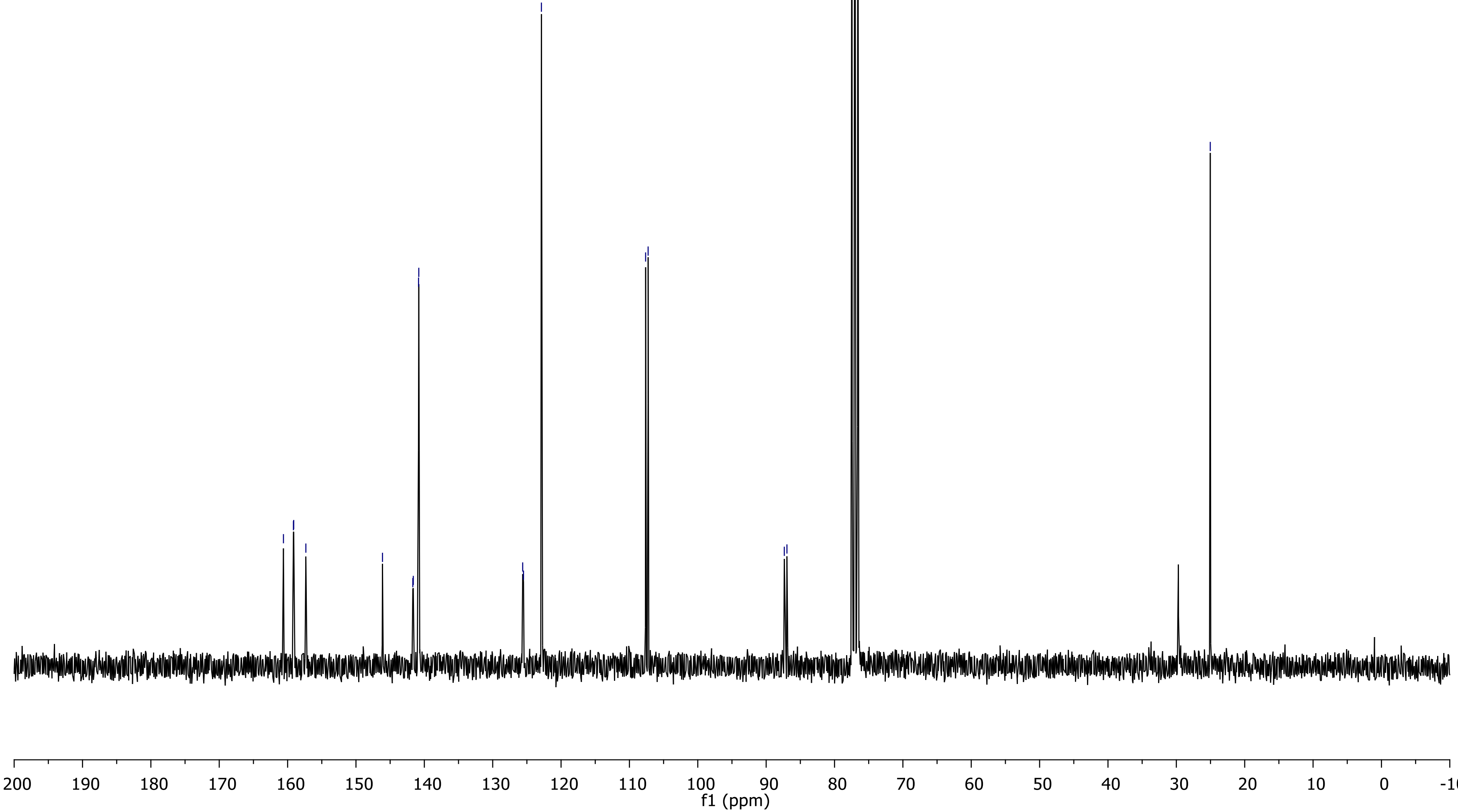

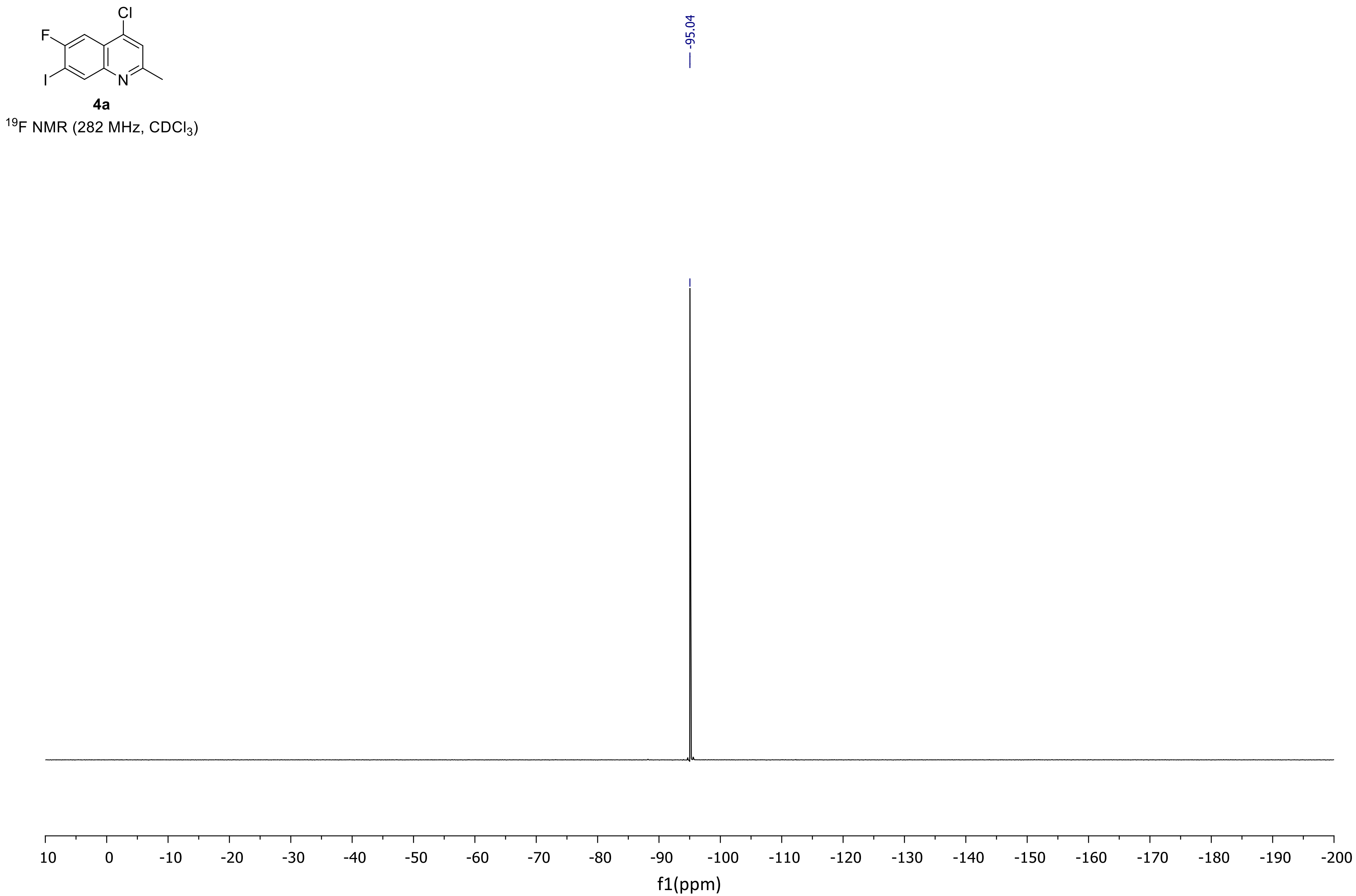

4a

^{19}F NMR (282 MHz, $CDCl_3$)

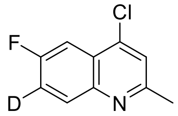

5a

^1H NMR (400 MHz, $CDCl_3$)

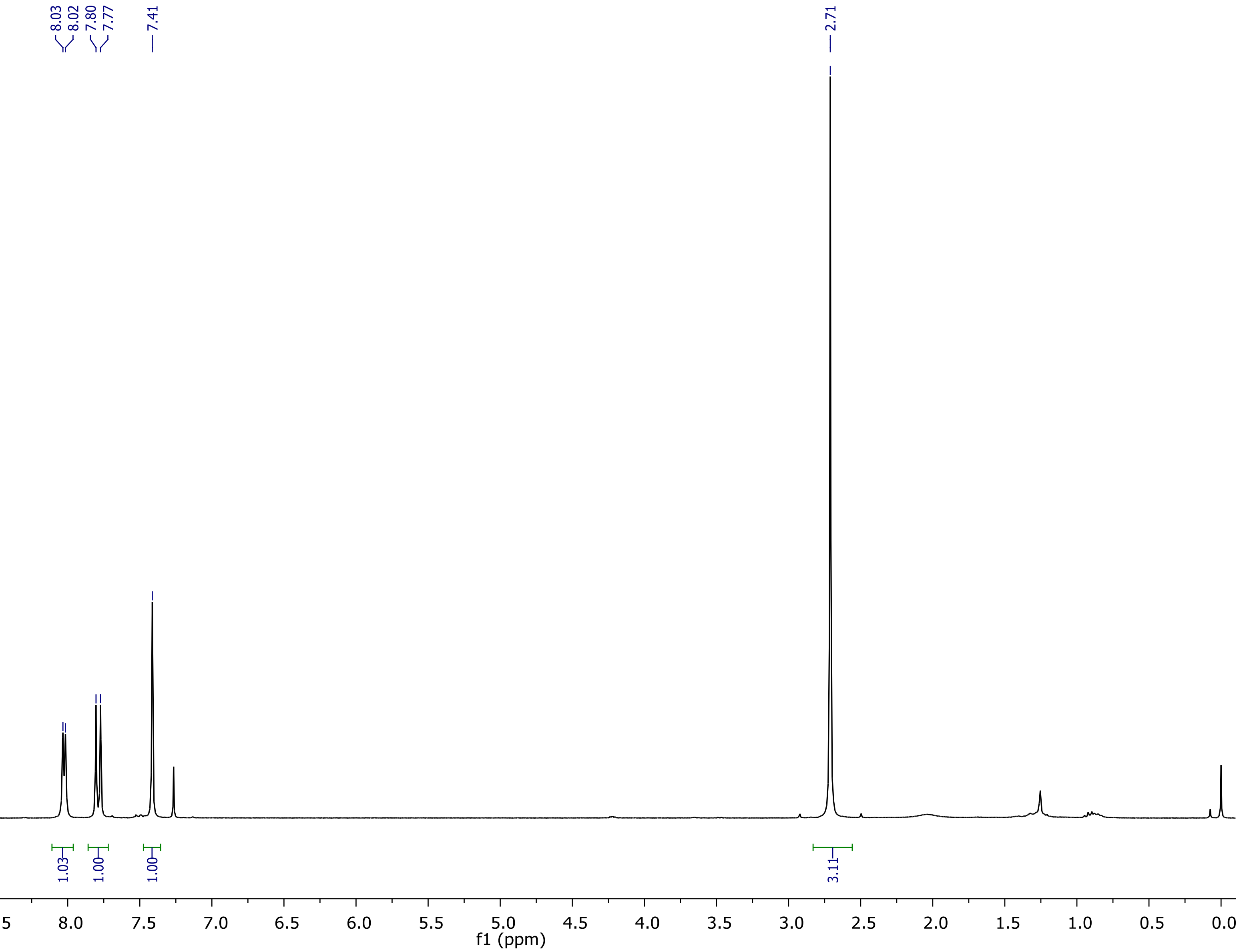

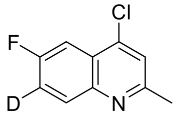

5a

$^{13}C\{^1H\}$ NMR (100 MHz, $CDCl_3$)

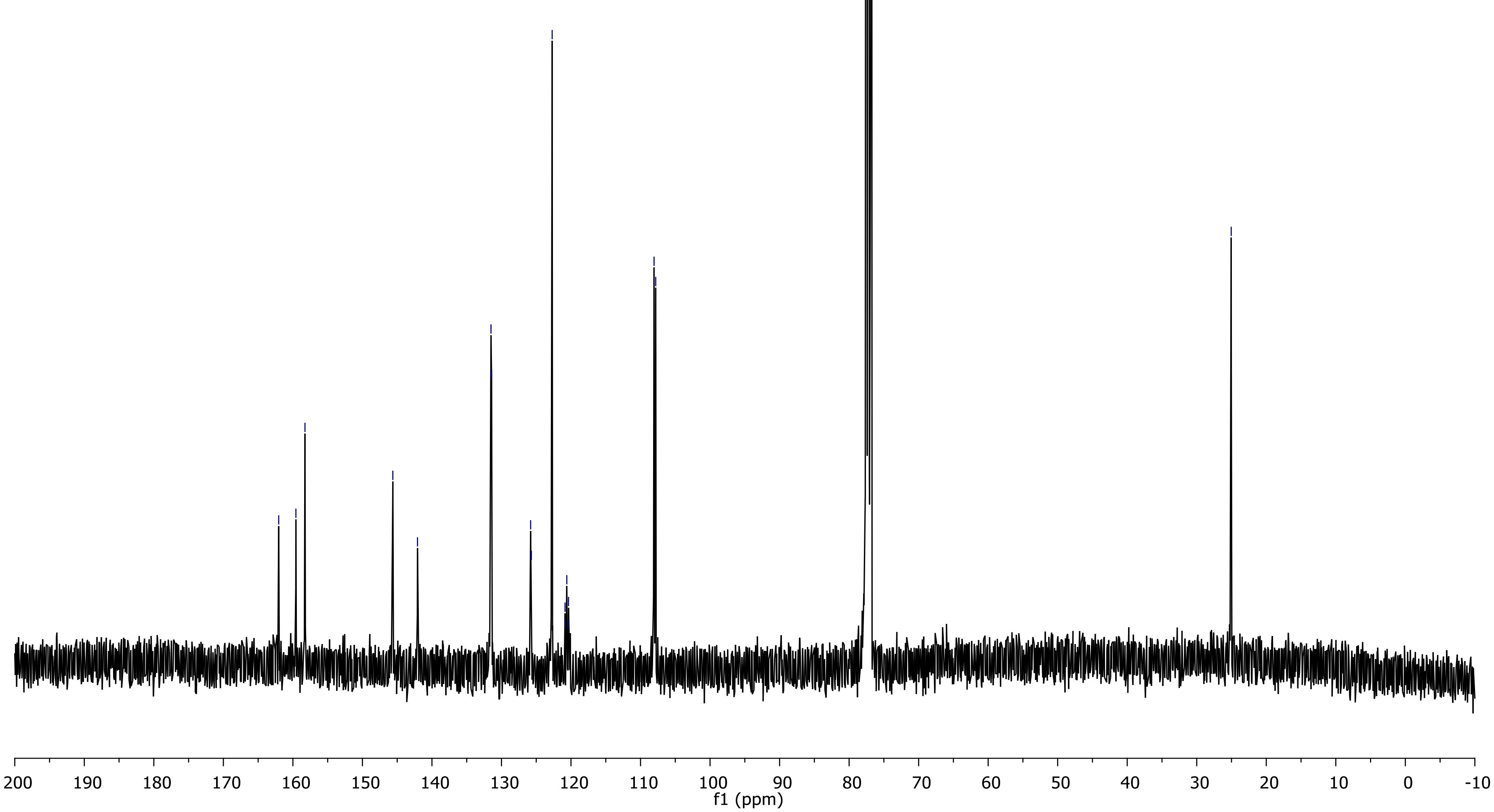

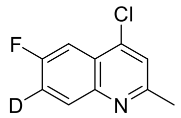

5a

^{19}F NMR (282 MHz, $CDCl_3$)

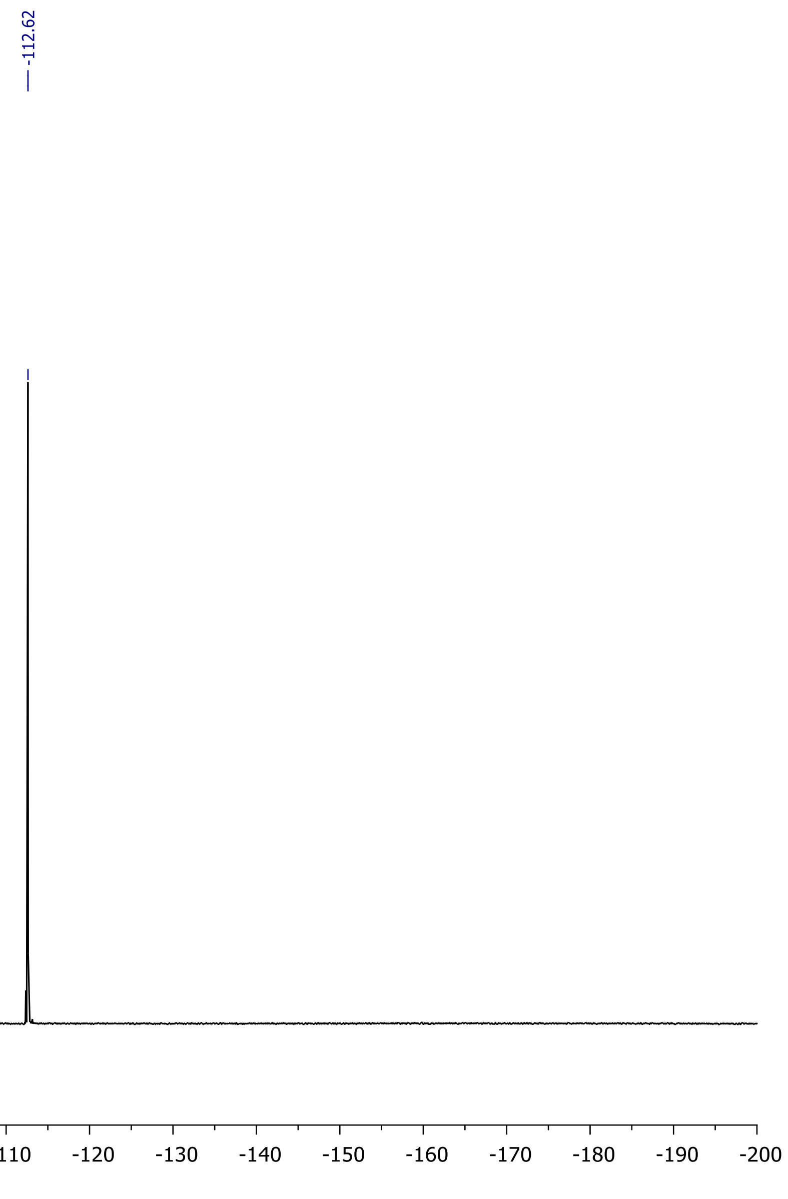

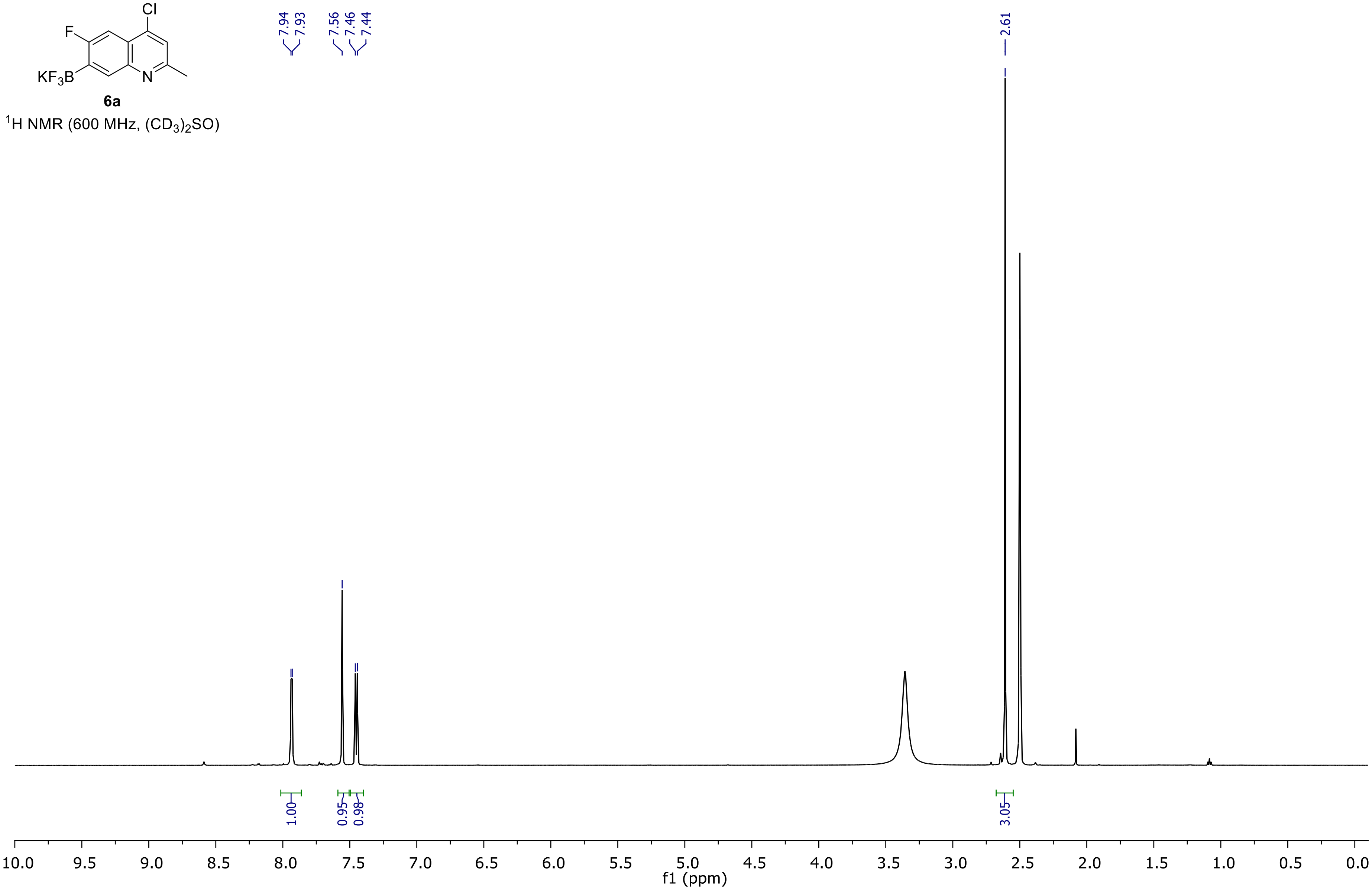

6a

^{1}H NMR (600 MHz, $(CD_3)_2SO$)

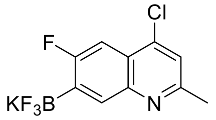

6a

$^{13}C\{^{1}H\}$ NMR (150 MHz, $(CD_3)_2SO$)

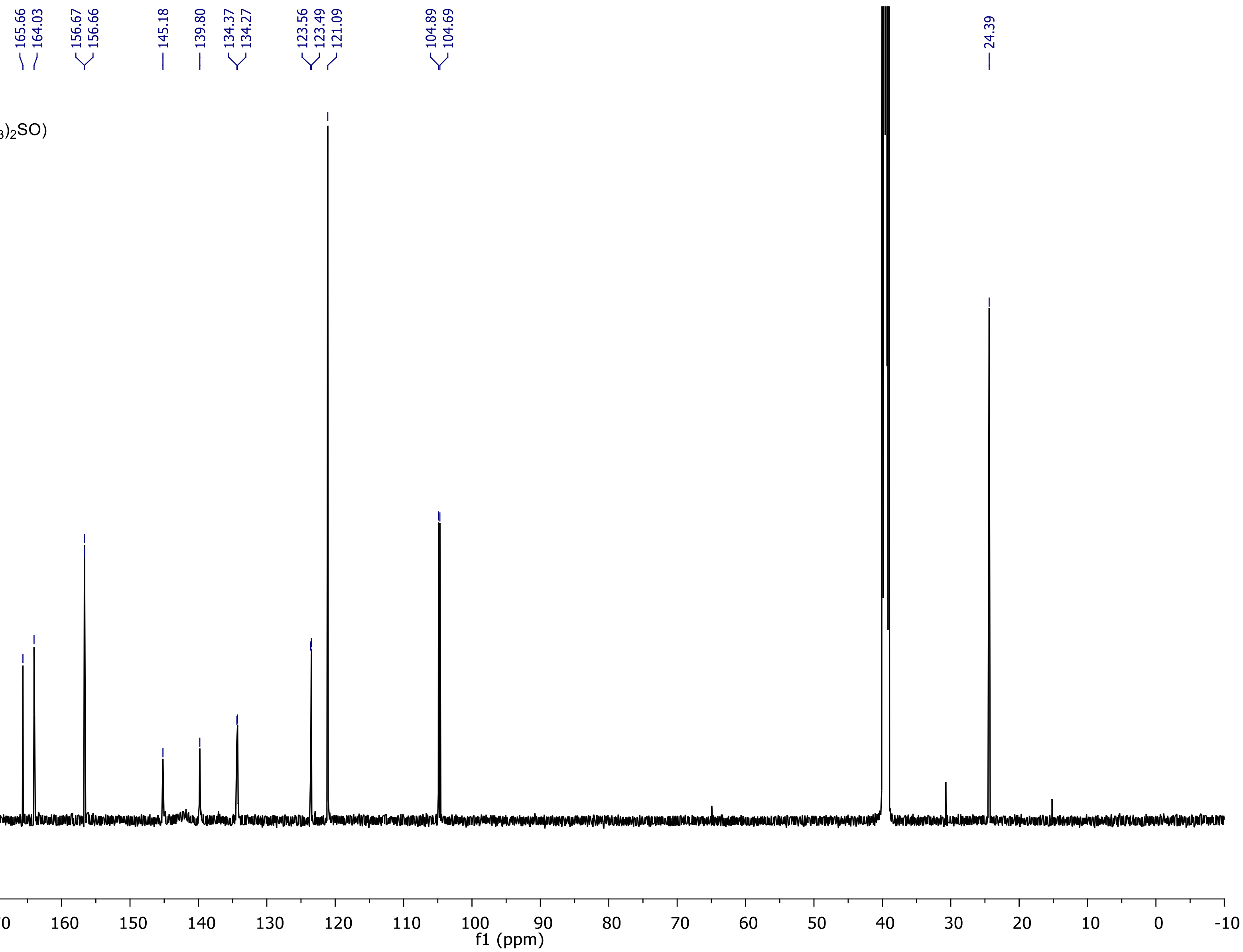

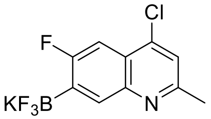

6a

^{19}F NMR (282 MHz, $(CD_3)_2SO$)

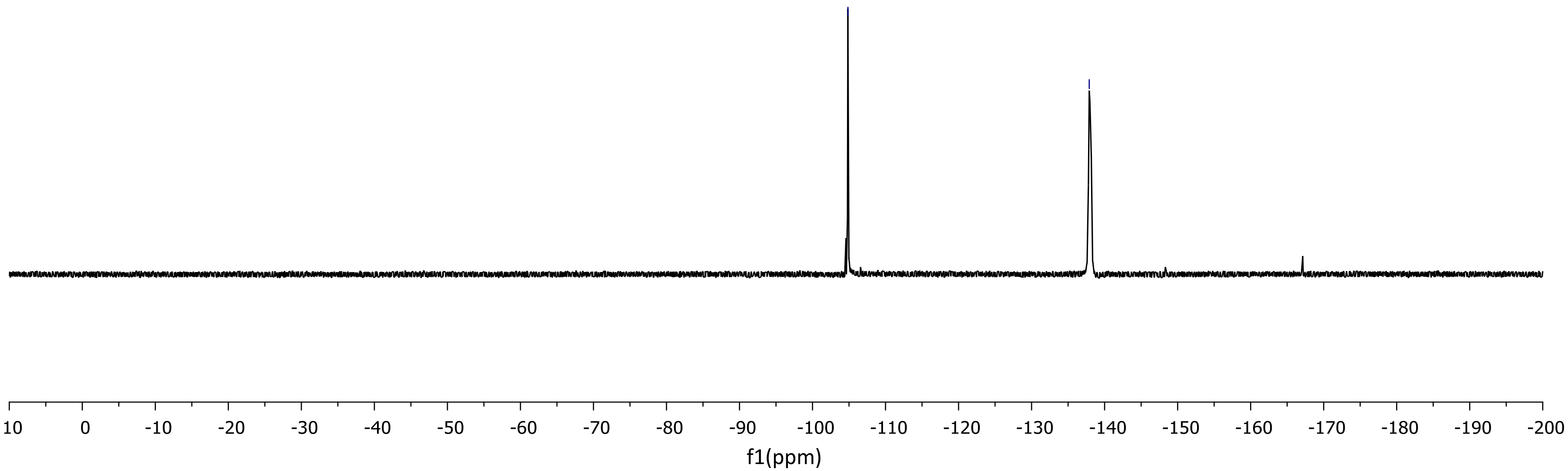

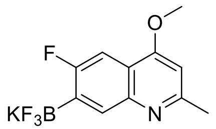

6b

^1H NMR (600 MHz, $(CD_3)_2SO$)

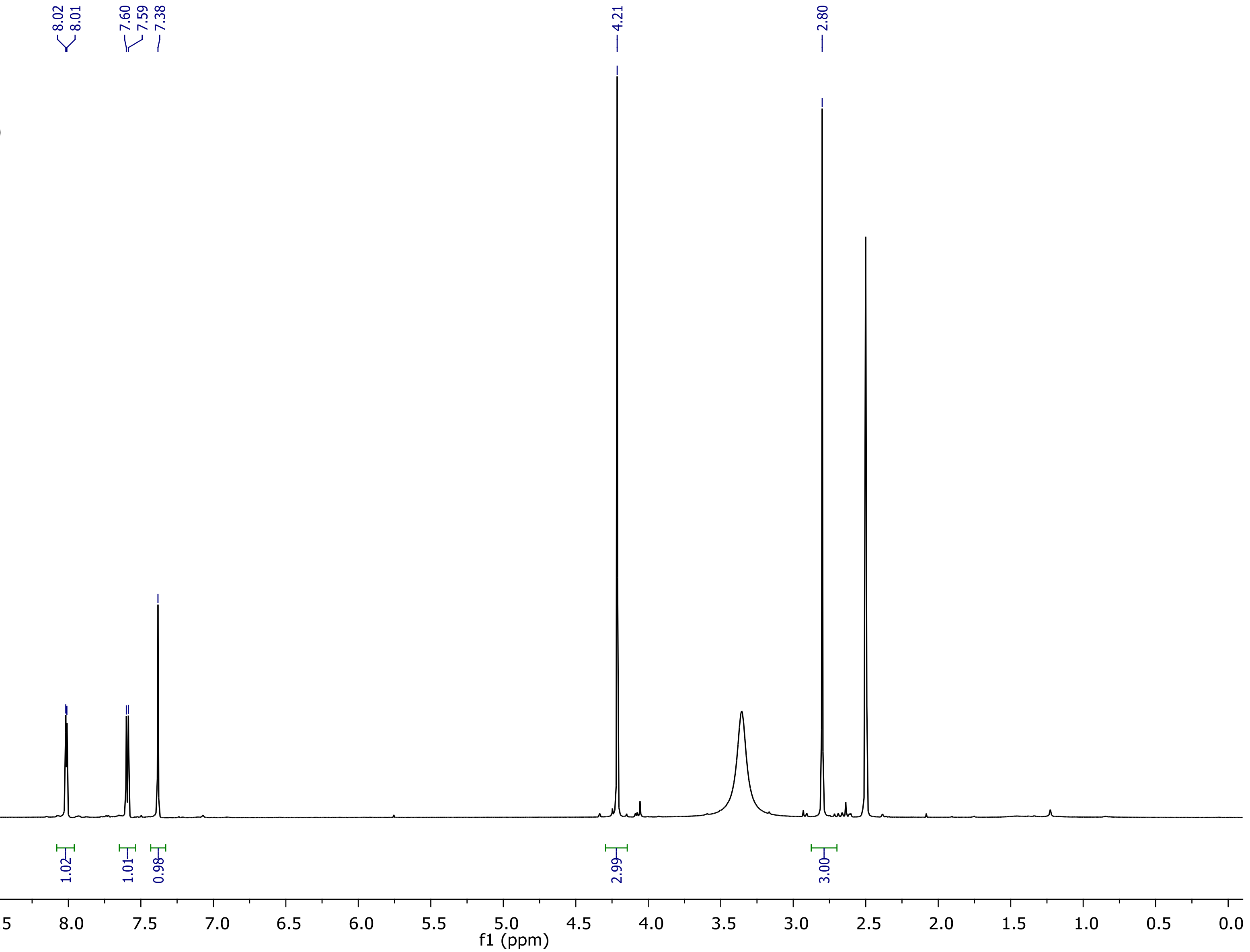

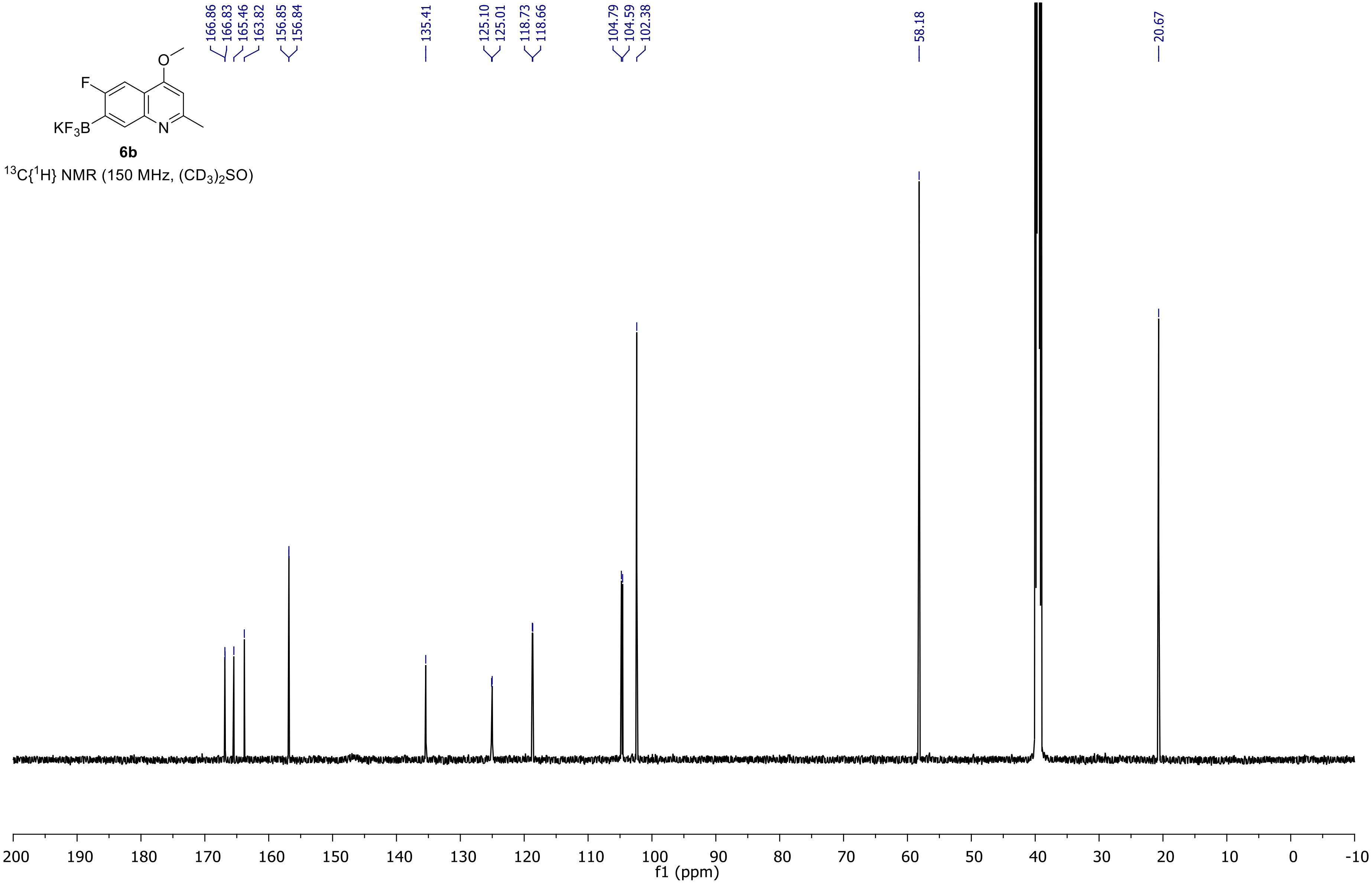

6b

$^{13}C\{^{1}H\}$ NMR (150 MHz, $(CD_3)_2SO$)

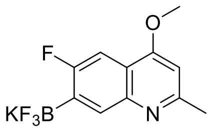

6b

^{19}F NMR (282 MHz, $(CD_3)_2SO$)

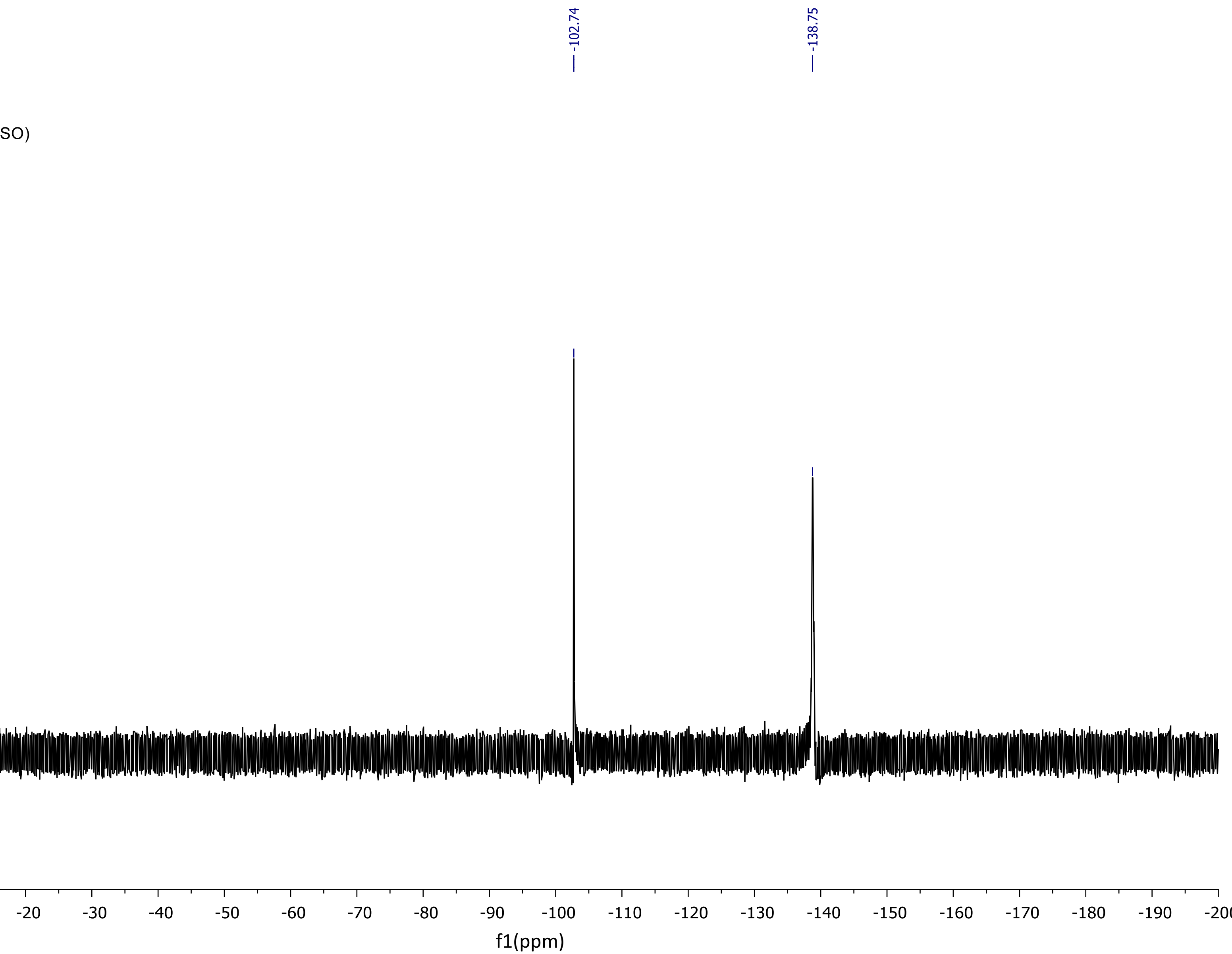

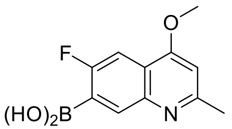

7b

^{1}H NMR (600 MHz, $(CD_3)_2SO$)

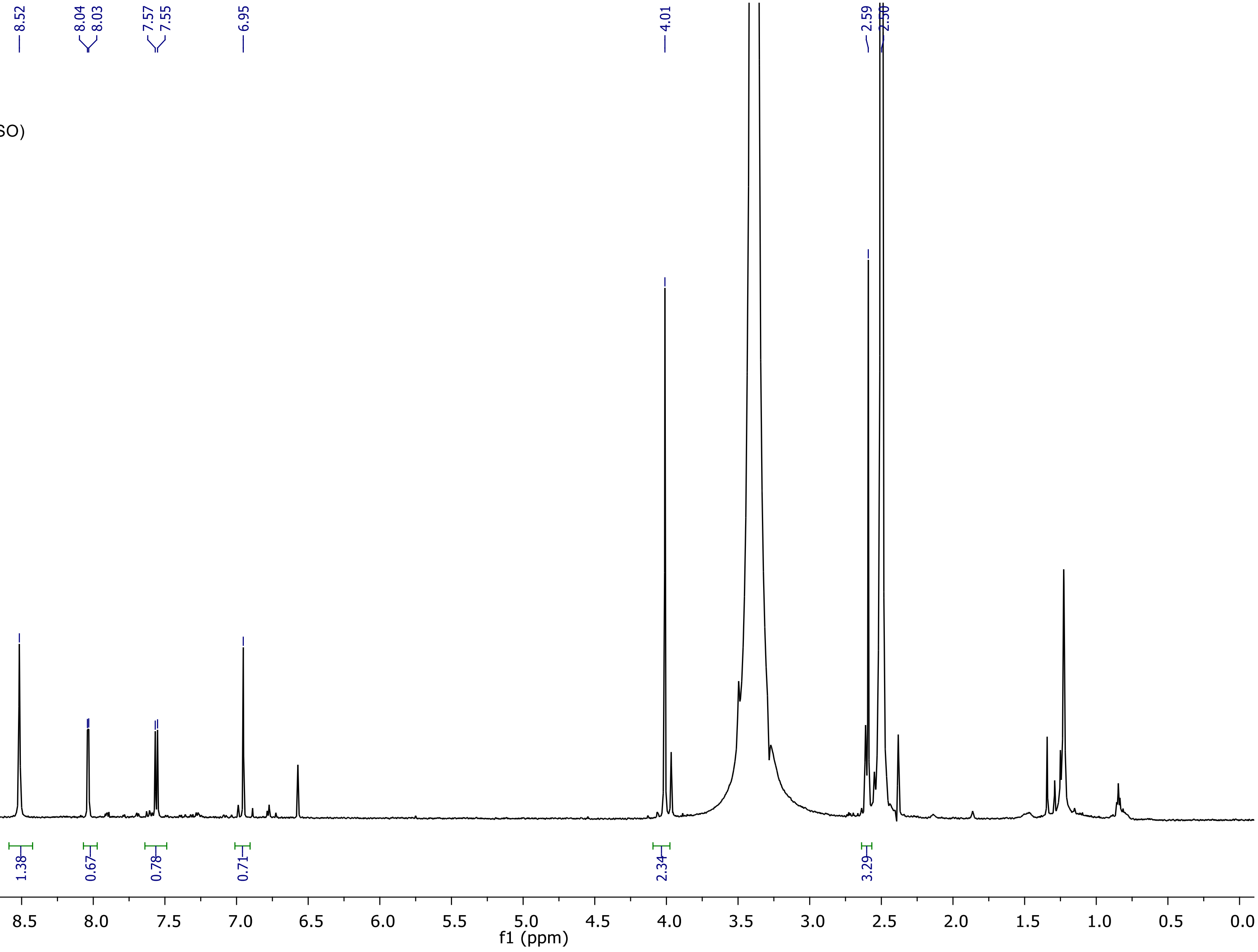

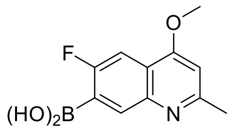

7b

$^{13}C\{^{1}H\}$ NMR (150 MHz, $(CD_3)_2SO$)

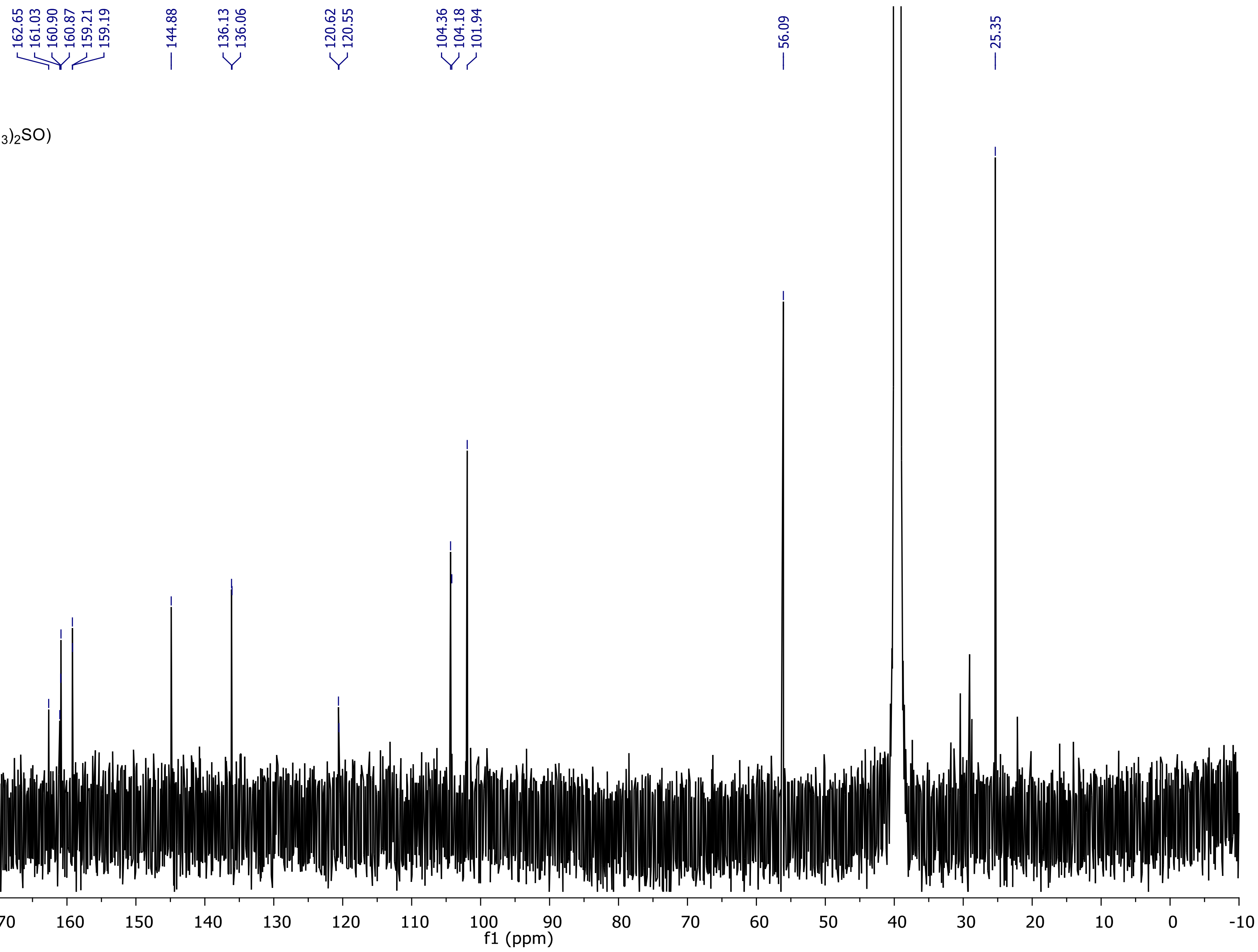

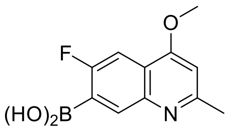

7b

^{19}F NMR (282 MHz, $(CD_3)_2SO$)

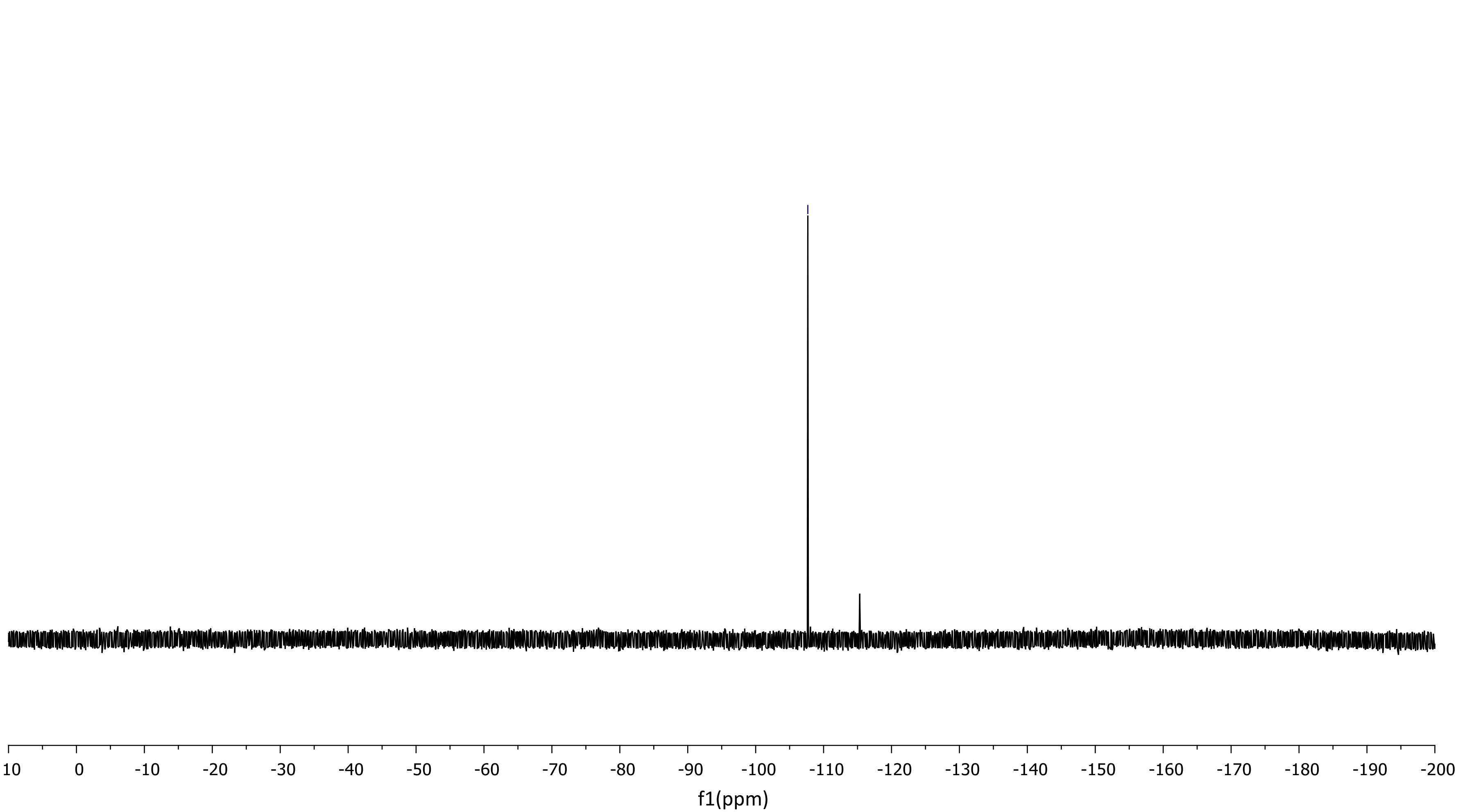

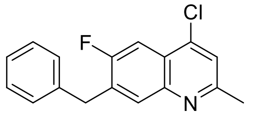

8a

^{1}H NMR (400 MHz, $CDCl_3$)

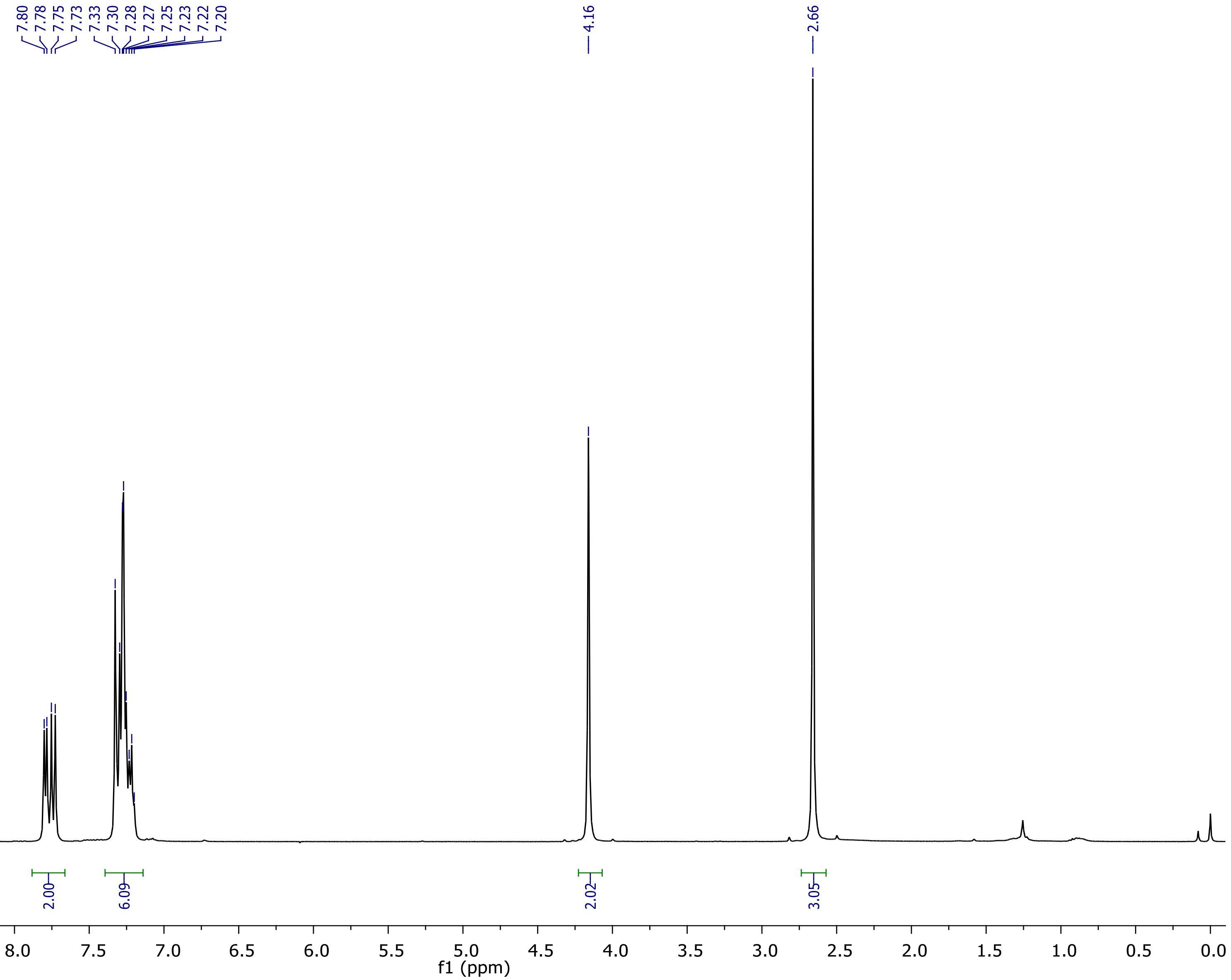

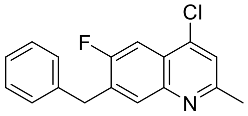

8a

$^{13}C\{^1H\}$ NMR (100 MHz, $CDCl_3$)

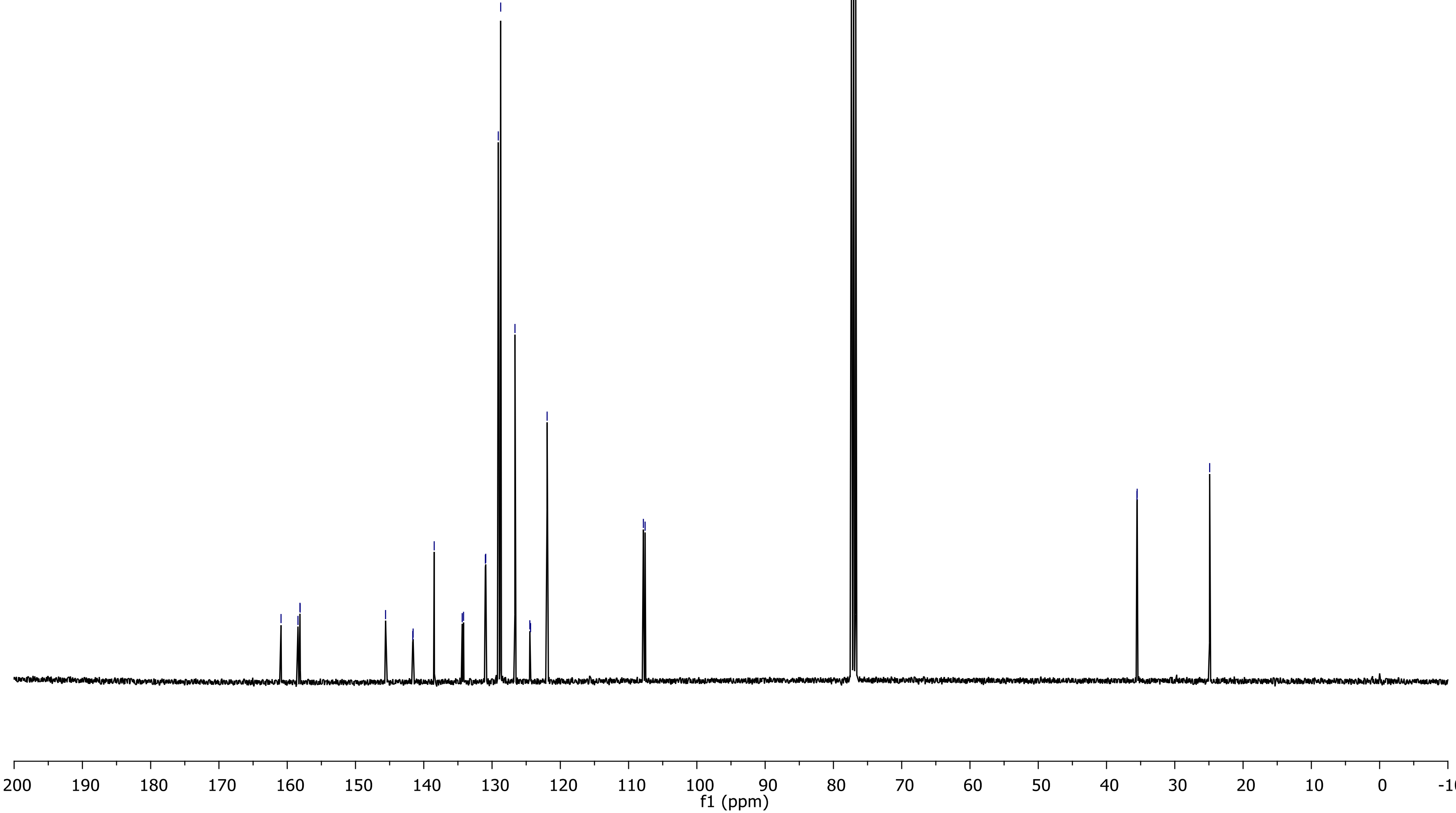

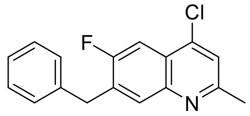

8a

^{19}F NMR (376 MHz, $CDCl_3$)

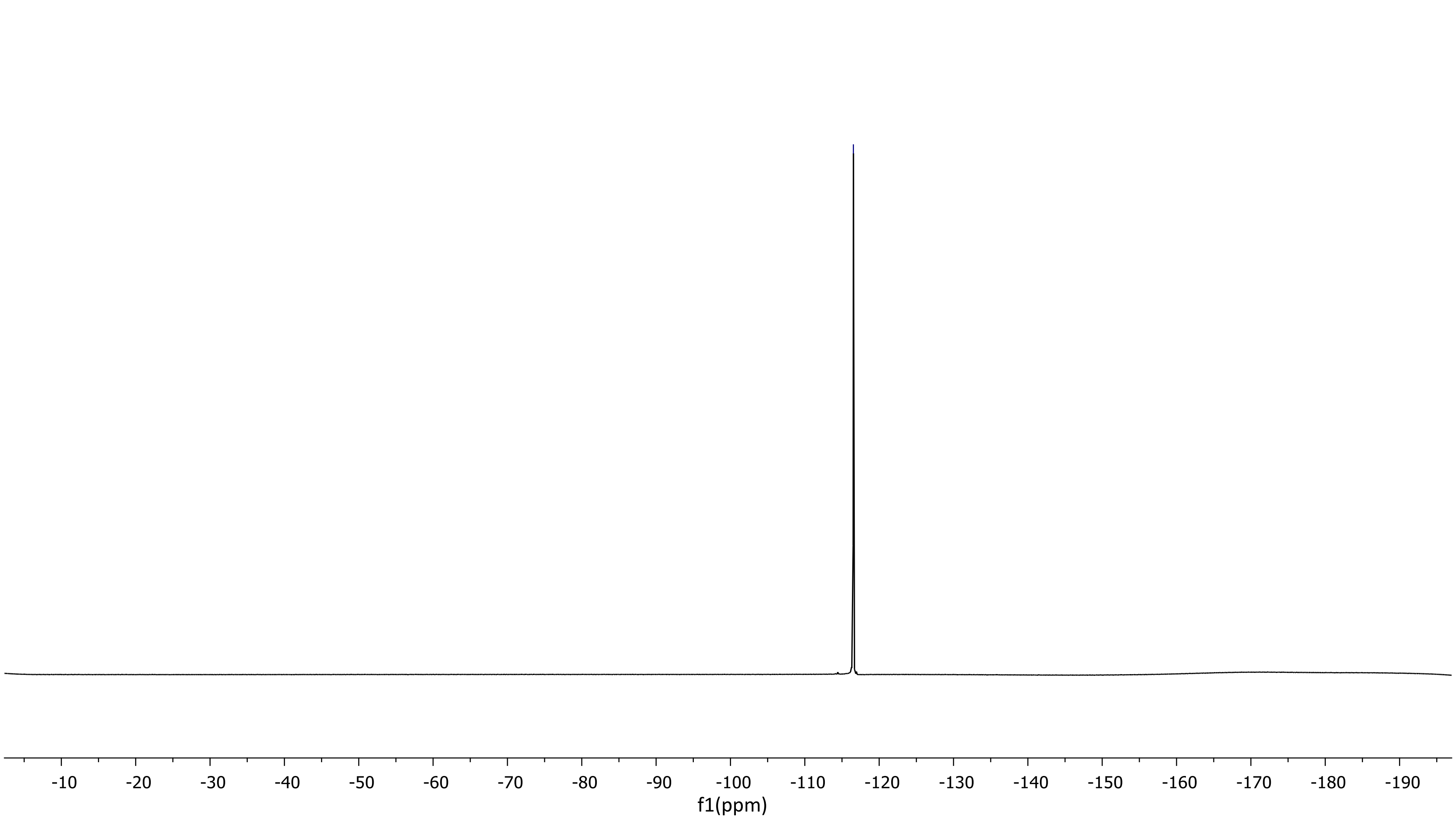

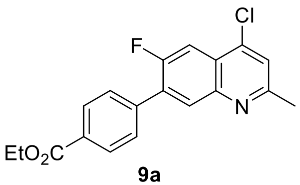

9a

^{1}H NMR (300 MHz, $CDCl_3$)

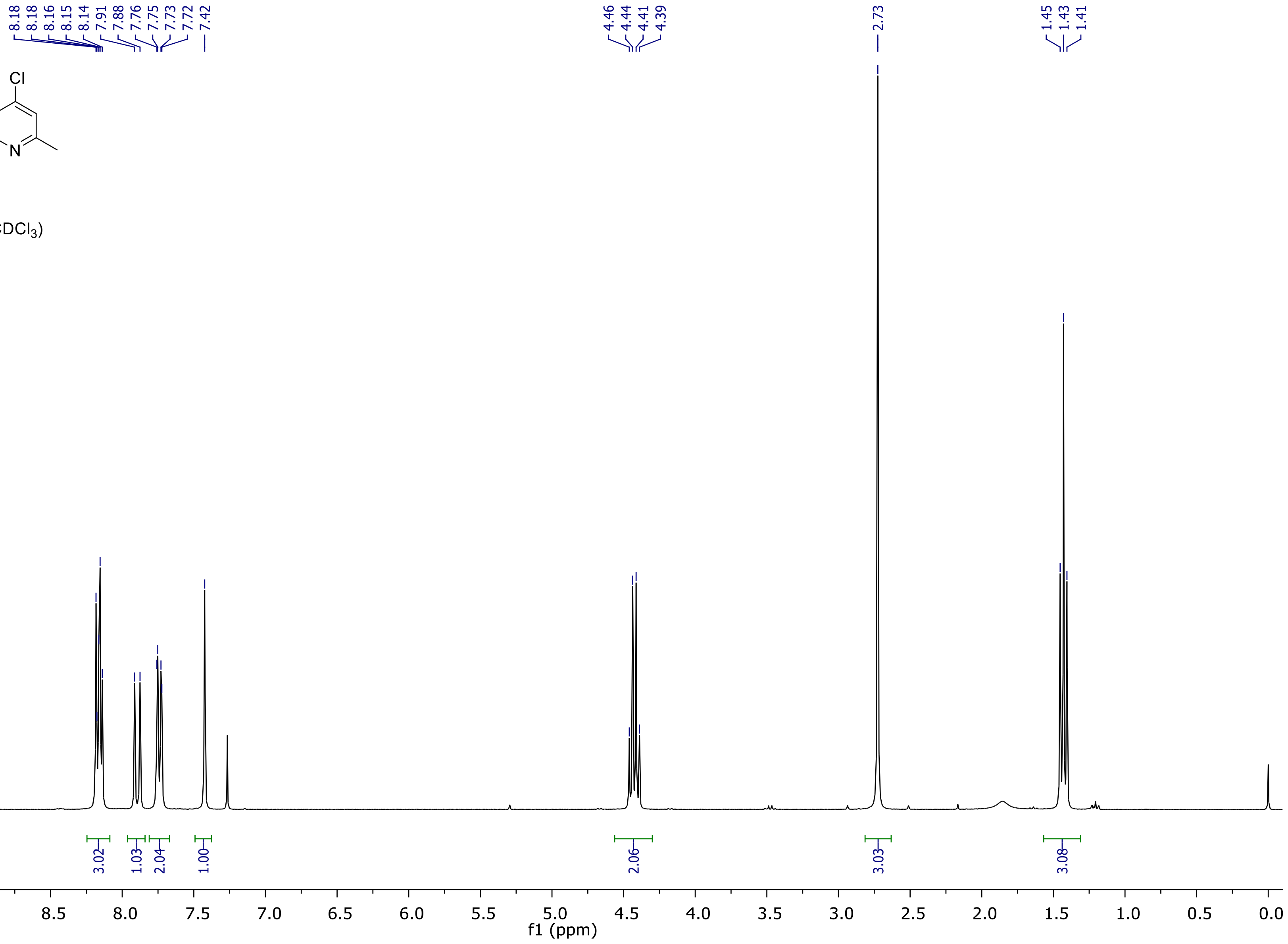

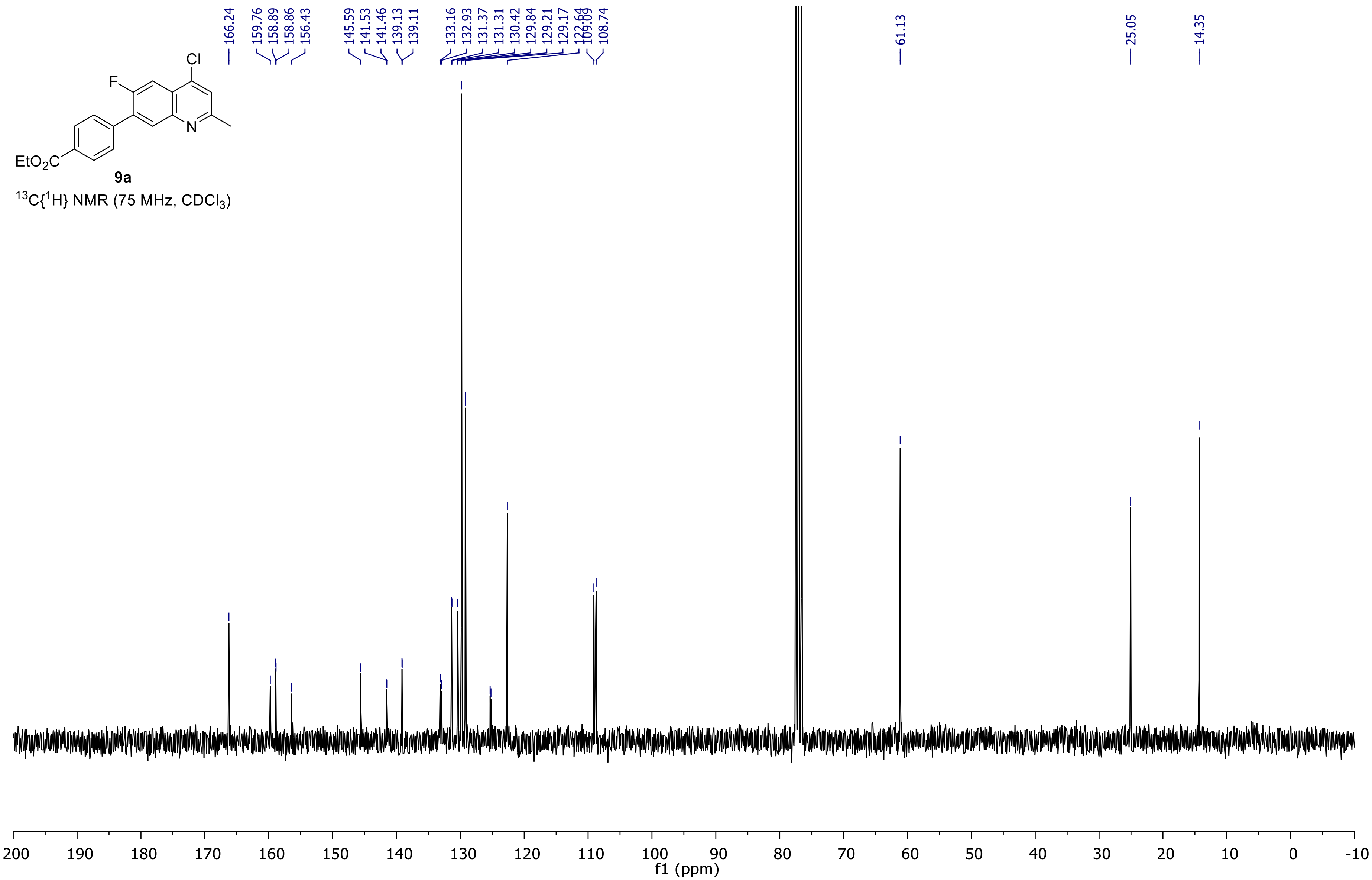

9a
$^{13}C\{^1H\}$ NMR (75 MHz, CDCl3)
166.24
159.76
158.89
158.86
156.43
145.59
141.53
141.46
139.13
139.11
133.16
132.93
131.37
131.31
130.42
129.84
129.21
129.17
122.64
109.09
108.74
61.13
25.05
14.35
f1 (ppm)

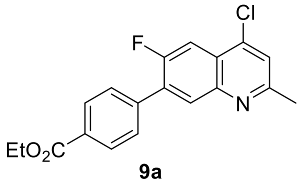

9a

^{19}F NMR (282 MHz, $CDCl_3$)

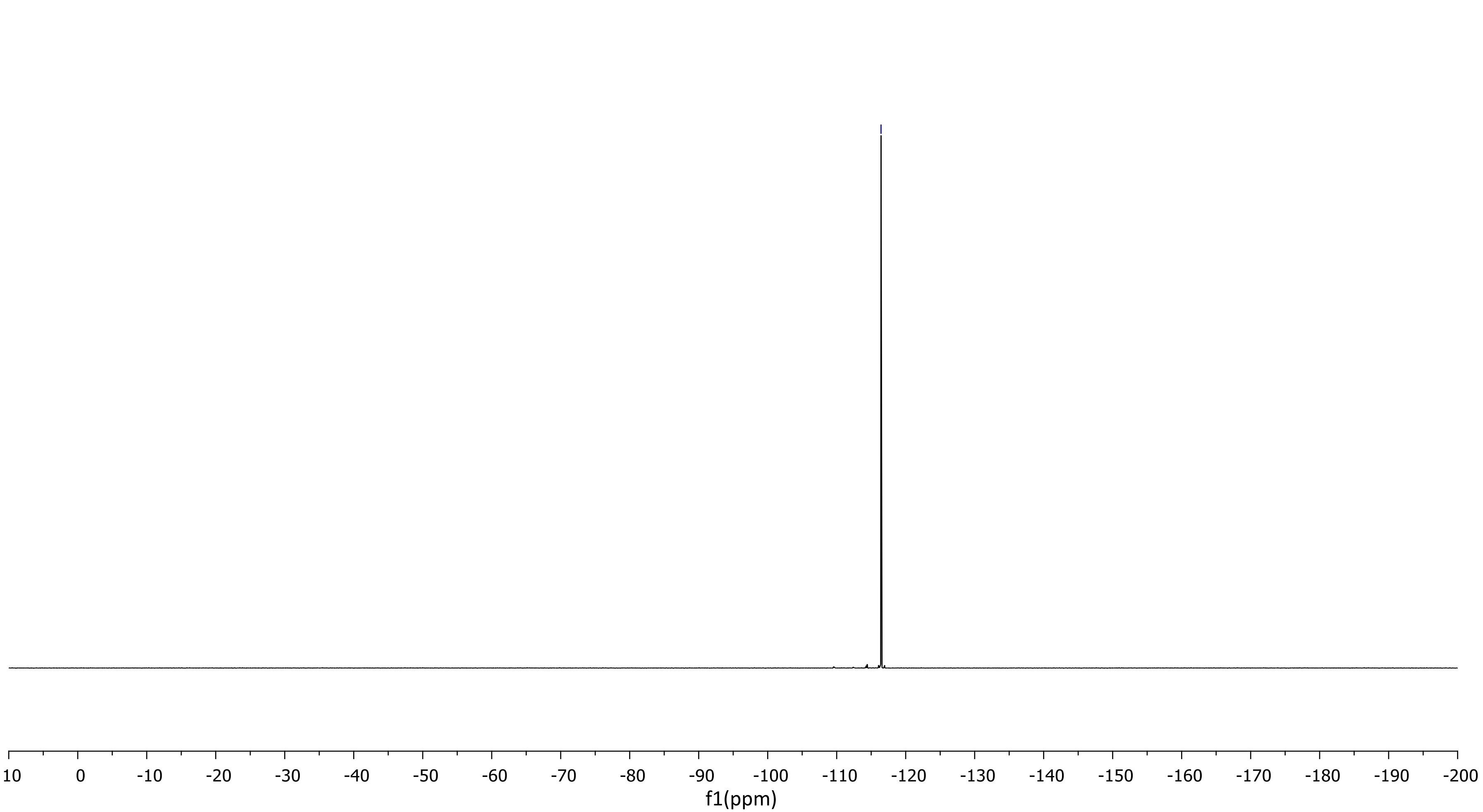

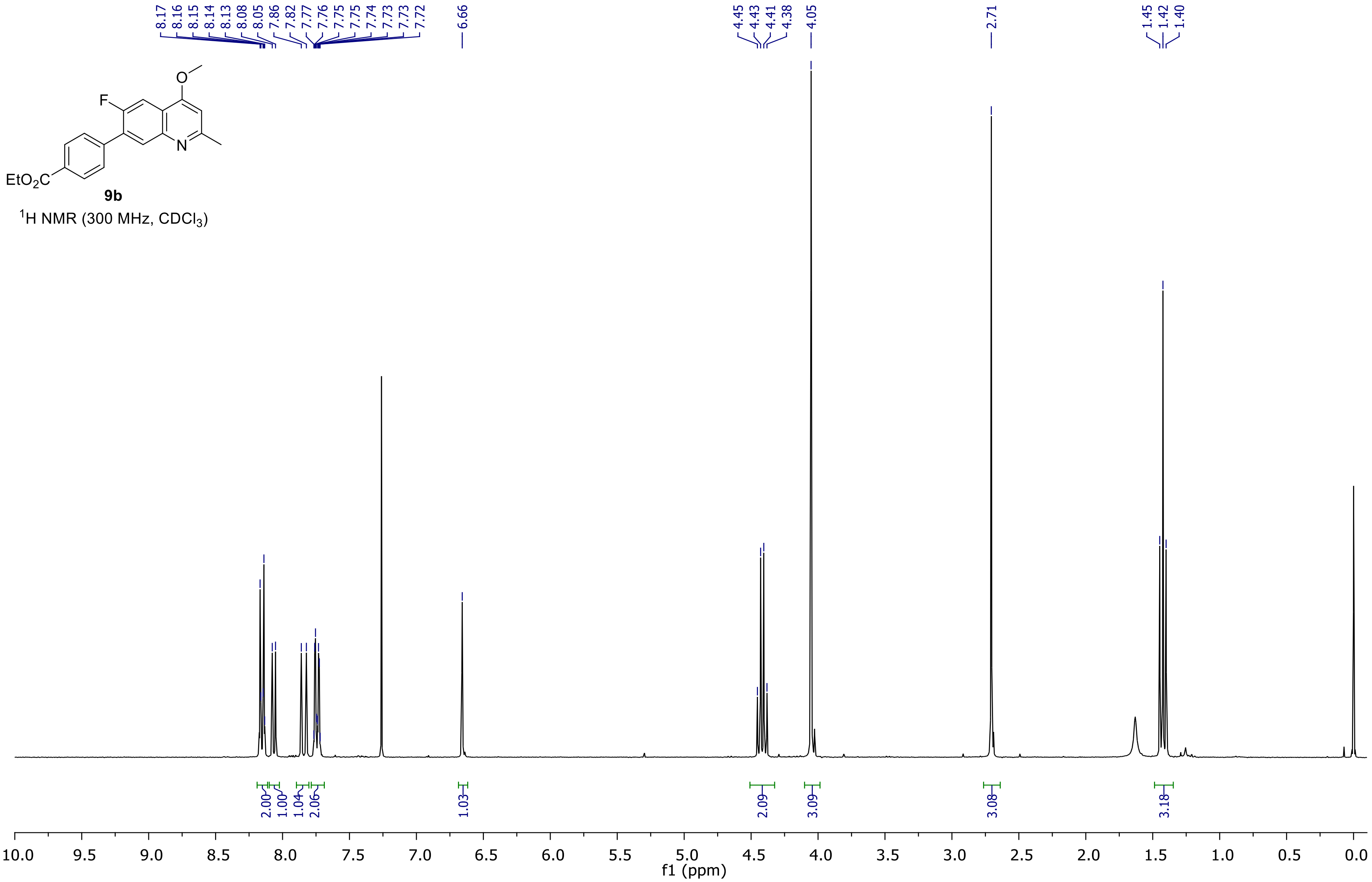

9b

^{1}H NMR (300 MHz, $CDCl_3$)

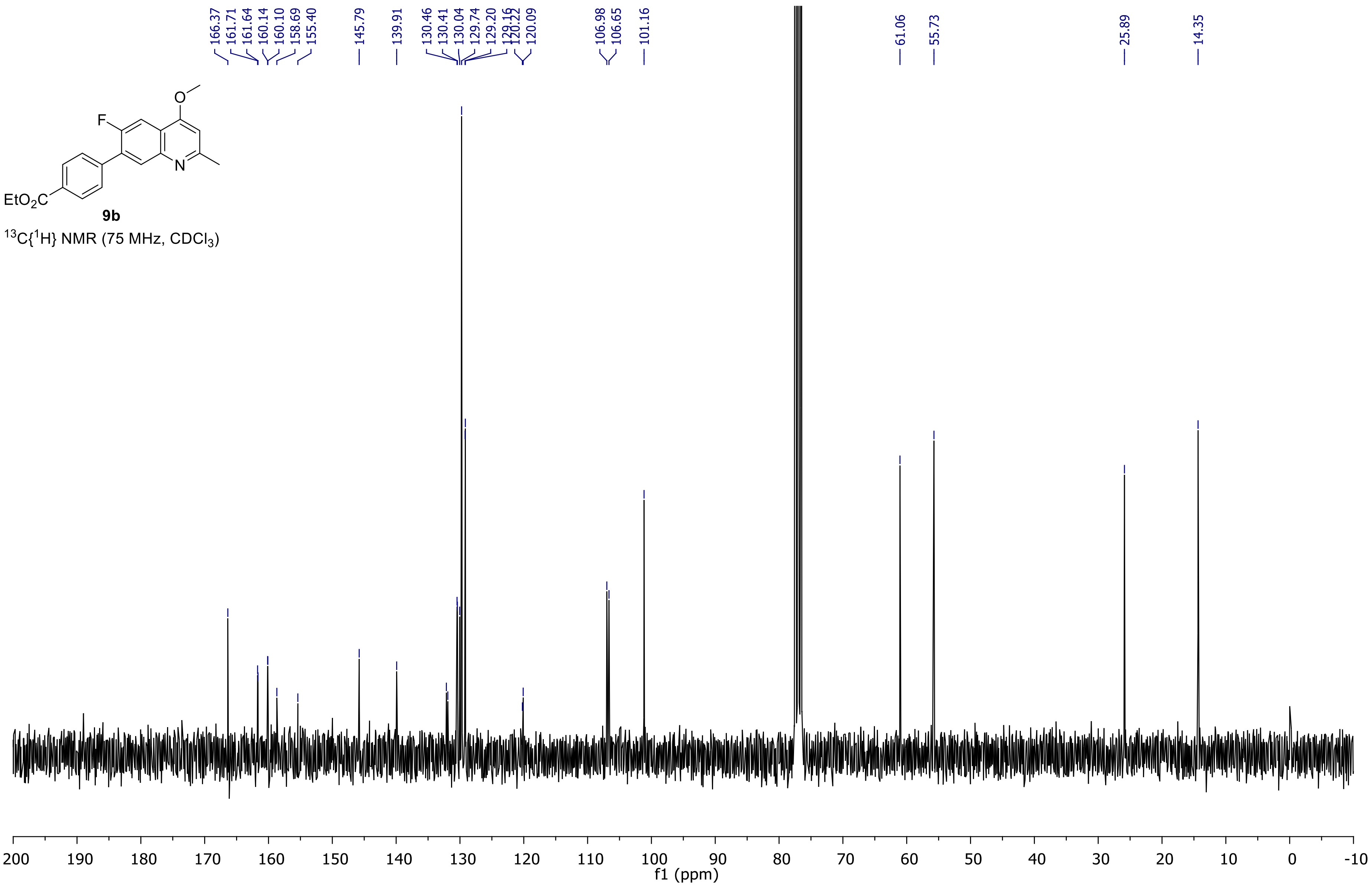

$^{13}C\{^1H\}$ NMR (75 MHz, $CDCl_3$)

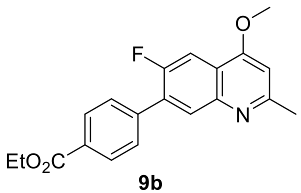

9b

^{19}F NMR (282 MHz, $CDCl_3$)

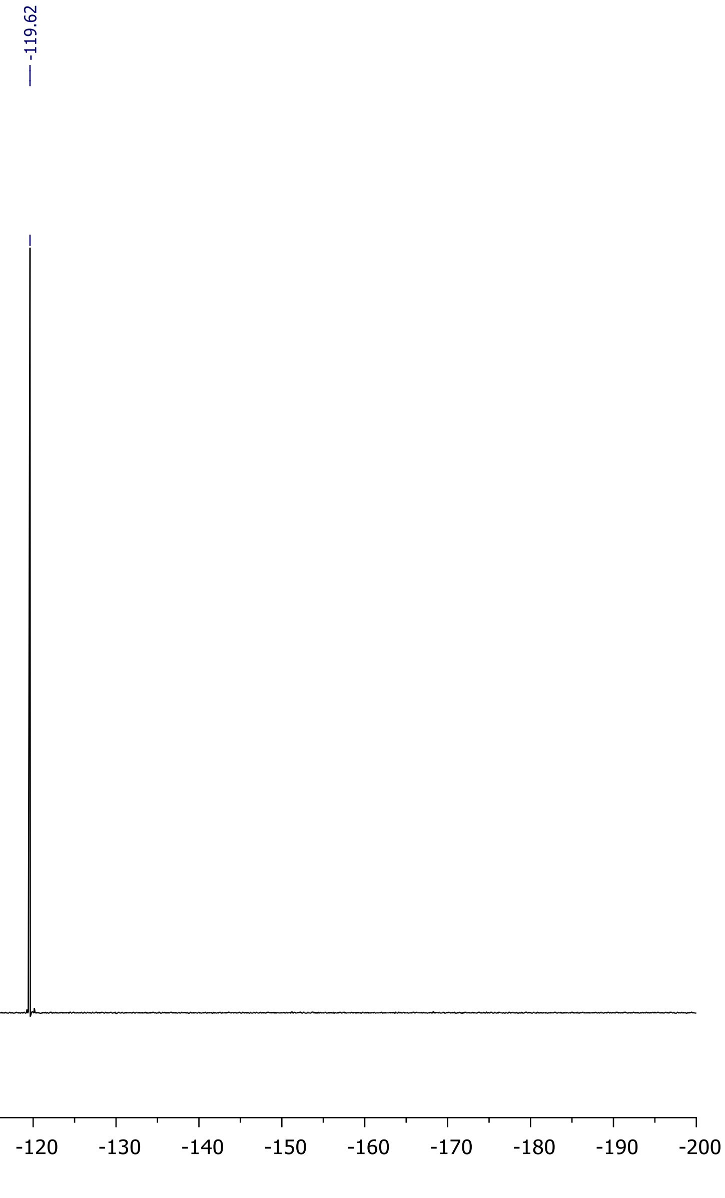

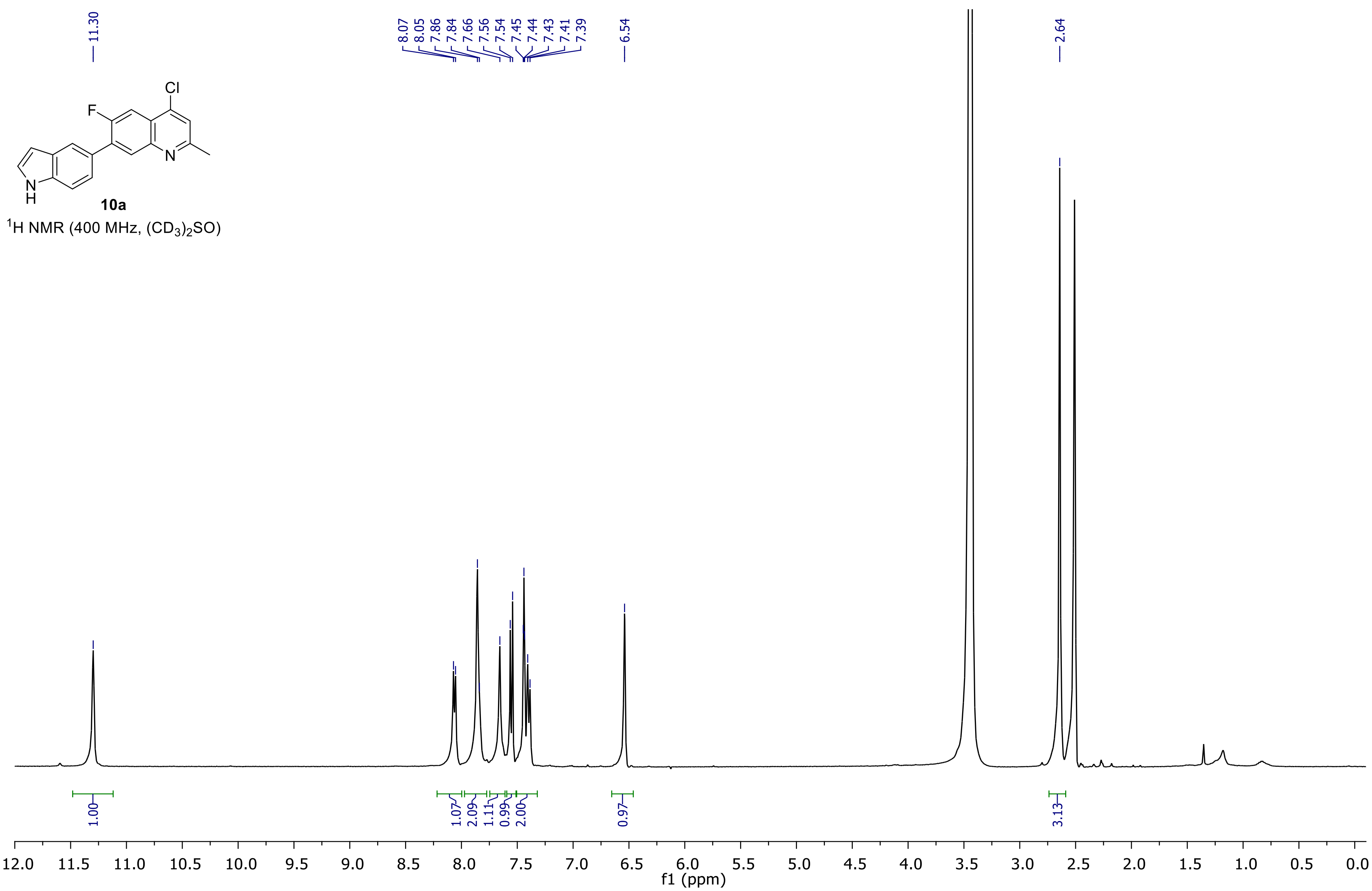
11.30
8.07 8.05 7.86 7.84 7.66 7.56 7.54 7.45 7.44 7.43 7.41 7.39
6.54
2.64
Cl
F
N
N
H
10a
^{1}H NMR (400 MHz, $(CD_3)_2SO$)
1.00
1.07 2.09 1.11 0.99 2.00
0.97
3.13
12.0 11.5 11.0 10.5 10.0 9.5 9.0 8.5 8.0 7.5 7.0 6.5 6.0 5.5 5.0 4.5 4.0 3.5 3.0 2.5 2.0 1.5 1.0 0.5 0.0
f1 (ppm)

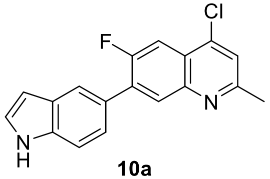

10a

$^{13}C\{^1H\}$ NMR (100 MHz, $(CD_3)_2SO$)

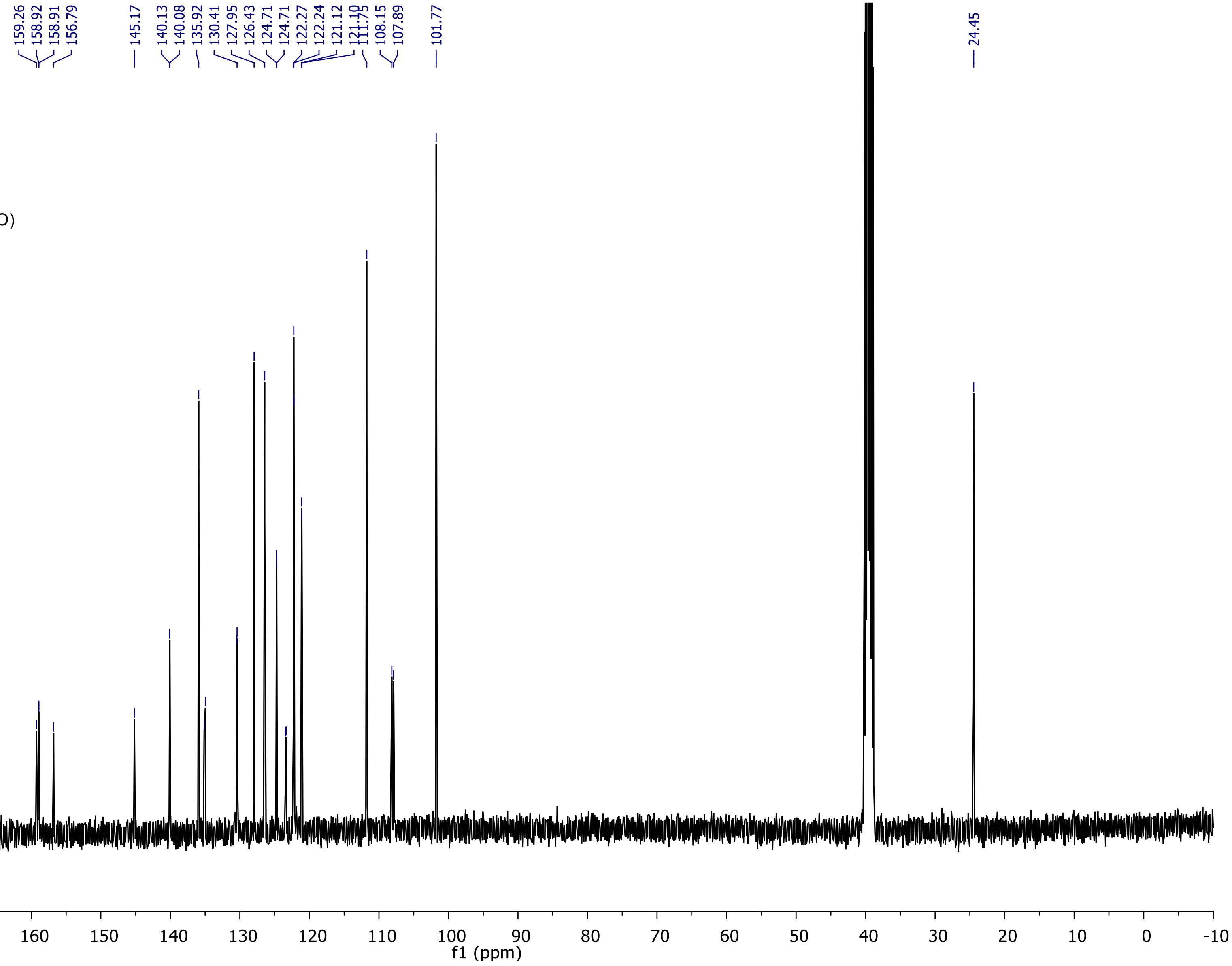

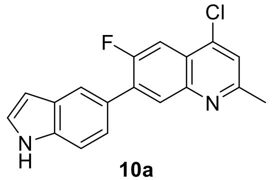

10a

^{19}F NMR (282 MHz, $(CD_3)_2SO$)

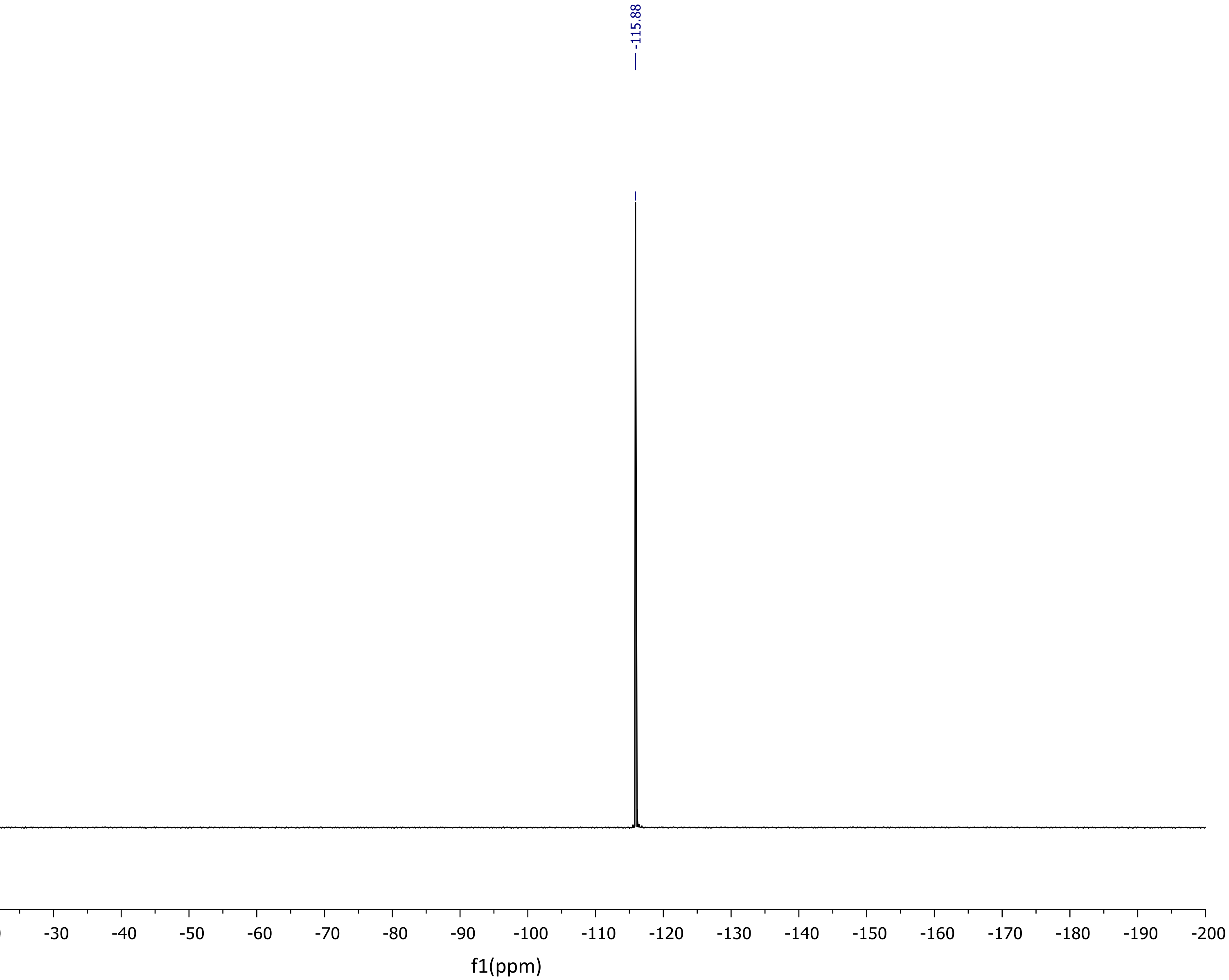

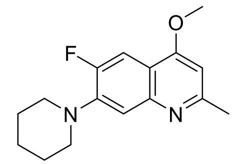

11b

^{1}H NMR (500 MHz, $CDCl_3$)

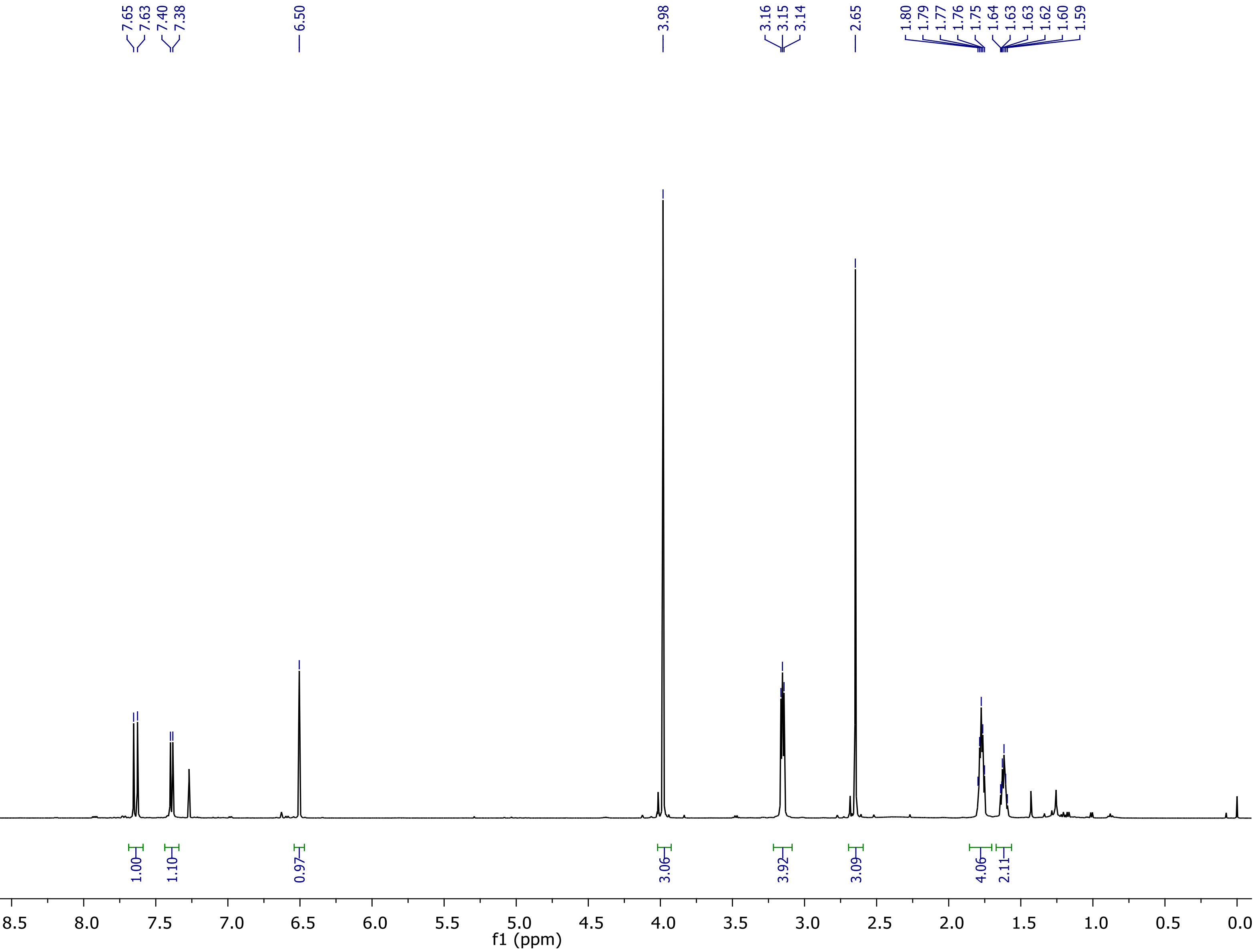

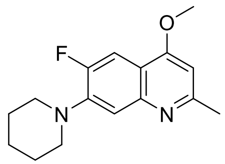

11b

$^{13}C\{^1H\}$ NMR (125 MHz, $CDCl_3$)

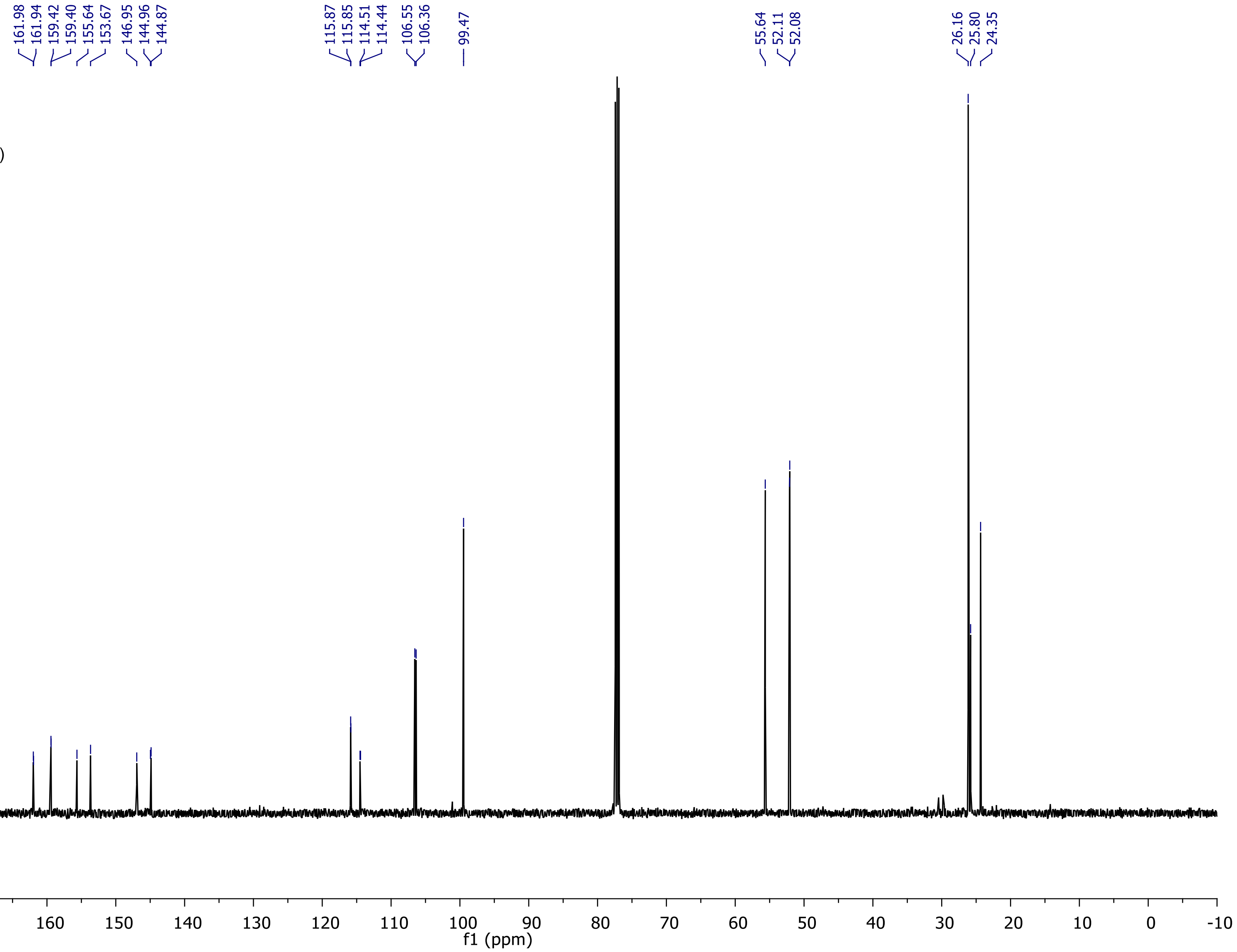

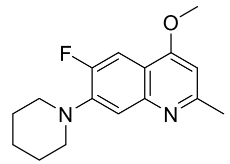

11b

^{19}F NMR (282 MHz, $CDCl_3$)

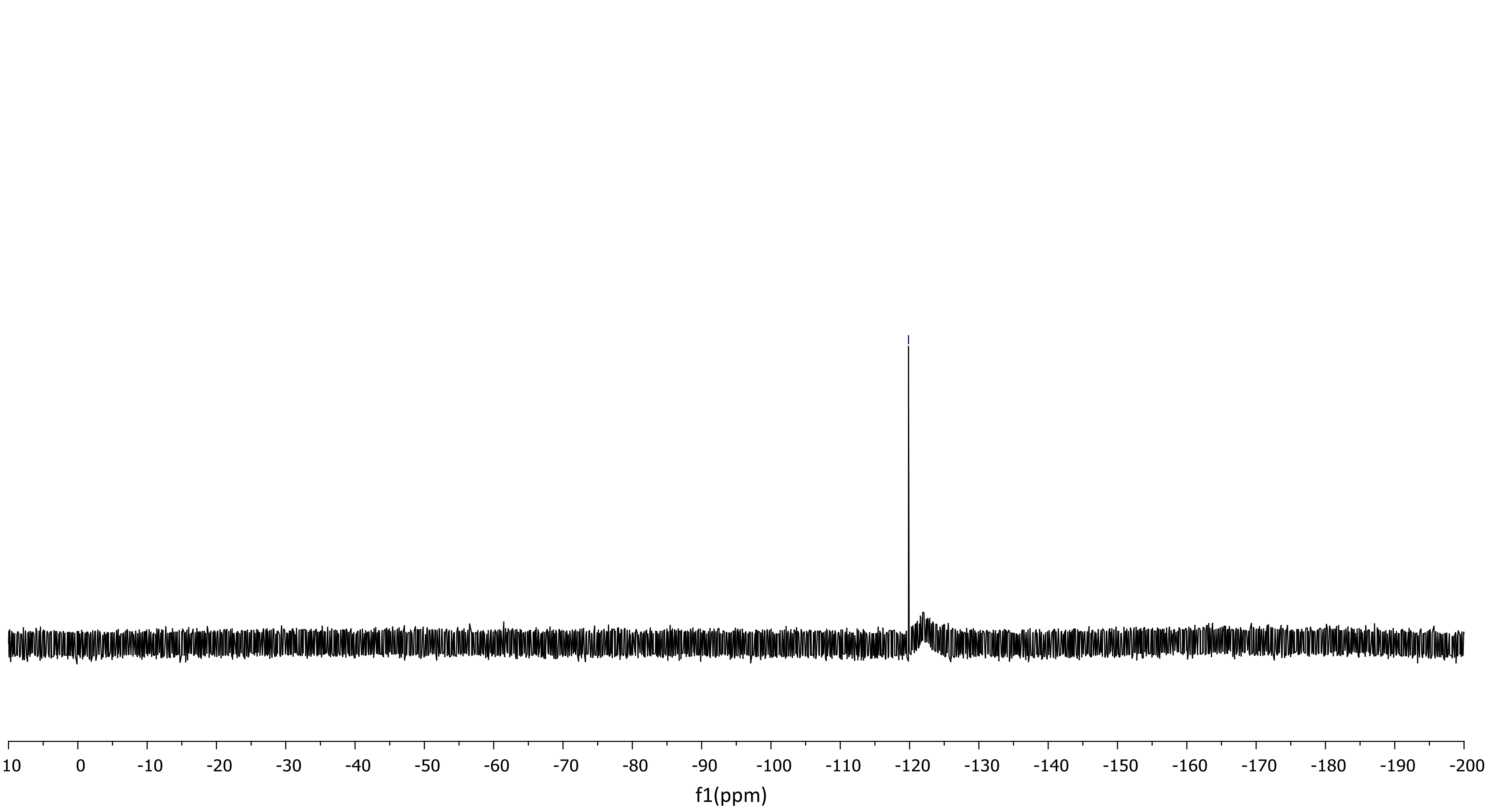

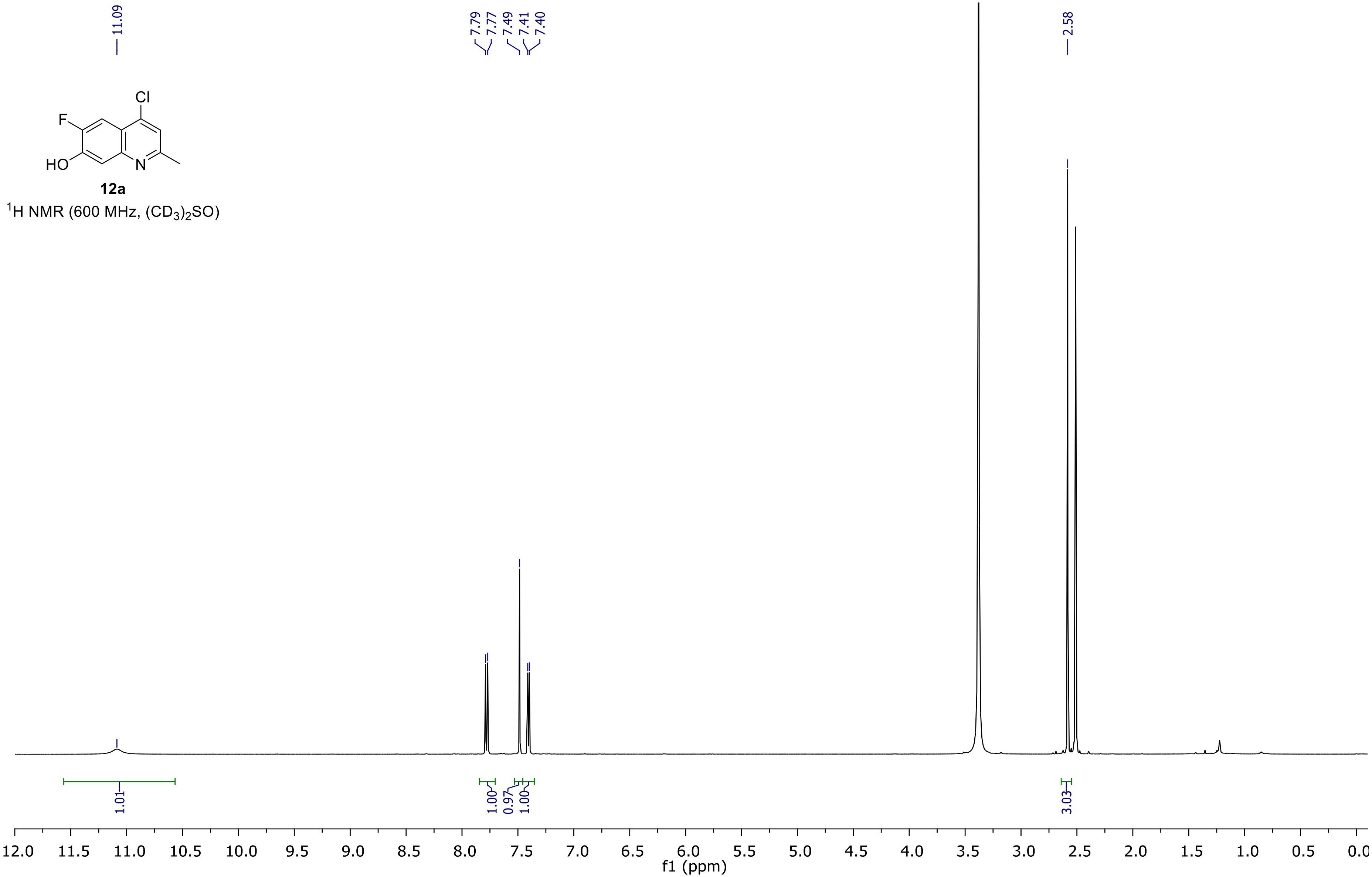

12a

^{1}H NMR (600 MHz, $(CD_3)_2SO$)

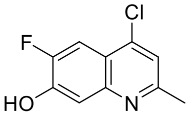

12a

$^{13}C\{^{1}H\}$ NMR (150 MHz, $(CD_3)_2SO$)

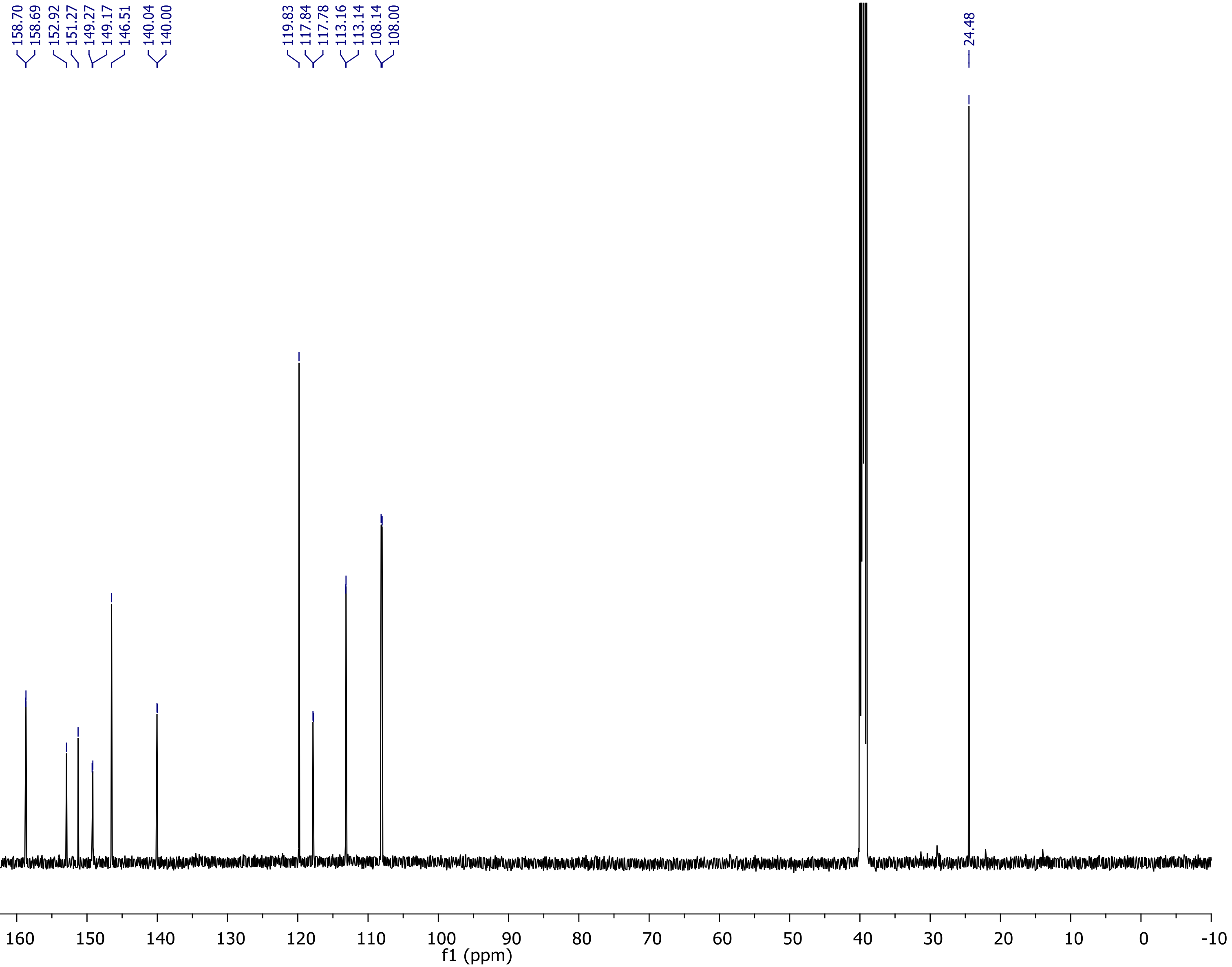

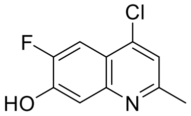

12a

^{19}F NMR (282 MHz, $(CD_3)_2SO$)

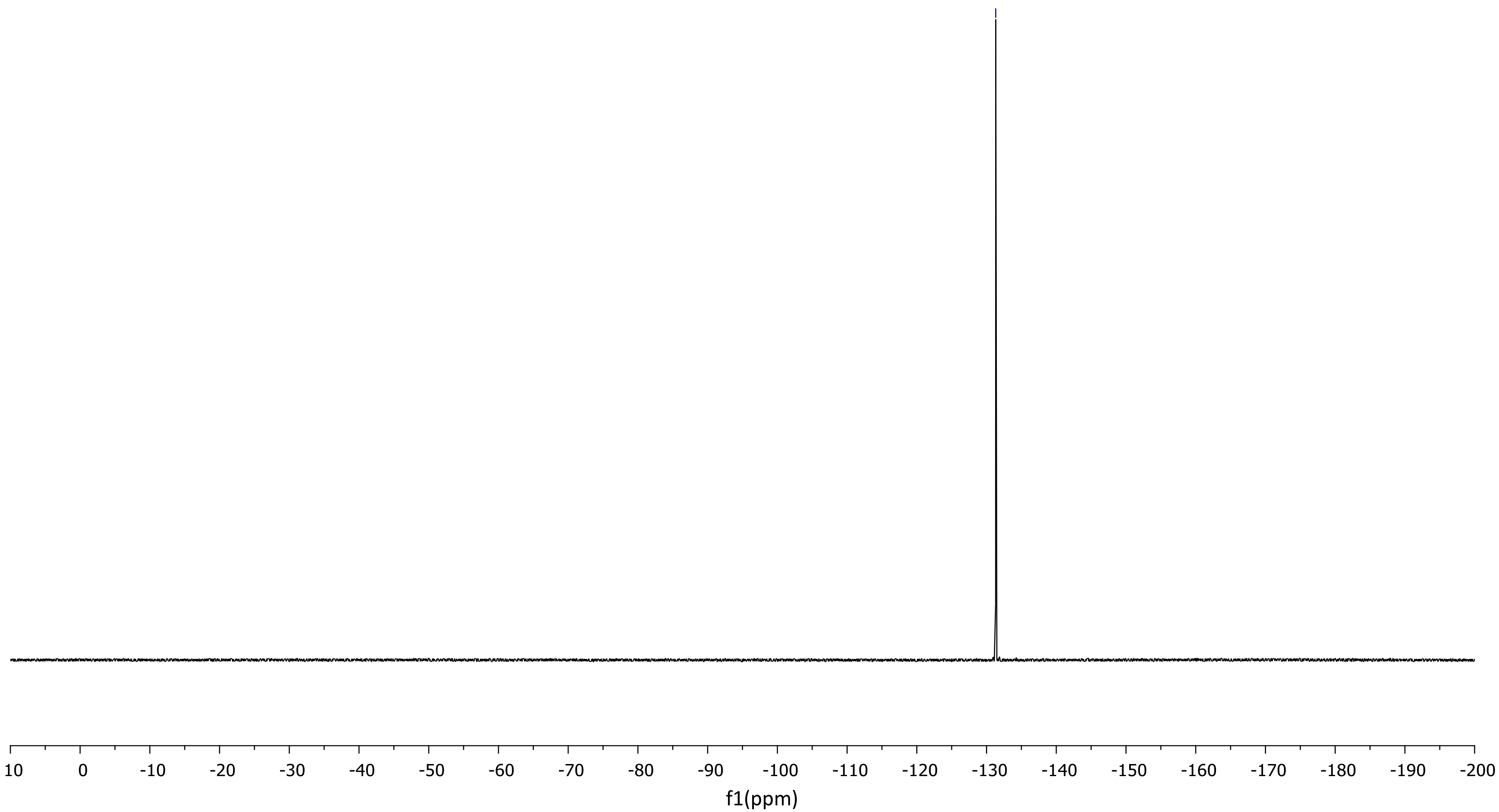

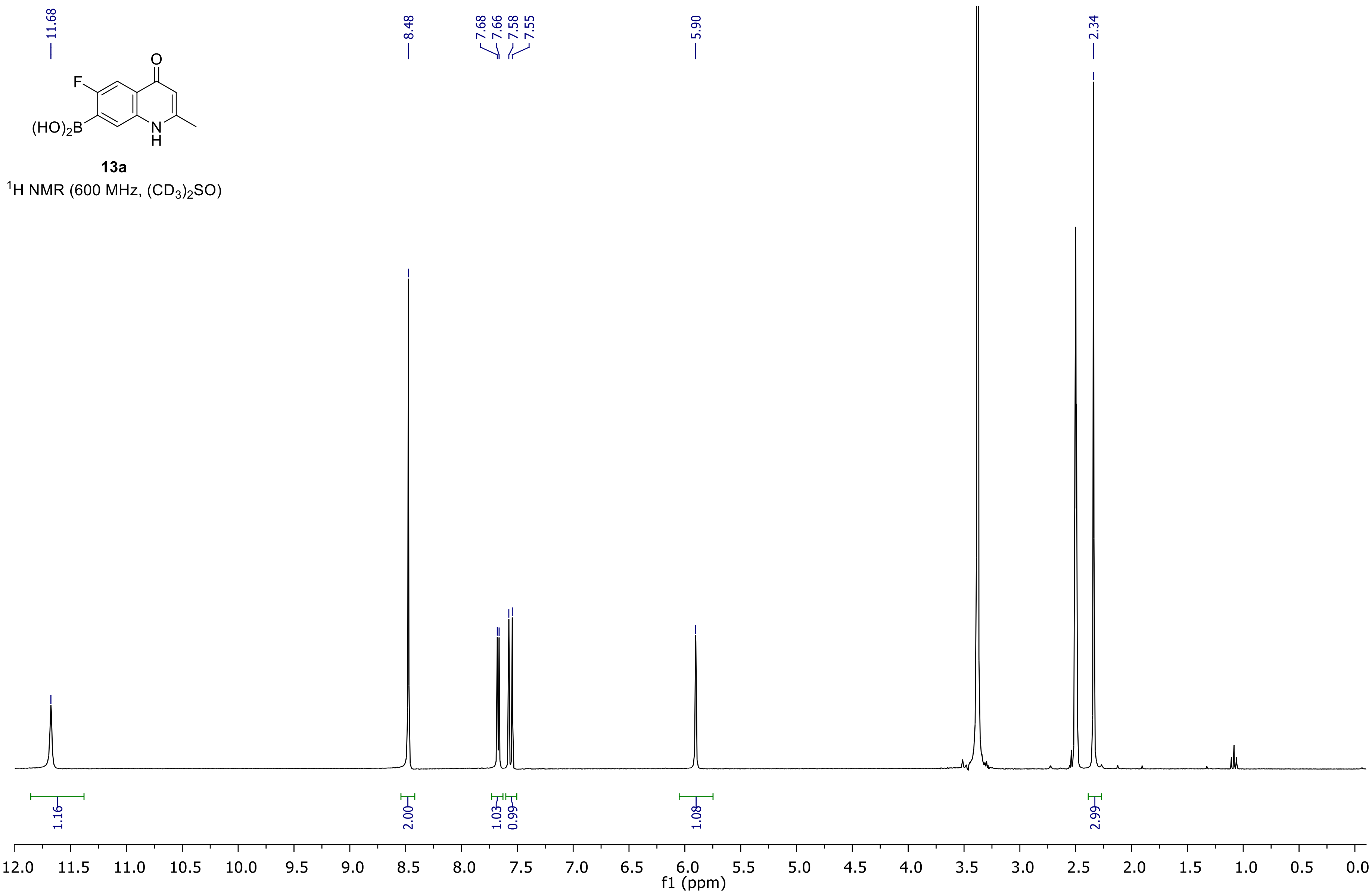
11.68
8.48
7.68
7.66
7.58
7.55
5.90
2.34
F
O
(HO)2B
N
H
13a
1H NMR (600 MHz, (CD3)2SO)
1.16
2.00
1.03
0.99
1.08
2.99
12.0
11.5
11.0
10.5
10.0
9.5
9.0
8.5
8.0
7.5
7.0
6.5
6.0
5.5
5.0
4.5
4.0
3.5
3.0
2.5
2.0
1.5
1.0
0.5
0.0
f1 (ppm)

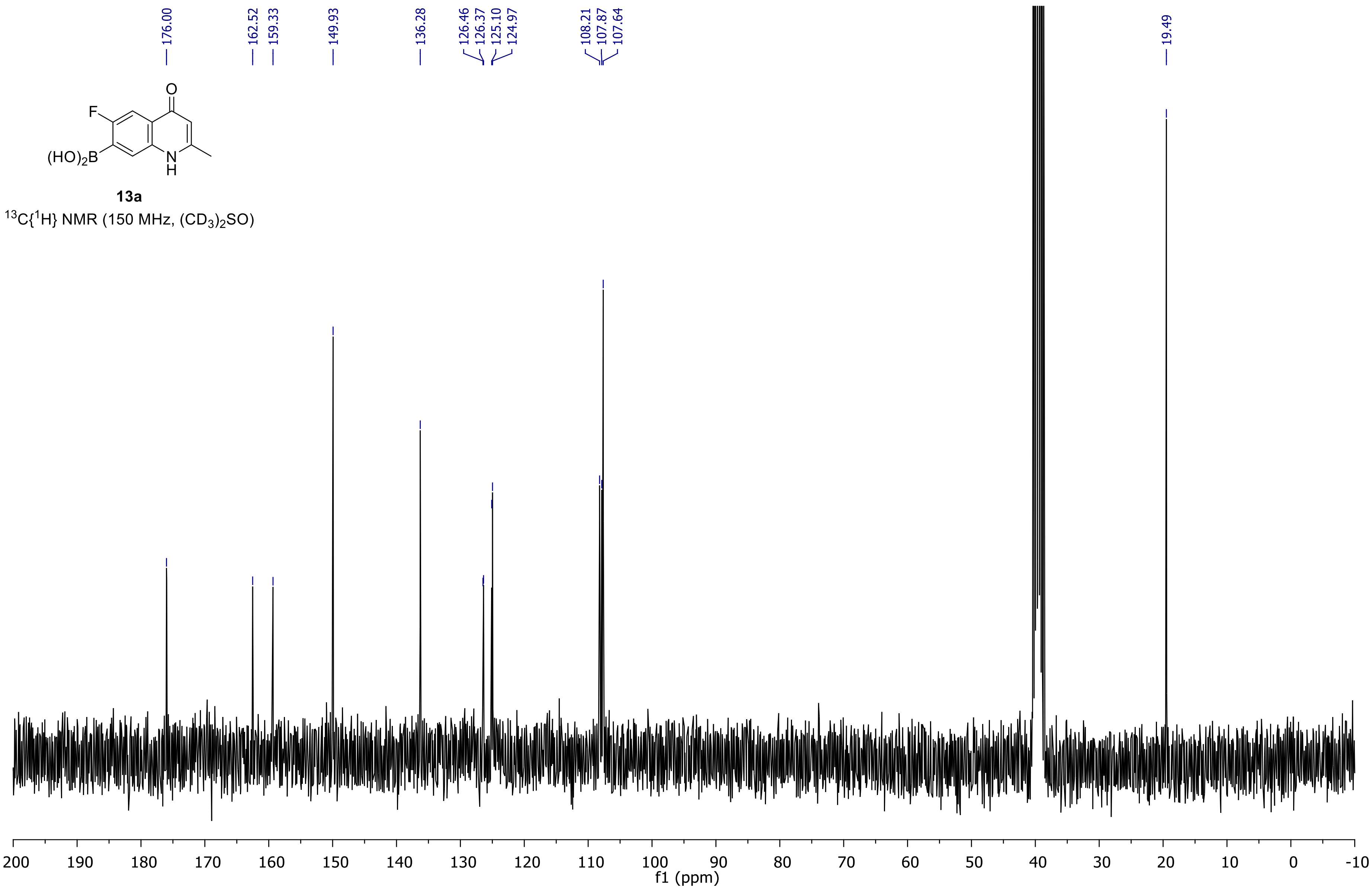

13a

$^{13}C\{^1H\}$ NMR (150 MHz, $(CD_3)_2SO$)

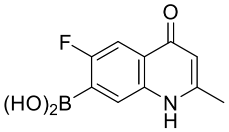

13a

^{19}F NMR (282 MHz, $(CD_3)_2SO$)

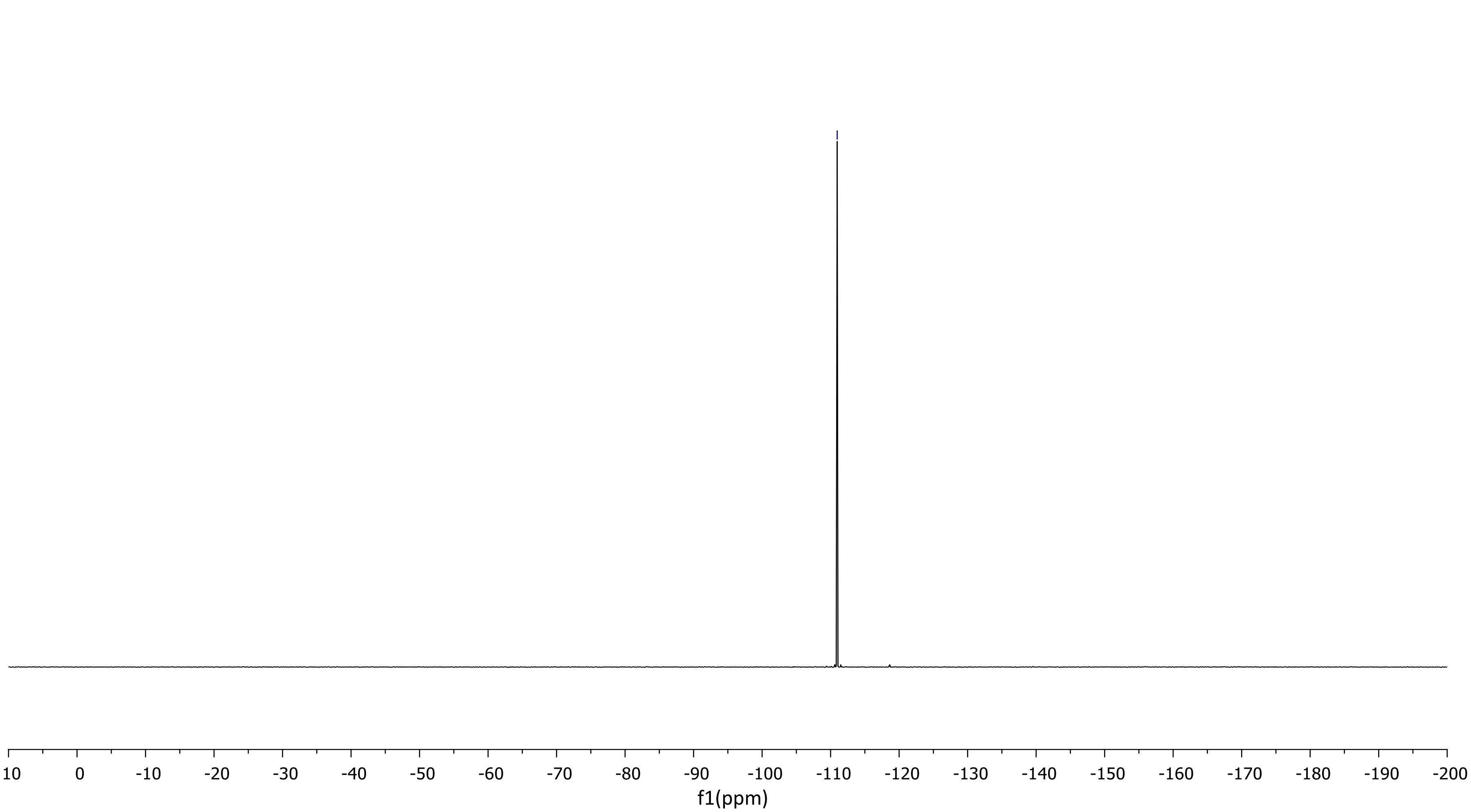

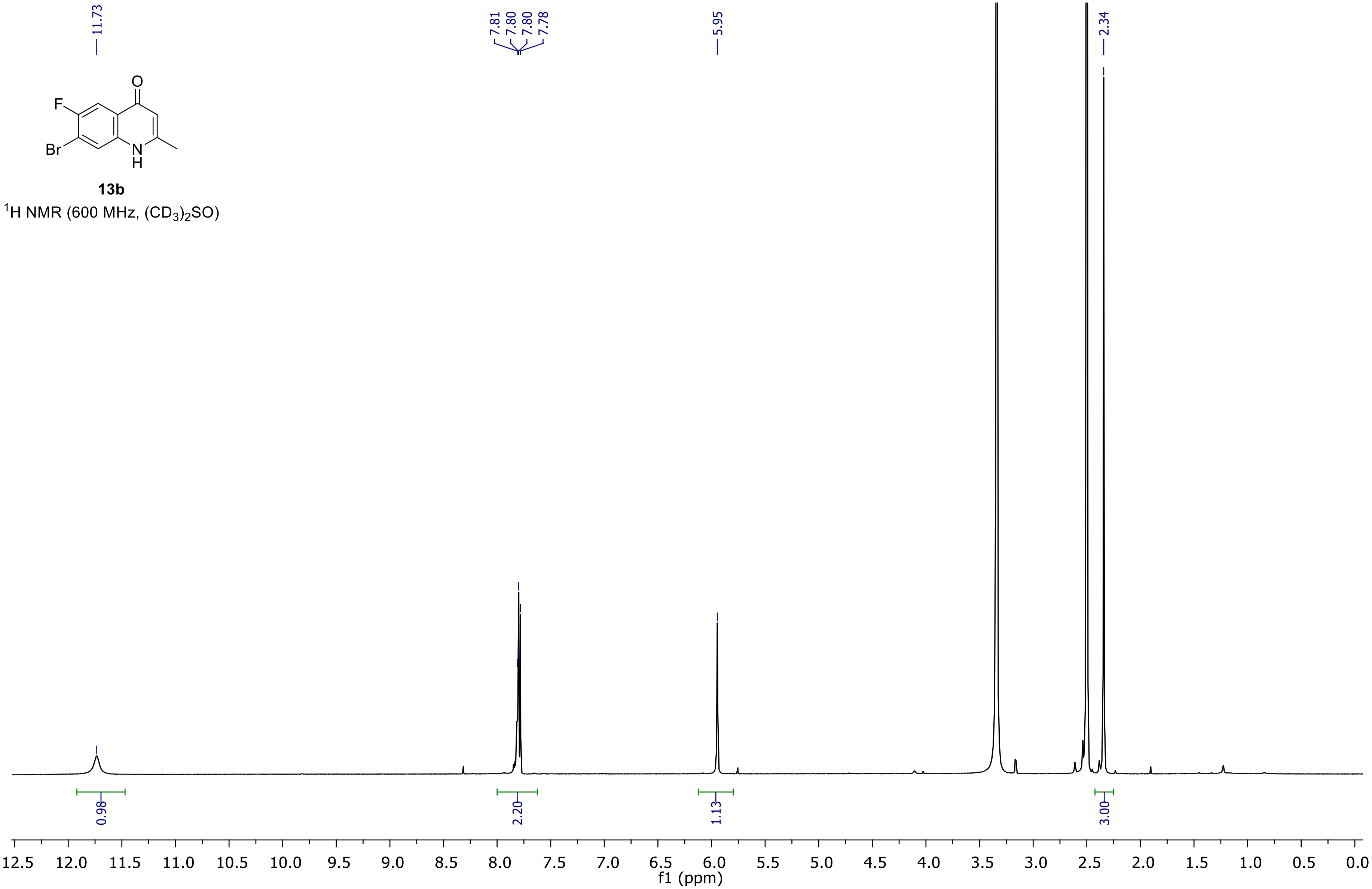

11.73
7.81
7.80
7.80
7.78
5.95
2.34
O
F
Br
N
H
13b
^{1}H NMR (600 MHz, $(CD_3)_2SO$)
0.98
2.20
1.13
3.00
12.5 12.0 11.5 11.0 10.5 10.0 9.5 9.0 8.5 8.0 7.5 7.0 6.5 6.0 5.5 5.0 4.5 4.0 3.5 3.0 2.5 2.0 1.5 1.0 0.5 0.0
f1 (ppm)

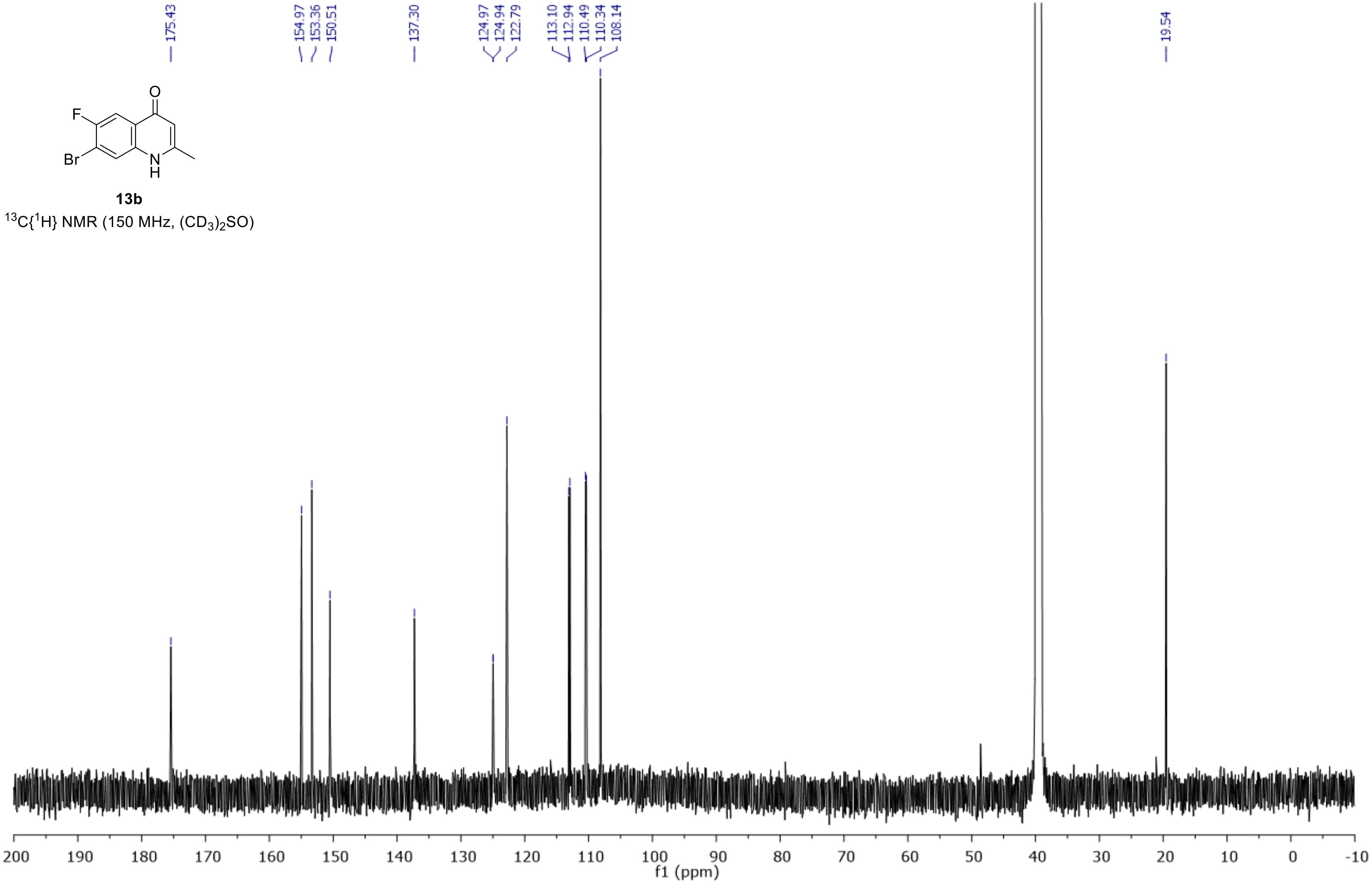
175.43
154.97
153.36
150.51
137.30
124.97
124.94
122.79
113.10
112.94
110.49
110.34
108.14
19.54
O
F
Br
N
H
13b
13C{1H} NMR (150 MHz, (CD3)2SO)
200 190 180 170 160 150 140 130 120 110 100 90 80 70 60 50 40 30 20 10 0 -10
f1 (ppm)

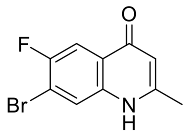

13b

^{19}F NMR (282 MHz, $(CD_3)_2SO$)

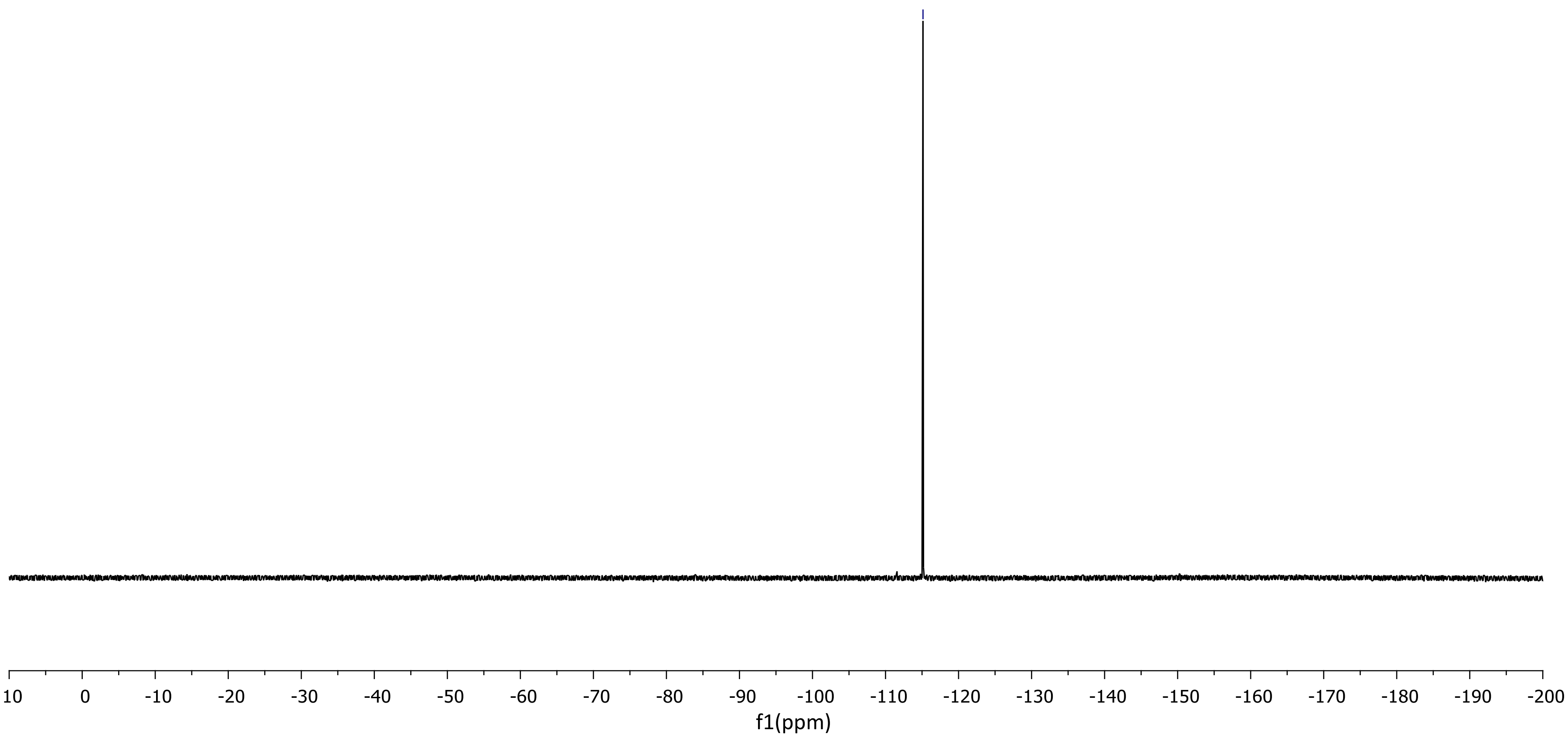

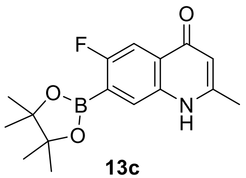

13c

^{1}H NMR (600 MHz, $(CD_3)_2SO$)

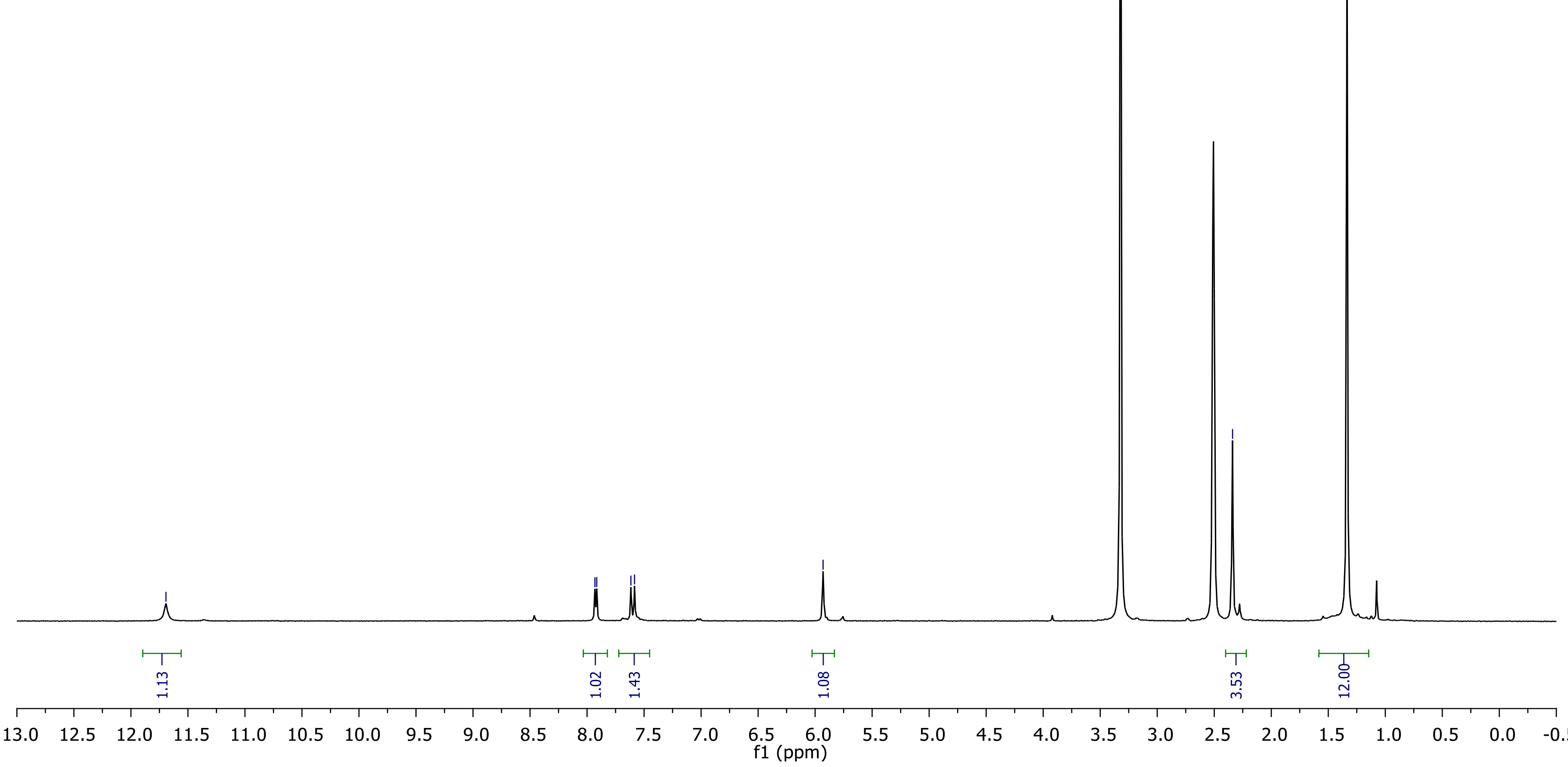

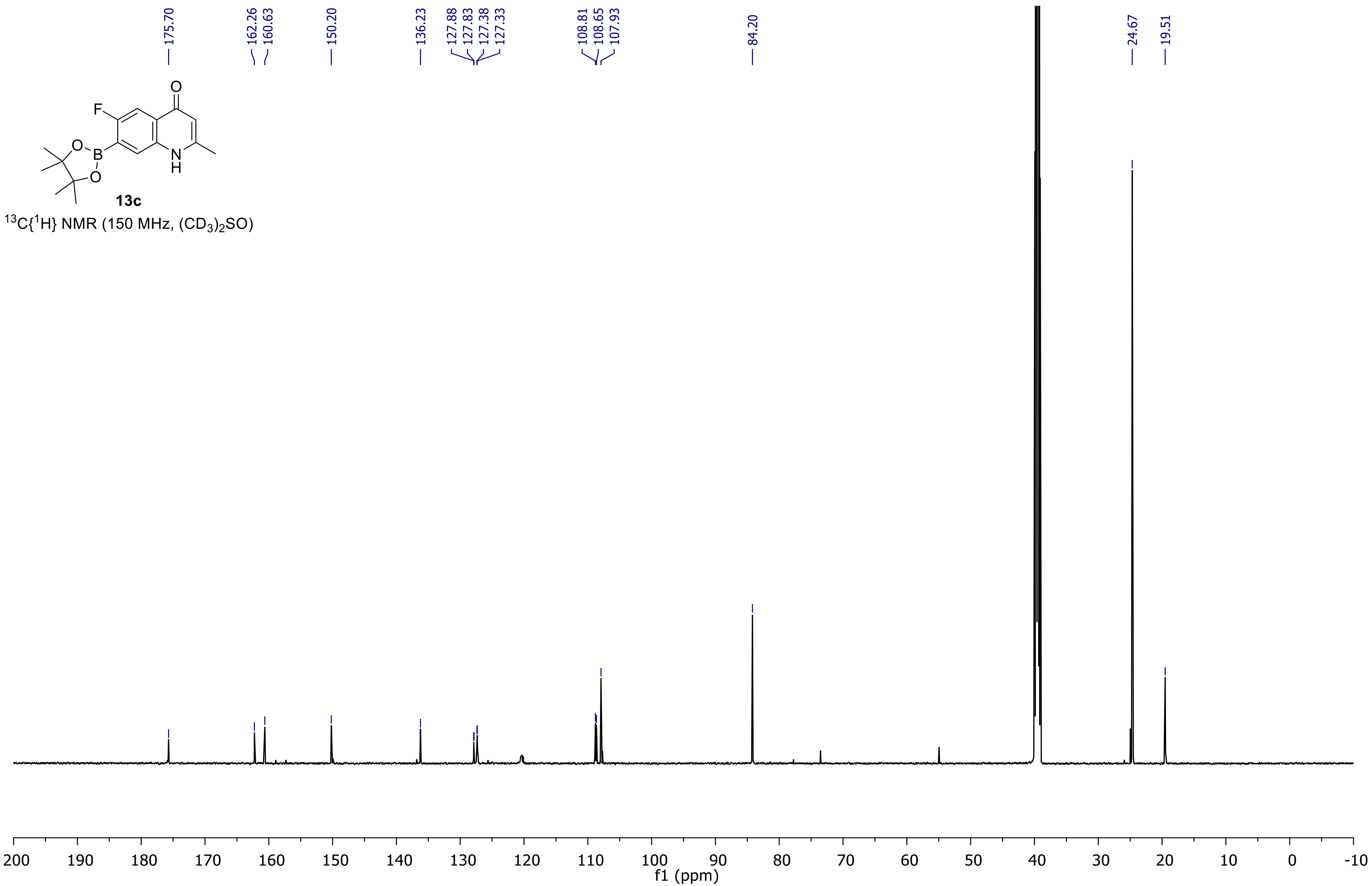
175.70
162.26
160.63
150.20
136.23
127.88
127.83
127.38
127.33
108.81
108.65
107.93
84.20
24.67
19.51
F
O
B
N
H
O
O
13c
$^{13}C\{^{1}H\}$ NMR (150 MHz, $(CD_3)_2SO$)
200 190 180 170 160 150 140 130 120 110 100 90 80 70 60 50 40 30 20 10 0 -10
f1 (ppm)

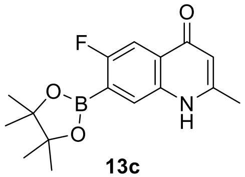

13c

^{19}F NMR (282 MHz, $(CD_3)_2SO$)

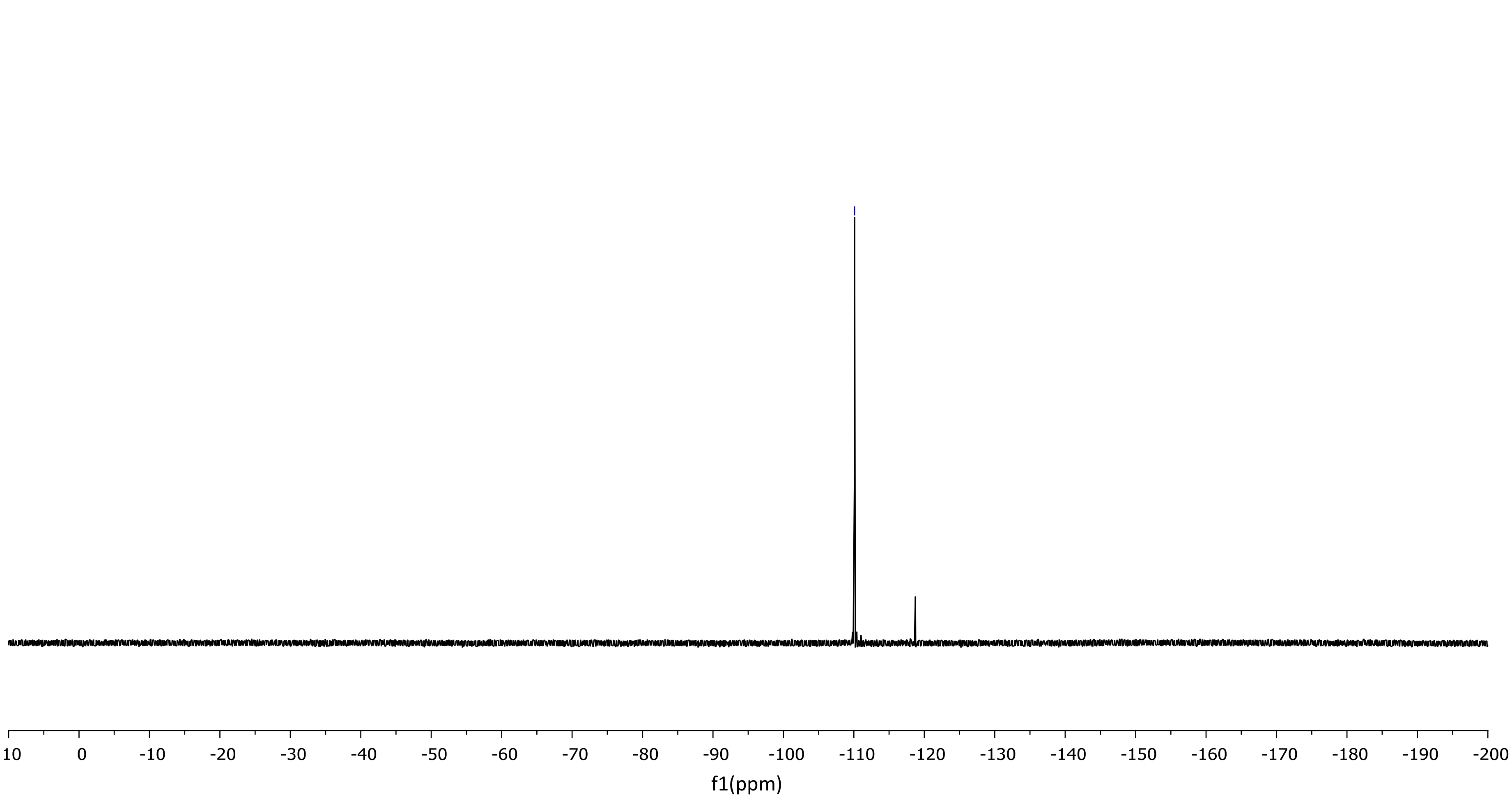

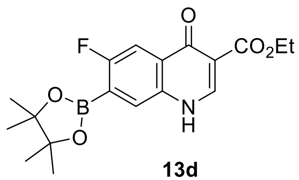

13d

^{1}H NMR (600 MHz, $(CD_3)_2SO$)

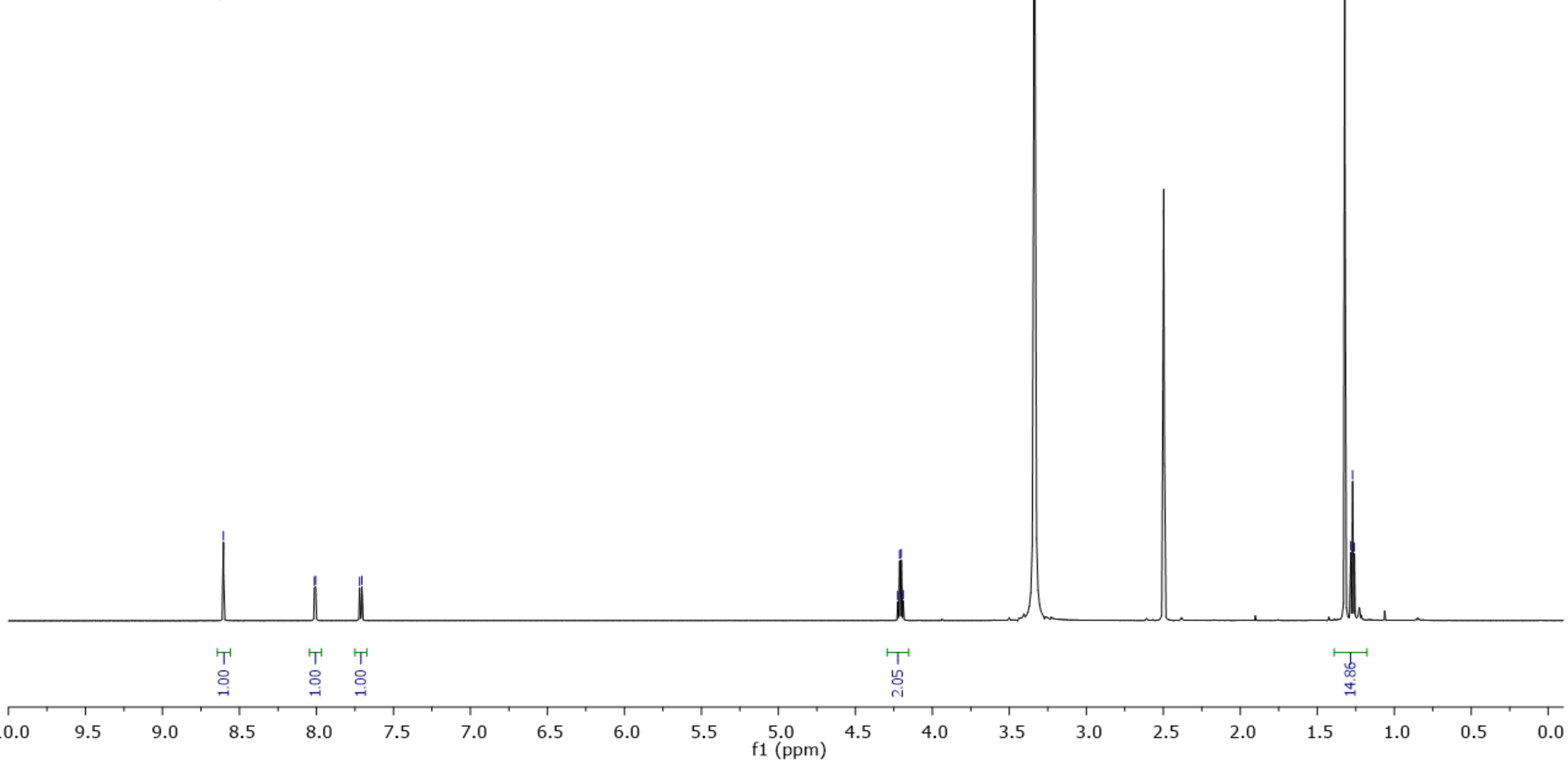

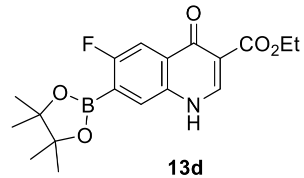

13d

$^{13}C\{^{1}H\}$ NMR (150 MHz, $(CD_3)_2SO$)

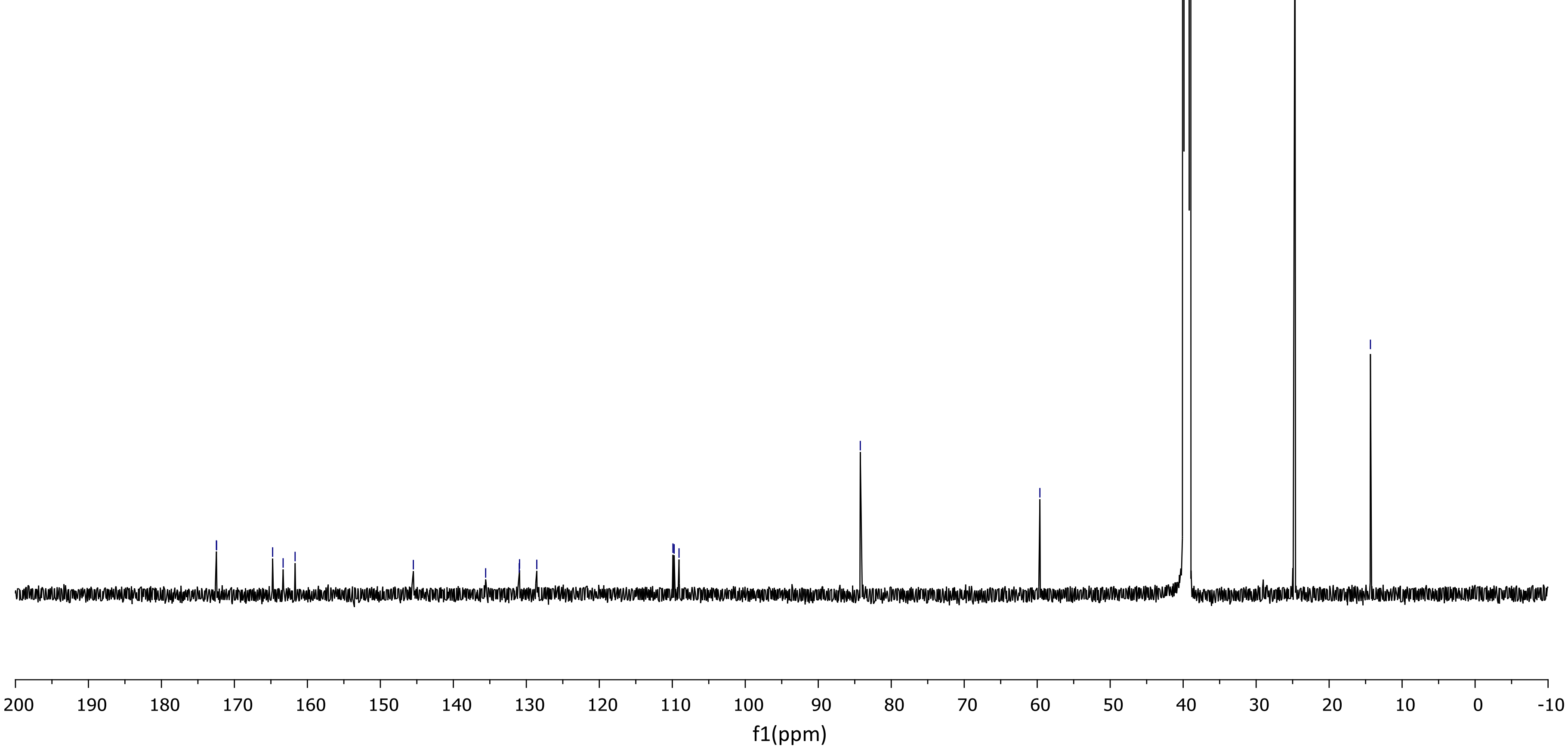

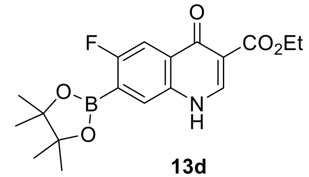

13d

^{19}F NMR (282 MHz, $(CD_3)_2SO$)

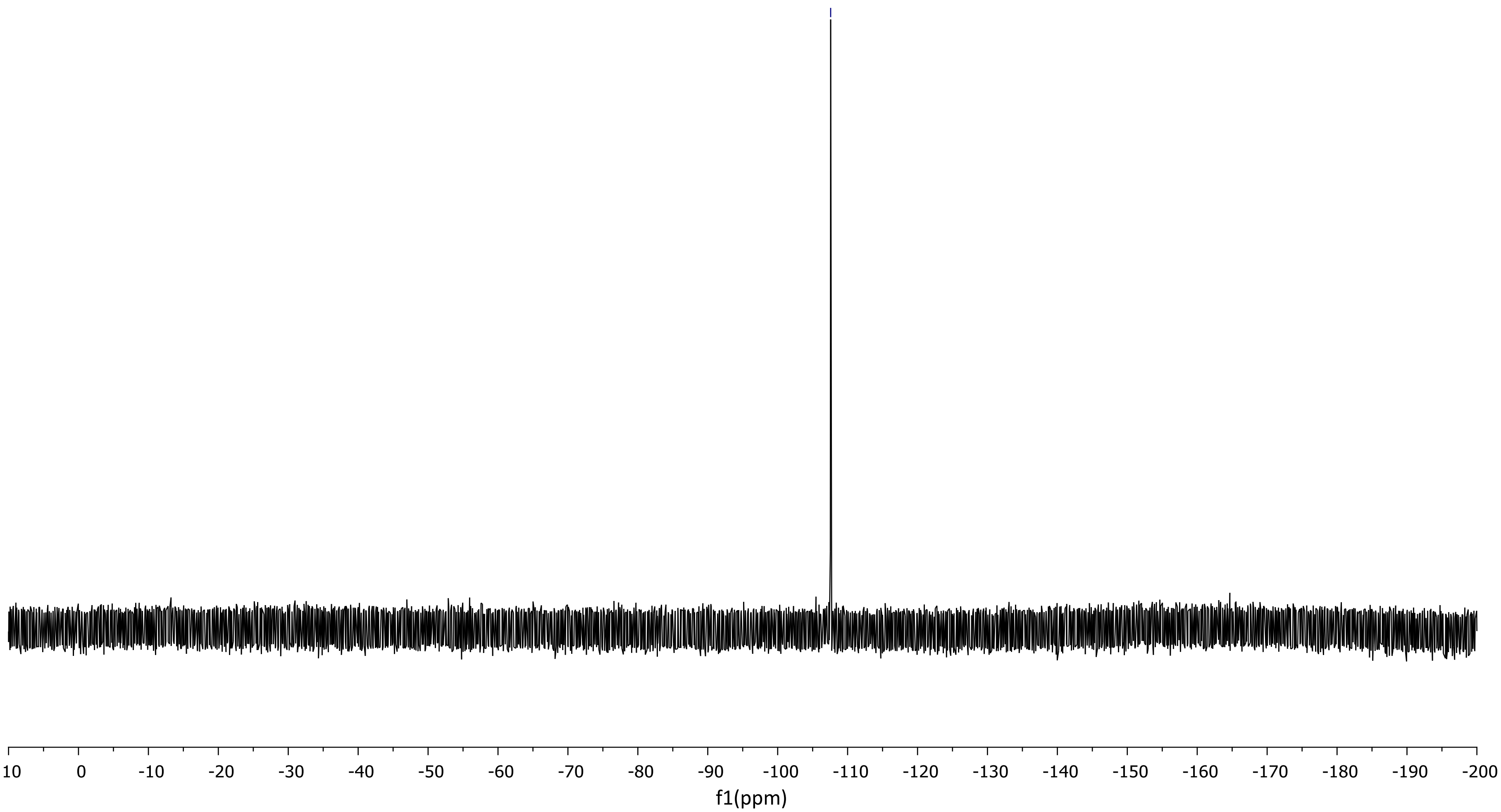

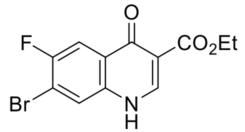

13e
^{1}H NMR (water suppression)
(600 MHz, $(CD_3)_2SO$, 313 K)

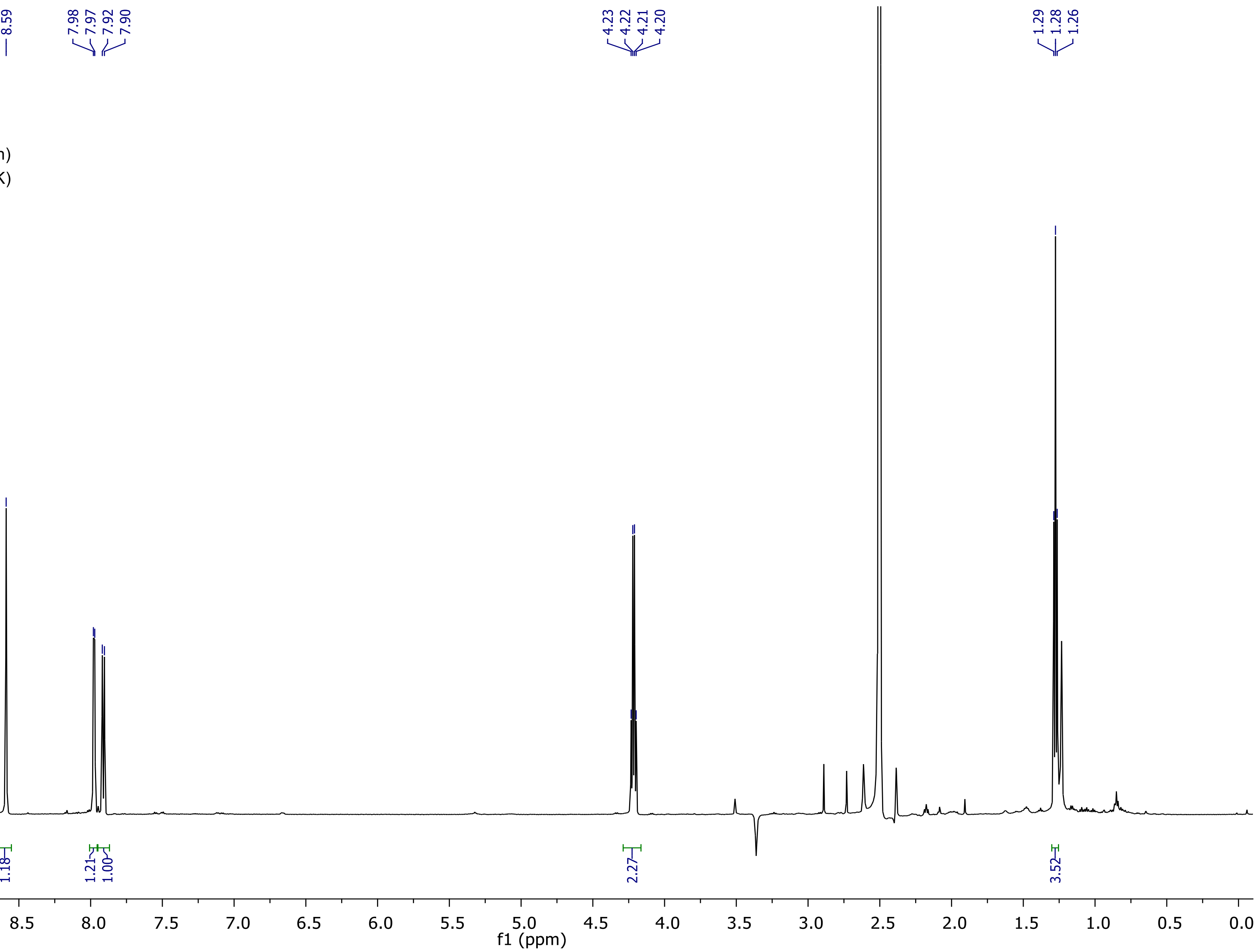

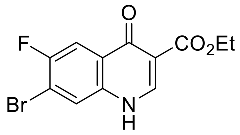

13e

$^{13}C\{^{1}H\}$ NMR (150 MHz, $(CD_3)_2SO$, 313 K)

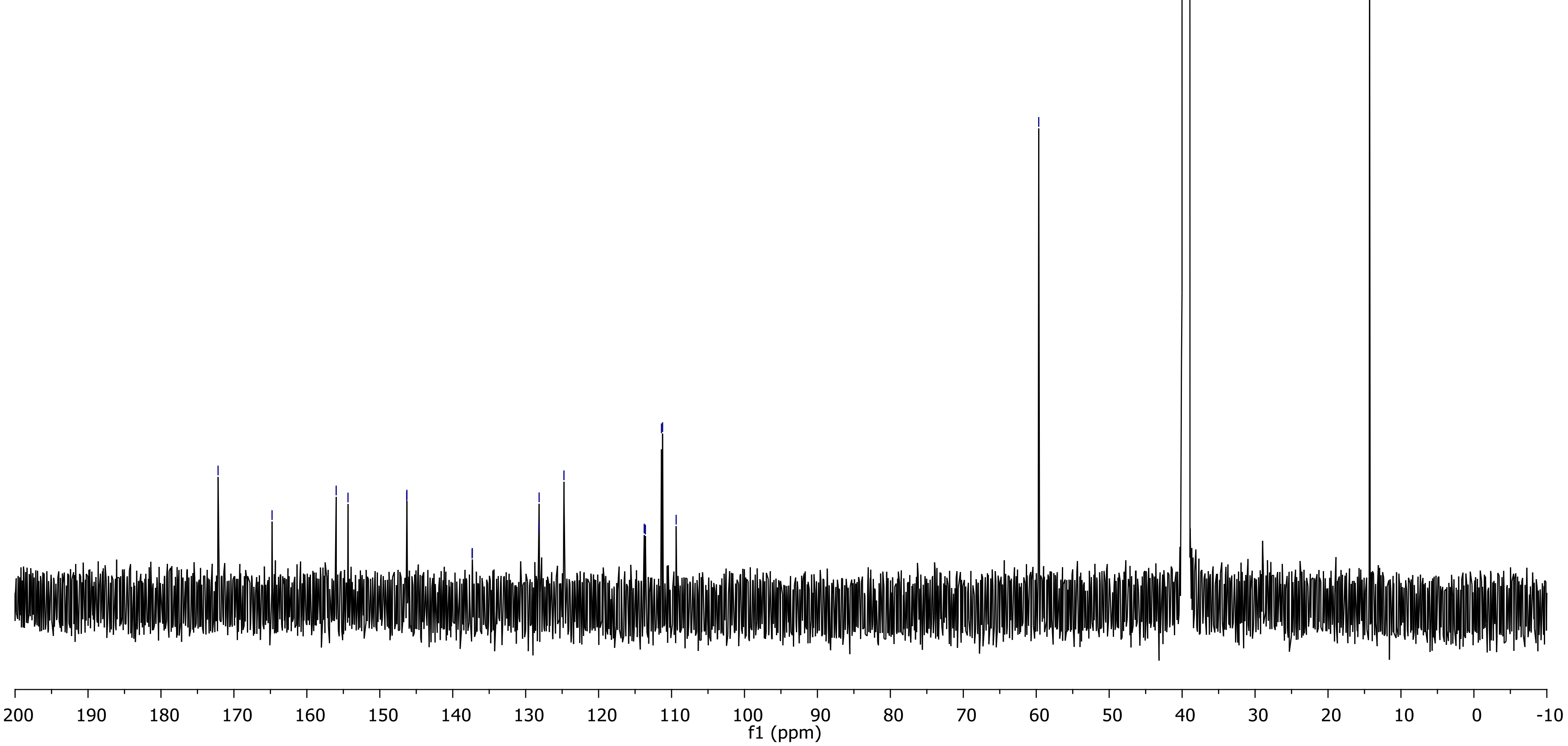

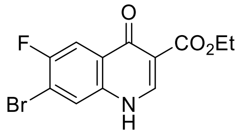

13e

^{19}F NMR (282 MHz, $(CD_3)_2SO$)

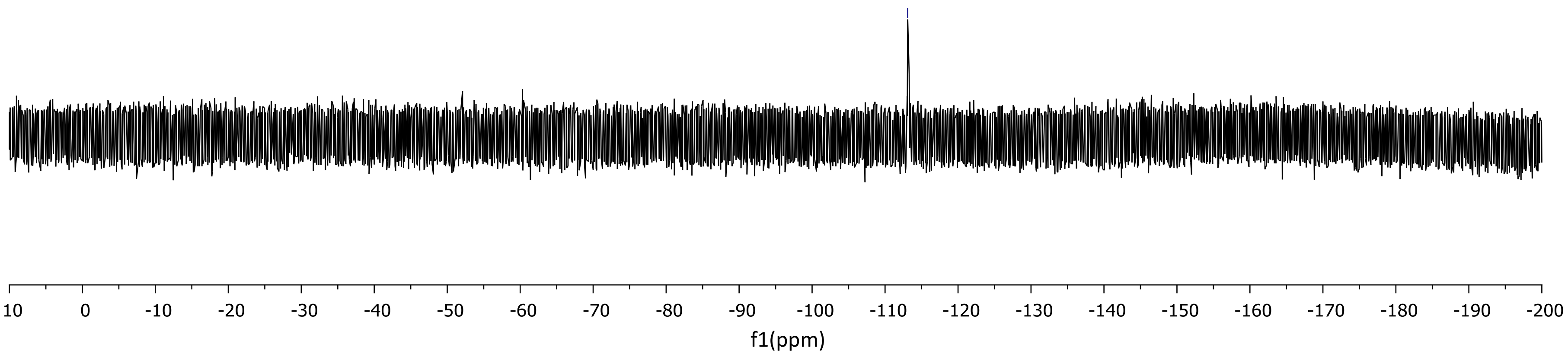

References

1. Larsen, M. A.; Hartwig, J. F., Iridium-Catalyzed C–H Borylation of Heteroarenes: Scope, Regioselectivity, Application to Late-Stage Functionalization, and Mechanism. *J. Am. Chem. Soc.* **2014,** *136* (11), 4287-4299.
2. Nayak, N.; Ramprasad, J.; Dalimba, U., Synthesis and Antitubercular and Antibacterial Activity of Some Active Fluorine Containing Quinoline–Pyrazole Hybrid Derivatives. *J. Fluor. Chem.* **2016,** *183*, 59-68.
3. Gale, D.; Wilshire, J., The Periodate Oxidation of Some Cycloalk[b]indoles and their N-Methyl Derivatives: Effect of Ring Size on the Spectral and Chemical Properties of the Resultant Lactams. *Aust. J. Chem.* **1974,** *27* (6), 1295-1308.
4. Kurasawa, Y.; Yoshida, K.; Yamazaki, N.; Iwamoto, K.; Hamamoto, Y.; Kaji, E.; Sasaki, K.; Zamami, Y., Quinolone Analogues 12: Synthesis and Tautomers of 2-Substituted 4-Quinolones and Related Compounds. *J. Heterocycl. Chem.* **2012,** *49* (6), 1323-1331.
5. Niedermeier, S.; Singethan, K.; Rohrer, S. G.; Matz, M.; Kossner, M.; Diederich, S.; Maisner, A.; Schmitz, J.; Hiltensperger, G.; Baumann, K.; Holzgrabe, U.; Schneider-Schaulies, J., A Small-Molecule Inhibitor of Nipah Virus Envelope Protein-Mediated Membrane Fusion. *J. Med. Chem.* **2009,** *52* (14), 4257-4265.
6. Sheldrick, G., SHELXT - Integrated Space-Group and Crystal-Structure Determination. *Acta Crystallogr. Sect. A, Found. Crystallogr.* **2015,** *71* (1), 3-8.
7. Sheldrick, G. M., Crystal Structure Refinement with SHELXL. *Acta Crystallogr. Sect. C, Struct. Chem.* **2015,** *71* (Pt 1), 3-8.
8. Hübschle, C. B.; Sheldrick, G. M.; Dittrich, B., ShelXle: A Qt Graphical User Interface for SHELXL. *J. Appl. Crystallogr.* **2011,** *44* (Pt 6), 1281-1284.
9. Spek, A. L., PLATON SQUEEZE: A Tool for the Calculation of the Disordered Solvent Contribution to the Calculated Structure Factors. *Acta Crystallogr. Sect. C, Struct. Chem.* **2015,** *71* (Pt 1), 9-18.
10. Brandenburg, K. *Diamond (version 4.6.7), Crystal and Molecular Structure Visualization, Crystal Impact GbR*, Putz, H., Brandenburg, K.: Bonn, Germany, 2022.
